# Phosphotyrosine phosphatase R3 receptors: Origin, evolution and structural diversification

**DOI:** 10.1371/journal.pone.0172887

**Published:** 2017-03-03

**Authors:** Javier U. Chicote, Rob DeSalle, Antonio García-España

**Affiliations:** 1 Hospital Universitari de Tarragona Joan XXIII, Institut d’Investigació Sanitària Pere Virgili, Universitat Rovira i Virgili, Tarragona, Spain; 2 Sackler Institute for Comparative Genomics, American Museum of Natural History, New York, NewYork, United States of America; Laboratoire de Biologie du Développement de Villefranche-sur-Mer, FRANCE

## Abstract

Subtype R3 phosphotyrosine phosphatase receptors (R3 RPTPs) are single-spanning membrane proteins characterized by a unique modular composition of extracellular fibronectin repeats and a single cytoplasmatic protein tyrosine phosphatase (PTP) domain. Vertebrate R3 RPTPs consist of five members: PTPRB, PTPRJ, PTPRH and PTPRO, which dephosphorylate tyrosine residues, and PTPRQ, which dephosphorylates phophoinositides. R3 RPTPs are considered novel therapeutic targets in several pathologies such as ear diseases, nephrotic syndromes and cancer. R3 RPTP vertebrate receptors, as well as their known invertebrate counterparts from animal models: PTP52F, PTP10D and PTP4e from the fruitfly *Drosophila melanogaster* and F44G4.8/DEP-1 from the nematode *Caenorhabditis elegans*, participate in the regulation of cellular activities including cell growth and differentiation. Despite sharing structural and functional properties, the evolutionary relationships between vertebrate and invertebrate R3 RPTPs are not fully understood. Here we gathered R3 RPTPs from organisms covering a broad evolutionary distance, annotated their structure and analyzed their phylogenetic relationships. We show that R3 RPTPs (i) have probably originated in the common ancestor of animals (metazoans), (ii) are variants of a single ancestral gene in protostomes (arthropods, annelids and nematodes); (iii) a likely duplication of this ancestral gene in invertebrate deuterostomes (echinodermes, hemichordates and tunicates) generated the precursors of PTPRQ and PTPRB genes, and (iv) R3 RPTP groups are monophyletic in vertebrates and have specific conserved structural characteristics. These findings could have implications for the interpretation of past studies and provide a framework for future studies and functional analysis of this important family of proteins.

## Introduction

Phosphotyrosine phosphatases, along with protein tyrosine kinases, regulate the levels of phosphotyrosine modification in cells [1, 2]. Phosphotyrosine phosphatases play a major role in tuning cell function, and are considered therapeutic targets since their deregulation leads to health disorders including cancer [2–5].

**Fig 1:**
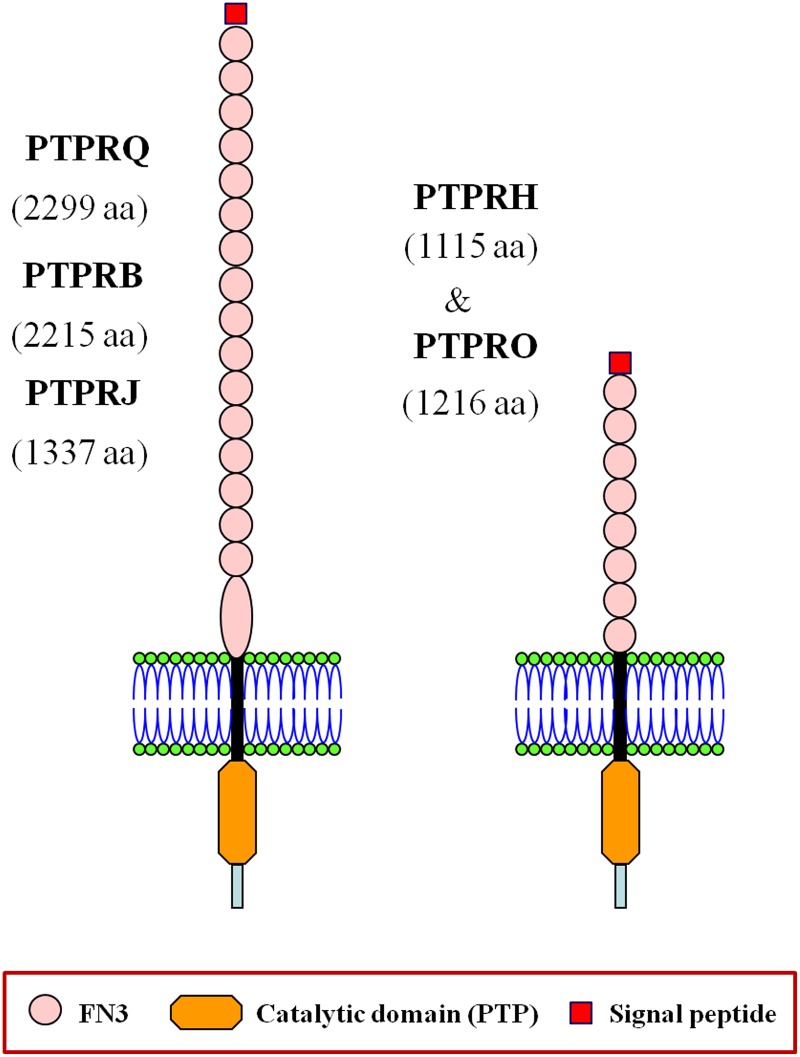
Domain architecture of human R3 RPTP members. Schematic representation of human R3 subtype RPTP protein members. For catalytic PTP, FN3 and signal peptide symbols see the figure. Black and light blue boxes represent the transmembrane segments and the cytoplasmatic regions after the PTP domain, respectively. Note the larger size of the juxtamembrane FN3 domain in PTPRQ, PTPRB and PTPRJ proteins [15]. Protein amino acid numbers are indicated in parenthesis below the protein names.

Receptor phosphotyrosine phosphatases (RPTPs) are single-spanning multidomain membrane proteins classified by their different domain compositions into eight subtypes (R1-R8) [6]. Subtype R3 RPTP members are characterized by a unique modular composition consisting of multiple extracellular fibronectin type III (FN3) repeats and a single (most RPTP subtypes have two) intracytoplasmic protein tyrosine phosphatase (PTP) domain [6, 7]. In vertebrates, R3 RPTPs comprise five members: PTPRB (VE-PTP), PTPRJ (DEP-1), PTPRH (SAP-1), PTPRO (GLEPP1) and PTPRQ (PTPS31) (Fig 1) [6, 8, 9]. These proteins catalyze the dephosphorylation of phosphotyrosine residues, except for PTPRQ, which dephosphorylates phosphatidylinositide substrates [10, 11]. The specific involvement of vertebrate R3 RPTPs in cancer and other pathologies has been the subject of several recent reports and reviews [1, 4, 12, 13]. In invertebrates, R3 RPTPs have been functionally characterized in protostome animal models: PTP52F, PTP10D and PTP4e in the fruitfly *Drosophila melanogaster* and F44G4.8/DEP-1 (nematode.2 sequence in this study) in the nematode *Caenorhabditis elegans* (reviewed by Jeon and Zinn, 2015) [14].

Both vertebrate and invertebrate R3 RPTPs are involved in the control of a variety of cellular processes, including cell growth, differentiation, mitotic cycle and oncogenic transformation [14, 16]. Despite displaying a related set of cellular activities, the evolutionary relationships between invertebrate and vertebrate R3 RPTP proteins are not fully resolved. In this study, by analyzing R3 RPTP sequences of vertebrate and invertebrate organisms, we show that R3 RPTPs most likely originated in the common ancestor of animals and underwent two waves of diversification in deuterostomes. Most likely, a duplication of an ancestral protoPTPRB gene that took place in the common ancestor of all deuterostomes (echinoderms, hemichordates, tunicates and vertebrates) generated protoPTPRQ, and a second wave of duplication in the common ancestor of all vertebrates generated PTPRO, PTPRJ and PTPRH. Our analysis of the evolution and structural diversification of R3 phosphatases will be useful to understand the structure and function of these proteins relevant to human health and disease.

## Material and methods

### Data mining and sequence analysis

Data mining was performed as previously described by us [15, 17–20] and others [21].We searched for R3 subtype PTPR sequences in scientific reports [9, 22–24], in the PFAM protein family database [25], and in online genomic and proteomic databases [21]. All sequences used in this study were checked for errors and curated manually. Protein sequences, organisms’ scientific names, accession numbers and structural characteristics are shown in S1 File.

The SMART server (http://smart.embl-heidelberg.de/smart/set_mode.cgi?GENOMIC=1) [26] and the NCBI CDD database [27] were used to determine the domain composition of the proteins. Intron-exon borders were determined as in Garcia-España et al., 2009 [28] using the “align two sequences” option of the NCBI BLAST program (www.ncbi.nlm.nih.gov). Splice consensus signals were then manually annotated. All sequences used in this study are listed in S1 File.

Alignments of protein sequences were performed using the MAFFT server (http://mafft.cbrc.jp/alignment/server/) or the ClustalW and the Multalin programs at the NPS@: Network Protein Sequence Analysis (http://npsa-pbil.ibcp.fr/cgi-bin/npsa_automat.pl?page=/NPSA/npsa_server.html). The NCBI “align two sequences” TBLASTN program was used in protein profiling.

Tyrosine phophorylation was assessed with the program NetPhos 2.0 (http://www.cbs.dtu.dk/services/NetPhos/) and furin cleavage prediction was performed with the ProP 1.0 Server (http://www.cbs.dtu.dk/services/ProP/).

### PTP sequence similarity determiantion

Human PTPRO, PTPRQ, PTPRB, PTPRJ and PTPRH, and invertebrate R3 RPTP sequences were Blasted with default parameters at the NCBI protein blast server database against the non-redundant protein databases of human (taxid:9606), chicken (taxid:9031) or zebrafish (taxid:7955) organisms.

### Phylogenetic analyses

Due to the high variability in FN3 repeats, and hence difficulties in obtaining meaningful alignments, phylogenetic trees were generated using the highly conserved PTP catalytic domain sequences as indicated [21]. The PTP protein domain of all invertebrate R3 RPTP sequences and of human PTPRO, PTPRQ, PTPRB, PTPRJ and PTPRH were obtained as outlined above for the following vertebrates: *Homo sapiens*, *Mus musculus*, *Gallus gallus*, *Xenopus tropicalis*, and *Danio rerio*. Subtype R3 PTPR sequences were obtained from the following invertebrate organisms organisms: *Capitella teleta*, *D*. *melanogaster*, *C*. *elegans*, *Ciona intestinallis*, *C*. *Savignyi*, *Haemonchus contortus*, *Saccoglossus kowalevskii*, *Oikopleura dioica*, *Strongylocentrotus purpuratus* and *Amphimedon queenslandica*. A FASTA file of the protein and DNA sequences for these taxa was created. The protein sequences were aligned using MAFFT at gap costs of 1, 2, 4, 6, 8, and 16, and all other parameters left at default. The alignments obtained in this way were elided according to the protocol of Wheeler et al., 1995 [29]. The elided matrix was then partitioned according to the gap costs and this matrix is included as S2 File. The DNA sequences were aligned using TranslatorX, a program that uses an amino acid alignment as a guide for the DNA sequence alignment. This matrix is included in S3 File.

Maximum Parsimony (using PAUP*; Swofford, 2003 [30]), Maximum Likelihood (using RaxML BlackBox; [31]) and Bayesian Phylogenetic (using MrBayes; [32]) inference were used to generate phylogenetic hypotheses for the PTP domain sequences. Bootstrap analysis was performed with Maximum Parsimony using TBR branch swapping and 100 random addition searches for 1000 replicates. Bayesian analysis of protein sequences used the WAG model, with gamma distribution and invariants for 1,000,000 generations. Bayesian analysis of DNA sequences used the GTR model, with gamma distribution and invariants for 1,000,000 generations. Trees generated using these approaches are shown in S1 Fig.

## Results

### Origin and characterization of R3 RPTPs in metazoans

To study the evolution of R3 RPTPs, we retrieved DNA and protein sequences with specific subtype R3 characteristics (see methods section) from vertebrates (human, mouse, chicken, frog and zebrafish), invertebrate deuterostomes (sea urchins, acorn worms and cionas), protostomes (annelids, flies and nematodes), and from early divergent metazoans (sponges). Despite extensive searches of genome and protein databases we could not find any orthologue of R3 RPTPs genes in amphioxus (*Branchiostoma floridae*) another deuterostome, or in other organisms that diverged before the split of duterostomes and protostomes: cinidarians (*Nematostella vectensis*, *Hydra vulgaris*, and *Hydra magnipapillata*) and placozoans (*Trichoplax adhaerens*). Aditionally we used in our analysis a RPTP sequence with only FN3 domains but with two PTP domains from capsaspora and RPTP sequences from subtype R2A (PTPRF) and subtype R5 (PTPRG) of human origin. All protein sequences, organism scientific names, structural characteristics and accession numbers are shown in S1 File.

**Fig 2:**
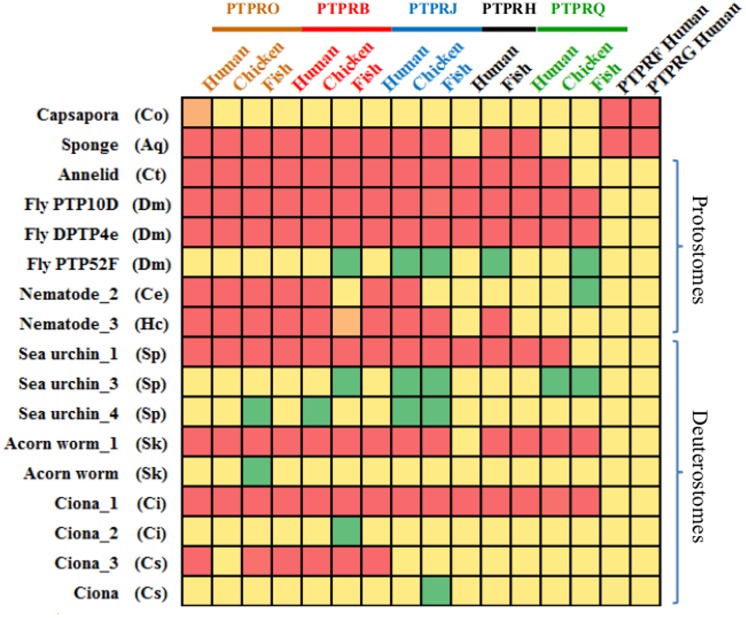
Invertebrate and vertebrate PTP domain similarity. Heat map representing color-coded blastp E value of PTP domains of R3 PTPRs from vertebrates and inverebrates deuterostomes, protostomes and sponges. Note the strong similarity of the sponge PTP sequence with PTPRF and PTPRG. E values are coloured from green (low similarity) to red (high similarity) (See S1 Table for numerical E values; 100% similarity corresponds to an E value of 0.0). Full scientific names of species are: Co, *Capsaspora owczarzaki*; Aq, *Amphimedon queenslandica*; Ct, *Capitella teleta*; Dm, *Drosophila melanogaster*; Ce, *Caenorhabditis elegans*; Hc, *Haemonchus contortus*; Sp, *Strongylocentrotus purpuratus*; Sk, *Saccoglossus kowalevskii*; Cs, *Ciona Savigny*; Ci, *Ciona intestinalis*; Hs, *Homo sapiens*. Other sequences Chicken (Gg) *Gallus gallus*; Fish (Dr) *Danio rerio*.

The results of our searches placed the probable origin of R3 RPTPs in the common ancestor of animals (metazoans) since no R3 subtype sequences were present in organisms which diverged early than sponges, including *Monosiga* and *Capsaspora*, the closest protists to the animal kingdom [33,34]. Although a phosphatase with only FN3 repeats exists in Capsaspora, it has two PTP domains [34]. Moreover, its catalytic PTP domain, the one closest to the transmembrane helix, is more similar to that of PTPRF (subtype R2A) and PTPRG (subtype R5) than to R3 subtype members (Fig 2 and S1 Table). The sponge (*Amphimedon queenslandica*) was the most early diverged organism in which we retrieved a sequence with full R3 subtype characteristics. Interestingly, the PTP domain of this sponge sequence, in addition to showing high similarity with the vertebrate members of the R3 subtypes PTPRO and PTPRB, was also strongly similar to the catalytic PTP domains of PTPRF and PTPRG of the R2A and R5 subtypes, respectively (Fig 2 and S1 Table). The analysis indicated that only one type of R3 sequence exists in protostomes (arthropods, annelids, nematodes). All protostome RPTP sequences recognized with the lowest E values PTPRB and PTPRO (Fig 2 and S1 Table), except for PTP52F, which is highly divergent and is present only in flies (S2 Table). By contrast, the sequences of invertebrate deuterostomes echinoderms (sea urchin), hemichordates (acorn worm) and tunicates (ciona), seemed to be of two types: one type that also recognizes PTPRB and PTPRO with high statistical significance (Sea urchin_1, Acorn worm_1, Ciona_1 and Ciona_3) and a second sequence type that recognizes all R3 vertebrates sequences with low significance (Sea urchin_3, Sea urchin_4, Acorn worm, Ciona and Ciona_2) (Fig 2 and S1 Table).

### Phylogenetic analyses indicate two waves of R3 RPTP diversification in deuterostomes

**Fig 3:**
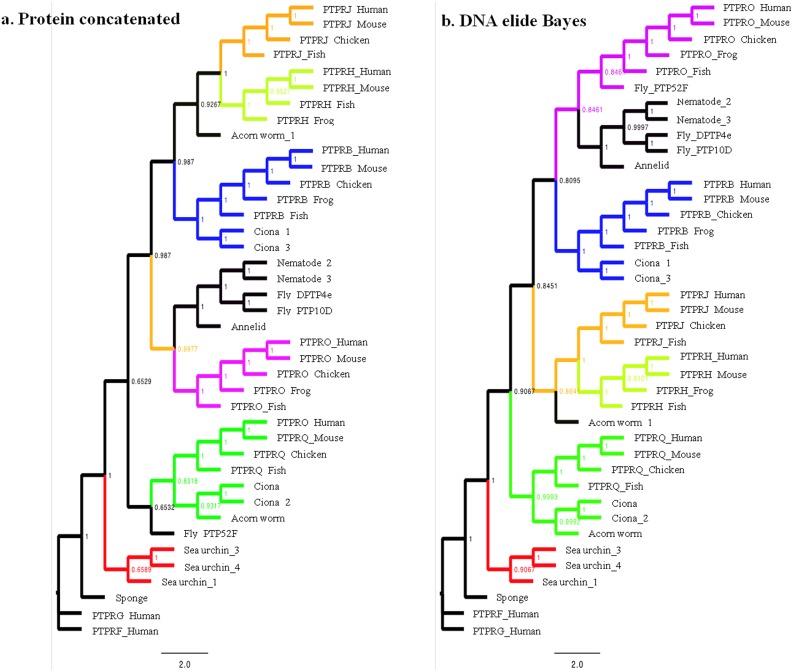
Protein and DNA trees showing the evolutionary relationships between RPTPs. A) Bayes protein concatenated tree (see text for description of model parameters and analysis specifics). Values at the nodes in the tree indicate the Bayesian posterior probability for that node. B) DNA elide Bayes tree (see text for description of model parameters and analysis specifics). Values at the nodes in the tree indicate the Bayesian posterior probability for that node. Protein and DNA parsimony, and additional Bayesian phylogenetic trees are included in S2 File.

Because of the different number of FN3 repeats between the sequences, phylogenetic trees were generated using PTP domain sequences [6]. The phylogenetic trees generated using PTP DNA and protein sequences from representative vertebrate and invertebrate organisms are summarized in Fig 3 and all analyses are presented in S1 Fig.

The following general statements can be made about the phylogenetic analyses of these sequences: (i) the predetermined RPTP groups (B, H, J, Q and O) are all individually monophyletic for the vertebrates in the analysis at high bootstrap and Bayesian posteriors; (ii) PTPRJ and PTPRH are closely related and seem to be each other’s sister; (iii) protostome sequences are also monophyletic with the exception of PTP52F, but sequences resembling PTP52F are found only in flies (S2 Table) thus its placement outside the protostome cluster its probably due to its high sequence divergence (see below); (iv) the sponge sequence appears early diverged in comparison to all other sequences; (v) the ascidian tunicate sequences Ciona_1 (*C*. *intestinalis*) and Ciona_3 (*C*. *savignyi*) cluster with PTPRB and Ciona_2 (*C*. *intestinalis*) and Ciona (*C*. *savignyi*) with PTPRQ, pointing to the ocurrence of duplications in early diverged deuterostomes that generate protoPTPRQ and PTPRB genes; and (vi) the PTPRB cluster is more closely related to PTPRO, PTPRJ, PTPRH and protostome clusters than to the PTPRQ cluster.

### Conservation of carboxy terminus tyrosine phophorylation

**Fig 4:**
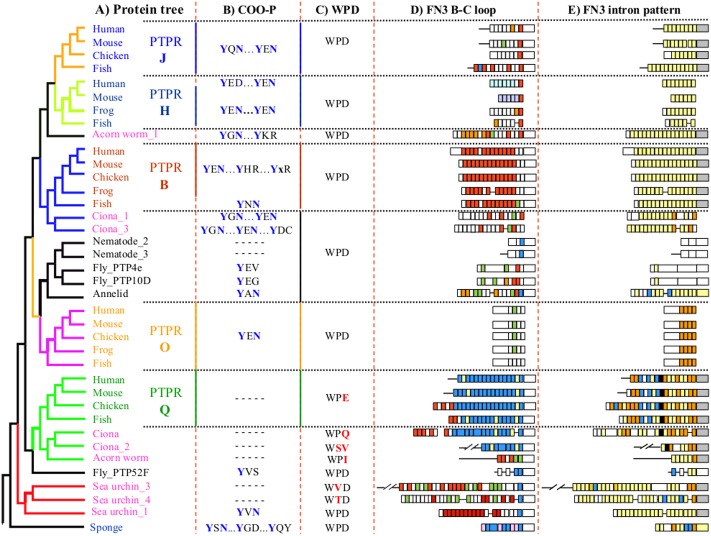
Structural characteristics and evolution of vertebrate and invertebrate R3 RPTPs. A) Protein tree from Fig 3 with metazoan sequences. The vertebrate clusters of R3 RPTPs are indicated by vertical lines of different colours. B) Carboxy terminus predicted tyrosine phosphorylation sites. Blue bold Y and N letters represent, respectively, the tyrosine predicted phosphorylation residue and the asparagine residue of the YxN motif of RPTP phosphorylation sites in some mammalian proteins [14]. Small x in PTPRB prediction sites represents any amino acid. Dashed horizontal lines indicate negative phosphorylation prediction. C) Aspartic acid modifications in the WPD loop of the catalytic domain. Divergent amino acids from the canonical WPD motif residues are indicated in red bold letters. The replacement of WPD with WPE in PTPRQs changes their substrate specificity from phospho tyrosine to inositol phosphate [11]. D) FN3 B-C loops. The distances between the loop flanking tryptophan (W) and tyrosine (Y) residues and the distance to a conserved glycine residue inside the loop were coded with different colors and plotted in the tree. Codes are of the form WnGnY where n indicates the number of amino acid residues between the W-G or G-Y residues. The alignments of the FN3 domains are provided in S2 Fig. Colour codes are: red, W4G4Y; orange W4G5Y; light blue W4G7Y; green W5G4Y; yellow W7G3Y; dark blue W7G4Y; lavender W9G2Y; and pink W9G4Y. White colour represents different codes from the above codes. E) FN3 intron/exon structure. FN3 domains flanked by two introns in phase 1 (1:1) are represented as yellow squares; with an additional intron inside the domain in phase 0 as orange squares (1:0:1), in phase 1 as blue squares (1:1:1) or in phase 2 as black squares (1:2:1). The larger juxtamembrane FN3 domains (1:1:2:1:2) are represented by grey squares. White squares indicate FN3 domains with intron codes different from the above. Absence of FN3 flanking introns is indicated by absence of the vertical sides of the squares.

To better understand how R3 RPTPs have diverged during metazoan evolution, we identified and mapped on the tree (Fig 4, panel A) several R3 RPTP structural characteristics (Fig 4, panels B–E). Since tyrosine phosphorylation has been reported to occur at the amino acid sequence YxN in the carboxy terminus (after the PTP domain) in some mammalian R3 RPTP proteins (reviewed in Matozaki et al, 2010) [16], we mapped the predicted tyrosine phosphorylatable residues in this region on the tree (Fig 4, panel B and S1 File). Tyrosine phosphorylation in the YxN sequence was predicted in the vertebrate proteins PTPRJ, PTPRH, PTPRO and PTPRB and in the invertebrate proteins Ciona_1, Ciona_3, Acorn worm_1, Sea urchin_1, Annelid and Sponge (Fig 4, panel B). The PTPRB (except zebrafish), Ciona_3, Acorn worm_1 and Sponge sequences contain additional tyrosine phosphorylation in motifs different from YxN. The fruitfly proteins PTP10D, PTP4e and PTP52F had also predicted phophorylation sites different from YxN. The phosphorylation patterns were markedly conserved within the vertebrate clusters (Fig 4, panel B). No phosphorylation residues were predicted for PTPRQs and, interestingly, for the deuterostome proteins closer to the PTPRQ group: Ciona_2 (*C*. *intestinalis*), Ciona, (*C*. *savignyi*) and Acorn worm (Fig 4, panel B).

### Modification of the PTP catalytic peptide WPD in PTPRQ and related invertebrate proteins

The PTP domain of R1-R8 RPTPs comprises eight conserved peptide motifs (motif 8 is also called the WPD loop) and is involved in substrate recognition [6, 11]. PTPRQ is the only R3 RPTP member that dephosphorylates inositol phosphate rather than tyrosine amino acids. This change in substrate is due to the replacement of aspartic acid (D) by glutamic acid (E) in the WPD loop [11]. Our analysis showed that the aspartic acid in the WPD loop is evolutionary conserved in all vertebrate and invertebrate proteins analyzed with the exception of PTPRQ members, which have the expected glutamic residue (WPE). Modifications of the WPD sequence were also observed in the deuterostome sequences closer to the PTPRQ cluster, Ciona (WPQ), Ciona_2 (WSV) and Acorn worm (WPI), and in two echinoderm sequences, Sea urchin_3 (WVD) and Sea urchin_4 (WTD) (Fig 4, panel C).

### PTPRQ and PTPRB proteins characteristic and differential FN3 B-C loop lengths

FN3 domains are present in many proteins and are in general highly divergent with pair-wise sequence identities lower than 25% [35]. Despite this low sequence identity, FN3 tertiary structure is mostly conserved among domains, consisting of seven antiparallel β-strands (named A, B, C, D, E, F and G from the amino terminus) distributed between two β-sheets (E-B-A and G-F-C-D), allowing high variability in the loops interconnecting the β-strands [35, 36]. In addition, most FN3 domains contain two highly characteristic residues, a tryptophan (W) in β-strand B and a tyrosine (Y) in β-strand C, which delimitate the B-C loop [35]. Interestingly, we observed that between these two residues (W and Y) most PTPRQ FN3 domains have 12 amino acids whereas the majority of PTPRB FN3 domains have only nine amino acids (S2 Fig). We color-coded the distance between these two residues and a conserved glycine (G) inside the B-C loop, which has high conformational value, and plotted the results in the phylogenetic tree (Fig 4, panel D and S2 Fig). Results showed that PTPRB protein B-C loops were predominately of the red code (W4G4Y; where the numbers indicate the residues between W-G or G-Y conserved amino acids), while in PTPRQ proteins and invertebrate Ciona and Ciona_2 B-C loops were of the blue code (W7G4Y) interspersed with domains of the yellow code (W7G3Y) (Fig 4, panel D and S2 Fig). Curiously, the FN3 B-C loop codes in human and mouse PTPRHs seems species specific, while in other vertebrate R3 PTPRs B-C loop codes are conserved between different species (Fig 4, panel D and S2 Fig).

### FN3 domains exon/intron patterns and the evolution of R3 RPTPs

Protein domains including FN3 are generally bordered by introns that are of the same phase, which facilitates domain shuffling [37]. When we determined the intron phases of the FN3 sequences in R3 RPTPs, we observed that the majority of domains were contained within one exon flanked by two phase 1 introns (1:1; yellow boxes in Fig 4, panel E and S1 File). Other R3 RPTP FN3 domains have an additional intron inside the domain sequence in phase 0 (1:0:1; orange boxes in Fig 4, panel E), phase 1 (1:1:1; blue boxes in Fig 4, panel E and S1 File) or phase 2 (1:2:1; blackboxes in Fig 4, panel E and S1 File), and a juxtamembrane larger FN3 domain in deuterostomes has two introns inside the domain in phases 1 and 2 (1:1:2.1; greyboxes in Fig 4, panel E and S1 File). FN3 domains in invertebrate R3 RPTP sequences and PTPRBs, PTPRHs and PTPRJs were mostly of the yellow-coded boxes (1:1; Fig 4, panel E), while the FN3 domain pattern of PTPRQs was a mixture of the four color-coded boxes. A strict intron pattern with four domains of the orange-coded boxes (1:0:1, Fig 4 panel E) and a similar amino terminus sequence was observed in PTPROs (S1 File). In nematodes and fruitflies, but not in annelids, the FN3 intron patterns were different from the patterns in other R3 proteins, possibly because of the high incidence of intron gains in nematodes and intron losses in fruit flies, two well-known phenomena occurring specifically in these species [28, 38].

Since homologous introns are frequently located in the same position in the protein sequence [28], we determined the relative positions of the blue-, black- and orange-coded introns (as coded in Fig 4, panel E) in the FN3 domain sequences (S3 Fig). Between vertebrate and invertebrate sequences, only the introns in phase 2 (black-coded boxes, Fig 4 Panel E) were unambiguously located in the same position in PTPRQ and in the two Ciona sequences, *C intestinalis* Ciona_2, and *C*. *savignyi* Ciona which are close to the PTPRQ cluster additionally suggesting these ciona sequences and PTPRQ proteins share a common ancestor gene. The introns inside the domains were predominantly found (87%) in the region corresponding to FN3 β-strands C, D and E and in their interconnecting loops (S3 Fig). The high variability in sequence between vertebrate and invertebrate proteins in this region did not allow us to determine which introns were homologous between FN3 domains in other vertebrate and invertebrate sequences.

### Conserved synteny between vertebrate PTPRBs and tunicate Ciona_1 loci

**Fig 5:**
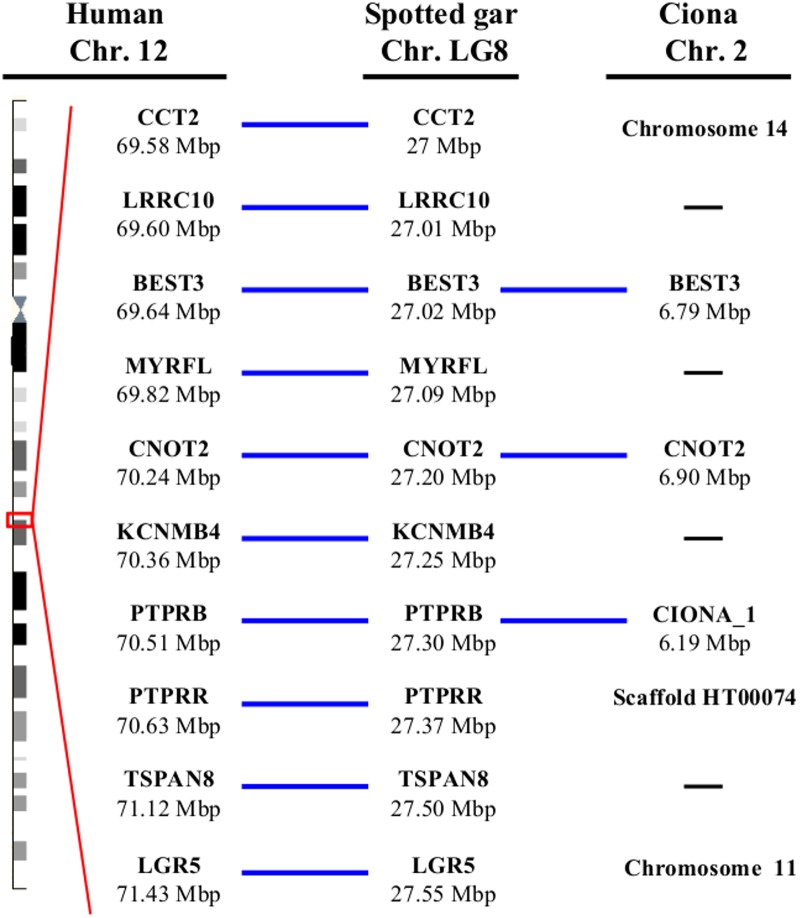
Synteny relationship between vertebrate PTPRBs and *C*. *intetinalis* Ciona_1 locus. Genes shown are those close to the PTPRB locus in human, the early diverged vertebrate spotted gar and around Ciona_1 locus (ENSCING00000003151) in the sea squirt (*C*. *intestinalis*). Blue lines indicate conserved genes and black lines indicate genes not present in the ciona genome. The genomic localization of the different genes is indicated below the gene name. The chromosome or scaffold number in the ciona genome for the genes present around PTPRB in vertebrates but not around the Ciona_1 gene in Chr.2 are also indicated. ENSEMBL *Ciona* genes are: CCT2 ENSCING00000005347; BEST3: ENSCING00000006827; CNOT2:ENSCING00000006804 or ENSCING00000021615; PTPRR ENSCING00000000420; LGR5 ENSCING00000018199 (see also S4 Fig).

An analysis of the conservation of synteny, the maintenace or co-localization of groups of genetic loci in the chromosomes of different species, showed that some synteny conservation still exists between the Ciona_1 locus in *C*. *intestinalis* and the PTPRB loci in vertebrates (Fig 5, S4 Fig), despite tunicates and vertebrates branching out over 500MYA [39]. Close to the Ciona_1 genetic locus in Chr. 2, we found the orthologs of BEST3 and CNTO2, genes that are located close upstream of PTPRB loci in vertebrates (Fig 5 and S4 Fig). Three other genes located close to PTPRB in vertebrates (LRCC10, MYRFL and KCNMB4) are absent in the *Ciona* genome.

## Discussion

This study provides new insights into how R3 RPTPs genes may have evolved and should facilitate our understanding of the structure and function of this group of integral membrane proteins with important roles in human disease.

### R3 RPTPs are an animal invention

Sponges were the most early diverged organisms in which we could retrieve a sequence with full R3 characteristics (sponge sequence). This observation points to the origin of R3 RPTPs in the common ancestor of animals. It is also in concordance with the fact that sponges, which are early diverged animals, are evolutionarly close to the expansion of the components of the cell phosphotyrosine signaling machinery tyrosine kinases, Src homology domains and PTP enzymes. This expansion took place just before the origin of metazoans (animals), which may have contributed to the appearance of animal multicellularity [40, 41].

### Fruitfly PTP52F constitutes a special case in the protostomes R3 RPTP monophyletic group

The divergent Fly_PTP52F sequence is a special case among protostome R3 sequences. It has been considered an unclassified member of the PTP superfamily [14, 21] and, in agreement, our analyses also show that the PTP52F PTP domain is not more closely related to R3 than to other RPTP subtypes. However, the conservation of R3 structural characteristics in PTP52F, such as the long FN3 juxtamembrane domain, includes PTP52F in the R3 RPTPs subfamily, in agreement with the reclassification proposed by Santhanam et al. 2013 [42]. Although PTP52F seems close to divergent Ciona_2 (*C*. *intestinalis*), Ciona (*C*. *savignyi*), acorn worm and PTPRQ proteins, PTP52 sequences can be found only in flies (*Muscomorpha*), suggesting that PTP52F is a fly invention and is no more related to PTPRQ than to other vertebrate proteins. This unrelatedness to PTPRQs is also suggested by the presence in PTP52F of a WPD loop sequence and a phophorylation motif in its cytosolic tail, two characteristics absent in PTPRQs and related invertebrate sequences. It is known that PTP4e, which is also found only in fruitflies, was generated from PTP10D, which is very similar to a recent duplication event [43]. Overall, our analyses agree with the existence of a single R3 RPTP invertebrate precursor gene that underwent an independent expansion in protostomes limited to flies, generating PTP52F and PTP4e as previously suggested [14].

Our analyses are in agreement with the lack of clear orthologous relationships with vertebrate proteins that have been reported for the well-studied fruit fly (PTP52F, PTP10D and PTP4e) and *C*. *elegans* (F44G4.8/DEP-1; Nematode_2 in our study) proteins [14].

### ProtoPTPRQ and protoPTPRB emergence in vertebrate deuterostomes

In invertebrate deuterostomes (echinoderms, hemichordates and urochordates), R3 RPTPs seem to be of two types: one type related to vertebrate PTPRO, PTPRB, PTPRJ and PTPRH and a second type with divergent PTP domains not closely related to R3 group members (Fig 2 and S1 Table). Interestingly, while all RPTPs of the first type have YxN phophorylation motifs, no tyrosine phophorylation sites were predicted in the sequences with the divergent PTP domain (Fig 4, panel B).

The synteny conservation between vertebrate PTPRB and tunicate Ciona_1 locus plus the results of the phylogenetic analysis and the conservation of FN3 introns patterns suggest Ciona_1 protein in *C*. *intestinalis* (Ciona_3 in *C*. *savignyi*) and vertebrate PTPRBs had a common ancestor. In the same regard, Ciona_2 (*C*. *intestinalis*) and Ciona, (*C*. *savignyi*) seem related to PTPRQs as they have mutated WPD loops, similar FN3-like intron/exon patterns, B-C loop motifs and the absence of phosphorylation signals.

In more early diverged deuterostomes, in previous studies based on sequence similarity, Sea urchin_3 and Sea urchin_4 have been considered divergent R3 RPTP proteins with no vertebrate orthologs [24], in agreement with our results that place these sequences in a cluster of their own. Nevertheless, the FN3 B-C loop structure in the Sea urchin_1 sequence still maintains similarities to that of vertebrate PTPRBs (Fig 4). In fact, we cannot rule out the possibility that a duplication of this ancestral gene occurred before the split of protostomes and deuterostomes followed by a subsequent loss of one copy in protostomes.

### R3 RPTPs diversification and structure function implications

All vertebrate R3 RPTPs clustered into the five known groups, PTPRQ, PTPRO, PTPRB, PTPRJ and PTPRH, in agreement with previous analyses with only vertebrate sequences [6, 9].

Our phylogenetic analyses indicate that PTPRO, PTPRB, PTPRJ and PTPRH are homologous (PTPRJ and PTPRH groups appeared as each other´s sisters), a result also supported by the similarity in their intron and posphorylation patterns (Fig 4).

These related proteins in our analyses undergo tyrosine phosphorylation in their carboxy-terminal region, which promotes the binding of Src family kinases [16]. It has been proposed that they dephosphorylate tyrosine kinases (RTK) to regulate RTK signaling since most of the known substrates of R3 RPTPs are RTKs [14, 16]. An involvement of these proteins and R3 RPTPs from invertebrate model animals, except PTP52F, in tubular organ development, has been suggested by Jeon and Zinn, 2015 [14]. Such is the involvement of PTPRB in blood vessel development in vertebrates and PTP4e and PTP10d in the development of the tracheal (respiratory) system in *Drosophila*, which has many similarities to the mammalian vascular system [14, 16]. Additionally, PTPRO has clear functions during neural development, similar to *Drosophila* R3 RPTPs including PTP52F. Our analysis indicates that vertebrate and protostome proteins had a common ancestor and although the sequences in protostomes are divergent, as indicated by Jeon and Zinn, 2015 [14], they probably still share common mechanisms of action with the vertebrate proteins, although the posphorylation sequence YxN is only conserved in the annelid sequence.

R3 proteins from *Drosophila* have common features with vertebrate R3 RPTPs, excluding PTPRQ, and have in fact been considered functional orthologs of PTPRH and PTPRJ [42, 44].

On the other hand, some tyrosine phosphatase family members are catalytically inactive or dephosphorylate complex carbohydrates, mRNA or phosphoinositides [12, 24] instead of tyrosine residues. This change in substrate in PTPRQs that dephosphorylate phosphatidyl inositol phosphate is mediated by the replacement of the aspartate in the WPD loop by a glutamate [11]. In vertebrates, only PTPRQs have this characteristic, and in contrast to other vertebrates RPTPs, PTPRQs lack tyrosine phosphorylation motifs. Additionally, PTPRQs have a unique combination of intron codes and B_C loop motifs, further suggesting their divergence from the PTPRO, PTPRB, PTPRJ and PTPRH cluster (Fig 4).

The common ancestry of vertebrate groups of R3 RPTP proteins, between PTP domain similarity and structural characteristics unrelated to the PTP domain in the different R3 RPTP groups, agrees with the reported classification of all RPTP receptors in eight almost identical subtypes (R1-R8) by considering either the different domain composition or catalytic PTP domain amino acid similarity [6]. These relationships also suggest co-evolution of the catalytic and interaction domains of R3 RPTP in vertebrates, presumably because of a requirement to ensure appropriate substrate recognition and/or cellular localization, as is suggested for protein kinases [45].

Our study indicates the existence of an R3 RPTP common ancestor before the divergence of protostomes and deuterostomes. A duplication event in non-vertebrate deuterostomes generated the protoPTPRB and PTPRQ genes and a second wave of duplications of the protoPTPRB in vertebrates generated the PTPRB, PTPRH, PTPRJ and PTPRO groups. This diversification patterns agrees with the differential structural characteristics of these proteins. These results will be useful to further understand the structure and function of these proteins relevant to human health and disease.

## Supporting information

S1 Fig Phylogenetic trees of R3 RPTPs DNA and protein sequences generated using parsimony analysis and Bayesian analysis. For these trees, we varied the input matrix so that the protein and DNA sequences were analyzed simultaneously [46, 47] as elided matrices. We also include the bootstrap results when Maximum Parsimony is used as the optimality criterion. The specifics of the analysis are given at the top of the tree in the figure legends preceding the trees.
(PDF)

S2 Fig Alignment of FN3 B-C loop regions of all R3 RPTPs. Alignment of B-C loop region of all FN3 domains. Domains are numbered from the one closest to the transmembrane as in S1 File. Only the human proteins are shown within the vertebrate clade. Fibronectin FN3 10th repeat is depicted in the top of the alignments and the B-C β-strands are highlighted in magenta. Tryptophan (W) in strand B and tyrosine (Y) in strand C are highlighted in black. The conserved glycines inside the B-C loop are shaded in blue.
(PDF)

S3 Fig Alignment of FN3 domains contained in more than one exon and with an additional intron inside the domain sequence. Alignment of the FN3 domains from Fig 4, panel E with introns inside the FN3 domain and represented by orange, blue or black squares. Fibronectin FN3 10th repeat is depicted in the top of the alignment and the A-F β-strands are highlighted in magenta. Highly conserved FN3 residues (W, Y, L, Y) are in blue bold letter. Introns in phase 2 are represented by red bold letters and highlighted in yellow; introns in phase 1 are represented by red bold letters and highlighted in grey; introns in phase 0 are indicated by highlighting the flanking amino acids in green. Chicken (Gg) and *Xenopus* (Xt) FN3 domains were omitted whentheir intron positions were identical to that of human (Hs) and zebrafish (Dr). FN3 domains are numbered starting from the one closest to the transmembrane domain as in Fig 4, panel D.
(PDF)

S4 Fig Synteny relationship between vertebrate PTPRBs and *C*. *intetinalis* ciona.1 locus. Caption of the ENSEMBL genomic region of PTPRB in human and spotted gar and of Ciona_1 in the sea squirt *C*. *intetinalis* genome.
(TIF)

S1 File List of all R3 RPTP sequences used in this study and their accession numbers. Transmembrane segments are highlighted in pink, and PTP catalytic domains are underlined. Exons are displayed in alternate colors. Amino acids in bold red colour indicate that they are split between adjacent exons by a phase 1 or 2 intron. FN3 domains are shaded in the same colors as in Fig 3 and are numbered in parenthesis. The cysteine residues of the long juxtamembrane FN3 domain are highlighted in yellow. Tyrosine phosphorylation predicted residues in the carboxy terminus are highlighted in red and the asparagines of the YxN motif in blue.
(PDF)

S2 File Elided matrix of the protein sequences partitioned according to the gap costs = 1, 2, 4, 6, 8, and 16. Each individual matrix appears in increasing gap cost order from top to bottom as labelled.
(PDF)

S3 File Matrix of the DNA sequences aligned using Translator X. (PDF)

S1 Table Vertebrate and invertebrate R3 RPTPs PTP domain similarities. The NCBI PBLAST E values were plotted in the table, E values lower than e-80 are shaded.
(PDF)

S2 Table PTP52F and PTP10D in protostomes. Organism reporte and NCBI PBLAST E values obtained after blasting the PTP domains of PTP52F (A) and PTP10D (B) in Protostomia (taxid:33317).
(PDF)
