# Supplementary material for: Phosphotyrosine phosphatase R3 receptors: Origin, evolution and structural diversification
Source: PLoS One. 2017 Mar 3;12(3):e0172887. doi: 10.1371/journal.pone.0172887 (PMC5336234; doi:10.1371/journal.pone.0172887)
Supplement: S1 Fig — For these trees, we varied the input matrix so that the protein and DNA sequences were analyzed simultaneously [46, 47] as elided matrices. We also include the bootstrap results when Maximum Parsimony is used as the optimality criterion. The specifics of the analysis are given at the top of the tree in the figure legends preceding the trees. (PDF) [file pone.0172887.s001.pdf]

Supplemental Figure S1. Description of and diagrams of phylogenetic trees generated in this study.

Figure S1.1.; This tree was generated using the elided protein + DNA data matrix + structural characters with alignments set at gap cost = 6. Maximum parsimony was used with 100 random addition replicates and TBR branch swapping. A single tree resulted from the search.

Figure S1.2 .; This tree was generated using the elided protein + DNA data matrix with alignments set at gap cost = 6. Maximum parsimony was used with 100 random addition replicates and TBR branch swapping for each bootstrap replicate. 1000 bootstrap replicates were accomplished to generate the bootstrap values shown on the nodes.

Figure S1.3. This tree was generated using the elided protein data matrix with alignments set at gap cost = 6. Maximum parsimony was used with 100 random addition replicates and TBR branch swapping.

Figure S1.4. This tree was generated using the elided protein data matrix with alignments set at gap cost = 6. Maximum parsimony was used with 100 random addition replicates and TBR branch swapping. For each bootstrap replicate. 1000 bootstrap replicates were accomplished to generate the bootstrap values shown on the nodes.

Figure S1.5. This tree was generated using the elided DNA data matrix with alignments set at gap cost = 1, 2, 4, 6, 8, and 16. Maximum parsimony was used with 100 random addition replicates and TBR branch swapping. A single tree resulted from the search.

Figure S1.6. This tree was generated using the elided DNA data matrix with alignments set at gap cost = 1, 2, 4, 6, 8, and 16. Maximum parsimony was used with 100 random addition replicates and TBR branch swapping., for each bootstrap replicate. 1000 bootstrap replicates were accomplished to generate the bootstrap values shown on the nodes.

Figure S1.7 . Figure S1.3. This tree was generated using the DNA data matrix with alignment set at gap cost = 6. Maximum parsimony was used with 100 random addition replicates and TBR branch swapping.

Figure S1.8 . Figure S1.3. This tree was generated using the DNA data matrix with alignment set at gap cost = 6. Maximum parsimony was used with 100 random addition replicates and TBR branch swapping, for each bootstrap replicate. 1000 bootstrap replicates were accomplished to generate the bootstrap values shown on the nodes.

Figure S1.9. This tree was generated using the protein data matrix with alignment set at gap cost = 6. Bayes phylogenetic inference was used to generate the tree. Bayesian analysis of protein sequences used the WAG model, with gamma distribution and invariants for 1,000,000 generations.

Figure S1.10. This tree was generated using the elided protein data matrix with alignment set at gap cost = 1, 2, 4, 6, 8, and 16. Bayes phylogenetic inference was used to generate the tree. Bayesian analysis of protein sequences used the WAG model, with gamma distribution and invariants for 1,000,000 generations.

Figure S1.11. This tree was generated using the DNA data matrix with alignment set at gap cost = 6. Bayes phylogenetic inference was used to generate the tree. Bayesian analysis of protein sequences used the GTR model, with gamma distribution and invariants for 1,000,000 generations.

Figure S1.12. This tree was generated using the elided DNA data matrix with alignment set at gap cost = 1, 2, 4, 6, 8, and 16. Bayes phylogenetic inference was used to generate the tree. Bayesian analysis of protein sequences used the GTR model, with gamma distribution and invariants for 1,000,000 generations.

Figure S1.13. This tree was generated using the DNA data matrix with alignment set at gap cost = 6. Likelihood analysis was applied using a bootstrap for 1000 replicates. The GTR model, with gamma distribution and invariants was used.

Figure S1.14. This tree was generated using the protein data matrix with alignment set at gap cost = 6. Likelihood analysis was applied using a bootstrap for 1000 replicates. The WAG model, with gamma distribution and invariants was used.

Figure S1.15. This tree was generated using the elided DNA data matrix with alignment set at gap cost = 1, 2, 4, 6, 8, and 16. Likelihood analysis was applied using a bootstrap for 1000 replicates. The GTR model, with gamma distribution and invariants was used.

Figure S1.16. This tree was generated using the elidedprotein data matrix with alignment set at gap cost = 1, 2, 4, 6, 8, and 16. Likelihood analysis was applied using a bootstrap for 1000 replicates. The WAG model, with gamma distribution and invariants was used.

Figure S1.1

```
/---------------------------------------------------------------- PTPRG Hs
|
+---------------------------------------------------------------- PTPRF Hs
|
|                                  /------------------------ Spongue
|                                  |
|                                  |                 /----- PTPRO human
|                                  |             /---41
|                                  |             |    \----- PTPRO mouse
|                              /---45        /---42
|                              |    |         |    \---------- PTPRO chiken
|                              |    |    /---43
|                              |    |    |    \--------------- PTPRO frog
|                              |    \---44
|                              |         \-------------------- PTPRO fish
|                              |
|                          /---50                      /----- Nematode 2
|                          |    |                 /---46
|                          |    |                 |    \----- nematode 3
|                          |    |             /---48
|                          |    |             |    |    /----- FlyDPTP4e
|                          |    |             |    \---47
|                          |    \-------------49        \----- Fly PTP10D
|                          |                 |
|                          |                 \--------------- annelid
|                          |
|                      /---57                      /---------- PTPRB human
|                      |    |                      |
|                      |    |                 /---52    /----- PTPRB mouse
|                      |    |                 |    \---51
|                      |    |             /---53        \----- PTPRBgallus
|                      |    |             |    |
|                      |    |         /---54    \--------------- PTPRB frog
|                  /---58    |         |    |
78                 |    |    |         |    \-------------------- PTPRB fish
|                  |    |    \--------56
|                  |    |             |                 /----- ciona 1
|                  |    |             \------------------55
|              /---59    |                              \----- ciona 3
|              |    |    |
|              |    |    \--------------------------------------- SeaUrchin 1
|              |    |
|              |    \-------------------------------------------- acornWorm 1
|              |
|              |                                  /----- PTPRJ human
|              |                              /---60
|          /---67                              |    \----- PTPRJmouse
|          |    |                         /---61
|          |    |                         |    \---------- PTPRJ chiken
|          |    |                     /---62
|          |    |                     |    \--------------- PTPRJ fish
|          |    |                     |
|          |    |                     |            /----- PTPRH human
|          |    \---------------------------66        /---63
|          |                          |        |    \----- PTPRH mouse
|          |                          |    /---64
|      /---75                          |    |    \---------- PTPRH fish
|      |    |                          \---65
|      |    |                              \--------------- PTPRH frog
|      |    |
|      |    |                                  /---------- Fly Ptp52F
|      |    |                                  |
|      |    |                      /-------------69    /----- ciona
|      |    |                      |             \---68
|      |    |                      |                 \----- ciona 2
|      |    |                      |
|      |    \----------------------------74    /-------------------- acornWorm
\---77                              |    |
    |                          |    |        /---------- PTPRQhuman
    |                          \---73        |
    |                              |    /---71    /----- PTPRQ mouse
    |                              |    |    \---70
    |                              \---72        \----- PTPRQ chiken
    |                                  |
    |                                  \--------------- PTPRQ fish
    |
    |                                             /----- SeaUrchin 3
    \----------------------------------------------------76
                                                  \----- SeaUrchin 4
```

Figure S1.2.

```
/-------------------------------------------------------------- PTPRG Hs(1)
|
+-------------------------------------------------------------- PTPRF Hs(2)
|
|      /----------------------------------------------------- Spongue(3)
|      |
|      |                                  /-------- Nematode 2(4)
|      |                           /-100--+
|      |                           |      \-------- nematode 3(38)
|      |                     /--86---+
|      |                     |       |      /-------- FlyDPTP4e(6)
|      |                     |       \-100--+
|      |     /----------82----------+            \-------- Fly PTP10D(7)
|      |     |                     |
|      |     |                     \----------------------- annelid(5)
|      |     |
|      |     |                           /--------------- Fly Ptp52F(8)
|      |     |                           |
|      |     +--------------76--------------+     /-------- ciona(13)
|      |     |                           \-100--+
|      |     |                                  \-------- ciona 2(39)
|      |     |
|      |     +---------------------------------------------- acornWorm(9)
|      |     |
|      |     +---------------------------------------------- SeaUrchin 1(10)
\--100--+     |
       |     |                                  /-------- SeaUrchin 3(11)
       |     +-----------------100-----------------+
       |     |                                  \-------- SeaUrchin 4(12)
       |     |
       |     |                                  /-------- PTPRQhuman(14)
       |     |                           /--66--+
       |     |                           |      \-------- PTPRQ mouse(15)
       |     |                     /--100--+
       |     |                     |      \--------------- PTPRQ chiken(16)
       |     +---------100----------+
       |     |                     \----------------------- PTPRQ fish(17)
       |     |
       |     |                           /--------------- PTPRB human(18)
       |     |                           |
       |     |                     /--100--+     /-------- PTPRB mouse(19)
       |     |                     |      \--75--+
       |     |              /--100--+            \-------- PTPRBgallus(20)
       |     |              |      |
       |     |      /-100--+      \----------------------- PTPRB frog(21)
       \--54--+      |      |
             |      |      \------------------------------- PTPRB fish(22)
             +--86---+
             |      |                           /-------- ciona 1(36)
             |      \-------------100-------------+
             |                                  \-------- ciona 3(40)
             |
             |                                  /-------- PTPRJ human(23)
             |                           /-100--+
             |                           |      \-------- PTPRJmouse(24)
             |                     /--100--+
             |                     |      \--------------- PTPRJ chiken(25)
             |              /--100--+
             |              |      \----------------------- PTPRJ fish(26)
             |              |
             |              |                   /-------- PTPRH human(32)
             +------98------+            /-100--+
             |              |            |      \-------- PTPRH mouse(33)
             |              |      /--70---+
             |              |      |      \--------------- PTPRH fish(35)
             |              \--94---+
             |                     \----------------------- PTPRH frog(34)
             |
             |                                  /-------- PTPRO human(27)
             |                           /--84--+
             |                           |      \-------- PTPRO mouse(28)
             |                     /--100--+
             |                     |      \--------------- PTPRO chiken(29)
             |              /--100--+
             |              |      \----------------------- PTPRO frog(30)
             +-----100------+
             |              \------------------------------- PTPRO fish(31)
             |
             \---------------------------------------------- acornWorm 1(37)
```

Figure s1.3

```
/---------------------------------------------------------------- PTPRG Hs
|
+---------------------------------------------------------------- PTPRF Hs
|
|         /------------------------------------------------------ Sponge
|         |
|         |                                    /------ nemato 2
|         |                                /-----+
|         |                                |     \------ nemato 3
|         |                           /-----+
|         |                           |     |     /------ Fly DPTP4e
|         |                           |     \-----+
|         |                      /-----+          \------ Fly PTP10D
|         |                      |     |
|         |                 /-----+    \----------------- annelid
|         |                 |     |
|         |            /------+   \----------------------- SeaUr 1
|         |            |      |
|         |            |      \----------------------------- AcoWor 1
|         |       /-----+
|         |       |     |                        /------ ciona 1
|         |       |     \------------------------------+
|         |       |                              \------ ciona 3
|    /-----+      |
|    |    |       |                              /------ PTPRB hum
|    |    |       |                          /-----+
|    |    |       |                          |     \------ PTPRB mou
|    |    |    /-----+                   /-----+
|    |    |    |     |                   |     \----------- PTPRB chi
|    |    |    |     |               /-----+
|    |    |    |     |               |     \----------------- PTPRB fro
|    |    |    |     |           /-----+
|    |    |    |     |           |     \----------------------- PTPRB fis
|    |    |    |     |           |
|    |    |    |     |           |                /------ PTPRJ hum
|    |    |    |     |           |            /-----+
|    |    |    |     |           |            |     \------ PTPRJ mou
|    |    |    |     \-----------+        /-----+
|    |    |    |                 |        |     \----------- PTPRJ chi
|    |    |    |                 |    /-----+
|    |    |    |                 |    |     \----------------- PTPRJ fis
|    |    |    |                 |    |
|    |    \-----+                |    |             /------ PTPRH hum
|    |          |                \-----+        /-----+
|    |          |                     |         |     \------ PTPRH mou
|    |          |                     |    /-----+
|    |          |                     |    |     \----------- PTPRH fis
|    |          |                     \-----+
\-----+         |                           \----------------- PTPRH fro
     |         |
     |         |                               /------ PTPRO hum
     |         |                               |
     |         |                           /-----+------ PTPRO mou
     |         |                           |     |
     |         |                      /-----+    \------ PTPRO chi
     |         |                      |     |
     |         \------------------------------+    \----------- PTPRO fro
     |                                |
     |                                \----------------- PTPRO fis
     |
     |                                    /----------- Fly Ptp52F
     |                                    |
     |                               /-----+    /------ ciona
     |                               |     \-----+
     |                               |           \------ ciona 2
     |                          /-----+
     |                          |     |         /------ SeaUr 3
     |                          |     \-----------+
     |                     /-----+                \------ SeaUr 4
     |                     |    |
     |                     |    \----------------------- acornWorm
     |                     |
     |                     |                   /------ PTPRQ hum
     \------------------------------+              /-----+
                           |              |    \------ PTPRQ mou
                           |         /-----+
                           |         |    \----------- PTPRQ chi
                           \-----------+
                                     \----------------- PTPRQ fis
```

Figure S1.4

```
/--------------------------------------------------------------- PTPRG Hs(1)
|
+--------------------------------------------------------------- PTPRF Hs(2)
|
|      /------------------------------------------------------ Sponge(3)
|      |
|      |                                /--------- nemato 2(4)
|      |                         /--100---+
|      |                         |        \--------- nemato 3(38)
|      +----------------72-----------------+
|      |                         |        /--------- Fly DPTP4e(6)
|      |                         \--100---+
|      |                                \--------- Fly PTP10D(7)
|      |
|      +------------------------------------------------------ annelid(5)
|      |
|      +------------------------------------------------------ Fly Ptp52F(8)
|      |
|      +------------------------------------------------------ acornWorm(9)
|      |
|      +------------------------------------------------------ SeaUr 1(10)
|      |
|      |                                /--------- SeaUr 3(11)
|      +---------------------78---------------------+
|      |                                \--------- SeaUr 4(12)
|      |
|      |                                /--------- ciona(13)
|      +--------------------100---------------------+
|      |                                \--------- ciona 2(39)
|      |
|      |                                /--------- PTPRQ hum(14)
|      |                         /---53---+
|      |                         |        \--------- PTPRQ mou(15)
|      |                  /---98---+
|      |                  |      \------------------ PTPRQ chi(16)
|      +-----------100------------+
|      |                  \--------------------------- PTPRQ fis(17)
|      |
|      |                                /--------- PTPRB hum(18)
|      |                         /---54---+
|      |                         |        \--------- PTPRB mou(19)
\---88---+                 /---84---+
       |                 |      \------------------ PTPRB chi(20)
       |          /---95---+
       |          |      \--------------------------- PTPRB fro(21)
       |     /--100---+
       |     |      \------------------------------------ PTPRB fis(22)
       +---61---+
       |     |                          /--------- ciona 1(36)
       |     \----------------100----------------+
       |                                \--------- ciona 3(40)
       |
       |                                /--------- PTPRJ hum(23)
       |                         /---99---+
       |                         |      \--------- PTPRJ mou(24)
       |                  /---99---+
       |                  |      \------------------ PTPRJ chi(25)
       |          /--100---+
       |          |      \--------------------------- PTPRJ fis(26)
       |          |
       |          |                     /--------- PTPRH hum(32)
       +-------76--------+         /--100---+
       |          |         |      \--------- PTPRH mou(33)
       |          |         |
       |          \-------64--------+------------------ PTPRH fro(34)
       |                    |
       |                    \------------------ PTPRH fis(35)
       |
       |                                /--------- PTPRO hum(27)
       |                         /---68---+
       |                         |      \--------- PTPRO mou(28)
       |                  /---99---+
       |                  |      \------------------ PTPRO chi(29)
       |          /---96---+
       |          |      \--------------------------- PTPRO fro(30)
       +-------98--------+
       |          \------------------------------------ PTPRO fis(31)
       |
       \------------------------------------------------------ AcoWor 1(37)
```

Figure S1.5

```
/------------------------------------------------------------ PTPRF Hs
|
+------------------------------------------------------------ PTPRG Hs
|
|            /------------------------------------------- Spongue
|            |
|            |                             /------ nemat 2
|            |                        /----41
|            |                        |    \------ nematode 3
|            |                   /----43
|            |                   |    |    /------ FlyDPTP4e
|            |                   |    \----42
|            |           /----------44     \------ Fly PTP10D
|            |           |        |
|            |           |        \------------------ annelid
|            |           |
|            |           |                 /------ PTPRO hum
|            |           |            /----45
|            |       /----49          |    \------ PTPRO mou
|            |       |   |       /----46
|            |       |   |       |    \----------- PTPRO chi
|            |       |   |  /----47
|       /----69      |   |  |    \------------------ PTPRO fro
|       |    |       |   \----48
|       |    |       |        \----------------------- PTPRO fis
|       |    |       |
|       |    |       |                     /------ PTPRB hum
|       |    |   /----56              /----50
|       |    |   |    |               |    \------ PTPRB mou
|       |    |   |    |          /----51
|       |    |   |    |          |    \----------- PTPRBg chi
|       |    |   |    |     /----52
78      |    |   |    |     |    \------------------ PTPRB fro
|       |    |   |    |  /----53
|       |    |   |    |  |    \----------------------- PTPRB fis
|       |    |   |    \----55
|       |    |   |         |                /------ ciona 1
|       |    |   |         \----------------------54
|       |    |   |                          \------ ciona 3
|       |    \----68
|       |        |                      /----------- SeaUr 1
|       |        |                      |
|       |        |    /----------------------58    /------ SeaUr 3
|       |        |    |                     \----57
|       |        |    |                          \------ SeaUr 4
|       |        |    |
|       |        |    |                     /------ PTPRJ hum
|   /----76      |    |                /----59
|   |   |        |    |                |    \------ PTPRJmou
|   |   |        |    |           /----60
|   |   |        |    |           |    \----------- PTPRJ chi
|   |   |        \----67     /----61
|   |   |             |      |    \------------------ PTPRJ fis
|   |   |             |      |
|   |   |             |      |              /------ PTPRH hum
|   |   |             |  /----65       /----62
|   |   |             |  |   |         |    \------ PTPRH mou
|   |   |             |  |   |    /----63
|   |   |             |  |   |    |    \----------- PTPRH fro
|   |   |             \----66    \----64
|   |   |                 |         \------------------ PTPRH fis
|   |   |                 |
|   |   |                 \---------------------------- acornWorm 1
\----77 |
    |   |                             /----------- acornWorm
    |   |                             |
    |   |                    /----------71    /------ ciona
    |   |                    |        \----70
    |   |                    |            \------ ciona 2
    |   |                    |
    |   |                    |            /------ PTPRQhum
    |   \-------------------------75      /----72
    |                        |       |    \------ PTPRQ mou
    |                        |  /----73
    |                        |  |    \----------- PTPRQ chi
    |                        \----74
    |                            \------------------ PTPRQ fis
    |
    \---------------------------------------------------------- Fly Ptp52F
```

Figure S1.6

```
/---------------------------------------------------------------- PTPRF Hs(1)
|
+---------------------------------------------------------------- PTPRG Hs(2)
|
|      /------------------------------------------------------- Spongue(3)
|      |
|      |                                  /-------- nemat 2(4)
|      |                           /--100--+
|      |                           |       \-------- nematode 3(38)
|      |                           |
|      |     /--------------89--------------+---------------- annelid(5)
|      |     |                              |
|      |     |                              |       /-------- FlyDPTP4e(6)
|      |     |                              \--100--+
|      |     |                                      \-------- Fly PTP10D(7)
|      |     |
|      |     +----------------------------------------------- Fly Ptp52F(8)
|      |     |
|      |     +----------------------------------------------- acornWorm(9)
|      |     |
|      |     +----------------------------------------------- SeaUr 1(10)
|      |     |
|      |     |                                  /-------- SeaUr 3(11)
|      |     +------------------85------------------+
\--93---+     |                                  \-------- SeaUr 4(12)
       |     |
       |     |                                  /-------- ciona(13)
       |     +-----------------100------------------+
       |     |                                  \-------- ciona 2(39)
       |     |
       |     |                                  /-------- PTPRQhum(14)
       |     |                           /--98---+
       |     |                           |       \-------- PTPRQ mou(15)
       |     |                    /-100--+
       |     |                    |      \---------------- PTPRQ chi(16)
       |     +----------100----------+
       |     |                    \----------------------- PTPRQ fis(17)
       |     |
       |     |                                  /-------- PTPRB hum(18)
       |     |                           /--100--+
       |     |                           |       \-------- PTPRB mou(19)
       |     |                    /--98--+
       |     |                    |      \---------------- PTPRBg chi(20)
       |     |             /--100--+
       \--58--+             |      \----------------------- PTPRB fro(21)
             |      /--100--+
             |      |      \------------------------------- PTPRB fis(22)
             +--78---+
             |      |                           /-------- ciona 1(36)
             |      \-------------100--------------+
             |                                  \-------- ciona 3(40)
             |
             |                                  /-------- PTPRJ hum(23)
             |                           /--100--+
             |                           |       \-------- PTPRJmou(24)
             |                    /-100--+
             |                    |      \---------------- PTPRJ chi(25)
             |             /--100--+
             |             |      \----------------------- PTPRJ fis(26)
             |             |
             |             |                    /-------- PTPRH hum(32)
             +------79-------+             /--100--+
             |             |             |       \-------- PTPRH mou(33)
             |             |      /--59--+
             |             |      |      \---------------- PTPRH fro(34)
             |             \--62---+
             |                    \----------------------- PTPRH fis(35)
             |
             |                                  /-------- PTPRO hum(27)
             |                           /--89---+
             |                           |       \-------- PTPRO mou(28)
             |                    /-100--+
             |                    |      \---------------- PTPRO chi(29)
             |             /--96---+
             |             |      \----------------------- PTPRO fro(30)
             +------100------+
             |             \------------------------------- PTPRO fis(31)
             |
             \----------------------------------------------- acornWorm 1(37)
```

Figure S1.7

```
/------------------------------------------------------------------ PTPRF Hs
|
+------------------------------------------------------------------ PTPRG Hs
|
|   /------------------------------------------------------------- Spongue
|   |
|   |                                             /----- nemat 2
|   |                                         /---41
|   |                                         |   \----- nematode 3
|   |                                     /---42
|   |                                     |   \---------- annelid
|   |               /------------------------44
|   |               |                     |       /----- FlyDPTP4e
|   |               |                     \--------43
|   |               |                             \----- Fly PTP10D
|   |               |
|   |               |                 /-------------------- Fly Ptp52F
|   |               |                 |
78  |               |                 |           /----- PTPRQhum
|   |               |                 |       /---45
|   |               |   /-------------48      |   \----- PTPRQ mou
|   |               |   |             |   /---46
|   |           /---63  |             |   |   \---------- PTPRQ chi
|   |           |   |   |             \---47
|   |           |   |   |                 \--------------- PTPRQ fis
|   |           |   |   |
|   |           |   |   |                 /--------------- acornWorm
|   |           |   |   |                 |
|   |           |   |   |                 |       /----- SeaUr 3
|   |           |   |   |             /---52  /---49
|   |           |   |   |             |   |   |   \----- SeaUr 4
|   |           |   |   |             |   \---51
|   |           |   |   |             |       |   /----- ciona
|   |           |   \---62        /---53      \---50
|   |           |       |         |   |           \----- ciona 2
|   |           |       |         |   |
\---77          |       |         |   \-------------------- acornWorm 1
    |           |       |         |
    |           |       |         |               /----- PTPRJ hum
    |           |       |    /---58           /---54
    |           |       |    |    |           |   \----- PTPRJmou
    |           |       |    |    |       /---55
    |       /---68      |    |    |       |   \---------- PTPRJ chi
    |       |   |       |    |    |   /---56
    |       |   |       |    |    |   |   \--------------- PTPRJ fis
    |       |   |       \----61   \---57
    |       |   |            |        \-------------------- PTPRH fis
    |       |   |            |
    |       |   |            |                    /----- PTPRH hum
    |       |   |            |                /---59
    |       |   |            |                |   \----- PTPRH mou
    |       |   |            \-----------------60
    |       |   |                             \---------- PTPRH fro
    |       |   |
    |       |   |                                 /----- PTPRO hum
    |       |   |                             /---64
    |       |   |                             |   \----- PTPRO mou
    |       |   |                         /---65
    |   /---75  |                         |   \---------- PTPRO chi
    |   |   |   |                     /---66
    |   |   |   |                     |   \--------------- PTPRO fro
    |   |   |   \-------------------------67
    |   |   |                         \-------------------- PTPRO fis
    |   |   |
    |   |   |                                 /----- PTPRB hum
    |   |   |                             /---69
    |   |   |                             |   \----- PTPRB mou
    |   |   |                         /---70
    \---76  |                         |   \---------- PTPRBg chi
        |   |                     /---71
        |   |                     |   \--------------- PTPRB fro
        |   |                 /---72
        |   |                 |   \-------------------- PTPRB fis
        |   \------------------------74
        |                     |               /----- ciona 1
        |                     \-----------------73
        |                                     \----- ciona 3
        |
        \-------------------------------------------------------- SeaUr 1
```

Figure S1.8

```
/-------------------------------------------------------------- PTPRF Hs(1)
|
+-------------------------------------------------------------- PTPRG Hs(2)
|
|       /----------------------------------------------------- Spongue(3)
|       |
|       |                               /--------- nemat 2(4)
|       |                       /---99---+
|       |                       |       \--------- nematode 3(38)
|       |                       |
|       +-----------------54----------------+------------------ annelid(5)
|       |                       |
|       |                       |       /--------- FlyDPTP4e(6)
|       |                       \--100---+
|       |                               \--------- Fly PTP10D(7)
|       |
|       +----------------------------------------------------- Fly Ptp52F(8)
|       |
|       +----------------------------------------------------- acornWorm(9)
|       |
|       +----------------------------------------------------- SeaUr 1(10)
|       |
|       |                               /--------- SeaUr 3(11)
|       +--------------------70---------------------+
|       |                               \--------- SeaUr 4(12)
|       |
|       |                               /--------- ciona(13)
|       +--------------------100--------------------+
|       |                               \--------- ciona 2(39)
|       |
|       |                               /--------- PTPRQhum(14)
|       |                       /---97---+
|       |                       |       \--------- PTPRQ mou(15)
|       |               /---99---+
|       |               |       \------------------ PTPRQ chi(16)
|       +-----------100-----------+
|       |               \--------------------------- PTPRQ fis(17)
|       |
|       |                               /--------- PTPRB hum(18)
|       |                       /--100---+
|       |                       |       \--------- PTPRB mou(19)
\---86---+               /---88---+
        |               |       \------------------ PTPRBg chi(20)
        |       /--97---+
        |       |       \--------------------------- PTPRB fro(21)
        |   /---98---+
        |   |       \----------------------------------- PTPRB fis(22)
        +---65---+
        |   |                           /--------- ciona 1(36)
        |   \---------------100----------------+
        |                               \--------- ciona 3(40)
        |
        |                               /--------- PTPRJ hum(23)
        |                       /--100---+
        |                       |       \--------- PTPRJmou(24)
        |               /---99---+
        |               |       \------------------ PTPRJ chi(25)
        |       /--88---+
        |       |       \--------------------------- PTPRJ fis(26)
        |       |
        |       |                       /--------- PTPRH hum(32)
        |       |               /--100---+
        +-------54--------+             |       \--------- PTPRH mou(33)
        |       +-------57-------+
        |       |               \------------------ PTPRH fro(34)
        |       |
        |       \----------------------------------- PTPRH fis(35)
        |
        |                               /--------- PTPRO hum(27)
        |                       /---94---+
        |                       |       \--------- PTPRO mou(28)
        |               /---98---+
        |               |       \------------------ PTPRO chi(29)
        |       /--94---+
        |       |       \--------------------------- PTPRO fro(30)
        +-------97--------+
        |       \----------------------------------- PTPRO fis(31)
        |
        \----------------------------------------------------- acornWorm 1(37)
```

Figure S1.9

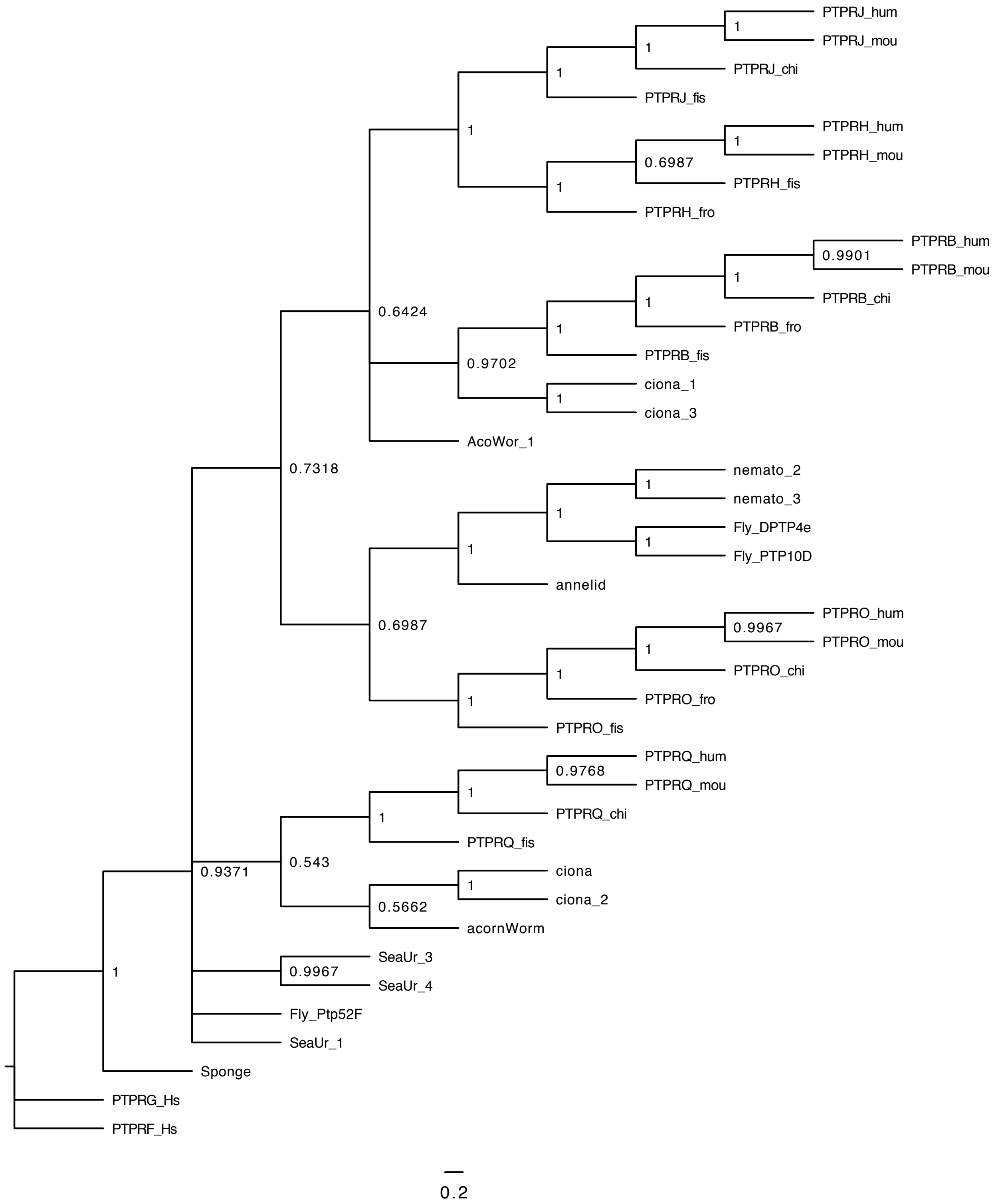
PTPRJ_hum
PTPRJ_mou
PTPRJ_chi
PTPRJ_fis
PTPRH_hum
PTPRH_mou
PTPRH_fis
PTPRH_fro
PTPRB_hum
PTPRB_mou
PTPRB_chi
PTPRB_fro
PTPRB_fis
ciona_1
ciona_3
AcoWor_1
nemato_2
nemato_3
Fly_DPTP4e
Fly_PTP10D
annelid
PTPRO_hum
PTPRO_mou
PTPRO_chi
PTPRO_fro
PTPRO_fis
PTPRQ_hum
PTPRQ_mou
PTPRQ_chi
PTPRQ_fis
ciona
ciona_2
acornWorm
SeaUr_3
SeaUr_4
Fly_Ptp52F
SeaUr_1
Sponge
PTPRG_Hs
PTPRF_Hs
0.9901
0.6987
0.6424
0.9702
0.7318
0.6987
0.9967
0.9768
0.9371
0.543
0.5662
0.9967
0.2

Figure S1.10

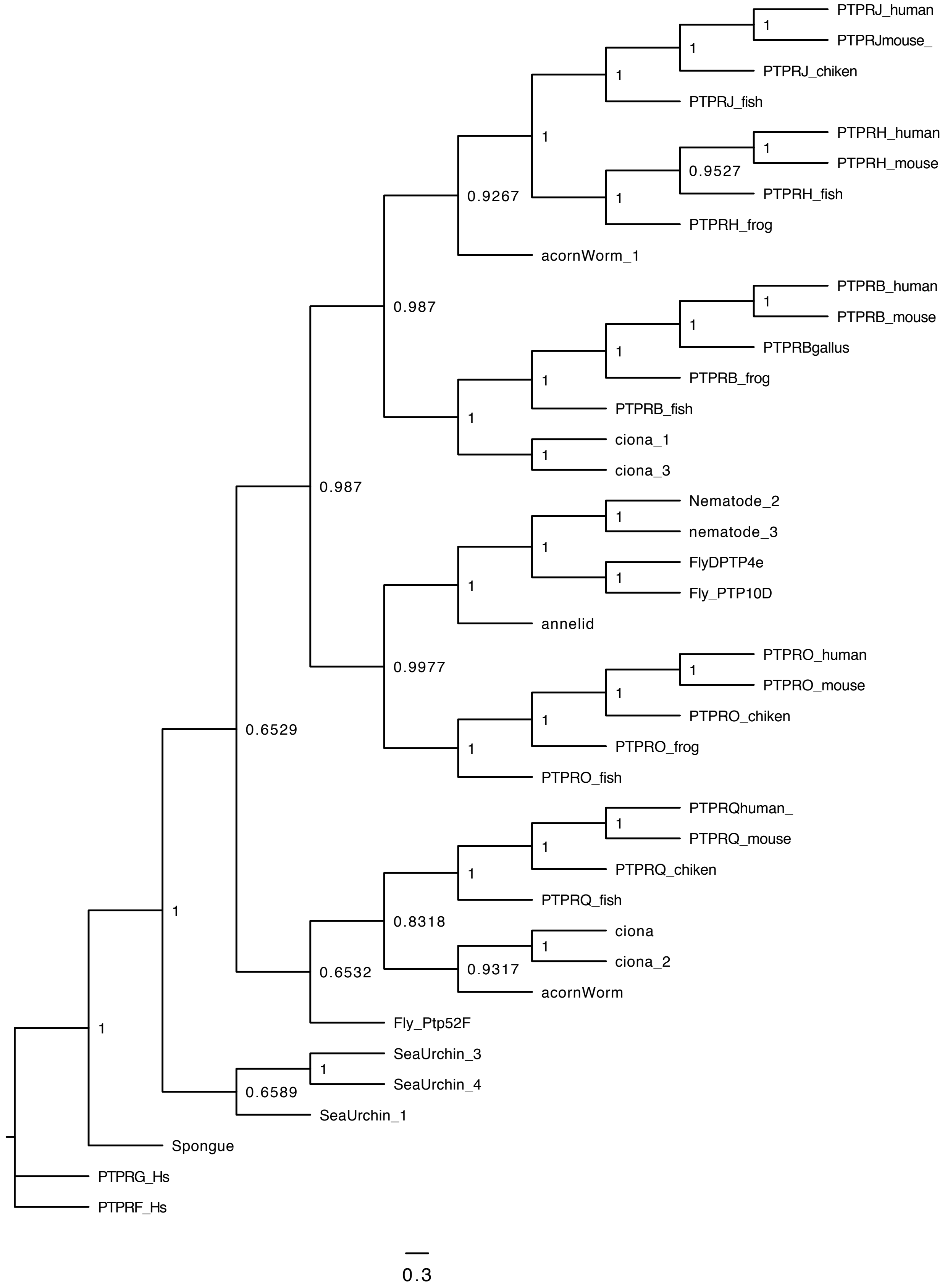
PTPRJ_human
PTPRJmouse_
PTPRJ_chiken
PTPRJ_fish
PTPRH_human
PTPRH_mouse
PTPRH_fish
PTPRH_frog
acornWorm_1
PTPRB_human
PTPRB_mouse
PTPRBgallus
PTPRB_frog
PTPRB_fish
ciona_1
ciona_3
Nematode_2
nematode_3
FlyDPTP4e
Fly_PTP10D
annelid
PTPRO_human
PTPRO_mouse
PTPRO_chiken
PTPRO_frog
PTPRO_fish
PTPRQhuman_
PTPRQ_mouse
PTPRQ_chiken
PTPRQ_fish
ciona
ciona_2
acornWorm
Fly_Ptp52F
SeaUrchin_3
SeaUrchin_4
SeaUrchin_1
Spongue
PTPRG_Hs
PTPRF_Hs
0.9267
0.9527
0.987
0.987
0.9977
0.6529
0.8318
0.9317
0.6532
0.6589
0.3

Figure S1.11

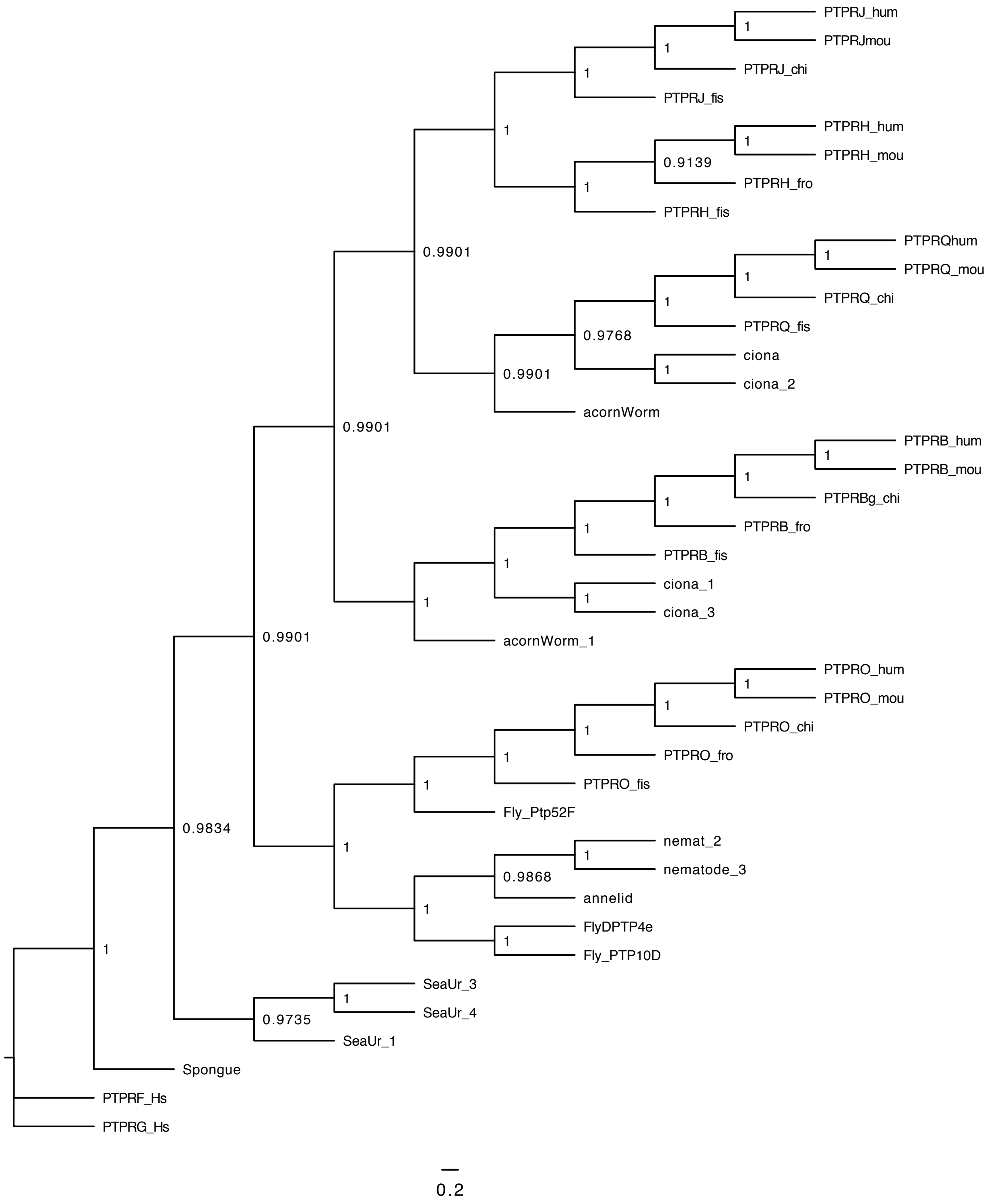
PTPRJ_hum
PTPRJmou
PTPRJ_chi
PTPRJ_fis
PTPRH_hum
PTPRH_mou
PTPRH_fro
PTPRH_fis
PTPRQhum
PTPRQ_mou
PTPRQ_chi
PTPRQ_fis
ciona
ciona_2
acornWorm
PTPRB_hum
PTPRB_mou
PTPRBg_chi
PTPRB_fro
PTPRB_fis
ciona_1
ciona_3
acornWorm_1
PTPRO_hum
PTPRO_mou
PTPRO_chi
PTPRO_fro
PTPRO_fis
Fly_Ptp52F
nemat_2
nematode_3
annelid
FlyDPTP4e
Fly_PTP10D
SeaUr_3
SeaUr_4
SeaUr_1
Spongue
PTPRF_Hs
PTPRG_Hs
0.9901
0.9139
0.9768
0.9834
0.9868
0.9735
1
0.2

Figure S1.12

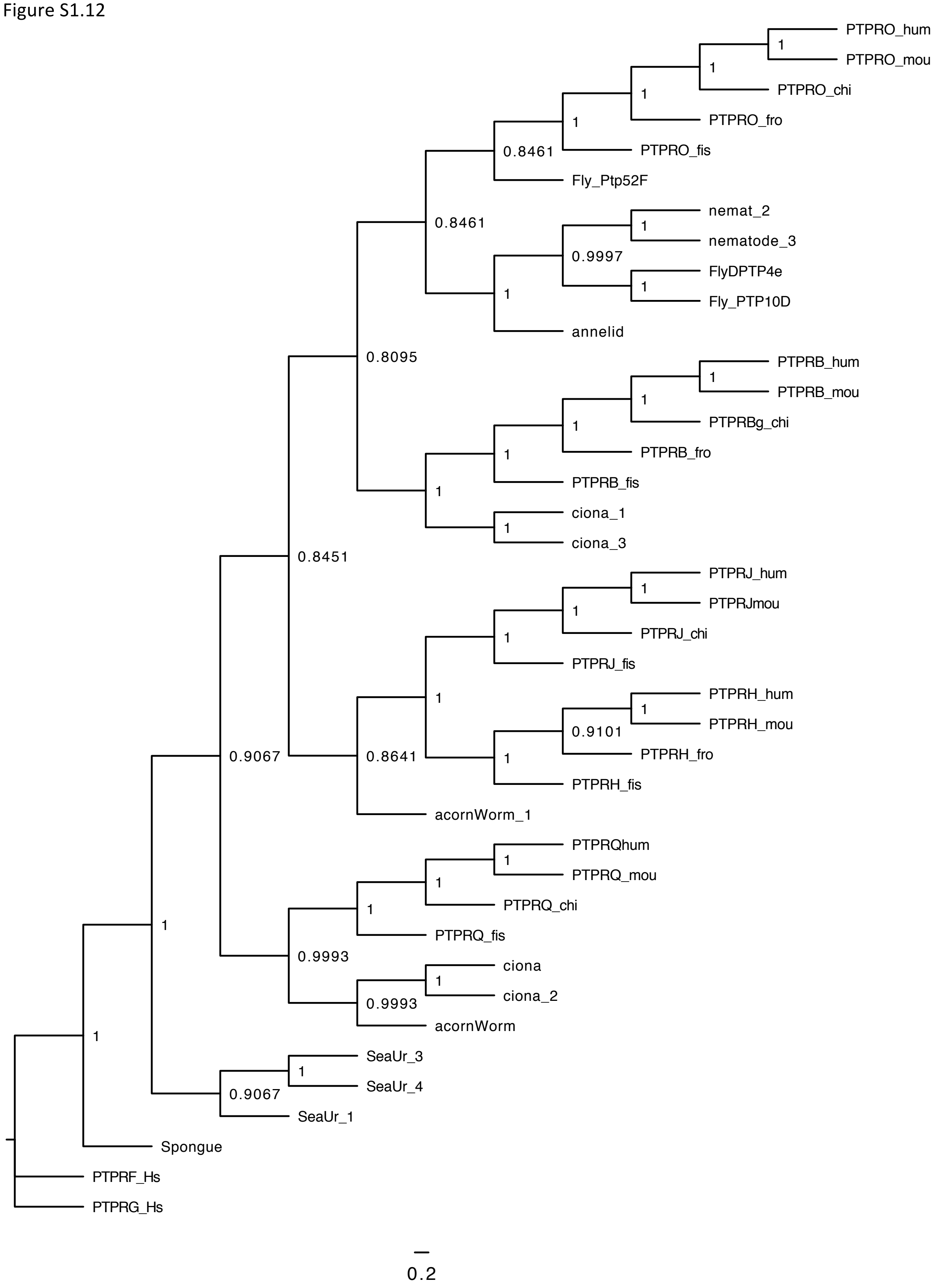
PTPRO_hum
PTPRO_mou
PTPRO_chi
PTPRO_fro
PTPRO_fis
Fly_Ptp52F
nemat_2
nematode_3
FlyDPTP4e
Fly_PTP10D
annelid
PTPRB_hum
PTPRB_mou
PTPRBg_chi
PTPRB_fro
PTPRB_fis
ciona_1
ciona_3
PTPRJ_hum
PTPRJmou
PTPRJ_chi
PTPRJ_fis
PTPRH_hum
PTPRH_mou
PTPRH_fro
PTPRH_fis
acornWorm_1
PTPRQhum
PTPRQ_mou
PTPRQ_chi
PTPRQ_fis
ciona
ciona_2
acornWorm
SeaUr_3
SeaUr_4
SeaUr_1
Spongue
PTPRF_Hs
PTPRG_Hs
0.8461
0.8461
0.9997
0.8095
0.8451
0.9101
0.9067
0.8641
0.9993
0.9993
0.9067
1
0.2

Figure S1.13

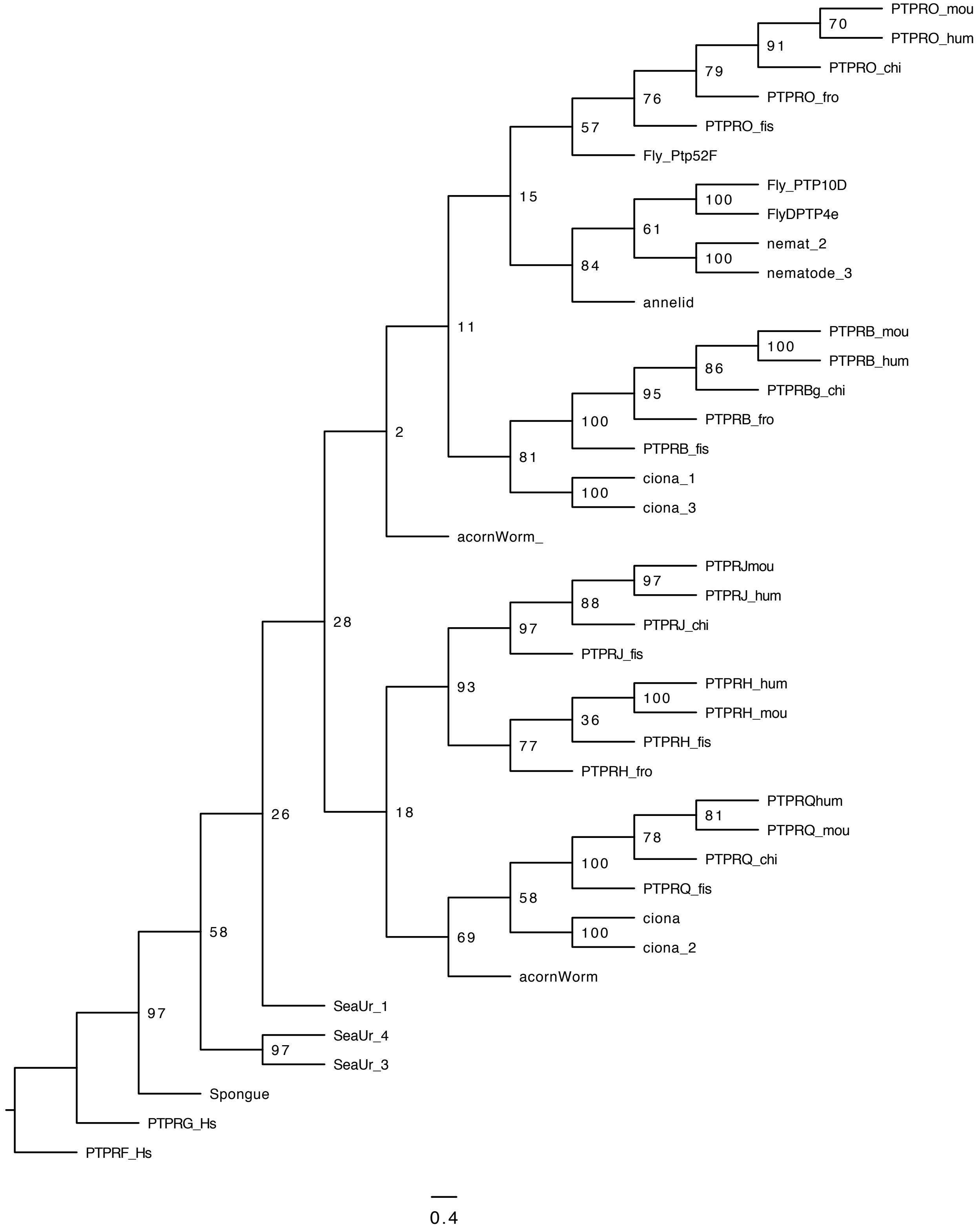
PTPRO_mou
70
PTPRO_hum
91
PTPRO_chi
79
PTPRO_fro
76
PTPRO_fis
57
Fly_Ptp52F
15
Fly_PTP10D
100
FlyDPTP4e
61
nemat_2
100
nematode_3
84
annelid
11
PTPRB_mou
100
PTPRB_hum
86
PTPRBg_chi
95
PTPRB_fro
100
PTPRB_fis
81
ciona_1
100
ciona_3
2
acornWorm_
PTPRJmou
97
PTPRJ_hum
88
PTPRJ_chi
97
PTPRJ_fis
28
93
PTPRH_hum
100
PTPRH_mou
36
PTPRH_fis
77
PTPRH_fro
26
18
PTPRQhum
81
PTPRQ_mou
78
PTPRQ_chi
100
PTPRQ_fis
58
ciona
100
ciona_2
58
69
acornWorm
SeaUr_1
97
SeaUr_4
97
SeaUr_3
Spongue
PTPRG_Hs
PTPRF_Hs
0.4

Figure S1.14

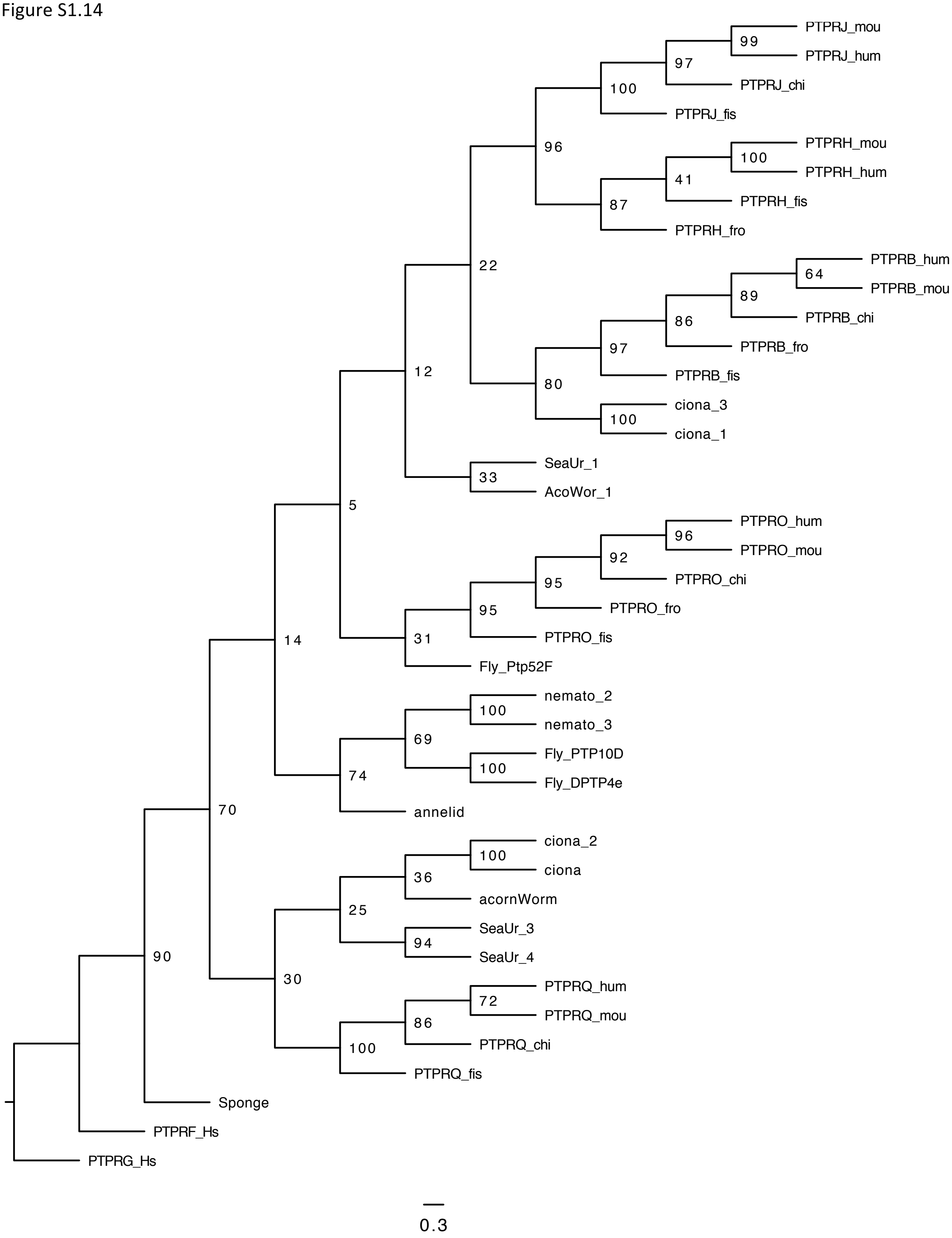
PTPRJ_mou
PTPRJ_hum
PTPRJ_chi
PTPRJ_fis
PTPRH_mou
PTPRH_hum
PTPRH_fis
PTPRH_fro
PTPRB_hum
PTPRB_mou
PTPRB_chi
PTPRB_fro
PTPRB_fis
ciona_3
ciona_1
SeaUr_1
AcoWor_1
PTPRO_hum
PTPRO_mou
PTPRO_chi
PTPRO_fro
PTPRO_fis
Fly_Ptp52F
nemato_2
nemato_3
Fly_PTP10D
Fly_DPTP4e
annelid
ciona_2
ciona
acornWorm
SeaUr_3
SeaUr_4
PTPRQ_hum
PTPRQ_mou
PTPRQ_chi
PTPRQ_fis
Sponge
PTPRF_Hs
PTPRG_Hs
99
97
100
96
100
41
87
22
64
89
86
97
80
100
12
33
5
96
92
95
95
31
14
100
69
100
74
70
100
36
25
94
90
30
72
86
100
0.3

Figure S1.15

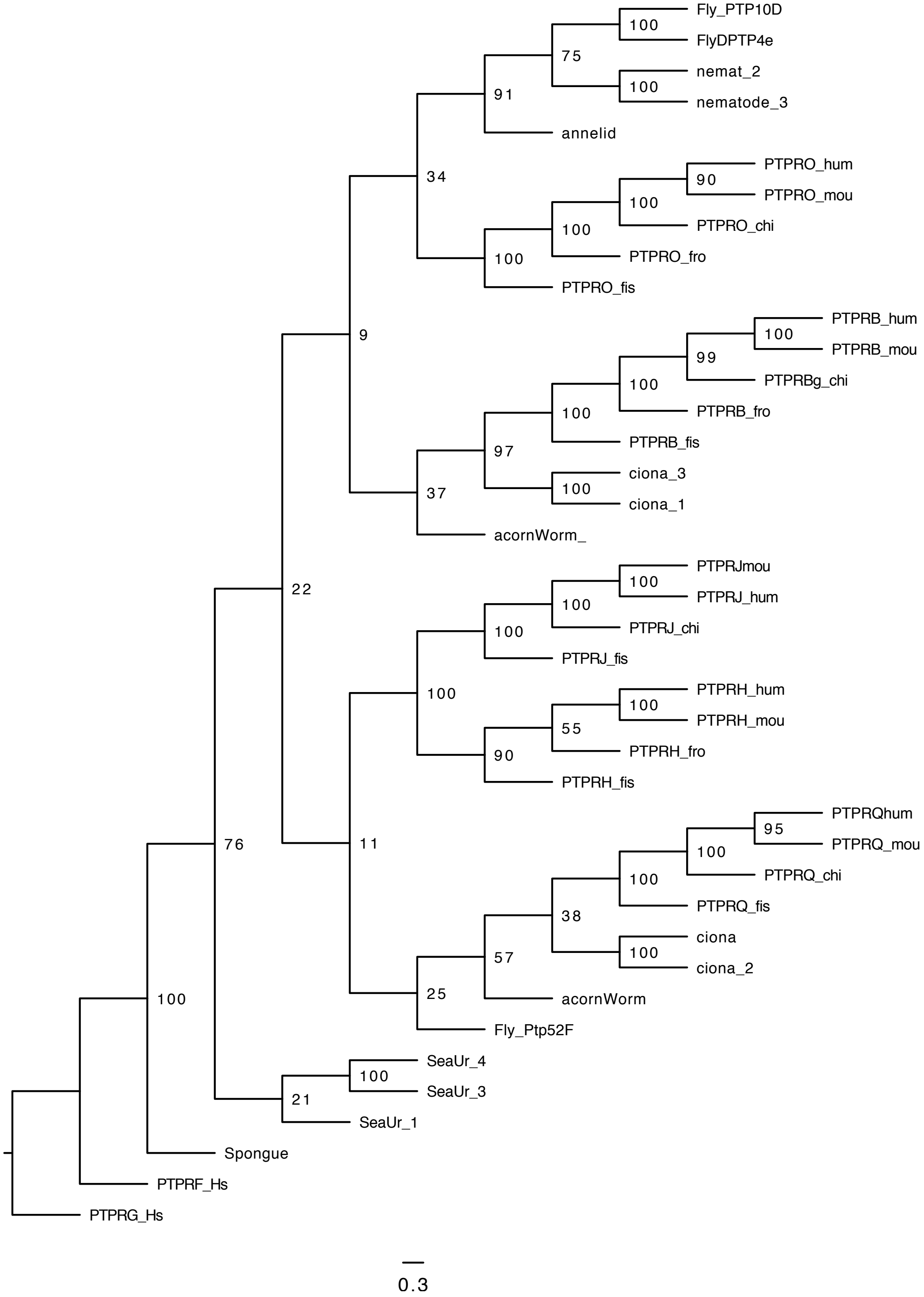
Fly_PTP10D
FlyDPTP4e
nemat_2
nematode_3
annelid
PTPRO_hum
PTPRO_mou
PTPRO_chi
PTPRO_fro
PTPRO_fis
PTPRB_hum
PTPRB_mou
PTPRBg_chi
PTPRB_fro
PTPRB_fis
ciona_3
ciona_1
acornWorm_
PTPRJmou
PTPRJ_hum
PTPRJ_chi
PTPRJ_fis
PTPRH_hum
PTPRH_mou
PTPRH_fro
PTPRH_fis
PTPRQhum
PTPRQ_mou
PTPRQ_chi
PTPRQ_fis
ciona
ciona_2
acornWorm
Fly_Ptp52F
SeaUr_4
SeaUr_3
SeaUr_1
Spongue
PTPRF_Hs
PTPRG_Hs
100
75
100
91
34
90
100
100
100
9
100
99
100
100
97
100
37
22
100
100
100
100
100
55
90
11
95
100
100
38
100
57
25
76
100
21
100
0.3

Figure S1.16

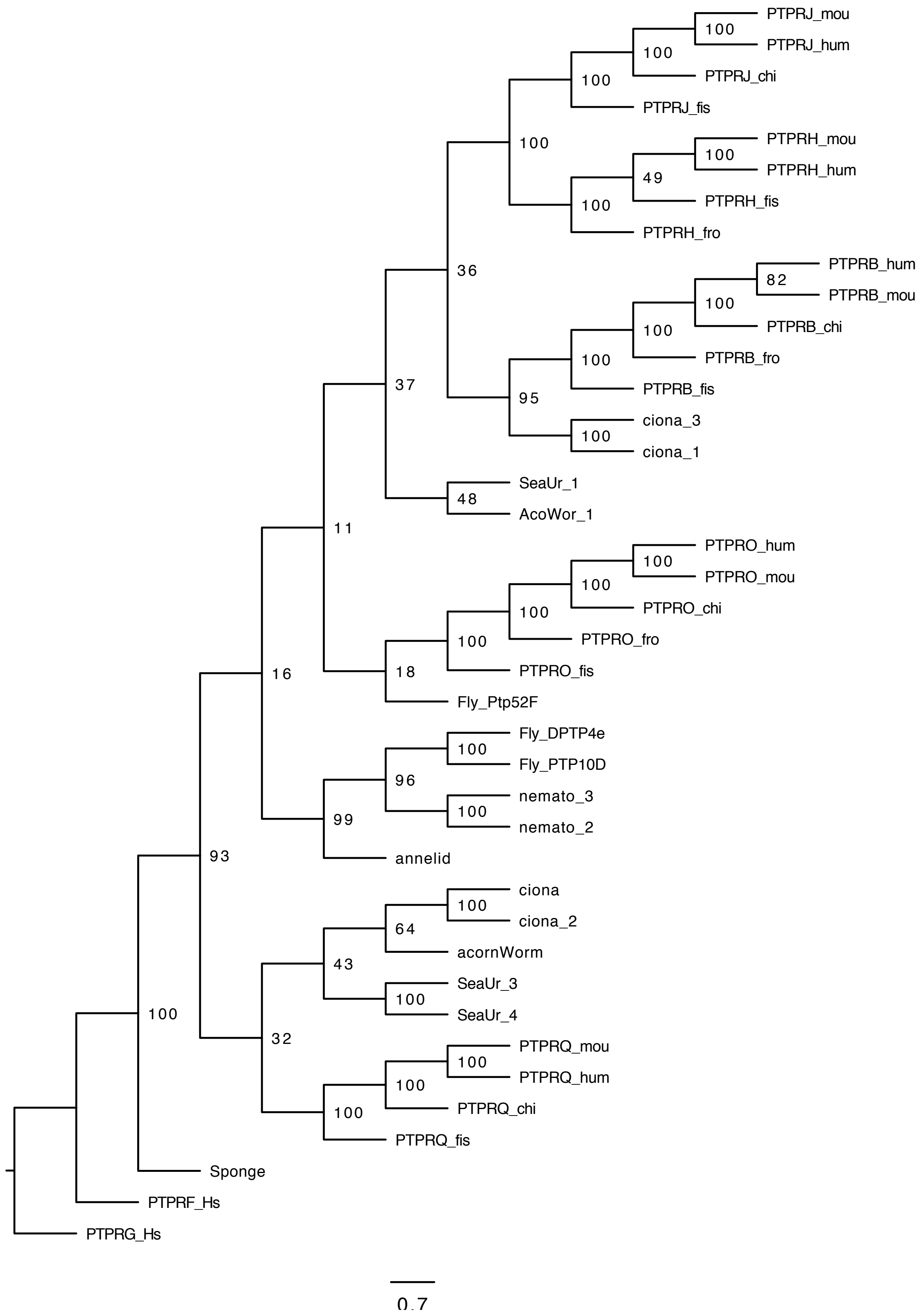
PTPRJ_mou
100
PTPRJ_hum
100
PTPRJ_chi
100
PTPRJ_fis
100
PTPRH_mou
100
PTPRH_hum
49
PTPRH_fis
100
PTPRH_fro
36
PTPRB_hum
82
PTPRB_mou
100
PTPRB_chi
100
PTPRB_fro
100
PTPRB_fis
95
ciona_3
100
ciona_1
37
SeaUr_1
48
AcoWor_1
11
PTPRO_hum
100
PTPRO_mou
100
PTPRO_chi
100
PTPRO_fro
100
PTPRO_fis
18
Fly_Ptp52F
16
Fly_DPTP4e
100
Fly_PTP10D
96
nemato_3
100
nemato_2
99
annelid
93
ciona
100
ciona_2
64
acornWorm
43
SeaUr_3
100
SeaUr_4
32
PTPRQ_mou
100
PTPRQ_hum
100
PTPRQ_chi
100
PTPRQ_fis
100
Sponge
PTPRF_Hs
PTPRG_Hs
0.7
