# Supplementary material for: Phosphotyrosine phosphatase R3 receptors: Origin, evolution and structural diversification
Source: PLoS One. 2017 Mar 3;12(3):e0172887. doi: 10.1371/journal.pone.0172887 (PMC5336234; doi:10.1371/journal.pone.0172887)
Supplement: S2 Fig — Alignment of B-C loop region of all FN3 domains. Domains are numbered from the one closest to the transmembrane as in S1 File. Only the human proteins are shown within the vertebrate clade. Fibronectin FN3 10th repeat is depicted in the top of the alignments and the B-C β-strands are highlighted in magenta. Tryptophan (W) in strand B and tyrosine (Y) in strand C are highlighted in black. The conserved glycines inside the B-C loop are shaded in blue. (PDF) [file pone.0172887.s002.pdf]

```
                                     B                 C
d1fnf_4           VS-DVPRDLEVVAA--TPTSLLISWDAPA---VTVRYYRITYGETGGNS-------PVQE
Hs.Q.16.orange    KP-GPPVFLAGERV--GSAGILLSWNTPPNPNGRIISYIVKYKEVCPWM-----QTVYTQ
Hs.Q.15.orange    AP-GKVVNLTVEAY--NASAVKLIWYLPRQPNGKITSFKISVKHARSGI-----------
Hs.Q.14.blue      VPEGPPQNCVTGNI--TGKSFSILWDPPTIVTGKF-SYRVELYGPSGRI-----------
Hs.Q.13.whiteLO   VP-GAVFDLQLAEV--ESTQVRITWKKPRQPNGIINQYRVKVLVPETGI--------ILE
Hs.Q.12.blue      VP-SSIKIINYKNI--SSSSILLYWDPPEYPNGKITHYTIYAMELDTNR----------A
Hs.Q.11.yellow    EP-ESSPQDVEVID--TADEIRLKWSPPEKPNGIIIAYEVLYKNIDTLY-----------
Hs.Q.10.blue      VPDSAPENITYKNI--SSGEIELSFLPPSSPNGIIQKYTIYLKRSNGNE----------E
Hs.Q.9.white      APDSPPQDFSVKQL--SGVTVKLSWQPPLEPNGIILYYTVYVWNRSSLK-----------
Hs.Q.8.orange     APSDPPKDVYYANL--SSSSIILFWTPPSKPNGIIQYYSVYYRNTSGTF--------MQN
Hs.Q.7.yellow     IPEGFVGNLTYESI--SSTAINVSWVPPAQPNGLVFYYVSLILQQTPRH---------VR
Hs.Q.6.yellow     VPETSPIINTFKNL--SSTSVLLSWDPPVKPNGAIISYDLTLQGPNENY-----------
Hs.Q.5.orange     VPLAPPQNLTLINC--TSDFVWLKWSPSPLPGGIVKVYSFKIHEHETDT--------IYY
Hs.Q.4.yellow     VP-DVVQNMQCMAT--SWQSVLVKWDPPKKANGIITQYMVTVERNSTKV-----------
Hs.Q.3.yellow     VP-SVPTNIAFSDV--QSTSATLTWIRPDTILGYFQNYKITTQLRAQKC-----KEWESE
Hs.Q.2.orange     PPDGPPENVHVVAT--SPFSISISWSEPAVITGP-TCYLIDVKSVDNDE------FNISF
Hs.Q.1.orange     APKDPPNNMTFQKIPDEVTKFQLTFLPPSQPNGNIQVYQALVYREDDPT-----------

Hs.B.16.white     AE-PERCNFTLAESKASSHSVSIQWRIL----GSPCNFSLIYSSDTLGA--------ALC
Hs.B.15.yellow    DP-LPPARFGVSKEKTTSTSLHVWWTPSS---GKVTSYEVQLFDENNQK------IQGVQ
Hs.B.14.yellow    VP-SPVKDIGIST---KANSLLISWSHGS---GNVERYRLMLMDKGILV---------HG
Hs.B.13.yellow    AP-MEVSNLKVTNDG-SLTSLKVKWQRPP---GNVDSYNITLSHKGTIK---------ES
Hs.B.12.yellow    FP-DKVANLEANNNG-RMRSLVVSWSPPA---GDWEQYRILLFNDSVVL---------LN
Hs.B.11.yellow    VP-LAVLQLRVKHA--NETSLSIMWQTPV---AEWEKYIISLADRDLLL---------IH
Hs.B.10.yellow    VP-AQVTDLHVANQG-MTSSLFTNWTQAQ---GDVEFYQVLLIHEN-VV--------IKN
Hs.B.9.yellow     VP-SSVSGVTVNNSG-RNDYLSVSWLLAP---GDVDNYEVTLSHDGKVV---------QS
Hs.B.8.yellow     VP-DKVQGVSVSNSA-RSDYLRVSWVHAT---GDFDHYEVTIKNKNNFI---------QT
Hs.B.7.yellow     IP-EPVKDLTLRNR--STEDLHVTWSGAN---GDVDQYEIQLLFNDMKV--------FPP
Hs.B.6.yellow     VP-SAVKNIHISPNG-ATDSLTVNWTPGG---GDVDSYTVSAFRHSQKV---------DS
Hs.B.5.yellow     VP-ASVQGVIADNAY-SSYSLIVSWQKAA---GVAERYDILLLTENGIL--------LRN
Hs.B.4.yellow     VP-AAVTDLRITEN--STRHLSFRWTASE---GELSWYNIFLYNPDGNL--------QER
Hs.B.3.yellow     VP-ASVSHLRGSNRN-TTDSLWFNWSPAS---GDFDFYELILYNPNGTK---------KE
Hs.B.2.yellow     AP-SPPSLMSFADI--ANTSLAITWKGPPDW-TDYNDFELQWLPRDALT--------VFN
Hs.B.1.yellow     KP-DKIQNLHCRPQ--NSTAIACSWIPPD---SDFDGYSIECRKMDTQE-------VEFS

Hs.H.8.Yellow     AP-NPGRNLTVETQ--TTSSISLSWEVPDGLDSQNSNYWVQCTGDGGTT-----------
Hs.H.7.Yellow     AP-NPVRNLRVEAQ--TNSSIALTWEVPDGPDPQNSTYGVEYTGDGGRA-----------
Hs.H.6.Yellow     AH-NPVRNLRVEAQ--TTSSISLSWEVPDGTDPQNSTYCVQCTGDGGRT-----------
Hs.H.5.Yellow     AP-NPVRNLTVEAQ--TNSSIALTWEVPDGPDPQNSTYGVEYTGDGGRA-----------
Hs.H.4.Yellow     AP-NPVRNLHMETQ--TNSSIALCWEVPDGPYPQDYTYWVEYTGDGGGT-----------
Hs.H.3.Yellow     VP-NAVTSLSKQDW--TNSTIALRWTAPQGPGQSSYSYWVSWVREGMTD----------P
Hs.H.2.Yellow     AP-NEVTDLQNETQ--TKNSVMLWWKAPGDPHSQLYVYWVQWASKGHPRRGQDPQANWVN
Hs.H.1.Yellow     YP-DTVTITSCVSTS-AGYGVNLIWSCPQG---GYEAFELEVGGQRGSQ-----------

Hs.J.8.yellow     GP-SPVFDIKAVSI--SPTNVILTWKSND---TAASEYKYVVKHKMENE----------K
Hs.J.7.BlueLONG   EP-IPVSDLRVALT--GVRKAALSWSNGN---GTASC-RVLLESIGSHE-----------
Hs.J.5.yellow     NA-IQVFDVTAVNI--SATSLTLIWKVSDNESSSNYTYKIHVAGETDSS-----------
Hs.J.4.yellow     PP-VPVSDFRVTVV--STTEIGLAWSSHD-----AESFQMHITQEGAGN----------S
Hs.J3.yellow      VP-SAVFDIHVVYV--TTTEMWLDWKSPDG--ASEYVYHLVIESKHGSN-----------
Hs.J.2.yellow     RP-SNVSNIDVST---NTTAATLSWQNFDDA-SPTYSYCLLIEKAGNSS-------NATQ
Hs.J.1.yellow     DP-ASMASFDCEVVP-KEPALVLKWTCPPG---ANAGFELEVSSGAWNN------ATHLE
                                          :


                                  B                        C
d1fnf_4           VSDVPRDLEVVAATPTS-LLISWD-APAV--------TVRYYRITYGETGGNS-PVQEFT
Hs.O.2.orange     -APVAPEITSVEYFNSL-LYISWT-------YGDDTTDLSHSRMLHWMVVAEGKKKIKKS
Hs.O.4.orange     -TEKPQHVSVHVLSSTT-ALMSWT-SSQEN-Y--NSTIVSVVSLTCQKQKESQRLEKQYC
Hs.O.5.white      ----KSTSGSFSFFPVQ-MILTWL-PPKP------PTAFDGFHIHIEREE----NFTEYL
Hs.O.3.orange     VPTGIKDLMLYPLGPTA-VVLSWT-RPYLGVF--RKYVVEMFYFNPATMTSEWTTYYEIA
Hs.o.1.orange     EPAPPKSLFAVNKTQTS-VTLLWV-EEGV---------ADFFEVFCQQVGSSQKTKLQEP
```

```
                                                    B                 C
d1fnf_4          ----VSDVPRDLEVV---AATPTSLLISWD-AP---AVTVRYYRITY-GETGGNS-PVQE
ciona.24         -----LAPPRSLHVNH-TSITEESFEIAWK--SA---VGLVEKYRIVL-KTKSADE--TKI
ciona.23.white   RAISDMAPPVNFRMT---SRGETEITLGWD--PAPGVQGV---YMVRC-EGFQEPC-PAEV
ciona.22.yelow   ----VPRTPLSLDTV---SRSDTTISLRWD--QP---TGILDGYKISY-TSRGGQL-EILF
ciona.21.white   ----RPLPPNDVRMD---NLTKSSVHVWWR--EPETFDQFLLVYKDDF-NTKNLMT-----
ciona.20.white   -----LAPPRSLHVNH-TSITEESFEIAWK--SA---VGLVEKYRIVL-KTKSADE--TKI
ciona.19.white   ----DMAPPVNFRMT---SRGETEITLGWD--PAPGVQGV---YMVRC-EGFQEPC-PAEV
ciona.18.yelow   ----VPRTPLSLDTV---SRSDTTISLRWD--QP---TGILDGYKISY-TSRGGQL-EILF
Ciona.17.Large.  -----PLPPNDVRMD---NLTKSSVHVWWR--EPETFDQFLLVYKDDF-NTKNLMT-----
CIONA.16.orange  YIIAEPGTPTALVLK---PVSTTELLTEWE--IPLNPNGIIRRYIIRF-KQNYPHP-STNY
CIONA.15.white   ------GPVSDLHVS---SVNAISATVAWQ--PPEQPNGEIIRYVLNI-STDHLPV-----
CIONA.14.white   ----PDDPPRHVRLT---SATHTSLNVTWF--PPNQPNGIVT-YTIAV-LQSDKMF-QSNL
ciona.13.yelow   ---RPGS-VQNLTAQ---VETSRSVTLQWN--PPINPNGVITGYWIFA-KVKNVTQ-----
ciona.12.yelow   --QDVPSPPINLSYY---NVSSTAVNITWD--EPLEANGVIIKYVVYY-MTKDTFL-DVTV
ciona.11.yellow  ----PSSPPINLSYY---NVSSTAVNITWD--EPLEANGVIIKYVVYY-MTKDTFL-DVTV
ciona.10.blue    ----PATPPFNVSYQ---NLTSTKVRLTWR--RPLVPNGIIQFYEISL-TSKNNKT-IRAT
ciona.9.yelow    ---VPGSPVTNVTYV---NLTSSSIGLEWS--LPKEPNGKILKYSIRY-SMLEGNM-NQYK
ciona.8.white    ---EPESAPYDIIFQ---QYNSTTIALTWR--PPVKPNGIIVNYTVYY-SNEDKVM-TKTT
ciona.7.yelow    ---APADPPRDVVVK---ALSSTSISVGWS--TPATPNGQIQFYTVFY-TDKISAV-HATN
ciona.6.orange   ---VPDNGVSDLSAL---AINATTIKVTWQ---PGIPLTGPTFFHIQY-VLNNTPV-----
ciona.5.orange   -----QDAVTDIQLI---NLTSDSALIKWL--APRQPNGVITHYTVHY-GRNSTIQ-----
ciona.4.yelow    ----APTAPTSLNCS---VASNFSVKISWD--LPMRTNGIIRGYNLEY-RSMDKNK-----
ciona.3.orange   ----YADAPPQLQLK---NLSDRSITIEWS--RPLQWNGRFHGYLITY-KPPDSCPNPAND
ciona.2.orange   ---VPDSPVRDVHVT---VVSSTSINVTWN--LPSSYAGP-TTYKLYS-EL----------
ciona.1.orange   ---APSDFPESVGVAT-VLNNSSSVHVLFK--EPLDPNGKLINYTIQH-RRLEDSK-LQSI

ciona1.17.white  ----AVDAPSGITVD---NIKSTEFTVSWT-TP----TDAISKMTVDI-AGTASNE-NDAV
ciona1.16.white  -----IFSAPVLTSVT—GTNSTIAVDLSWTYDN-GGGANAVSEYLIKW-DGGGSTG----S
ciona1.15.white  -------TSAPSSATTAGATTTS-IDLTWTAPAVGGGKNNVLAYTIQWTGGAGGS-
ciona1.14.yelow  -LPSLPEQPTLTRST---TNPTTVIDVSW—PAV--TSGTETVDYVVEW-TPDEGPA-ANKA
ciona1.13.yelow  ----NPPTPTGVSLSNP-TNGQTSLKVDWQITK---NNFVISSYEISL-TPATSGA-AVTK
ciona1.12.yelow  ----VPPTPTGVTLSQSAGDQTTSLKVDWV-MP---SLYVVSSYNISL-IPESSGSAIVTQ
ciona1.11.yelow  -VSAVPGTPNLYQPTD-GSDKTTILYANWT-VP----TGVVDSYQLLV-YLGSVGGTLVAN
ciona1.10.yelow  ----NPPTPTGVTLSQPNVNQTTSLKVDWVMIP---TSFVISSYIISL-ISTTSGA-VVNK
ciona1.9.yelow   ----DPPTPTGVQLFQPTVNQTTSLKVDWQITE---TDFVISSYDITL-TPSTSGA-AVTK
ciona1.8.yelow   ----DPPTPTNVTLSKPTVNQTTSLKVDWHITE---AYFVVSSYEISLKITGTTGA-AVTK
ciona1.7.yelow   -DPGVPGIPTLSQPTD-GSDKTTILYANWT-AP----TGVVDSYKLLV-YRGDVGGTVDAN
ciona1.4-5.whit  ----ITKGVSNLMAQ---SQTTNSIYVTWN-PP--PMDVLFTNYFVEY-SKMNIFL-MPED
ciona1.4-5.whit  ----KPEPVTNFRST---EQTSYSIYMSWD-LP---TLGIFQSQNVTY-VPSDSSE-NAIV
ciona1.3.yelow   ----GPPVATNISIT---ASNTTCVHGT---II-YVGRGIIHFYSLTW-GFHGSKL-----
ciona1.2.white   ----EPNSVLSVMLS---SVSITEISVQWT-----RNSGGVSGYEVIA-TNSNCVV-EANI

ciona2.10.yelow  --DAPGSQVMNVSYV---NLTSTSVNVSWL--QPAEPNGRITKYKIES-KLK---------
ciona2.9.white   ---EPSSPPDGIFFH---QHNSTSISLTWN--PPLTPNGIITLYSVHY-RHGNKSL-IRTT
ciona2.8.orange  ---APADPPHDVRVA---AISSTSINVTWS--PPTTPNGLIQFYTVYY-QHNPSTV-QTKN
ciona2.7.white   ---VPEQKVQELSPR---VVNSTAIRVTWL---PGQPLTGVTYFMVNV-STKT--------
ciona2.6.white   ----PSSPVRNISII---TITQTTVTITFL--PPTKSNGVITSYSIMT-SLDHKQL-VNGS
ciona2.5.white   ----PEDSPQKVQIT---NITDQSASLHWE--PPTLPNGIIRTYTISY-QSNESQR-NFNS
ciona2.4.yelow   ----EPSAPTNLECK---IYDNYKILVTWD--LPLLSNGRVIAYKLSH-SVLNDTQ-----
ciona2.3.yellow  ----YATAPQDLNIN---KTSDVTITIQWN--KPNNVNGRLNGYVIMY-IPFDACQ-PNSG
ciona2.2.orange  ---VPEHSVENLQAI---VTSSTTVNVTWE--SPESFAGP-TTYKVEA-FHSTTMQ-----
ciona2.1.orange  ---VPSDFPRDVNVAI-VMGNSSSVRVLFK--PPRDPNGVLTNYTIQF-KRLENSE-SRNA

ciona3.12.yello  ----PKPTAVTLSQP--TSNKTTSLEVTWSMIGTQ---FIVSSYIINLTP--ANS--GSNAP
ciona3.9.yellow  -----PPKPTAVTLSQPTSDKTTSLEVTWSMIGTE---YIVSSYIINLTP--ANS-GSNAPT
ciona3.8.yellow  --------PTAVTLSQPTSVKTTSLKVTWSMITTE---YIVSSYIINLTP--ANS-GSNAQI
ciona3.11.yello  -----PPKPTAVTLSQPTSNKTTSLEVTWSMIGTE---YIVSSYIINLTP-ANS--ASNAPT
ciona3.10.yello  --------------------PTTSLVVTWVKPA-----GLVDTYGVTLYN--E---GSLNNP
ciona3.15.yello  -----FVAPTLNPPTNP---TSSSIDLSWTAPGNGGGAVTVTGYTITYTG-G----GGGTQD
ciona3.13.yello  -----PAAPPSLLPGSN---ATNMIDVSWTAPTGG---NVIVDYVVEWMA-H----STTATE
ciona3.5.orange  -----PEAVTRLLKDSS---TTESISITWTLPTA----GLFDKQIVEVNK-V----NTPDTN
ciona3.3.yellow  -----PNPVLSMMATPS---NMNDIFVEWLNNT-----GGVEGYEVQASL-S----GIVRSN
ciona3.14.yello  ----PDKVVLAANTTTA---TIDTIGLNWNPVSG----GVPVTYTVTWSL------PTSGSP
ciona3.4.yellow  -------PPTKLQAAAN---STSSIIVSWSPPIS----GAWWYYVLTYYN-S----THPYTI
ciona3.2.orange  -----PDSVKNLKVLKSSTKPTTELDLTWDQPA-----GDGENITIMYTQ-N----PGGLVT
ciona3.16.yello  -  -NAQPAAPTGLSVTSS--KSNQLTISWTDTS-----G--TTYKVYVGTGPANVADAVDK
ciona3.7.yellow  -------PNPPASFTVNPVA--ANNEITFSFTAPVTP--NVFSHFKLTTNA-T---GTTNTT
ciona3.6.yellow  ----------------------PIQASVSFTPPQ----NGATMYQANLTD------TSGG-
```

```
                                              B                 C
d1fnf_4          VSDVPR-----DLE---VVAATPTSLLISWDAPAVT---VRYYRITYGET-GGNSPV--
seaurchin1.19.y -----------DIVTVENEGNTDSLKVTWTPPTGA----TDQVVTWSPADGSG-----
seaurchin1.18.y KPSPV-------NLTE--SGATNDSISAEWTKPSG---AIDQYTFSCSEGTEDP-----
seaurchin1.17.y LPNMV-------HIEA--GDATNSSFVASWNHPEG---EMDSFQVFCYENDTAPEE---
seaurchin1.16.y LPNPV-------ELTA--SDSAVDSVRATWLKPAG---EVTSYEVQCSNGT--------
seaurchin1.15.y LPEAV-------TLNEERGMVTTTTIAASWTMPSG---IVEYYDVFCLVGD--------
seaurchin1.14.y LPLSA-------NLTA--GNSTINSVSAMWPYPGG---LVDVFQVNCSNGT--------
seaurchin1.13.y -PESV-------MLNEERDRVNTSTITASWTMANG---IVDYYQVSCSNGT--------
seaurchin1.12.y LPSQV------SDLAE--SGATTSSVSATWTKPDG---VVSSYTVTCPDGS--------
seaurchin1.11.y LPSQV------TDLAE--SGATTSSVSATWTKPDG---VVSSYTVTCPDGS--------
seaurchin1.10.y LPSQV------TDLAE--SGATTSSVSATWTKPDG---VVSSYTVTCPDGS--------
seaurchin1.9.ye FPNSV-------VLRE--DGVTTRQITVQWVDPVG---EEDSFIVDCGDDG--------
seaurchin1.8.ye TPDGV-------TFEE--GSASTTAVTVTWIALA----RADGFRVNCSEGTPSHEGT--
seaurchin1.7.ye LPVSV-------NLTA--GGSTTSSVTATWDIPGG---IVDEFEVECSNGT--------
seaurchin1.6.ye LPESV-------SLNEAIDMVTTTTITASWTKPNG---IVDYYEVSCSNGD--------
seaurchin1.5.ye LPEAV-------ELSE--GESNTTVISATWTVRNS---VVDTFNITCSDGT--------
seaurchin1.4.ye LPASV------ESIAV--TEPTTTTVDVQWNLTQCSECVYNYFLLTFQPDS--------
seaurchin1.3.ye VPEAV------GNISV--TQYATTTADVQWNLMQCSDCIYNYFLLTFEPDS--------
seaurchin1.2.ye VPATP------SDLSI---VPGQRELNLVWAYQG----DADNFTITVTPNQGI------
seaurchin1.1.wh KPSPV------GNPQA--EAVDKNTITLTYESPLEPNGDITGYRISYIGTRDGNTD---

seaUrchin3.20.o LPSAV------SDVDV---VPEAAALTLSWDPVFG---DVDGYHASYQLKENPYQ----
seaUrchin3.19.y VNYSP------AEIVI--ETVTSTSIALSWGASTSP--TASSYVIEASDAGNV------
seaUrchin3.18.y VPTAP------RSLST--DSISQSIVTVSWSQGLG---NVDEYDIYLRQAGES------
seaUrchin3.17.y QPRII-----------NVVETGRNLRLSWANDFGN--DILKYQLIYIEDKEGAT----
seaUrchin3.16.y KPLRP-------TFTV--NRRSPSAVSLLGNLLGG---ILKFFRIRVTPQVTGAGA---
seaUrchin3.15.y PPLTQQ-----GELSF--GEITPSGFTVYWTPFLGR----TSYELSINNQAGLVV----
seaUrchin3.14.y RPLAP------GTPLA--TQVLDTSIQLQWAQSSDG--NVAGYEICYSPDDG-------
seaUrchin3.13.y -PVAS-------NFAVTNTQAADHVIILEWDVPERA--SYDYFEVSYTPDVGYP-----
seaUrchin3.12.y ---------AVNNLIVVIGERTTTSLEILWGASASG--SVDEYQVSYVG----------
seaUrchin3.11.w LHEING-----LSAMV--TDQASSMIHLTITPPFEDPMRYDGYNVRVRLGGVSSPV---
seaUrchin3.10.w -AAAP------LDITL--DELETDSMKVSWQEGTG---SFSNYLVTYSPVGST------
seaUrchin3.9.wh LPEAI----NIDSLSV---TRSKNALDATWSAPSTG--NYDGYRVCHYPRG--------
seaUrchin3.8.wh LIGDPGD----INIAL--EDVDTNSVRITWVAIN----SITRYTVGGGTGA--------
seaUrchin3.7.ye RPDMP------RNLLF--NTITTNSISIGWEKPLTG--GVDSYRVTYSPSSDGSP----
seaUrchin3.6.ye SGTTIG-----MDIGA---GYTSTQFTVVWNVPTSG--DFNQFQVTYDPYDPTNAQ---
seaUrchin3.5.ye PPLPV------TGVVI--TQRTPYSLSLDWSDVIS---ADIQYRITYAPLEGALFPNNE
seaUrchin3.4.wh QPLSP------QNLVV--SDVGVEEVSLSWNLPAEVNFPIAYYEIDLVPNEP-------
seaUrchin3.3.wh -PTQP------QRLST--PYVTTTELRVEWTLGNS---NFTTFQVSFSKDS--------
seaUrchin3.2.or KSKPP------QNLMQ--DEVTSDSITVSWGAPLN---VVNMYHIIVKDEDGA------
seaUrchin3.1.or KPSVV------RDPVV--AINSPNSVFLTWTPPDSPNGILTGFVVEITGEDLTSRKRRE

SeaUrchin4.21.y SPLTP-------STNI--SDIKATTALLTFSLREG----IFDDYVVMLSSPTDSCGA---
SeaUrchin4.20.y LQNFTTATVVNNEVEI--SKVGTNSLEIIWKTD------NVDEYLLHYSGPDGP------
SeaUrchin4.19.y KPDPI------SNLVL---TPQENTINLTWTSPST----MYDGFLICWYSATT-------
SeaUrchin4.18.y TETSP--------LIL--KDIATDTISVSWVTIPG-----VVDYTLSYSPPDGNEQA---
SeaUrchin4.17.y NPLPI------YDPSV--TNTTSCNLLYQWCLPDS----DFDNISITIVPDVSMR-----
SeaUrchin4.16.y EPLPV------NNVTL--DDSTIFTLTISWENPIDG---SFNMFLVEYAPVRSPDS----
SeaUrchin4.15.y RQQNS------EELRL--QTRTSTSISVSWGNTTRP---EVTGFVLRITFQEGDA-----
SeaUrchin4.14.y LPGMP------GNISCDPTKTTQSLLEIEWGSTQEG---NFDGYAISTQTGDGEE-----
SeaUrchin4.13.w APLPV------VQVII--VLRRPTSFTIQITLPRRG---RYRFLRLRVRRRSQGLD----
SeaUrchin4.12.y PRMEE------GDISF--NEVTTTSISFAWGMVSADTNGPFDFYLLNLKNELSID-----
SeaUrchin4.11.y RPDKP------TDIML--SDLTTTSLTIDWKAPLT----SYQGFRLCWAYG---------
SeaUrchin4.10.y FPPAE------LHVNI--TSFNTTAVSIVWGTDIPL--LDVSSYVVSYWETANNGS----
SeaUrchin4.8.ye -PESA------VDLQF--TSIGRDYVVLTWDNPAG----MIDSYNISYYPVNDITKL---
SeaUrchin4.7.bl VVGVL------GSLNI--TAFDETTISIEWEQV------DVEEYILSYDALEGG------
SeaUrchin4.6.ye VPNPP------TNLRT--TMVAAQSITVSWDPPSEG---GSDTYNITYNVTGGQ------
SeaUrchin4.5.ye TTSAQT-----LSLAV--TPEDNTEFTVSWEDLDTE---AFEQYCVMYEPYEAQENR---
SeaUrchin4.4.ye DPAAI------AEIRIMDGSLGPRAVMLEWDQPEG----IRGLYEVTLDPADEGSV-DVD
SeaUrchin4.3.bl LPDPP------QNLVI--SEIGEDTATLSWADPAEM---NFDILEVVMTTMP--------
SeaUrchin4.2.ye KPASP------SDISV--SNNELTRVTVTWQPSEG----VVARYSYTYGLPGST------
SeaUrchin4.1.or MPSAV------GGLTI--TKTGIVDAEVSWSSPLLPN-GVIANYSLTIHISHDN------


                                                B              C
d1fnf_4          VSDVPR-----DLE-----VVAATPTSLLISWD-APAVTVRYYRITYGET-GGNSPV---
acorworm.6.yell -----RVA--FNIS-----AINVTTSSFTIRWD-EAVGDFDGYYIDVSPALTGLSSS---
acorworm.5.yell ---FPEKV--DSLR-----VSMVTETEIHVVWD-RPDGVVSTYVISIQPS-EGTATL---
acorworm.4.yell -GTIPP-----VLE-----VTSFTMDTITVATYVDNRLSHGMLHLSYFPT-DAEPIP---
```

```
acorworm.3.yell EPSPPG-----SIV-----VSRITEFGAIVNWE-PSSGDFNFYLVTYSSA-SVNTAL---
acorworm.2.yell LLAPPG-----RLS-----VNGVTSSSVQISWLPAISGLAVEYYVTYAPFVEGATPA---
acorworm.1.oran ---LPSSVKGFNVT-----VESPYQVTLTIQQPSFANGILESYIVNVVGYKEGFPPHIL-

acornworm1.1.wh ---FPA-----NVN-----VIDYGTDYITVNWT-HSGEHVDEFVITAIAT-ENEAANGT-
acornworm1.16.y IPEPPY-----NVT-----VESYNTDSIHLRWEPPLSNVFTHYNVTYVAD-SDPGMT---
acornworm1.15.y ---EPN-----SVV-LARDETQITTDTIAVTWSSYSGDIVDTYTVQCSSN-GTAEEA---
acornworm1.14.y ---KPN-----SVV-LARNDSQITNDTIAVIWNSNAGDEVSYYTVECSDG-GTPGQA---
acornworm1.13.y ---KPK-----SVT-LARDETQITNDTIAVTWSSYSGDIVNTYTVQCSSG-GTAEEA---
acornworm1.12.y ---KPN-----SVT-LARDDSQITTDTIAVTWSSNSGDIVDTYTVQCSSG-GTAEEA---
acornworm1.11.y ---IPN-----QAD-PVRNDNLVTNTTIGVKWT-DPGGNINHYEIGCPDG-GTPSAN---
acornworm1.10.y ---KPN-----KVR-LERDDDNITNDTIAVTWNSNSGDEVTYYTVECSSG-GTAEDS---
acornworm1.9.ye ---ESN-----AIT-LTKDSEYVTTTSIAVTWT-NPGGAVAYYTVECSEG-GTAENG---
acornworm1.8.ye ---EPLPV--TDLT-----KVDATTAFIEFSWIEPESGTWSGYDVGYLSE-DGTQQF---
acornworm1.7.ye ---IPN-----AVTDLVLTTPNQTNPEIVIMWS-PVEDEVSHYRVKCNDS-DDVTTE---
acornworm1.6.ye ---HPKPV--TSLS-LT--SPDNSVDTIHVSWTAPEDSDVDGYRVVCTET-DSGDKI---
acornWorm1.5.ye ---YPKPV--TSLS-LT--SPDNSVDTIHVSWTAPEDSDVDGYRVVCTET-DSGDKI---
acornWorm1.4.ye ---YPN-----SVT-IT--STSTTDSLNVTWS-LPDGNWTGYRIEYVPQ-DNLANAVLR
acornWorm1.3.ye ---VPATA--YNVTEV---ESSKTSYSLEFSWKINTTSLHDYYEVYYRGQLDTSFRN---
acornWorm1.2.ye TPLSPG-----AVS-----VIDVTETEIKIQWAQACENCTYDYYVHHEPE-DGEVDV---
acornWorm1.1.ye LPDTPE-----NIT------IETGMRSLNVTWD-EPEGEYAGYNISIEPS-TNRAGK---
                                                          :

d1fnf_4         VSDVPRDLEVV-AATPTS--LLISWD-A--PAVTVRYYRITYGET---GGNS-PVQEFTV
nematode1.3     -PSPVKAVNIN-QNSGSC--VEVTWQTD--EFSGADFYTIQYALQ---SNPS-NSTNMTI
nematode1.2     -SSPAVNVSLE-SVTRSSATLRIVFATHH-DSTSISNCQMHIVVRDM-NGKSVFDKRMQL
nematode1.1     -PAAVQNLKVE-PLNSYS--VMLTWLPPAMPNGILTHYNVNVTKM----GSD-ETRTIDV

nematode2.3     -PSPLKEVNIN-QNAGSC--VEVSWQND--EFSGADFYTIQYSLQ---STPN-NSTNMTI
nematode2.2     -SSPAVNVSLE-SVTRSSATLRIVFSTHH-DSTSISNCQMHIVVRDM-NGKSVFDKRMQL
nematode2.1     -PAAVQDLKVE-PLNSYS--VMLTWLPPALPNGILTHYAVNVTKI----GSD-ETRTIDV

nematode3.4     PRVPEFTTQNS-DITMHN--ITLRAVKN--EKFLEDSFLIEYRQLEPEQGYP-VLEVLDI
nematode3.3     -PGAVSDLIVS-E-NSSC--LTVEWDVP--PNSGADSFLLRYRQL---NAAV--NLSVTI
nematode3.2     -PASPTDFQVSPDITKGK--YRLTFDLP--PNSNYDGCHVSVVSETL-EALE-DDGEVNE
nematode3.1     -PGPVLSLSAR-PLNPYS--AQLMWLPPALPNGILTHYVVDVKSE---DDPN-DSRSLN


d1fnf_4         VSDVPRDL-----EVVAATPTS-LLISWD-APA----VTVRYYRITYGETGGN-------
flyPTP52F.3.whi QPSVIQD------PVVEAEAYS-MEVSWK-TPEY-ADLCIDGYRLSGWMEDDK-------
flyPTP52F.2.yel FPEQPESV-----MLEKSTVSS-LLFNWQ-PPTY-TNGPIKYYQAFLMRHEASYFVPADC
flyPTP52F.1.yel -QGPV--------NNNAVYEAN-VTITWK-VPCK-SNGDIEYFQLAFNGTRNNFA-----

flyPTP10D.10.ye APDPPSNL-----SVQVRSGKN-AIILWS-PPT---QGSYTAFKIKVLGLSEASS-----
flyPTP10D.9.whi KPNTPGKF-----IVWFRNETT-LLVLWQ-PPYP--AGIYTHYKVSIEPPDANDSVL---
flyPTP10D.8.whi -PLRPLNVTF---DRDFITSNS-FRVLWE-APKG--ISEFDKYQVSVATTRRQ-------
flyPTP10D.7.whi -PLPVRNLR----SINDDKTNT-MIITWE-ADP---ASTQDEYRIVYHELETF-------
flyPTP10D.6.whi ---PSSPII----EDLKSIRMG-LNISWK-SDV---NSKQEQYEVLYSRNGT--------
flyPTP10D.1-5.w YPNPPRNM-----TIETVRSNS-VLVHWS-PPE---SGEFTEYSIRYRTDSEQ-------
flyPTP10D.3.whi -PLIQSDVVVAN-GEKEDERDT-ITLSYTPTPQS--SSKFDIYRFSLGDAEIR-------
flyPTP10D.2.whi -PEPITQL-----HATNITDTE-ISLRWD-LPK----GEYNDFDIAYLTADN--------
flyPTP10D.1.whi -PGRVERF-----HPTDVQPSE-INFEWS-LPSSEANGVIRQFSIAYTNINNL-------

flyPTP4e.10.yel APDPPANL-----SVQLRSSKS-AFITWR-PPG---SGRYSGFRIRVLGLTDL-------
flyPTP4e.9.whit KPNTPGKF-----IVWFRNETT-LLVLWQ-PPFP--AGIYTHYRVSITPDDAIQSVL---
flyPTP4e.8.whit -PERVLNVTF---DEAYTTSSS-FRVRWE-PPRT--YSEFDAYQVMLSTSRRI-------
flyPTP4e.7.whit RPRPVRSLG----GFLDDRSNA-LHISWE-PAE---TGRQDSYRISYHEQTNASEVPA--
flyPTP4e.6.whit ---PAAPLI----QELRSIDQG-LMLSWR-SDV---NSRQDRYEVHYQRNGT--------
flyPTP4e.5.whit FPKPPQNL-----TLQTVHTNL-VVLHWQ-APE---GSDFSEYVVRYRTDASP-------
flyPTP4e.4.whit -PQPVSNV------VPLVDSRN-LTLEWP-RPD----GHVDFYTLKWWPTDEEDRVE---
flyPTP4e.3.whit -----SDVFIANAGHEQGQDET-ITLSYTPTPAD--STRFDIYRFSMGDPTIK-------
flyPTP4e.2.whit -PLPISDL-----KAIQVAARE-ITLHWT-APA----GEYTDFELQYLSADEE-------
flyPTP4e.1.whit -PGKVDYF-----QPSDVQPGE-VTFEWSLEPAE-QHGPIDYFRITCQNADDA-------


                                        B                C
d1fnf_4         -VSDVPRDLEV--VAATPTS-LLISWDAPA---VTVRYYRITYGETGGNS---PVQEFTV
annelid.14.yelo -IPGKPINFRTVDNSVGATF-FTLTWESAAG--SQQDDFVVVYRKIGSSD-----VVQVF
```

```
annelid.13.whit -RPPTPRIHNI--TAVDTKS-VTVQYSAGAG--GSQNSFVVTYVNQEGQTN--EPVCSYS
annelid.12.whit --PLPPSAPKL--FERSETT-LTVIGDVPSTNSTVFDGCKASVTPAIQNG-----PYVFK
annelid.11.blue -MPGVISRHTG--IATTTKQ-LAVSWGAPNS--GQVGGYVAKLYEHGKSV---AIESKTL
annelid.1o.yelo -----------------ST-LVVQWDEPST--GFYDEFLVRIQSHGETE-----KITKS
annelid.9.yelow -KPERPSNLKK--ESTTSDS-ITVKWNAPVS--ANFDSYRITINSTDHEN-------EVL
annelid.8.yelow -NPAAPTDLRI--LSRNTNM-MSFTWSPPTNP-GTIDDYKYSITISGAAT---PEIERSG
annelid.7.blue  -GPSSVADLLV--TSYNDDS-ITVEWEKPTG--TTVNGFTISIDPPPSGQ----SGSKDL
annelid.6.yelow -KPKRALSLSA--NYVSTDS-ISLSWKVDQS--SSQDNFKITYEVTGSSTEWTRETTFTQ
annelid.5.yelow IQPKSVRELK---AEVIPSG-ISLSWLPGFD--STQNSYRYQYQGKNVKL----NIVPWV
annelid.4.yelow -YPLPPTDLTVDRSATTVSS-VRVQWKDDVTR-SYITSWDIKIADRGTDNI--RRVGSTH
annelid.3.yelow -KPVIRSTLSEDTEKTTEDT-IAVTYSESKA--GVFDHYLFSLLNSSDTVI------KQR
annelid.2.yelow -KPNKPKH----KCNQGTDK-LTIILIKPS---GFVDEYILKCLNEDCNS-------TEI
annelid.1.yelow -PPGPVQEFSYSENSLKPFE-IRLTWMEPRNPNGKIIKYHIKYNGIKENQVPHTGSEVDV
                         .

                                        B              C
d1fnf_4         -VSDVPRDLEV-VAATPTS-LLISWDAP-----AVTVRYYRITYGETGGNS---------
sponge.9.yellow -MSSQVNDTNVDIDRDNMR-LNISWAPP--SRPAGNILRYEVTLSIRGKIC------LTI
sponge8.white   -APTPPLNFTL-TELSLSS-LKASWEEP--STLNGVLANYTVYCNLSSLQFYSQQLMLSF
sponge7.white   --AGPPEGFNV-TDVTATS-VSLEWRRP--SVPNGVILHYFLQYSAYNVTI---------
sponge.6.orange ---VPGTNLDG-FNTSSMS-LRITWTELLEDDQNGVIIGYNISYFSLPAVGQ--------
sponge.5.yellow -VPTSPSDVSY-INISSTS-IEVSWNPP--TDFNGPNEGYVITYIRLESDTESM------
sponge.4.yellow -LPGPPSNVS--TMSTNTS-ISITWSPP--LDPNGLLLSYSINVTLNSTYAQ----YLSF
sponge.3.blue   APAAPVANPMA-SPVSSTA-VNVSWLPPNLSNWNGLITNYTIEYRTNDEYIR--------
sponge.2.blue   TPSGPPLDVSV-IVDSPST-ARISWSPPMYIDRNGIIVNYTVRIITTVRGT---------
sponge.1.orange -VPSAPDPVIV-KRRNDTA-IQVNWTRP--AEPNGIILGYLIYYIGTKNNTGTE--YSNI
```
