# Supplementary material for: Phosphotyrosine phosphatase R3 receptors: Origin, evolution and structural diversification
Source: PLoS One. 2017 Mar 3;12(3):e0172887. doi: 10.1371/journal.pone.0172887 (PMC5336234; doi:10.1371/journal.pone.0172887)
Supplement: S3 Fig — Alignment of the FN3 domains from Fig 4, panel E with introns inside the FN3 domain and represented by orange, blue or black squares. Fibronectin FN3 10th repeat is depicted in the top of the alignment and the A-F β-strands are highlighted in magenta. Highly conserved FN3 residues (W, Y, L, Y) are in blue bold letter. Introns in phase 2 are represented by red bold letters and highlighted in yellow; introns in phase 1 are represented by red bold letters and highlighted in grey; introns in phase 0 are indicated by highlighting the flanking amino acids in green. Chicken (Gg) and Xenopus (Xt) FN3 domains were omitted whentheir intron positions were identical to that of human (Hs) and zebrafish (Dr). FN3 domains are numbered starting from the one closest to the transmembrane domain as in Fig 4, panel D. (PDF) [file pone.0172887.s003.pdf]

```
                                   A          B                    C                        D           E          F
d1fnf_4          VSDVPRDLEVVAA--TPT-SLLISWDAPA-------VTVRYYRITYGETG--------GNSPVQEFTVP---GSKSTATISGLKPGVDYTITVYA-VT

Hs.Q.9.black     PDSPPQDFSVKQL--SGV-TVKLSWQPPL----EPNGIILYYTVYVWNRS--------SLKTINV--------TETSLELSDLDYNVEYSAYVTA-ST
Mm.Q.9.black     PDSPPQNFSVKQL--SGV-TVMLSWQPPL----EPNGIILYYTVYVWDKV--------SLKTINA--------TEVSLELSDLDYHADYSAYVTA-ST
Gg.Q.9.black     PSSPPESLSVKQL--SGV-TVKLSWKPPL----EPNGIILYYTVYVWNKT--------SKRSVNV--------TETSLEFTDLENNSEYSAYVAA-ST
Dr.Q.9.black     PSSPPKFLIARKL--SDT-EVELSWEAPE----EANSEILYYIVRVWNLS--------TEFVANV--------TETSVVVS-VDGPGQYNASVSS-WT

ciona.8.black    PESAPYDIIFQQY--NST-TIALTWRPPV----KPNGIIVNYTVYYSNED--------KVMTKTT--------TKPRTVLRNLEKFTEYEVYLTA-ST
ciona2.8.black   PSSPPDGIFFHQH--NST-SISLTWNPPL----TPNGIITLYSVHYRHGNK--------SLIRT--------TATPGITLNNLKKFTSYDVYIRA-ST

Hs.Q.10.blue     PDSAPENITYKNI--SSG-EIELSFLPPS----SPNGIIQKYTIYLKRSN--------GNEERTIN------TTSLTQNIKVLKKYTQYIIEVSA-ST
Mm.Q.10.blue     PDSAPENITYKNI--SSE-EIEIFFLPPR----SPNGIIQKYTIYLKRSN--------SHEARTIE------TTSLTLTIGGLKKYTHYVIEVSA-ST
Gg.Q.10.blue     PNSAPENITYWNI--SST-EIELSFFPPS----IPNGIIQTYTIYLKRIN--------GTEERVIN------TTHLVLRITDLKKYTEYMIEVSA-ST
Hs.Q.11.blue     VPSSIKIINYKNI--SSS-SILLYWDPPE----YPNGKITHYTIYAMELD--------TNRAFQIT------TIDNSFLITGLKKYTKYKMRVAA-ST
Mm.Q.12.blue     VPSSIQIINYKNI--SSS-SILLYWDPPE----YPNGKITHYTIYAMELD--------TNRAFQMT------TVDNSFLITGLKKYTRYKMRVAA-ST
Gg.Q.12.blue     VPSSVQSISYKNI--SSS-SVLLYWDPPA----NPNGKIIHYTVYAMELD--------TKRAFHTT------TSNNSLLMTGLKKYTNYKMRVAA-ST
Dr.Q.12.blue     VPSSVQNVSYQNL--SST-SIRVSWEPPL----NPNGKITHYAVYAQNLL--------TNQELRQM------TDTTTAVLTGLDKYSSYKVRVAA-ST
Hs.Q.14.blue     PEGPPQNCVTGNI--TGK-SFSILWDPPT----IVTGKFS-YRVELYGPS--------GRILDNS-------TKDLKFAFTNLTPFTMYDVYIAA-ET
Mm.Q.14.blue     PEGPPQNCITGNV--TGK-AFSISWDPPA----IVTGKFS-YRVELYGPT--------GRILDNS-------TKDLRFVFTHLTPFTMYDVYVAA-ET
Gg.Q.14.blue     PEDPPQNFAKVNI--TSK-SFSVMWDPPT----IVTGKFS-YRVELYGPS--------GHILDNS-------TKDLKFVFHNLVPFTMYDVYVGA-ET
Dr.Q.14.Blue     PEDPPQNVVLRNI--TSK-SVSLTWEPPK----IITGRFS-YVIQLHSSE--------GLISENS-------TIDQMFIYTGLTPYTTYYIHVMA-KS
Gg.Q.20.blue     ---VPSAVKYLNYSRDSE-SITVTWPPAQ-------NKFDGYVLSIKSKI-----------FNKENMLS---SGVRMYKAECLLPGTDFLISIVT-TS

ciona.10.blue    PATPPFNVSYQNL--TST-KVRLTWRRPL----VPNGIIQFYEISLTSKN--------NKTIRATTE-----NDVTAVTVDHLTAYSEYTATVRA-NT
ciona2.3.blue    ----LNNVEIFNL-QVPTPVYIFTWDLPL----LSNGRVIAYKLSHSVLN------D-TQTYFVK-------PPQINQTLQHLQPFTEYFISVNA-KT
seaUrchin3.25.b  VSLDALDLETYSV--TET-TIGVAFGPAL-------VAPSGYTLSLTSDG-----------ITIGLIII-PVAGSTKFVFTGLTSGTLYRAQVQV-TG
SeaUrchin4.3.bl  LPDPPQNLVISEI--GED-TATLSWADPA----EMNFDILEVVMTTMPAT-------------KTTILK---LNTNTVELTNLVPGQTYTVQLAS-VI
SeaUrchin4.7.bl  VVGVLGSLNITAF--DET-TISIEWEQVD---------VEEYILSYDALE--------GGSGNVTVQS----TDPNSYTFTGLTPGTQYTIGVQP---
annelid.7.blue   GPSSVADLLVTSY--NDD-SITVEWEKPT------GTTVNGFTISIDPPP--------SGQSGSKDLT----ENIQEYTWTGLSAGREYNLIIEV-IF
annelid.11.blue  QPGVISRHTGIAT--TTK-QLAVSWGAPN------SGQVGGYVAKLYEHG--------KSVAIESKTLT---TNMRQATFHNLTPGQVYDVSVSS-YV
sponge.2.blue    PSGPPLDVSVIVD--SPS-TARISWSPPMY--IDRNGIIVNYTVRIITTV--------RGTIRETNISN---VSGNYYDASDLPQFASFNVTVAA-AT
sponge.3.blue    PAAPVANPMASPV--SST-AVNVSWLPPNL--SNWNGLITNYTIEYRTND------E-YIRPSIEV(17)TPLQLESIIIPSLHEFVNYSFIITL-SN

Hs.O.1.orange    EPAPPKSLFAVNK--TQT-SVTLLWVE--------EGVADFFEVFCQQVG------S-SQKTKLQEPVA---VSSHVVTISSLLPATAYNCSVTS-FS
Mm.O.1.orange    EPAPPKSLFAVNK--TQT-SVTLLWVE--------EGVADFFEVFCQQLG------S-GHNGKLQEPVA---VSSHVVTISSLLPATAYNCSVTS-FS
Gg.O.1.orange    EPAPPKSLFAVNK--TQT-SVTLLWVE--------EGVADFFEVYCQQAG------S-NQEVKVQEPVT---VSSHVVTISSLTPATSYNCSVTT-FS
Dr.O.1.orange    DPAPPRSLYAVNA--SDT-SVTLIWAE--------EGVVDHYLITCRALG------AHAEQKKVREPLV---TSAHVLTVSGLQASTTYNCSVRS-SS
Hs.O.2.orange    ---APVAPEITSVEYFNS-LLYISWTYGDDTTDLSHSRMLHWMVVAEGKK--------KIKKSV---------TRNVMTAILSLPPGDIYNLSVTA-CT
Dr.O.2.orange    ---APEAPHISSVEFSHG-SVFVRWTYGDLFTDLTHSRMLHWQVVAEGKK--------SARRFSVDV----TRSVMKASLALPAGDIYNLTVTA-CT
Hs.O.3.orange    VPTGIKDLMLYPL--GPT-AVVLSWTRPY------LGVFRKYVVEMFYFN--------PATMTSEWTTY(7)SLTASVRIANLLPAWYYNFRVTM-VT
Dr.O.3.orange    EPTGVRDLVVYPL--SPS-AVILSWQRPY------NVAFRKYVLQTFFFN--------SATQTAQWSTY(7)SVIASVRVTDLLPAWFYNFRVTM-VT
Hs.O.4.orange    LTEKPQHVSVHVL--SST-TALMSWTSSQ-------ENYNSTIVSVVSLTC--QKQKESQRLEKQYCTQV---NSSKPIIENLVPGAQYQVVIYL-RK
Dr.O.4.orange    PQERPQAVSVKML--DSS-TAAVSWAPST-------HTYNGSLISVQSLTC--LRPSISQRMELNYCSEE---NITSDIISSLTPGAQYRVVVYH-TN

Hs.Q.1.orange    PKDPPNNMTFQKIPDEVT-KFQLTFLPPS----QPNGNIQVYQALVYBLACK------D-PTAVQIHNLSI(4)NTFVIAMLEGLKGGHTYNISVYA-VN
Dr.Q.1.orange    PKDPPKNVTLTVIPEEVT-RVYVTFSPPD----EPNGNISAYRVDIYRNG--------QLDFFINSLSV(4)NNTMTAIIDGLKGGFNYSIRIAA-VN
Hs.Q.2.orange    PDGPPENVHVVAT--SPF-SISISWSEPA----VITGP-TCYLIDVKSVD--------NDEFNISFIKSN--EENKTIEIKDLEIFTRYSVVITA-FT
Dr.Q.2.orange    PDAPPGAISVVP---SAN-GLKIEWDKPS----VISGP-TSYIIDITALD--------GSGYNITLVRHS--EEIRTVIVGNLSAFTLHSVTITA-FT
Hs.Q.5.orange    PLAPPQNLTLINC--TSD-FVWLKWSPSP----LPGGIVKVYSFKIHEHE--------TDTIYYKNIS----GFKTEAKLVGLEPVSTYSIRVSA-FT
Dr.Q.5.orange    PMSPPRNLTIFNH--TAN-SVWLQWEPSP----EPNGVVQLYGFRILELN--------TDSFRYQNSS----DASTQAELGGFKPHNSYEISVCT-FT
Hs.Q.8.orange    PSDPPKDVYYANL--SSS-SIILFWTPPS----KPNGIIQYYSVYYRNTS--------GTFMQNFTLHE—(7)MTVSTIIDKLTIFSYYTFWLTA-ST
Dr.Q.8.orange    PSDPPQDVVYTLL--TIS-TVRLSWRPPN----EPNGIIQYYTIYYTDNN--------TEYTERVP------GSEHQLLLSDLQAGQDYSVWMSS-ST
Hs.Q.16.orange   KPGPPVFLAGERV--GSA-GILLSWNTPP----NPNGRIISYIVKYKEVC------PWMQTVYTQVRSK---PDSLEVLLTNLNPGTTYEIKVAA-EN
Dr.Q.16.orange   --SAPSKPEGERV--GST-GILLSWRMPM----PLDPSIHSFVIRYKTKS------PYPDPSFTEITKS---LDIPETLLNTLTPGATYNIKVAA-VN
Gg.Q.17.orange   EPAKPEGLKFFNV--SSN-SFSLYWRLPY-------GHVDRFCVDLIPDH--------G-SVVISDLG----VREYQADFYNTTPGTTYNVTVST-VS
Dr.Q.17.orange   VPASPEGLRVISV--SPR-SFSLHWLASP-------GCEKTYQVQLYPDH--------GNINITTDTA----DNNVQAQVSSVTPGTSYTVTVNA-VA
Gg.Q.19.orange   CPDPPSDLQVLGQ--EEN-TVYLSWKLPR-------GGFDKFQLSYCLMN--------NEKPFTRT------VYDSRTVVKNLAPGMEYTFQLRT-IK
Gg.Q.21.orange   CLAAPLNIREGNV--TDT-SVQIAWDRAE-------GDFQQYEVTCTNCA--------SAFRVQK-------VKQETATFSNLVPGKLYSFTVRT-EK

ciona.1.orange   PSDFPESVGVATVLNNSS-SVHVLFKEPL----DPNGKLINYTIQHRRLED--------SKLQSIVVSV(9)HEPYSVSVSGLLGGRYYQFRVRA-AT
ciona.2.orange   PDSPVRDVHVTVV--SST-SINVTWNLPS----SYAGP-TTYKL-------------------------------YSELVPLYDYFDHTAQ---
ciona.3.orange   YADAPPQLQLKNL--SDR-SITIEWSRPL----QWNGRFHGYLITYKPPD--------SCPNPANDTQV(4)SNLTSVTLEALSKYRAYNISVGS-IT
ciona.5.orange   -QDAVTDIQLINL--TSD-SALIKWLAPR----QPNGVITHYTVHYGRNS--------TIQ-----------TNVTEATLRDLHPFQLYWIEVYP-WT
ciona.6.orange   PDNGVSDLSALAI--NAT-TIKVTWQPGI----PLTGP-TFFHIQYVLNN--------------------TPVHDLNFISFQPHTEYVIKVYPRVV
ciona.15.orange  EPGTPTALVLKPV--STT-ELLTEWEIPL----NPNGIIRRYIIRFKQNY--------PHPSTNYTLIE---TNQTRYLLGNLEPGAEYVALFAA-VN
ciona2.1.orange  PSDFPRDVNVAIVMGNSS-SVRVLFKPPR----DPNGVLTNYTIQFKRLEN--------SESRNAIIP(10)NVPYYVTINGLLGGRLYKFRVRA-AT
ciona2.2.orange  PEHSVENLQAIVT--SST-TVNVTWESPE----SFAGP-TTYKVEAFHSTT--------MQRVTSPLL---TSSTHSYVMSGLDEDSIYGVTVST-MT
ciona2.7.orange  PADPPHDVRVAAI--SST-SINVTWSPPT----TPNGLIQFYTVYYQHNPS--------TVQTKNV------TKGMQVVIGNLRKFTNYLVWVTS-ST
ciona1.1.orange  PGPQPDLSRNDSCTNLMT-ECVAVWYTPA-(7)TSSRKKRSYDNPIGNNLI----------TFVIGSGESTISPWKTLFVNLPLAVDTPYVVAVAAKTS
ciona3.6.orange  KPEAVTRLLKD---SSTTESISITWTLPT----AGLFDKQIVEVNKVNTPD-------------TNVT-EINDNSTTTFNITGLTSGASYNISVITVSS
ciona3.2.orange  PDSVKNLKVLKSS-TKPTTELDLTWDQPA---GDGENITIMYTQNPGGLVT-------------------NKIASFTDTTKQLTVEPGYNYTVALTVN
seaUrchin3.20.o  LPSAVSDVDVVP---EAA-ALTLSWDPVF-------GDVDGYHASYQLKE--------NPYQISDLFYVSD-PSNPTIRLTGLSPLSEYIITLTS-FS
seaUrchin3.26.o  IPFVVRNLALTGEP-TAI-SFSISWNQPA------SGMYDGYAVYIAGQD--------NVERLTSRLDD---PDTTELLIEDLDPNQEYTVSVYA-VA
seaUrchin3.33.o  GVFDQLELSVTTV--SNT-SIALVWGIYED--RDNLPELTNYELSITPPD------A------ISLIRG---LDVFRATFERLIPGQEYSIQLKL-TT
seaUrchin3.37.o  GALQEGVIEFRDK--TTT-LIEVLFGTAP-----LSSEVVQYRVTLTSEE--------GASTSFNVAPTS--EPFSVVTFDGLTPGRLYTVSLQA-F-
SeaUrchin4.1.or  MPSAVGGLTITKT--GIV-DAEVSWSSPL----LPNGVIANYSLTIHISHDNGTLSPYGDTVTISAVAE---QTEYSQDVIGLAAGQYYVFSVYA-TN
acorworm.1.oran  LPSSVKGFNVTVE--SPY-QVTLTIQQPS----FANGILESYIVNVVGYKEG--FPPHILDFEIPPDET---DDITEYLLEDVIAGYTYTFTVKP-QN
sponge.1.orange  VPSAPDPVIVKRR--NDT-AIQVNWTRPA----EPNGIILGYLIYYIGTK--------NNTGTEYSNIN(10)NTLSYLITNLLADTQYFINVTA-YT
sponge.6.orange  ---PGTNLDGFNT--SSM-SLRITWTELLE--DDQNGVIIGYNISYFSLP--------AVGQPINNF-----TSDTSYNISGLDVYTDYNVSVAA-YT
```
