# Supplementary figures and images for: Phosphotyrosine phosphatase R3 receptors: Origin, evolution and structural diversification
Source: PLoS One. 2017 Mar 3;12(3):e0172887. doi: 10.1371/journal.pone.0172887 (PMC5336234; doi:10.1371/journal.pone.0172887)

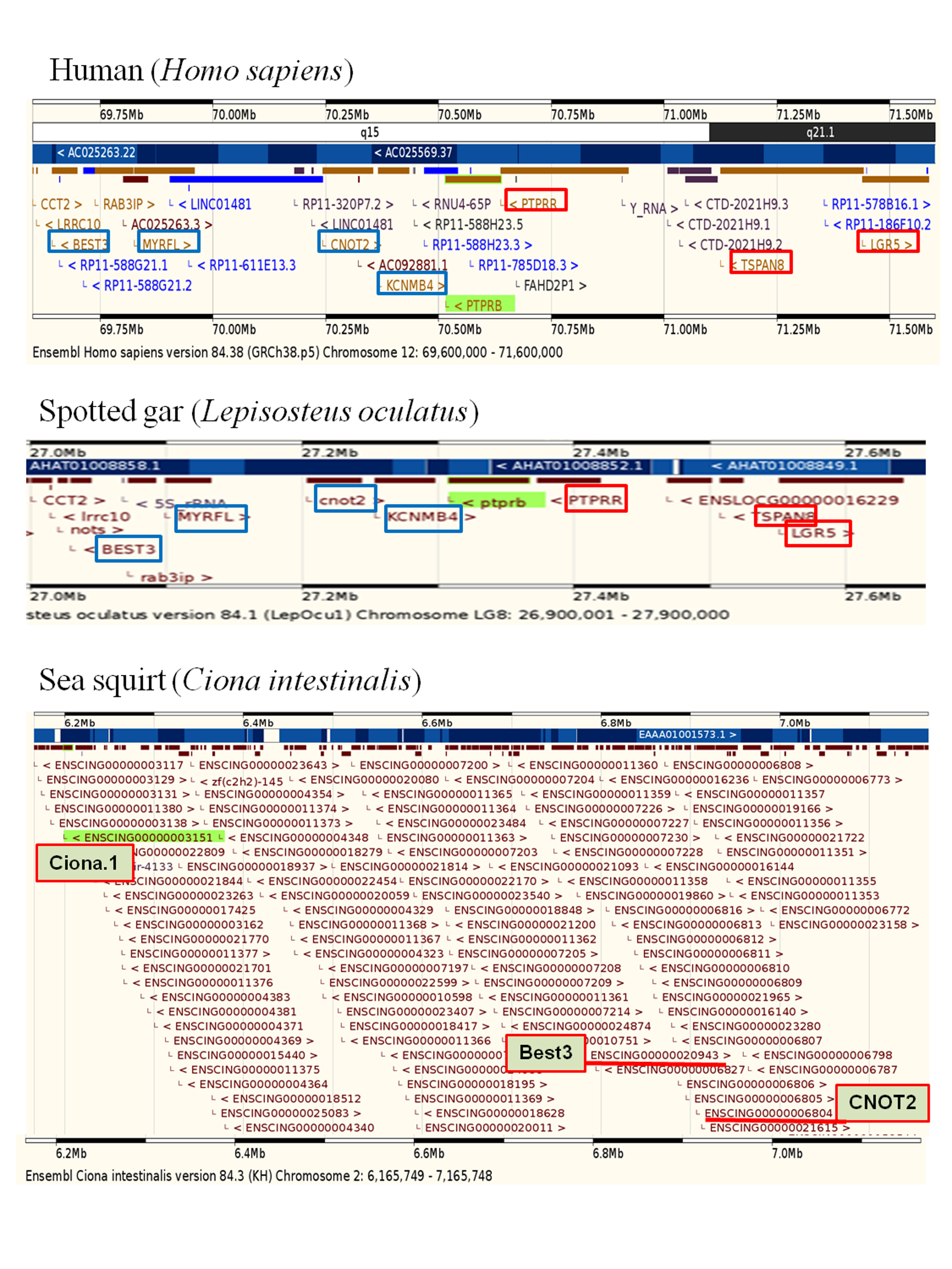

Supplement: S4 Fig — Caption of the ENSEMBL genomic region of PTPRB in human and spotted gar and of Ciona_1 in the sea squirt C. intetinalis genome. (TIF) [file pone.0172887.s004.tif]
