# Supplementary material for: Phosphotyrosine phosphatase R3 receptors: Origin, evolution and structural diversification
Source: PLoS One. 2017 Mar 3;12(3):e0172887. doi: 10.1371/journal.pone.0172887 (PMC5336234; doi:10.1371/journal.pone.0172887)
Supplement: S1 File — Transmembrane segments are highlighted in pink, and PTP catalytic domains are underlined. Exons are displayed in alternate colors. Amino acids in bold red colour indicate that they are split between adjacent exons by a phase 1 or 2 intron. FN3 domains are shaded in the same colors as in Fig 3 and are numbered in parenthesis. The cysteine residues of the long juxtamembrane FN3 domain are highlighted in yellow. Tyrosine phosphorylation predicted residues in the carboxy terminus are highlighted in red and the asparagines of the YxN motif in blue. (PDF) [file pone.0172887.s005.pdf]

Protein sequences

VERTEBRATES

PTPRQ

>PTPRQ.human gi|222537743|ref|NP_001138498.1| phosphatidylinositol phosphatase PTPRQ precursor [Homo sapiens]
MDFLIIFLLLFIGTSETQVDVSNVVPGTRYDITISSISTTYTSPVTRIVTTNVTKPGPPVFLAGERVGSAGILLSWNTPPNPNG
RIISYIVKYKEVCPWMQTVYTQVRSKPDSLEVLLTNLNPGTTYEIKVAAENSAGIGVFSDPFLFQTAESA(15)PGKVVNLTVE
AYNASAVKLIWYLPRQPNGKITSFKISVKHARSGIVVKDVSIRVEDILTGKLPECNENSESFLWSTASPSPTLGRVTPPSRTTH
SSSTLTQNEISSVWKEPISFVVTHLRPYTTYLFEVSAATTEAGYIDSTIVRTPESV(14)PEGPPQNCVTGNITGKSFSILWDP
PTIVTGKFSYRVELYGPSGRILDNSTKDLKFAFTNLTPFTMYDVYIAAETSAGTGPKSNISVFTPPDV(13)PGAVFDLQLAEV
ESTQVRITWKKPRQPNGIINQYRVKVLVPETGIILENTLLTGNNEYINDPMAPEIVNIVEPMVGLYEGSAEMSSDLHSLATFIY
NSHPDKNFPARNRAEDQTSPVVTTRNQYITDIAAEQLSYVIRRLVPFTEHMISVSAFTIMGEGPPTVLSVRTRQQVPSSIKIIN
YKNISSSSILLYWDPPEYPNGKITHYTIYAMELDTNRAFQITTIDNSFLITGLKKYTKYKMRVAASTHVGESSLSEENDIFVRT
SEDE(12)PESSPQDVEVIDVTADEIRLKWSPPEKPNGIIIAYEVLYKNIDTLYMKNTSTTDIILRNLRPHTLYNISVRSYTRF
GHGNQVSSLLSVRTSETV(11)PDSAPENITYKNISSGEIELSFLPPSSPNGIIQKYTIYLKRSNGNEERTINTTSLTQNIKVL
KKYTQYIIEVSASTLKGEGVRSAPISILTEEDA(10)PDSPPQDFSVKQLSGVTVKLSWQPPLEPNGIILYYTVYVWNRSSLKT
INVTETSLELSDLDYNVEYSAYVTASTRFGDGKTRSNIISFQTPEGA(9)PSDPPKDVYYANLSSSSIILFWTPPSKPNGIIQY
YSVYYRNTSGTFMQNFTLHEVTNDFDNMTVSTIIDKLTIFSYYTFWLTASTSVGNGNKSSDIIEVYTDQDI(8)PEGFVGNLTY
ESISSTAINVSWVPPAQPNGLVFYYVSLILQQTPRHVRPPLVTYERSIYFDNLEKYTDYILKITPSTEKGFSDTYTAQLYIKTE
EDV(7)PETSPIINTFKNLSSTSVLLSWDPPVKPNGAIISYDLTLQGPNENYSFITSDNYIILEELSPFTLYSFFAAARTRKGL
GPSSILFFYTDESV(6)PLAPPQNLTLINCTSDFVWLKWSPSPLPGGIVKVYSFKIHEHETDTIYYKNISGFKTEAKLVGLEPV
STYSIRVSAFTKVGNGNQFSNVVKFTTQESV(5)PDVVQNMQCMATSWQSVLVKWDPPKKANGIITQYMVTVERNSTKVSPQDH
MYTFIKLLANTSYVFKVRASTSAGEGDESTCHVSTLPETV(4)PSVPTNIAFSDVQSTSATLTWIRPDTILGYFQNYKITTQLR
AQKCKEWESEECVEYQKIQYLYEAHLTEETVYGLKKFRWYRFQVAASTNAGYGNASNWISTKTLPGP(3)PDGPPENVHVVATS
PFSISISWSEPAVITGPTCYLIDVKSVDNDEFNISFIKSNEENKTIEIKDLEIFTRYSVVITAFTGNISAAYVEGKSSAEMIVT
TLESA(2)PKDPPNNMTFQKIPDEVTKFQLTFLPPSQPNGNIQVYQALVYREDDPTAVQIHNLSIIQKTNTFVIAMLEGLKGGH
TYNISVYAVNSAGAGPKVPMRITMDIKA(1)PARPKTKPTPIYDATGKLLVTSTTITIRMPICYYSDDHGPIKNVQVLVTETGA
QHDGNVTKWYDAYFNKARPYFTNEGFPNPPCTEGKTKFSGNEEIYIIGADNACMIPGNEDKICNGPLKPKKQYLFKFRATNIMG
QFTDSDYSDPVKTLGEGLSERTVEIILSVTLCILSIILLGTAIFAFARIRQKQKEGGTYSPQDAEIIDTKLKLDQLITVADLEL
KDERLTRLLSYRKSIKPISKKSFLQHVEELCTNNNLKFQEEFSELPKFLQDLSSTDADLPWNRAKNRFPNIKPYNNNRVKLIAD
ASVPGSDYINASYISGYLCPNEFIATQGPLPGTVGDFWRMVWETRAKTLVMLTQCFEKGRIRCHQYWPEDNKPVTVFGDIVITK
LMEDVQIDWTIRDLKIERHGDCMTVRQCNFTAWPEHGVPENSAPLIHFVKLVRASRAHDTTPMIVHCSAGVGRTGVFIALDHLT
QHINDHDFVDIYGLVAELRSERMCMVQNLAQYIFLHQCILDLLSNKGSNQPICFVNYSALQKMDSLDAMEGDVELEWEETTM

>PTPRQ.mouse gi|124487427|ref|NP_001074901.1| ENSMUST00000050702 phosphatidylinositol phosphatase PTPRQ precursor [Mus musculus]
MDFLFFFLFSLIGTSESQVDVSGSFDDTVYDITLSSISATTYSSPVSRTLATNVSKPGPPVFLAGERVGSAGILLSWNTPPNPN
GRIISYVVKYKEVCPWMQTAYTRVRAKPDSLEVLLTNLNPGTTYEIKVAAENSAGIGVFSDPFLFQTAESA(15)PGKVVNLTV
EALNYSAVNLIWYLPRQPNGKITSFKISVKHARSGIVVKDVSIKVEDLLSGKLPECNENSDSFLWSTTSPSPTLSRATPPLRTT
HLSNTLARNKISSVWKEPISFVVTHLRPYTTYLFEVSAVTTEAGYIDSTIVRTPESV(14)PEGPPQNCITGNVTGKAFSISWD
PPAIVTGKFSYRVELYGPTGRILDNSTKDLRFVFTHLTPFTMYDVYVAAETSAGVGPKSNLSVFTPPDV(13)PGAVFDLQIVE
VEATEIRVSWRKPRQPNGIISQYRVKVSVLESGVILENTLLTGQDEYINNPMTPEIMNLVDPMIGFYEGSGEMSSDLHSLASFI
YNSHPHDFPARTRVEDQRSPVVATRNQYMTDIAAEHLSYVIRRLVPFTEHTISVSAFTVMGEGPPTVLTVRTREQVPSSIQIIN
YKNISSSSILLYWDPPEYPNGKITHYTIYAMELDTNRAFQMTTVDNSFLITGLKKYTRYKMRVAASTHVGESSLSEENDLFVRT
PEDE(12)PESSPQDVKVTDVSPSELSLTWSPPEKPNGIIIAYEVFYQNADALFVKNTSTTNITLSDLKPYTLYNISIQSYTRL
GHGNQSSSLLSVRTSETV(11)PDSAPENITYKNISSEEIEIFFLPPRSPNGIIQKYTIYLKRSNSHEARTIETTSLTLTIGGL
KKYTHYVIEVSASTLKGEGVRSMPISILTEEDA(10)PDSPPQNFSVKQLSGVTVMLSWQPPLEPNGIILYYTVYVWDKVSLKT
INATEVSLELSDLDYHADYSAYVTASTRFGDGKTRSSVINFRTPEGE(9)PSDPPKDVHYVNLSSSSIILFWTPPVKPNGIIQY
YSVYYQNTSSTFVQNFTLLEVTQEPGNVTVSARIYKLAVFSYYTFWLTASTLVGNGNKSSDVIHVYTDQDI(8)PEGGVGNLTY
ESLSSTAINVSWTPPSQPNGLVFYYVSLNLQQSPPRHRRPPLTTYENSIYFDNLEKYTDYIFKITPSTEKGFSETYTAQLHIKT
EEDV(7)PDTPPIINTFKNLSSTSILLSWDPPLKPNGAILSYHLTLQGTHANRTFVTSGNHIVLEELSPFTLYSFLAAARTMKG
LGPSSILFFYTDESA(6)PLAPPQNLTLINYTSDFVWLTWSPSPLPGGIVKVYSFKIHEHETDTVFYKNISGFQTDAKLAGLEP
VSTYSISVSAFTKVGNGNQFSNVVKFTTQESV(5)PDAVQNIACVARDWQSVSVMWDPPRKANGIIIHYMITVEGNSTKVSPRD
PMYTFTKLLANTSYIFEVRASTSAGEGNESQCNVSTLPETV(4)PSVPTNTAFSNVQSTSVTLRWIKPDTILGYFQNYKITTQL
RAQKCREWEPEECVEHQEVQYLYEANQTEDTVRGLKKFQWYRFQVAASTNAGYGNASSWISTQTLPGP(3)PDGPPENVRVVAT
SPFGINISWNEPAIITGPTFYLIDVKSVDNDNFNISFVKSNEENKTTEINDLEVFTRYSVVITAFVGNVSGAYTDGKSSAEVII
TTLESV(2)PKDPPNNMTFQKIPDEVTKFQLSFLPPSQPNGNIQVYQALVYREDDPTAVQIHNLSIIQKTDTSVIAMLEGLKGG
HTYNISVYAINSAGAGPKVQMRITMDIKA(1)PARPKTKPIPIHDATGKLLVTSTTITIRMPICYYNDDHGPIRNVQVLVAEAG

AQQDGNVTKWYDAYFNKARPYFTNEGFPNPPCIEGKTKFSGNEEIYVIGADNACMIPGNEEKICNGPLKPKKQYLFKFRATNVM
GQFTDSEYSDPIKTLGEGLSERTVEIILSVTLCILSIILLGTAIFAFARIRQKQKEGGTYSPRDAEIIDTKFKLDQLITVADLE
LKDERLTRLLSYRKSIKPVSKKSFLQHVEELCTNNNLKFQEEFSELPKFLQDLSSTDADLPWNRAKNRFPNIKPYNNNRVKLIA
DVSIPGSDYINASYVSGYLCPNEFIATQGPLPGTVGDFWRMVWETRAKTLVMLTQCFEKGRIRCHQYWPEDNKPVTVFGDILIT
KLMEDIQIDWTIRDLKIERHGDCMTVRQCNFTGWPEHGVPENTTPLIHFVKLVRTSRAHDATPMVVHCSAGVGRTGVFIALDHL
TQHIHDHDFVDIYGLVAELRSERMCMVQNLAQYIFLHQCILDLLSNKGGHQPVCFVNYSTLQKMDSLDAMEGDVELEWEETTM

>PTPRQ.chicken XP_001235338.2| PREDICTED: phosphotidylinositol phosphatase PTPRQ
ENSGALT00000017794[Gallus gallus]

MPDTGVFDGIYVTTNGGPNATFSLKSDGKLTVENLTPGTEYDFCVFTKSREMLSSSYRVTGVKTCLAAPLNIREGNVTDTSVQI
AWDRAEGDFQQYEVTCTNCASAFRVQKVKQETATFSNLVPGKLYSFTVRTEKEGFRDSVLVAKEIETV(20)PSAVKYLNYSRD
SESITVTWPPAQNKFDGYVLSIKSKIFNKENMLSSGVRMYKAECLLPGTDFLISIVTTSGLKRSHPTFLKISTC(19)PDPPSD
LQVLGQEENTVYLSWKLPRGGFDKFQLSYCLMNNEKPFTRTVYDSRTVVKNLAPGMEYTFQLRTIKGWDSSVAVEKNVITK(18
)PAGICNLALKMVNTSSATLMWNPTKTNFTSYKASLSNTTFISKFMIPGAVSKFSVTNLTAGGIYNFTLQRLQGNIEGSPAFLE
IVAE(17)PAKPEGLKFFNVSSNSFSLYWRLPYGHVDRFCVDLIPDHGSVVISDLGVREYQADFYNTTPGTTYNVTVSTVSSST
YSSPASRTVTTNVTN(16)PGPPAFLAGERVGSAGILLSWNTPQHPNGRILSYIVKYKEVCPWMQTAYTQVTSKPDSLEVLLTN
LNPGTTYEIKVAAENSAGVGVFSAPFLFQTAESA(15)PGKVVNLTVEALNYSAVNLIWFLPRQPNGKITSFKISVKHARSGIV
VKDVLVKVEDLLSGRLPECNDNSESFLWSTTTPSTTFGKSTIPSSTTVTASTEASSQMSAVWNEPISFVITNLRPYTTYLFEVS
AVNTEAGYIDSTIVRTPESV(14)PEDPPQNFAKVNITSKSFSVMWDPPTIVTGKFSYRVELYGPSGHILDNSTKDLKFVFHNL
VPFTMYDVYVGAETSAGVGPKTNLTVFTPSDV(13)PGAVSDLHLVEVEATYIKIVWRKPQQPNGIITQYRVKVHVQEKEVTLE
NIILIGKNKHLIDSLEPYMINENIEPSPTWNSIETANELYEGSGEMFSSIQTVSPVILTTVSDDHLPAINGAAELHSTLDDQYA
TDILGEELSYIIKGLVPFTDYTISVSAFTAIGEGPPSVLTVRTREQVPSSVQSISYKNISSSSVLLYWDPPANPNGKIIHYTVY
AMELDTKRAFHTTTSNNSLLMTGLKKYTNYKMRVAASTVIGESALSEENDIFVRTPEDE(12)PDSPPQNVEIINVTATEINLK
WLPPEQPNGLITHYEVLYSDSSDLFVRNTSSTNISLTEMMPYTLYNISVRAFTRLGHGNQSSFPLLVRTSETV(11)PNSAPEN
ITYWNISSTEIELSFFPPSIPNGIIQTYTIYLKRINGTEERVINTTHLVLRITDLKKYTEYMIEVSASTMLGEGLRSAPLHILT
DEDA(10)PSSPPESLSVKQLSGVTVKLSWKPPLEPNGIILYYTVYVWNKTSKRSVNVTETSLEFTDLENNSEYSAYVAASTRF
GDGNIKSDTIIFRTSEGA(9)PGDPPKDIVYKNLTSTSIMLFWSPPQKPNGNIRYYSVYFRNDSGIFIQNFTSHDNDSDVNMSP
SAVLDDLAKYSHYTLWLTASTAVGDGNKTSEIIDVYTDQDI(8)PDGPVENLVYQNISSSSVNVSWLPPSQPNGLVFFHVSLSL
LQLGTNKILSFLTYNTSIIFDNLEKYTDYILKITPATDKGSSELHALSLHIRTDEDV(7)PESAPIIKTFSNLSTTSVMLSWDP
PVKPSGIIISYDLNLFGSEKNISFSTTNNFIILEDLVPSTLYSIYTAARTMKGSGPSSVLHFYTDESV(6)PLAPPQNLTITNY
TADSVWLKWEPSPQPNGVITRYNLKIYQNDTEKIFYQNISGSNNEAKLDGLKPFSTYFISVSAFTKLGNGNQFSNAVQFTTMES
V(5)PDVVQNVHCIATSWESIFMQWEPPASSNGVITYYIVTVEGSSTNFSSDTTLHTFQNLLSNISYQFKIKAATSAGDGEEQI
CNASTLPEEV(4)PSAPRDIVFSNVQSTSVTLNWRSPKSIPGYFQNYKITTQLQSIYCSNWETKECIEDEIHQYLYEKGVNAQI
EETVYGLKKYRWYRFAVAASTNAGYGSSSPWISTQTLPGS(3)PDGPPENVTVLATSPHSINISWSEPVVITGPTCYLIDITSV
DNENYKAQFLRTNDEGKILEISDLKAFTRYSVVIIAFTGDVSAAAIEGKASSPVIVSTFEAV(2)PEDPPNNVTFQKIPDEVTK
FQVTFVPPSEPNGNIQVYQAMVYNEDDPAAIRIHNLSVIDKTDQSVTAMIEGLKGGHTYNVSVYAINGAGAGPKIQLKITMDIK
E(1)PPRPKKKPAPVYDTNGALLVTATTITIRMPVCYYSDDHGPIKKIQVLVVEAGAQHDGNVTKWHDAYFNRPRPYFTNEGFP
NPPCIEGKEDLSGKEEIYVIGADTTCMISGSQDKICNGPLKPRKQYLFKFRATNVKGQFTDSDYSDPVKTLGEGRSGGSVEVIL
AVTLCILSVVLLVAAVYAFARIRQKQKEGGTYSPRDAEIIDTKFKLDQLITVADLELKDERFTRYSSFFFRRKEIFVIQLLSYR
KSIKPISKKSFLQHVEELCTNNNLKFQEEFSELPKFLEDLASTDADLPWNRSKNRFPNIKPYNNNRVKLMPDAGIPGSDYINAS
YVSGYLCPNEFIATQGPLPGTVGDFWRMVWETRAKTLVMLTQCFEKGRIRCHQYWPEDNKPVTVFGDIVITKLVEDIQIDWTIR
DLKIERHGDCMMVRQCNFTSWPEHGVPETTAPIIHFVKLIRASRAHDNTPMVVHCSAGVGRTGVYIALDHLTQHINDHDFVDIY
GLVAELRSERMCMVQNLAQYIFLHQCVLDLLTSRGSSQPICFVNYSALQKMDSLDAMEGDVELEWEETTM

>PTPRQ.fish |XP_009291679.PREDICTED: phosphotidylinositol phosphatase PTPRQ ptprq-
001 A8DZA4 ENSDART00000143740[Danio rerio]

MTTTAAAQDTFRYGLTDHHPLQHQLDLHESSVSIHDLTPGSDYSFDIQSFLGSDFSQTTSKNISTRPAGVCSLFVSDVNVTSAT
VSWAAAAGGFDFYRVIVRNSSHKWTIDASPHLQEVTVSGLWSGCSYNTTVQRFRNTISGAAATINIHSV(17)PASPEGLRVIS
VSPRSFSLHWLASPGCEKTYQVQLYPDHGNINITTDTADNNVQAQVSSVTPGTSYTVTVNAVASSGFSSPVSRILVTTIESS(1
6)APSKPEGERVGSTGILLSWRMPMPLDPSIHSFVIRYKEMCPYPDPSFTEITKSLDIPETLLNTLTPGATYNIKVAAVNKAGV
GPFSQSLYYKTAEAP(15)PGLVSNLTAFAEDHTSVIVTWFLPVRINGLITKFAVKVKHARTGQIVRTKELNAEDIMNGALPHC
NDAADILSRGTPSPSQTSQLTSAVLPPITLSAVPPASIWSVPISVKIDELRPYTPYVFEVSAFTSDGEGQIASTMVRMPEAA(1
4)PEDPPQNVVLRNITSKSVSLTWEPPKIITGRFSYVIQLHSSEGLISENSTIDQMFIYTGLTPYTTYYIHVMAKSAGAAGPAA
VINITTLAEA(13)PSAVSLLKAEAVDSTSVRLSWRSPIQPNGLITHYRILVLYHDTLVQDITLRGQNLISPLRRNARSLDFTT
ESVFTLTSHETFSTLGRDFTNTDTEAPSALPLTPSTPHTPPPWLTVTHDRTDASVQTQTDAVTEDLTTEHPPVTPVLPATEVSA
DLSSDQITHVVRRLSPFTEYKFSVSAKNIIGEGPSTEVTVKTNEQVPSSVQNVSYQNLSSTSIRVSWEPPLNPNGKITHYAVYA
QNLLTNQELRQMTDTTTAVLTGLDKYSSYKVRVAASTAVGESPLTNEDYIYVVTLEDV(12)PDSPPRGLTIVKTTSSTATLSW
SPPEKPNGIIRMYEISYTNGTYSNTVNSTSASATLRYLKPHTHYNVTVRAFTLLGHGEQISETLQMLSGEDV(11)PGSPPYDL
SYESIGSSSVNVSWSPPLLANGVILFYNVEYWNATQSLNQTTHMPYIVLSNLRKYAHYRISVQAATQVGMGNHTSEILNITTLE
DV(10)PSSPPKFLIARKLSDTEVELSWEAPEEANSEILYYIVRVWNLSTEFVANVTETSVVVSVDGPGQYNASVSSWTRLGDG
GLLIYITFSTIESA(9)PSDPPQDVVYTLLTISTVRLSWRPPNEPNGIIQYYTIYYTDNNTEYTERVPGSEHQLLLSDLQAGQD
YSVWMSSSTSVGDGGLFSPSLNFTTLEDV(8)PSGPVHNLSATIYSSTAVVISWDPPLEPNGRVYYQLSLQEAGITHPSINRTV

NMTITKTTTDTIYLFTKLRKYFPYIINVTPATSAGSAVNHTTMLHLRTDEDV(7)PSSPPLSGSNKNLSSTSIQVSWFPPVEAN
GEIIEYAVNLQGPSTSNKNYTSETHLVLSELTPFTPYNLSIAAVTRVGTGPPVVLSLHTDEAG(6)PMSPPRNLTIFNHTANSV
WLQWEPSPEPNGVVQLYGFRILELNTDSFRYQNSSDASTQAELGGFKPHNSYEISVCTFTRAGNGDQYSLPVLFTTNESV(5)S
DAVGNLSCSGLDWDSVYMEWELPDHPNGEILYYLIRSGDLEDEAHPVVLTHTVAHTLTGLSPHAFYLITVAAVNSAGVGEEANC
SAHTPPESV(4)PGPSHSLTVTDVTSDSVSVSWQRPVHVPGLLQGYNVEIEQLSRNCERQQQQVESCMEAQILEWVEGETTRAT
LTSLLKYRQYRIRVVAFTRAGAGEPSEWIYTQTLAGN(3)PDAPPGAISVVPSANGLKIEWDKPSVISGPTSYIIDITALDGSG
YNITLVRHSEEIRTVIVGNLSAFTLHSVTITAFTGPLSNARRDGKASEPVLIRTLEDE(2)PKDPPKNVTLTVIPEEVTRVYVT
FSPPDEPNGNISAYRVDIYRNGQLDFFINSLSVISNPNNTMTAIIDGLKGGFNYSIRIAAVNGAPGFGPSSEVHVTTGVKA(1)
PPKPKKTPRAALNSAGVIISTSKTITIEMPECFFTDDHGPIQKVQVIVSEPAVMDYGNLSNWKSVFLHPTAPYLTDDGFLNPEC
PKNSERMSSSTKTYVIGEDEGCLSEDAETLCNGPLKPKTHYVFKFRATNIRGQFTDSEYSDKVRTADDRLLTRDEQIILGVLLS
FFLALFLILIIYGSVKIHRRKKEGGTYSPRQAEIIETKFKLDQLIAVADLELKEEKINRLLSYRKSLKPISKKSFLQHVEDLCA
NDNAKFQEEFAELPKLLQDLATSDADLPWNRSKNRFTNIKPYNNSRVKLLSEPGMPGSDYINASFVSGYLCPNEFIATQGPLPS
TVADFWRMIWETGTKTIVMLTQCFEKGRIRCHQYWPEDNKPVTVFADIIITKLTEDVRPDWTVRALKVERHGSYMIVHHFNYTS
WPEHGVPESSSTLVQFVKAVRSNRGHENTTIVVHCSAGVGRTGVFIALDHLIQHLRDHEFVDIYGLVAELRSERMCMVQNLAQY
MFLHQSTLDLLSAKGNSQSIWFVNYSALEKMDSLDAMEGDVELEWEETTM

PTPRB

>PTPRB.human PTPRB-001 ENST000003344142200 [Homo sapiens]

MEAEFYMVILTCLIFRNSEGFQIVHVQKQQCLFKNEKVVVGSCNRTIQNQQWMWTEDEKLLHVKSALCLAISNSSRGPSRSAIL
DRCSQAPRWTCYDQEGFLEVENASLFLQKQGSRVVVKKARKYLHSWMKIDVNKEGKLVNESLCLQKAGLGAEVSVRSTRNTAPP
QILTTFNAVPDGLVFLIRNTTEAFIRNAAENYSQNSSERQHPNLHMTGITDTSWVLSTTQPFSSTTEETGLAEPERCNFTLAES
KASSHSVSIQWRILGSPCNFSLIYSSDTLGAALCPTFRIDNTTYGCNLQDLQAGTIYNFRIISLDEERTVVLQTDPLPPARFGV
SKEKTTSTSLHVWWTPSSGKVTSYEVQLFDENNQKIQGVQIQESTSWNEYTFFNLTAGSKYNIAITAVSGGKRSFSVYTNGSTV
(15)PSPVKDIGISTKANSLLISWSHGSGNVERYRLMLMDKGILVHGGVVDKHATSYAFHGLTPGYLYNLTVMTEAAGLQNYRW
KLVRTA(14)PMEVSNLKVTNDGSLTSLKVKWQRPPGNVDSYNITLSHKGTIKESRVLAPWITETHFKELVPGRLYQVTVSCVS
GELSAQKMAVGRTF(13)PDKVANLEANNNGRMRSLVVSWSPPAGDWEQYRILLFNDSVVLLNITVGKEETQYVMDDTGLVPGR
QYEVEVIVESGNLKNSERCQGRTV(12)PLAVLQLRVKHANETSLSIMWQTPVAEWEKYIISLADRDLLLIHKSLSKDAKEFTF
TDLVPGRKYMATVTSISGDLKNSSSVKGRTV(11)PAQVTDLHVANQGMTSSLFTNWTQAQGDVEFYQVLLIHENVVIKNESIS
SETSRYSFHSLKSGSLYSVVVTTVSGGISSRQVVVEGRTV(10)PSSVSGVTVNNSGRNDYLSVSWLLAPGDVDNYEVTLSHDG
KVVQSLVIAKSVRECSFSSLTPGRLYTVTITTRSGKYENHSFSQERTV(9)PDKVQGVSVSNSARSDYLRVSWVHATGDFDHYE
VTIKNKNNFIQTKSIPKSENECVFVQLVPGRLYSVTVTTKSGQYEANEQGNGRTI(8)PEPVKDLTLRNRSTEDLHVTWSGANG
DVDQYEIQLLFNDMKVFPPFHLVNTATEYRFTSLTPGRQYKILVLTISGDVQQSAFIEGFTV(7)PSAVKNIHISPNGATDSLT
VNWTPGGGDVDSYTVSAFRHSQKVDSQTIPKHVFEHTFHRLEAGEQYQIMIASVSGSLKNQINVVGRTV(6)PASVQGVIADNA
YSSYSLIVSWQKAAGVAERYDILLLTENGILLRNTSEPATTKQHKFEDLTPGKKYKIQILTVSGGLFSKEAQTEGRTV(5)PAA
VTDLRITENSTRHLSFRWTASEGELSWYNIFLYNPDGNLQERAQVDPLVQSFSFQNLLQGRMYKMVIVTHSGELSNESFIFGRT
V(4)PASVSHLRGSNRNTTDSLWFNWSPASGDFDFYELILYNPNGTKKENWKDKDLTEWRFQGLVPGRKYVLWVVTHSGDLSNK
VTAESRTA(3)PSPPSLMSFADIANTSLAITWKGPPDWTDYNDFELQWLPRDALTVFNPYNNRKSEGRIVYGLRPGRSYQFNVK
TVSGDSWKTYSKPIFGSVRTK(2)PDKIQNLHCRPQNSTAIACSWIPPDSDFDGYSIECRKMDTQEVEFSRKLEKEKSLLNIMM
LVPHKRYLVSIKVQSAGMTSEVVEDSTITMIDR(1)PPPPPPHIRVNEKDVLISKSSINFTVNCSWFSDTNGAVKYFTVVVREA
DGSDELKPEQQHPLPSYLEYRHNASIRVYQTNYFASKCAENPNSNSKSFNIKLGAEMESLGGKCDPTQQKFCDGPLKPHTAYRI
SIRAFTQLFDEDLKEFTKPLYSDTFFSLPITTESEPLFGAIEGVSAGLFLIGMLVAVVALLICRQKVSHGRERPSARLSIRRDR
PLSVHLNLGQKGNRKTSCPIKINQFEGHFMKLQADSNYLLSKEYEELKDVGRNQSCDIALLPENRGKNRYNNILPYDATRVKLS
NVDDDPCSDYINASYIPGNNFRREYIVTQGPLPGTKDDFWKMVWEQNVHNIVMVTQCVEKGRVKCDHYWPADQDSLYYGDLILQ
MLSESVLPEWTIREFKICGEEQLDAHRLIRHFHYTVWPDHGVPETTQSLIQFVRTVRDYINRSPGAGPTVVHCSAGVGRTGTFI
ALDRILQQLDSKDSVDIYGAVHDLRLHRVHMVQTECQYVYLHQCVRDVLRARKLRSEQENPLFPIYENVNPEYHRDPVYSRH

>PTPRB.mouse 201 ENSMUST00000092167 [Mus musculus]

MLRHGALTALWITLSVVQTGVAEQVKCNFTLLESRVSSLSASIQWRTFASPCNFSLIYSSDTSGPMWCHPIRIDNFTYGCNPKD
LQAGTVYNFRIVSLDGEESTLVLQTDPLPPARFEVNREKTASTTLQVRWTPSSGKVSWYEVQLFDHNNQKIQEVQVQESTTWSQ
YTFLNLTEGNSYKVAITAVSGEKRSFPVYINGSTV(15)PSPVKDLGISPNPNSLLISWSRGSGNVEQYRLVLMDKGAIVQDTN
VDRRDTSYAFHELTPGHLYNLTIVTMASGLQNSRWKLVRTA(14)PMEVSNLKVTNDGRLTSLNVKWQKPPGDVDSYSITLSHQ
GTIKESKTLAPPVTETQFKDLVPGRLYQVTISCISGELSAEKSAAGRTV(13)PEKVRNLVSYNEIWMKSFTVNWTPPAGDWEH
YRIVLFNESLVLLNTTVGKEETHYALDGLELIPGRQYEIEVIVESGNLRNSERCQGRTV(12)PLAVLQLRVKHANETSLGITW
RAPLGEWEKYIISLMDRELLVIHKSLSKDAKEFTFTDLMPGRNYKATVTSMSGDLKQSSSIKGRTV(11)PAQVTDLHVNNQGM
TSSLFTNWTKALGDVEFYQVLLIHENVVVKNESVSSDTSRYSFRALKPGSLYSVVVTTVSGGISSRQVVAEGRTV(10)PSSVS
GVTVNNSGRNDYLSVSWLPAPGEVDHYVVSLSHEGKVDQFLIIAKSVSECSFSSLTPGRLYNVTVTTKSGNYASHSFTEERTV(
9)PDKVQGISVSNSARSDYLKVSWVHATGDFDHYEVTIKNRESFIQTKTIPKSENECEFIELVPGRLYSVTVSTKSGQYEASEQ
GTGRTI(8)PEPVKDLTLLNRSTEDLHVTWSRANGDVDQYEVQLLFNDMKVFPHIHLVNTATEYKFTALTPGRHYKILVLTISG

DVQQSAFIEGLTV(7)PSTVKNIHISANGATDRLMVTWSPGGGDVDSYVVSAFRQDEKVDSQTIPKHASEHTFHRLEAGAKYRI
AIVSVSGSLRNQIDALGQTV(6)PASVQGVVAANAYSSNSLTVSWQKALGVAERYDILLLNENGLLLSNVSEPATARQHKFEDL
TPGKKYKMQILTVSGGLFSKESQAEGRTV(5)PAAVTNLRITENSSRYLSFGWTASEGELSWYNIFLYNPDRTLQERAQVDPLV
QSFSFQNLLQGRMYKMVIVTHSGELSNESFIFGRTV(4)PAAVNHLKGSHRNTTDSLWFSWSPASGDFDFYELILYNPNGTKKE
NWKEKDVTEWRFQGLVPGRKYTLYVVTHSGDLSNKVTGEGRTA(3)PSPPSLLSFADVANTSLAITWKGPPDWTDYNDFELQWF
PGDALTIFNPYSSRKSEGRIVYGLHPGRSYQFSVKTVSGDSWKTYSKPISGSVRTK(2)PDKIQNLHCRPQNSTAIACSWIPPD
SDFDGYSIECRKMDTQEIEFSRKLEKEKSLLNIMMLVPHKRYLVSIKVQSAGMTSEVVEDSTITMIDR(1)PPQPPPHIRVNEK
DVLISKSSINFTVNCSWFSDTNGAVKYFAVVVREADSMDELKPEQQHPLPSYLEYRHNASIRVYQTNYFASKCAESPDSSSKSF
NIKLGAEMDSLGGKCDPSQQKFCDGPLKPHTAYRISIRAFTQLFDEDLKEFTKPLYSDTFFSMPITTESEPLFGVIEGVSAGLF
LIGMLVALVAFFICRQKASHSRERPSARLSIRRDRPLSVHLNLGQKGNRKTSCPIKINQFEGHFMKLQADSNYLLSKEYEDLKD
VGRSQSCDIALLPENRGKNRYNNILPYDASRVKLCNVDDDPCSDYINASYIPGNNFRREYIATQGPLPGTKDDFWKMAWEQNVH
NIVMVTQCVEKGRVKCDHYWPADQDPLYYGDLILQMVSESVLPEWTIREFKICSEEQLDAHRLIRHFHYTVWPDHGVPETTQSL
IQFVRTVRDYINRSPGAGPTVVHCSAGVGRTGTFVALDRILQQLDSKDSVDIYGAVHDLRLHRVHMVQTECQYVYLHQCVRDVL
RAKKLRNEQENPLFPIYENVNPEYHRDAIYSRH

>PTPRB.chicken PTPRB-201 ENSGALT00000016330 **[Gallus gallus]**

MSSPNQQWQWMADDKLFHMKSGQCLSISTRSALSSRSIIINCSQAVRWTCHEEDGLLKVANSSLFLTKQGQKVMAKQSKKYLHT
WMQLEASETGKPVYVNLCSKQAAHNSLSTGLPLPNFPDLWMGTMNSTTTTSSPLNTTTSRPDNLPASSSTQMSIRNVTEHFTKN
FIENLYFDLKSAVTEKPTVPTTPPQYSSTTEETKSAYLLTDRGKTHSCSFIYALFQSPNTHKQLWQRRGMQVPAPYTEETNSEH
SVPPVFRFHVDKPQLSNLRANLASSLAKCLQSVSVKDCTTHLLKAHLFSTTLEIIMLTILYTYITTNITSKSALLRWSAAAHAC
NFNLTGRSEDGRPAGCRPASAGNGSYGCTVRDLEAGTWYHLRIEPLADGEAANVTLQTDPLPPPRFEINKEKTTFTSLQVRWDP
SPGKVDLYNIVVFDHSNKKLQESSIPGRFSKTEETFTGLVPGNKYNIVLTAVAGNKSTPEFRINGSTA(15)VPSPVKNIQVIV
KTDSIHASWSPGSGHVDLYKLVLFDNHGTVHESHQENPLTSYIFSGLTAGHLYNLSVITQAAELESTAFRIVRTA(14)PAEVL
DLTVTNDDSFDTLKVKWRRPSGGVDFYNITLSHLRSVKEVKTLQPAVTETHFDKLTPGRLYQITARTISGELFTDKMATGRTF(
13)PQKVSELKAGGGGWLRTLRVNWLPPAGDLERYHLLLWNHSALVLNTTLGKNVTEYLVRDVGLIPGRQYEVEVVVESGDLQS
RASCTGRTA(12)PEPVLQLRVKHANESSLSVMWVTPVAEWDSYVVSLGDRDLTVIKKGLGKEAKEFTFNELVPGRKYTATVTT
ISGILSNWTSVEGRTV(11)PAQVTGLTVASQGSTNSLFTNWTKALGDVDSYQVLLIHENVVIKNETVPSETNEYHFYPLKPGG
LYSVVVTTVSGGISSRQTIAEGRTV(10)PSSVTGVTVNNSGRSDYLSVSWLPASGDVDSYLVTLSHDDQIIQTLTISKSLSEC
SFSSLTPGTLYNVMITTKSGKYENHSFSRERTV(9)PSGVQGLTVSNSARSDYLKVSWLHASGSFDSYEVIIKNNNDFIQTKSV
PKDENECVFTNLVPGRQYSVTVSTRSGKYETSERVFGRTM(8)PESVKGLTLSNRSTEDLRVTWSKAEGDVDKYEIQLLYNDMK
IFPPIFLGNTIEECWFTALTPGRLYKIVVLTISGDAQRATFIEGLTI(7)PSAVRNINVSPNGMTDSLKVSWTPGGGDVDSYTV
TIFQQNHQLDSRSVSKDVSEHTFHDLEAGEQYRVVVQSNSGALHNSLAAFGRTI(6)PASVQELLADHAYSSHSLLVSWQKAPG
VAERYDILLLNEQGVLLSNKSEPATAKQHKFEDLLPGKKYKIHILTVSGGLFSKRAETVGRTV(5)PAAVTNLKVTKNTTDQLS
FSWTTSQGELDSYDIFLYNPDKSLHDRISGEQHLQQCSFQNLRQGRMYRMVIVTHSGDLTNESSVFGRTA(4)PAPVVGLKASN
RNMTDSLWFTWNPAAGDVDFYEFNLYNPNGTQKESLQGKDLKEWYFQGLVPGRKYTLVVVTHSGDLINTANAEGRTA(3)PSPP
NTVSFTDVANTSLSITWLGPPDWTDYDDFELQWLPKDPLTVFNPYSSSKSKVRIIYGLRPGRLYKFSVRTVSGDSWKTYSQSQS
ESVRTR(2)PDKIQSLHCRPQTSTAIACSWTPPDSDFDGYSVECKKLDTREVEFSKRIEKDRTLLNIMTLVPHKRYLVSIKVHS
ADMTSEVVEDSTITMIDR(1)PPQPPPDIRVNKKEVLITKSSINFTFNCSWFSDTNGAVKYFTVVVREADGSEGPKPDEQHPLP
SYLEYKHNDSIRIYQTNYFASRCAENPDSDYKSFDIKLGGEMENLGGKCDPDQQKFCDGPLKPRTAYRISIRAFTQLFSEDPKE
LPQPLFADTFFSLPITTEAEPLFGVIEGVSAGLFLIVMLVAVTALFVCRQKVSNGHERPTARLSIRRDKPLAVHLNLGQKGNRK
TSSPIKVSHFEAHFTKLQADSNYLLSKEYEDLKDVGRNQTCDIALLPENRGKNRYNNILPYDTSRVKLSNVDDDPCSDYINASY
IPGNNFRREYIATQGPLPGTKDEFWKMAWEQNVHNIVMVTQCVEKGRVKCDHYWPLDQDSLYYGDLIVEMLSESVLPEWTIREF
KICSEEQLDSTRLIRHFHYTVWPDHGVPETTQSLIQFVRTVRDYINRTPDTGPTVVHCSAGVGRTGTFIALDRILQQLDSKDTV
DIYAAVHDLRLHRVHMVQTECQYVYLHQCVRDVLRARKLRSEQENPLFPIYENVNPEYHRDAVYSRH

>PTPRB.frog xenpopus ptprb-201 ENSXETT00000024858 Xenopus tropicalis

MLRYAFGLSLWIVLNLIKNVEPVKCTINNTDVTVTSQSIYLEWTSPGSHCNFSLTCSSHNFWEITCNPIQKSNDSYECTLSGLE
AGTVFDLRIVSLQDGEMKNLSLQTDPLPPSNFVVNKNSVTSSSVQANWAPSSGKVDLYKVKLLDSKKEIVQEIEVSGISLTKEV
TFKNLTPGTKYSIEITAISGNKRSPTSVINASTV(13)PSTVKSIDANSKTDTIDASWKPGHGNIDSYKLILLNEDRKITEVVL
EQNITSYSFHGLLPGYVYNLTVISEAAGLRTWNSKLIRTA(12)PAEVQAISVQSDGRTDALKVQWKEPRGKLDFYNVTLSDTG
SIKYSRTVHPGTPTEITFTGLTPGHLYQVGISTIAGELSTSRMATGQTI(11)PEKVADLKAVSNGLRSLRLSWVPPSGDWDKY
NIVLYNNTAVLVNTTIDKKYREYVIQDIGLIPGRQYEAVVIVESGKFKNLARCKGRTA(10)PPAVMDLRLKHANESSLTIMWV
MPVAEWDNYVVSLADRDLTPVNKVLSKEAKEFTFNHLAQGRKYIATVTSISGDLSSRASVEGRTV(9)PAQVTNLNVENQGTTS
SLHADWTKAPGDVDSYQVLLIHEKIVIKNETVSNEINKYSFHSLKPGGLYSVVVTTVSGGISSRQTVSEGRTGRKLLSIISS(8
)NTGSLTPFLHQSWSSNSLRYIGWTSLICQGKFLAKDLFFAWPGIECSFPCVIKGALYFVRMDYQQTVGTNRYPRDNEAIPLVV
VNTCCINCLSSSYRKLSVIESISPINKRNNIKKRSVLSVSSEYTHPRLRHSGCVTTRANSGMLKLQLPGAGLLVPEAVKKLTLA
ERSSEELHVTWSRADGDVDHYEIQLLFNDMKVFPPITLNNTADEYKFTSLTPGRLYKIVVLTFSGEAQRAMFVEALTV(7)PST
VKNIHISPNGMTNSLKVNWTPGGGDVDSYTVSIFHHNSPIGSHTTSKHIFEHTFQNLNPGELYKVDVQTNSGSLHKSLESFGRT
I(6)PATVHSLSADNAYSSHLLIVSWQSAKGVTDRYDILLLSDHGIVITNKSVPIMAKSHKFEDLVPGKKYRIQVFTVSGGLFS
AAVETEGQTV(5)PAAVSDVKITGNSTDSLSFTWNSSEGELDSYGIFLYNPDNSLHDRKSGNPDLRHCSFQGLLPGRLYKMVIV
THSGSLTNESSIHGRTV(4)PAPVSSLQVSNRNTSESLWFTWAPALGDVDIYELILYNPNGTEKEKIQDKDLTESHFQNLVPGR

LYTLVIFTHSGDLFNKATAVGRTE(3)APQPPNSVSFADVKINSLSIMWLGPPDWTDYDDFELQWSPKDLLVVVNPYSIGRSKG
RIINGLYPGRQYTFGVQSVSGNTGKTLSPAIFGTVRTK(2)PDKIHHLHCRPQTSTAISCSWTPPDSDFDGYSVECRRNGSDDV
EFSKRIEKDKSSITISTLVPHKRYVVSIKVHSADMTSQVIEDSAITMIDR(1)PPPPPLLIRVNKKDTFISKSSIHFRFNCSWF
SDTNGAVKYFTVIVSEADGNDNQRPEASLPLPSYADYKTNKSTKIYQTSYFPSRCAENPDYNIQSYKIKLGTGMELLGGKCDQN
ENKYCDGPLSPRTSYRISVRAFTQLFTEEMRTFPEPLYSDTFFSLPITTEAGSLFFNKNITDLPLQIFPQTKAMILVVLCRYEQ
DPMTTHLSSQIERIPSVHLNVGHIQIGDRISSRPILT**AQFEEHFSKLQTDSNYLLSREYENLKDFGRDQSSDTALLPENRGKNR**
YSNILPYDSTRVKLANVDDDPCSDYINASYMPGINFRREYIATQGPLPATKDDFWKMVWEQNVHIIVMVTQCTERGRAKCDHYW
PMDQDSYYYGDLIVQMLSESVLPEWTIREFKICSEDQIDAPRLVRHFHYTVWPDHGVPETTQSLIQFVRTVRDYINRTPGSGPT
VVHCSAGVGRTGTFIVLDRMLQQVDTVDSVDIFGAVRDLRIHRMYMVQTECQYVYLYQCVRDVLRARKLRNEQDNPLFPIYENV
NPEYHRDAVYLRH

>PTPRB.fish Zebrafish ptprb-202 ENSDART00000127299 NP_001303656.1 FN428722 Danio rerio

MLRNKAFHAAVWAACGILSTMVVDATECSIEIVKVISSTESIRLTLDNANVKCQYTVSVKDRHTDSKGCRQDREHSKCQIESLD
PGTWYHLDVISTLDEKQQSQRAVTLQTRPSAVENLQVSGDANSLDVSWQPGKGKTERYWIVLIDSSGRDSAWNSTVASTATSYT
MKGLISGRLYNITVVTEVGELQNSASTQAQTA(14)PASVSKLRTENNGDRNSIRVLWDKASGDVDSYLVSLTTPGSNSIEKVL
PPDDTYVVFENLSPGKGYQVSVSTRSGALSNKTWITGKAD(13)PGKVSDLVIENLSVRGALKISWTPPSGEWEHIRVALSNGS
EVLRNQTVGRTAKEILLSGLNLLPGRVYRMAVSVENGGLANTVIYEGEIG(12)LPPVSQLHVRHSDETSLSALWAHAASSSSR
DGYIIQLFQSNTSTVIQTRNLSRDMRECTFNVLTPGRLYDITVTTTAKIIRSSATLQGRTQ(11)PLKVNHLKLSNKGSTDSLN
ASWEKPLGDLDFYHMLLLRDQQTVHNITASANTTSRLLPFLRPGALHKILVTTISGSQTSKLAEAECRTV(10)PAAVSDITVT
NSGPDFLNVSWKAAEGDVDNYVVMLKDQEKIVHTLATSKATTECVFRSLVSGRLYSISIATHSGSYRNQTLLQERTK(9)PSTV
QNPTAIHSARDDFLKLYWNHASGDYDYYVVTIEHNGTRLQSQKLNRTQSDCAFSDLVPGRLYNLTVSTWSGQHYSAVSIYGRTF
(8)PGAVGNLSLTERGGTFLRVSWTSAPGDVDHYELQILFNDTQVSAAVNLSSAIGEHLFSALTPGRLYKIVLSTHSGSYQRAE
ILEGRTV(7)PSQVQSVHLSAGTADGSLRVSWSSADGDLDFYSVSLFQETHLQDSRRVPKHITQAEFYNLIPGQLYSVTVQSVS
GSQTNNSTTTGRTE(6)PSTVTGLRADNELSTHSLLVSWNPAVGVHDGYRLWLLDDGGTNIHNASVPAASRHHLFENLTPGRRY
QAHVRTLSGTAESKDVVAEGQTR(5)PSAVSALHVCSNTSSDLSFCWASAVGWVDGYELYLYDRDETLHYHTTLGSDALGWSFT
LLQPGTHYKMTITSKSGKLSNQSSVWAHTA(4)PASVPELHVENQGQTDSLLLSWTPAPGGLTGYSVTVDGREQRVGPEVTQVV
FHSLVAGRLYLATVQTWSEDLSNSTTAVGRTV(3)PAAPSSVSVSCSGGSVDMKWHVPDTGDYEDFEVTWFPQDTLYISHLRPT
QRILEGLHPGRLYNISLRTVSGKRHSPVTYSRPVYHTIRTP(2)PLPAPSIHCFPLSSTSVSCSWTPPHSDYDGFVVQCHRQGS
GKAVYTHTLGNHTLSQQFDRLEPFKNYTIYVAVMSGDKQSSTVKNSVITMIDR(1)PPVPPVTVRVNEHSAVITHFTIRFKFNC
SWFSDANGAIRYFTIIATESNDVDNGLPEQRHPLPSYLDYRQNHSIKAYQTGYFHSTCAEGSDGKVQVFEINLGAGMKHLGGAC
KLDPESIQHGSHLCDGPLRSRTSYRLSVRAFTQLFDEENREFPHPLYTDTYLSLPLLTQSAPRSGLTGGITAALFLITMVLALT
ALLIYRKRAHKIAVQESPVMKMCKWKALPTSQMCLRIRSPVQA**ANFESHLAKLQSDSSYLLSEEFEGLKDVGRIQTQNAARLLG**
NRNKNRYNNILPYDSTRVRLSCLEDDPCSDYINANYIPGNNFRWEYIATQGPLPGTKDDFWRMVWEQNVHSLVMVTQCVERGMV
KCDHYWPTDSEPLCYGDIVVQLLSEKVFPEWTIRDFKISCESQLRYPRMVRQFHYTIWPDHGVPDTTQSLIQFVRTVRDFINRT
NSPGISVVHCSAGVGRTGTFIVLDRALQQLDRNCTVDIYGCVFDLRLHRSYMVQTECQYAYIHQCVRDVLRARKLGCERDNPLF
PIYNNII

PTPRJ

>PTPRJ.human ENSG00000149177 [Homo sapiens]

MKPAAREARLPPRSPGLRWALPLLLLLLRLGQILCAGGTPSPIPDPSVATVATGENGITQISSTAESFHKQNGTGTPQVETNTS
EDGESSGANDSLRTPEQGSNGTDGASQKTPSSTGPSPVFDIKAVSISPTNVILTWKSNDTAASEYKYVVKHKMENEKTITVVHQ
PWCNITGLRPATSYVFSITPGIGNETWGDPRVIKVITE(8)PIPVSDLRVALTGVRKAALSWSNGNGTASCRVLLESIGSHEEL
TQDSRLQVNISGLKPGVQYNINPYLLQSNKTKGDPLGTEGGLD(7)ASNTERSRAGSPTAPVHDESLVGPVDPSSGQQSRDTEV
LLVGLEPGTRYNATVYSQAANGTEGQPQAIEFRTN(6)AIQVFDVTAVNISATSLTLIWKVSDNESSSNYTYKIHVAGETDSSN
LNVSEPRAVIPGLRSSTFYNITVCPVLGDIEGTPGFLQVHTP(5)PVPVSDFRVTVVSTTEIGLAWSSHDAESFQMHITQEGAG
NSRVEITTNQSIIIGGLFPGTKYCFEIVPKGPNGTEGASRTVCNRTV(4)PSAVFDIHVVYVTTTEMWLDWKSPDGASEYVYHL
VIESKHGSNHTSTYDKAITLQGLIPGTLYNITISPEVDHVWGDPNSTAQYTR(3)PSNVSNIDVSTNTTAATLSWQNFDDASPT
YSYCLLIEKAGNSSNATQVVTDIGITDATVTELIPGSSYTVEIFAQVGDGIKSLEPGRKSFCTD(2)PASMASFDCEVVPKEPA
LVLKWTCPPGANAGFELEVSSGAWNNATHLESCSSENGTEYRTEVTYLNFSTSYNISITTVSCGKMAAPTRNTCTTGITD(1)P
PPPDGSPNITSVSHNSVKVKFSGFEASHGPIKAYAVILTTGEAGHPSADVLKYTYEDFKKGASDTYVTYLIRTEEKGRSQSLSE
VLKYEIDVGNESTTLGYYNGKLEPLGSYRACVAGFTNITFHPQNKGLIDGAESYVSFSRYSDAVSLPQDPGVICGAVFGCIFGA
LVIVTVGGFIFWRKKRKDAKNNEVSFSQIKPKKSKLIRV**ENFEAYFKKQQADSNCGFAEEYEDLKLVGISQPKYAAELAENRGK**
NRYNNVLPYDISRVKLSVQTHSTDDYINANYMPGYHSKKDFIATQGPLPNTLKDFWRMVWEKNVYAIIMLTKCVEQGRTKCEEY
WPSKQAQDYGDITVAMTSEIVLPEWTIRDFTVKNIQTSESHPLRQFHFTSWPDHGVPDTTDLLINFRYLVRDYMKQSPPESPIL
VHCSAGVGRTGTFIAIDRLIYQIENENTVDVYGIVYDLRMHRPLMVQTEDQYVFLNQCVLDIVRSQKDSKVDLIYQNTTAMTIY
ENLAPVTTFGKTNGYIA

>PTPRJ.mouse Ptprj-201 ENSMUST00000168621 [Mus musculus]

MTRGGGRGSSRGRGSRELGATRGGWAPLAPPREAPASLRPRPLRARRARLRRVAAAAAAAMSPGKPGAGGAGTRRTGWRRRRRR
RRLETETRAPGFGHTAGRVPGTFQGAQGMKPAARETRTPPRSPGLRWALLPLLLLLRQGQVLCAGAAPNPIFDIEAVVSPTSVL
LTWKHNDSGASECRIENKMESNLTFPVKNQTSCNITGLSPGTSYTFSIISVTTNETLNKTITTE(8)PWPVSDLHVTSVGVTQA
RLTWSNANGTASYRMLIEELTTHSSVNISGLKPGTNNSFAFPESNETQADFAVAEEVPD(7)ANGTKRIPVTNLSQLHKNSLVS
VDPPSGQDPSLTEILLTDLKPDTQYNATIYSQAANGTEGQPRNKVFKTN(6)STQVSDVRAMNISASSMTLTWKSNYDGSRTSI
VYKIHVAGGTHSVNQTVNKTEAIILGLSSSTLYNITVHPFLGQTEGTPGFLQVYTS(5)PDQVSDFRVTNVSTRAIGLAWRSND
SKSFEIFIKQDGGEKHRNASTGNQSYMVEDLKPGTSYHFEIIPRGPDGTEGLSSTVNGSTD(4)PSAVTDIRVVNISTTEMQLE
WQNTDDASGYTYHLVLESKSGSIIRTNSSQKWITVGSLTPGTLYNVTIFPEVDQIQGISNSITQYTR(3)PSSVSHIEVNTTTT
TAAIRWKNEDAASASYAYSVLILKTGDGSNVTSNFTKDPSILIPELIPGVSYTVKILTQVGDGTTSLVPGWNLFCTE(2)PEPV
TSFHCEVVPKEPALVLKWACPFGMYTGFELGVRSDSWDNMTRLENCTSDDDTECRTEVAYLNFSTSYNISIATLSCGKMALPAQ
NICTTGITD(1)PPTPDGSPNITSVSHNSVKVKFSGFEASHGPIKAYAVILTTGEAAQPSADVLKYTYEDFKRGASDTYVTYLI
RIEEKGQSQGLSEVLNYEIDVGNQSTTLGYYNGRLEPLGSYRACVAGFTNITYNLQNDGLINGDESYVSFSPYSEAVFLPQDPG
VICGAVFGCIFGALAITAVGGFIFWRKKRTDAKNNEVSFSQIKPKKSKLIRVENFEAYFKKQQADSNCGFAEEYEDLKLIGISL
PKYTAEIAENRGKNRYNNVLPYDISRVKLSVQTHSTDDYINANYMPGYHSKKDFIATQGPLPNTLKDFWRMVWEKNVYAIVMLT
KCVEQGRTKCEEYWPSKQAQDYGDITVAMTSEVVLPEWTIRDFVVKNMQNSESHPLRQFHFTSWPDHGVPDTTDLLINFRYLVR
DYMKQIPPESPILVHCSAGVGRTGTFIAIDRLIYQIENENTVDVYGIVYDLRMHRPLMVQTEDQYVFLNQCVLDIIRAQKDSKV
DLIYQNTTAMTIYENLEPVSMFGKTNGYIA

>PTPRJ.chicken   PTPRJ-202 ENSGALT00000045335 NP_989952.1| [Gallus gallus]
MRRLPLLPPCPLLLLLLLPAEVRCTTACTDDCSLKNVTEEMGTSSNDELSVNATSGNRRLSEDVSLPGRAMSDQNSVAQPRAVL
DLKTEYVGVTSVNLTWTVNDTASDSYTYRIEVRNGSSINNKTSDITDAEITGLIPGTLYTFTVFAVAADGQTAGEGASISLYTK
PSPVLDLKAEYVGVTSVNLTWTVNDTASASYTYRIEVTSDSSIDSLTSSVTMAEITGLIPGTLYSFKVFAVAADNRTEADGASI
SLYTK(6)PSPVLDLKAEYVGVTSVNLTWTVNDTASASYTYRIEVTSDSSIDSLTSNVTMAEITGLIPGTLYNFTVFAVAADNR
TEADGAFTSLYTK(5)PTPVTDLKAEHGVTSVSLNWMVNDTASDSYTYRIEVRNGHSVNNKTSNIPETEITGLNPGTLYTFTVF
AVAADGETEGEGASISVYTK(4)PRAVLHLKTEYVGVTSVNLTWTVNDTDSASYTYRIEVRNGSSINNKTSDITDAEITGLDPG
TLYIFTVFAVAADGQTAGEGASISLYTK(3)PSMVLNLKAEYVTMTSVNLTWMVNDAESASYTYRIEVAHESLINETMSNVTKS
IVTYLIPGTSYNFTVFAIAADNQTEGEGASISQNTV(2)PSSVNAFQCEAVANMSYLTLKWNCPYGGYSGFDIEIFNGTWTKKQ
QSQFCGREGSEEIFKTEPLDYYKTYTVSVTTVSDGLTSLPVQKICKTSITD(1)PPVPNKAPLVKAVSHNSLSVEFPDFESVNG
PLKAYAVMIVTEAEGCLPSKSDLDYTYNDFKQKMTATYVTYVIDVEEISSSSHSQNGHNIVDVGKGNTMYGYENGPLIPLHSYR
ASVAGFTNINFTVANKIMGEQSYVSFSPCSEVVSLPQDPGVIAGAVIGCLLAILAVVAIGGYIFWRRRRKDKRNTEVSFSPIKS
KMIKVENFESYFKKQQADSNCGFAEEYEELKSAGVHQPKFAAEIAENRGKNRYNNVLPYDISRVKLSNPSCTTDDYINANYMPG
YSSKKAFIAAQGPLPNTIEDFWRMIWEKNIYSIVMLTKCVEQARTKCEQYWPDKQSKSYGDIIVTMVSEVVLPEWTIRDFNVEN
ADTMESHTVRQFHFTSWPDHGVPETTDLLINFRHLVHEYSSQNPIDSPILVHCSAGVGRTGTFIAIDRLIQQIEMENTVDVYGV
VYDLRMHRPLMVQTEDQYVFLNQCVMDIIRSQKEKKTDLIYQNTTAMAIYENFTPGPAFGKANGYHA

>PTPRJ.fish   FN428723 NP_001129714.1| [Danio rerio]
MGQHYRLQAIVSRTTVLFVVLVSIHHGHCTDCSLCQYKTSTTTSEINVFVGNSSFCIAQNASFTNSSQTNITVTGLLPGNTYFL
IISCTSVCCENFSSSPAQVQNITVVNYTNSSVTVAWTVPDGHVDSYTVNCSTQSIPNINDNNTTISSLPPGCLYNLTVTSISGV
LTNVSNIVQFATK(12)PNPPEAVRVSGQTFSSISLLWSTPLSMSGVAVSYGVSYVPVQPGVNLPLTQTTSSNMTNITNLLSGT
QYTITVVTNGVNNLSSSFINLIGYTV(11)PKIVQNLAVSNVSTNSVSLTWLPPNGTSSLFSYNINISSLGQTFYTTSNSYQIT
QLQPGTQYNCYVTTLIVAGNISGPSQFIQCNTK(10)PLPVSNLKAIPVGTTVILLSWTPSTALYLYNVSANGTWSTYNSSEGA
NVTGLTPGNNYTFTVITVANGLSSEPVTISAFTG(9)LGKAIIISAIGNTQTMTVQWTPPAGTVSLYSARILLMDGTVLNTKNQ
TKTTSMMFSDLLPGTQYSVIVSSISGPVKQDSDSVTNATL(8)PTPPGEISTTQSTNTLNISWAQPLNMSSVSYYFLLQYNNST
LINCSQNYTSLTGLNAGFPYNIIMWTVGAMNYSSSSKSLTAFTN(7)PSVVTNVTVNEFTETSVTLSWQQNDPQQSGYSYLLNY
KTINGTSNQTTVNNNTVKLEQLQSASQYNISIIALTPGGTKSDPQFITACSR(6)PNRVNNVSSVALNVSTVRLSWARPLQYNQ
LVSYQVLVSNCTKNSMNMSTSTEMITVTNLQPGTLCQISIYSVACGLLGQPVNTSVLTM(5)PSTVQPVVNSQGSNNSLLVLWS
HPDGGLDMYILNISSDGWSDFSFLNSTENNYTFTQLKAATLYTVTLTTVKGSFQETSGAVVSATY(4)PNSPGMITVLFKNTHS
VLLHWDIAQNMTPGSFNYSLSFWSSKNNSLYLTPNNTLLLDGLQSGTPYNVSLATVGPLNLQSESMRYNVTTM(3)PDPVKNLK
VVSTTTNTISVTWKMVDVPKYVVSVNNAVKITSSNNMTIHNLQPSTQYNISVQSSTFDNTEGEAVCLQDCTD(2)AAPVENVTC
VGPNLTLPMLNLFWNHPLGGYMNFEVNLSSSISEKTKNLNYNFTGLKYNKTYSVTLRTLGCGKSSTVKNISCTTGFTN(1)PVV
PKITAASVSEQQYNKFALVIQSDAFNDSNGTVCSYGLLVSSGSFDCLDNNQNYNQCLLKTYDDWKAKQSDTFLAVVKTNVSKSE
LETIIIGDGSKWNKYTNGELNAKGTYNFAIVAFTLLEVKDGKVDVSKSYYSVSAYQSVTLPENPVVIGGAAAGVGVTVIIVVII
IGIVACTRKKRKEVSNSVPIHSLSDPIKVEDYEAYYKRQRADSFCGFAEEFEDLRPVGINQSKTVAVFPENKAKNRYNNVLPYD
SSRVKLSVLSSPFDDYINANYMPGYITKKEFIAAQGPLPGTVNDFWRLIWEKNVHTIVMLTKCNEQGRVKCEEYWPAEMKTFSN
LTVTTISDIPLEDWTLRDFEVKNMKTAEIRSVRHFHFTAWPDHGVPETTELLINFRHLVREHMDEYSRHSPTLVHCSAGVGRTG
TFIAIDRLIFQIERDGVVDVYGIIHDLRMHRPLMVQTEDQYVFLNQCAMDIIKSRTGNNVDLIYQNTAALTIYENFEPLKKGKN
GYHKA

PTPRO

>PTPRO.human ENSP00000281171 [Homo sapiens]
MGHLPTGIHGARRLLPLLWLFVLFKNATAFHVTVQDDNNIVVSLEASDVISPASVYVVKITGESKNYFFEFEEFNSTLPPPVIF
KASYHGLYYIITLVVVNGNVVTKPSRSITVLTKPLPVTSVSIYDYKPSPETGVLFEIHYPEKYNVFTRVNISYWEGKDFRTMLY
KDFFKGKTVFNHWLPGMCYSNITFQLVSEATFNKSTLVEYSGVSHEPKQHRTAPYPPQNISVRIVNLNKNNWEEQSGNFPEESF
MRSQDTIGKEKLFHFTEETPEIPSGNISSGWPDFNSSDYETTSQPYWWDSASAAPESEDEFVSVLPMEYENNSTLSETEKSTSG
SFSFFPVQMILTWLPPKPPTAFDGFHIHIEREENFTEYLMVDEEAHEFVAELKEPGKYKLSVTTFSSSGSCETRKSQSAKSLSF
YISPSGEWIEELTEKPQHVSVHVLSSTTALMSWTSSQENYNSTIVSVVSLTCQKQKESQRLEKQYCTQVNSSKPIIENLVPGAQ
YQVVIYLRKGPLIGPPSDPVTFAIV(4)PTGIKDLMLYPLGPTAVVLSWTRPYLGVFRKYVVEMFYFNPATMTSEWTTYYEIAA
TVSLTASVRIANLLPAWYYNFRVTMVTWGDPELSCCDSSTISFITA(3)PVAPEITSVEYFNSLLYISWTYGDDTTDLSHSRML
HWMVVAEGKKKIKKSVTRNVMTAILSLPPGDIYNLSVTACTERGSNTSMLRLVKLE(2)PAPPKSLFAVNKTQTSVTLLWVEEG
VADFFEVFCQQVGSSQKTKLQEPVAVSSHVVTISSLLPATAYNCSVTSFSHDSPSVPTFIAVSTMV(1)TEMNPNVVVISVLAI
LSTLLIGLLLVTLIILRKKHLQMARECGAGTFVNFASLERDGKLPYNWRRSIFAFLTLLPSCLWTDYLLAFYINPWSKNGLKKR
KLTNPVQLDDFDAYIKDMAKDSDYKFSLQFEELKLIGLDIPHFAADLPLNRCKNRYTNILPYDFSRVRLVSMNEEEGADYINAN
YIPGYNSPQEYIATQGPLPETRNDFWKMVLQQKSQIIVMLTQCNEKRRVKCDHYWPFTEEPIAYGDITVEMISEEEQDDWACRH
FRINYADEMQDVMHFNYTAWPDHGVPTANAAESILQFVHMVRQQATKSKGPMIIHCSAGVGRTGTFIALDRLLQHIRDHEFVDI
LGLVSEMRSYRMSMVQTEEQYIFIHQCVQLMWMKKKQQFCISDVIYENVSKS

>PTPRO.mouse Ptpro-201 ENSMUST00000077115 **[Mus musculus]**

MGHLPRGTLGGRRLLPLLGLFVLLKIVTTFHVAVQDDNNIVVSLEASDIVSPASVYVVRVAGESKNYFFEFEEFNSTLPPPVVF
KATYHGLYYIITLVVVNGNVVTKPSRSITVLTKPLPVTSVSIYDYKPSPETGVLFEIHYPEKYNVFSRVNISYWEGRDFRTMLY
KDFFKGKTVFNHWLPGLCYSNITFQLVSEATFNKSTLVEYSGVSHEPKQHRTAPYPPRNISVRFVNLNKNNWEEPSGSFPEDSF
IKPPQDSIGRDRRFHFPEETPETPPSNVSSGSPPSNVSSAWPDPNSTDYESTSQPFWWDSASAAPENEEDFVSALPADYDTETT
LDRTEKPTADPFSAFPVQMTLSWLPPKPPTAFDGFNILIEREENFTDYLTVDEEAHEFVAELKEPGKYKLSVTTFSSSGACETR
KSQSAKSLSFYISPTGEWIEELTEKPQHVSVHVLSSTTALMSWTSSQENYNSTIVSVVSLTCQKQKESQRLEKQYCTQVNSSKP
VIENLVPGAQYQVVMYLRKGPLIGPPSDPVTFAIV(4)PTGIKDLMLYPLGPTAVVLSWTRPILGVFRKYVVEMFYFNPTTMTS
EWTTYYEIAATVSLTASVRIASLLPAWYYNFRVTMVTWGDPELSCCDSSTISFITA(3)PVAPEITSVEYFNSLLYISWTYGDA
TTDLSHSRMLHWMVVAEGRKKIKKSVTRNVMTAILSLPPGDIYNLSVTACTERGSNTSLPRLVKLE(2)PAPPKSLFAVNKTQT
SVTLLWVEEGVADFFEVFCQQLGSGHNGKLQEPVAVSSHVVTISSLLPATAYNCSVTSFSHDTPSVPTFIAVSTMV(1)TEVNP
NVVVISVLAILSTLLIGLLLVTLVILRKKHLQMARECGAGTFVNFASLEREGKLPYSWRRSVFALLTLLPSCLWTDYLLAFYIN
PWSKNGLKKRKLTNPVQLDDFDSYIKDMAKDSDYKFSLQFEELKLIGLDIPHFAADLPLNRCKNRYTNILPYDFSRVRLVSMNE
EEGADYINANYIPGYNSPQEYIATQGPLPETRNDFWKMVLQQKSHIIVMLTQCNEKRRVKCDHYWPFTEEPIAYGDITVEMVSE
EEEEDWASRHFRINYADEAQDVMHFNYTAWPDHGVPPANAAESILQFVFTVRQQAAKSKGPMIIHCSAGVGRTGTFIALDRLLQ
HIRDHEFVDILGLVSEMRSYRMSMVQTEEQYIFIHQCVQLMWLRKKQQFCISDVIYENVSKS

>PTPRO.chicken PTPRO-201 ENSGALT00000037455 **[Gallus gallus]**

MLPQFTIAGHGSEPGGGRRGAAARGRGAEEPQPPPPGRSRGPRPPRSPMPDRRRRGARGGPGARSCLLWLFVLLKNAEPFQVIV
QEDNCIVVSLEAFDVVSSSSVYVVKIAGESKNYFFQFEEFNSTLPAPVIFNAKYHGLYYIVTLLVVNSNMVSKSARSITVLTKP
LPVSSVSIYDYKPSPETGVLFEIQYPEKYNVFTRVNISYWEGKDYRTMLYKDFFKGKTVFNHWLPGICYSNITFQLVSEATFNK
STLVEYSGVRHEPKQHRTVPYPPRNISVQIVPINRNNWEEHSGNFAEESFMGQQEIISKEKLSRFPSDPPEIPVVNTTAAWPDY
RSNSTEYETTSQPYWWSNEAESSEGEEEFVNAVPRDYGASEANASGKLTAEPAPFLPVQMVLTWLPPKPPTAFDGFHINIEREE
NSTELLTVSEDTHKFVAELKEPGKYKLSVTTFSSSGSCEVRGSQSAKTLSFYISPTGEWIEELTEKPQHVTVTVLSSTTALTSW
TASHESGSNGTIVSVVSLTCQKQKESQRLEKHYCTEVNSSSSLIENLVPGAQYQVVVYLRKGPLVGPPSDPVTFSIV(4)PTGI
KDLTLYPLGPTAVVLSWNRPFLGVFRKYVVEMFYFNPSTMTSEWTTYYEIAATISLTASVRIANLLPAWYYNFRVTMVTWGDPE
LSCCDSSTISFITA(3)PVAPEITSIEYYNHLLYVTWTYGGDGTDLSHSRMLHWMVIAEGKKKIKKSVTRSVMTAVLSLPPGDI
YNLSVTACTERGSNTSLPQLVKLE(2)PAPPKSLFAVNKTQTSVTLLWVEEGVADFFEVYCQQAGSNQEVKVQEPVTVSSHVVT
ISSLTPATSYNCSVTTFSHNSPSVPTYIAVSTMV(1)TEMNPNIVVISVLAILSILLIGLLLVTLVVLRRKHLQMARECGAGTF
VNFASLERDGKLPYNWRRSVFAFLTLLPSCLWTDYLLAFYINPWSKNGLKKRKLTNPVQLDDFDGYIKDMAKDSDYKFSLQFEE
LKLIGLDIPHFAADLPMNRCKNRYTNILPYDFSRVRLVSMNEEEGSDYINANYIPGYNSPQEYIATQGPLPETRNDFWKMVLQQ
KSQIIVMLTQCNEKRRVKCDHYWPFTEDPIAYGDITVEMLSEEEHTDWVYRNFRISYADEVQDVMHFNYTAWPDHGVPTANAAE
SILQFVQMVRQKSVKSKGPMIIHCSAGVGRTGTFIALDWLLQHIRDHEFVDILGLVSDMRSYRMSMVQTEEQYIFIHQCVQLMW
QKKKQQFCISDVIYENVSKS

>PTPRO.frog Xenopus Tropicalis ptpro-202 ENSXETT00000048747 NM_001113813.1 PARTIAL
Xenopus tropicalis

TFQVALTQDNCLVISLESPDVLSTKSVYLVKITGESKNYFFQFEEFNSTLPSPLVFNATYHGLYYIITLIAVNSNMASRSIKSI
TVLTKPLPVSHVVIHDYRPSPQTGVVFEVQYPEKFNVFSRVNISYWEGSHFRTVLYKDFFKGKTVFNHWLPGTCYGNITLQLVS
EASFNKSTLVEYSGVGHRPQFHRTVPFPPRNISVQILRLNLSFTDLLNGKNNEDSLKKYQDFTEPESSAEMFEDQNERSTFPSL
YNSTDPQYKNNTLPEATSQPFWWANETLPPDTDGFVNGVLPDYEDPPVGSSVPNSSSLSFKLLISWLPPKAPTAYDGFNIYIFR
DGNASAVTAVDESAHEFVAEMREPGKYWVEITSFSSTGSCEIRESSAAKAISFYIKLLASAGELTPWGPRSTEPYKLLLYSDVI
IIIILGLYELHKSHIQISPSCSLTCPKQKESQRLERHYCKEVNSPKPVDHIETPNRVLQKVGYLRVTPRKVAGQISDPPHFLVN
PPGVTDLLLYPLGPSAVVLGWRRPYLGAFRKYMVEMFYFNPAAMSSEWTPYYEVAATVSLTASVVPFAPRVPCAPHVPFAPRVP
FAPRVPCAPHVPFAPRVPCAPHVPFAPRVPCAPHVPCALXEVRGDINISRRILYTALFPSGSHEMLQGVSRSGTATVISVPPGD
IYNITVTACTERSSNTCAFTLLQSDPAPIRTLFAVNKTQTSVTLLWVEEGIADYFEVSCWQNREPGAETNQKDPVAVFSHIVTI
SSLQPDTSYNCSVTSISHRTP**(1)**SAPTFLTVSTLVAELNPNVVVISVLALLSVLLIALLFLMLIVLRRKHLQMSRDCGAGTFV
NFASLEKDGKLPYNWRRSIFAFLTVLPSCLWTDYLLAFYINPWNKNGLKKRKLTPVQLD**DFDLYIKEMSKDSDYKFSLQFEELK**
MVGLDIPHFAADLPSNRGKNRYTNILPYDCSRVKLISLEADEGADYINANYIPGYSAAQEYIATQGPLPETRNDFWKMILQQKC
QVIVMLTQCNEKRRIKCDHYWPFTPEPVNYGDITVEMASEEEQSDWAQRVFRVSYADETQCVTHFNFTAWPDHGVPTVNAAESV
LQFVQVVRQKALKSKGPVTVHCSAGVGRTGTFIALDWLMQHIRDHEFVDILGLVSELRSHRMSMVQTEEQFVFIHQCAQLMWKK
KRQQFRISDVIYENVSKS

>PTPRO Xenopus laevis gi|1050385751|gb|OCT87944.1| hypothetical protein
XELAEV_18016573mg []

MPPPAAQPVSHPSRQTRDCDISECPPRRSRISECDREPGRRRRRRRPARISVRPGGTERQDPSLHFPFNPRGQRAAADPLQRLR
PPRPARVMILLTCLLLFIFLQDGSTFQVAVTDDNCLVISLEPSDVLNTKSVYLVKITGESKNYFFQFEEFNSTLPSPLVFNASY
HGLYYILTLIAVNSKMTSRSSKSITVLTKPLPVSHVTIHDYKPSPQTGVVFQVQFPEKFNVFSRVNISYWEGRHFRTVLYKDFF
KDKTVFNHWLPGTCYSNITLQLVSEASFNRSTLVEYSGVGHQPQSHRTVPFPPRNISVQILPLNHSITDLLNGKNNEVTVTKYQ
DFPESSAEMFDDQNERSTFIQLYNLTDPQYKNNTFPEATAQPFWWASETIPPDTEGFVNGMLPDYEDPPVRSSEPPSPSPNFKL
LISWLPPKAPTAYDGFNIYIFRDGNASVVSSVDESTHEYVAELVEPGKYFVEITSFSSTGSCEIRESSPAKAISFYISPTGDWF
EEMTEKPRDVAVKIVNSTNALVSWASSPDISPIVSVISLTCSKQKESQRLEKHYCKEVNVSSNIIGNLVPGAQYKVVVYFQRPP
LIG**(4)**PPSDAVLFAIDPTGIKDLVLFPLGPSAVILSWSRPYLGVFRKYIVEMFYFNPVAMSSEWTPYYEIAATVSLTASVKVT
DLLPAWYYKFRVTMATWGEP**(3)**QLSCCDTTVSFVTAPVAPQITSVEYSYGLLHVTWAYADGSFDLSHSRIVHWQVIAEGKKRI
KKSVSRSGMATVLSVPAGDIYNITVTAFTERSS**(2)**NTSLPHLVKLDPAPIKTLFAVNKTQTSVTLLWVEEGITDYFQVSCWHN
VETGTNKNDKEPVTVFSHVVTISSLEPATSYNCSVTSISHRTSS**(1)**APTFITVSTLVTELNPNVVVISVLALLSILLIALLLL
MLIVLRRKHLQTSRDCGAGTFVNFATLEKDGKLPYNWRRSIFAFLTVLPSCLWTDYLLAFYINPWNKTGLKKRKLTNPVQLDDF
DSYIKEMSKDSDY**RFSLQFEELKMVGLDIPHFAANLPSNRGKNRYTNILPYDFSRVKLISLDADEGADYINANYIPGYSAVQEY**
IATQGPLPETRNDFWKMILQQKCQVIVMLTQCNEKRRIKCDHYWPFTSEPVNYGDITVEMVSEEEQSDWAQRVFRVSYGDESQC
VTHFNFTAWPDHGVPTVNAAESVLHFVHVVRQKALKTKGPITVHCSAGVGRTGTFIALDCLMQHIRDHEFVDILGLVSELRSYR
MSMVQTEEQFVFIHQCVQLMWKKKRQQFRISDVIYENVSKS

>PTPRO.fish zebrafish ptpro-204 ptpro-204 ENSDART00000122021 Danio rerio

MSSARARFFFIIFIFIQSAAGFRVQLQDGVRMLMSLDDGDIGPGVSEYAVRVSGEPLTHTLLFQQAADAHTLPEPLLFNASYHG
LLYSISLMTDGRTHTTRSIITQPLPLDSVEMWDYAPSPETAVVFQIRSPDRNIFTRVNISYTEGHQRRYMLYKDFLHGKTVFKH
WLSGVCYSNITFQLISEASVNRSALLSRSDITHNPQQHRTVPNPPLNVSLKILHLSGRGPPGPVLTGAILKNSHNASVVRRERD
LPEYEEQEPTSDTLNTESNLTHTQLNQSESENESESEPVTAEPTLNSSTQSLWAWQTVSPAPTEEEEEGFVNALVPEYEDSNEP
GSALGIPLEPAVMPAVMPTPLAPVLLQLRWSPPAPHTAYDAFNIYIYRNGNSTETATVDENTHEFLAELSESGTYRIHVTTLSS
AGDCEARESSANTAFTFYLSPSGEWMEQPQERPQAVSVKMLDSSTAAVSWAPSTHTYNGSLISVQSLTCLRPSISQRMELNYCS
EENITSDIISSLTPGAQYRVVVYHTNGPLVSPASEPVIIDIE**(4)**PTGVRDLVVYPLSPSAVILSWQRPYNVAFRKYVLQTFFF
NSATQTAQWSTYYEIAATASVIASVRVTDLLPAWFYNFRVTMVTWGDPPLSCCDTSTVSFITA**(3)**PEAPHISSVEFSHGSVFV
RWTYGDLFTDLTHSRMLHWQVVAEGKKSARRRFSVDVTRSVMKASLALPAGDIYNLTVTACTERSCNTSAPHIMKLD**(2)**PAPP
RSLYAVNASDTSVTLIWAEEGVVDHYLITCRALGAHAEQKKVREPLVTSAHVLTVSGLQASTTYNCSVRSSSYSSSSDPVHITV
STTV**(1)**REMNPSVAAISALAVLSVLLISLLVLFLLVLRKKHMELSRECGAETFVNFASFERDGKLPYNWRRSLFAFLTLLPSC
LWTDYLLAFYINPWSKTALKKRKLTSPVQL**SDFEAYLKDMGKDSAYKFSLQFEELKSVGLDLSHEAADLPINRPKNRYTNILPY**
DFSRVKLISLHNDEGSDYINANYIPGYNSPREYIATQGPLPDTRNDFWKMVLQQKVHIIVMLTQCNERRRVKCDHYWPFSDEPV
AYGEISVEMLAETDSPEWTIRSFRLAYADETQDVLHFNYTSWPDHGVPTVNAIESILQFVQIVRQQVNRSKGPIVVHCSAGVGR
TGTFISLDRLMQHIQEHEYVDVLGLVSDMRSHRLSMVQTEEQYVFIHQCVLLMWKKKKQQSHTSDVIYENVSKS

PTPRH

>PTPRH.human ENSP00000365528 [Homo sapiens]
MAGAGGGLGVWGNLVLLGLCSWTGARAPAPNPGRNLTVETQTTSSISLSWEVPDGLDSQNSNYWVQCTGDGGTTETRNTTATNV
TVDGLGPGSLYTCSVWVEKDGVNSSVGTVTTATA**(8)**PNPVRNLRVEAQTNSSIALTWEVPDGPDPQNSTYGVEYTGDGGRAGT
RSTAHTNITVDGLEPGCLYAFSMWVGKNGINSSRETRNATTA**(7)**HNPVRNLRVEAQTTSSISLSWEVPDGTDPQNSTYCVQCT
GDGGRTETRNTTDTRVTVDGLGPGSLYTCSVWVEKDGVNSSVEIVTSATA**(6)**PNPVRNLTVEAQTNSSIALTWEVPDGPDPQN
STYGVEYTGDGGRAGTRSTAHTNITVDRLEPGCLYVFSVWVGKNGINSSRETRNATTA**(5)**PNPVRNLHMETQTNSSIALCWEV
PDGPYPQDYTYWVEYTGDGGGTETRNTTNTSVTAERLEPGTLYTFSVWAEKNGARGSRQNVSISTV**(4)**PNAVTSLSKQDWTNS
TIALRWTAPQGPGQSSYSYWVSWVREGMTDPRTQSTSGTDITLKELEAGSLYHLTVWAERNEVRGYNSTLTAATA**(3)**PNEVTD

LQNETQTKNSVMLWWKAPGDPHSQLYVYWVQWASKGHPRRGQDPQANWVNQTSRTNETWYKVEALEPGTLYNFTVWAERNDVAS
STQSLCASTY(2)PDTVTITSCVSTSAGYGVNLIWSCPQGGYEAFELEVGGQRGSQDRSSCGEAVSVLGLGPARSYPATITTIW
DGMKVVSHSVVCHTESAG(1)VIAGAFVGILLFLILVGLLIFFLKRRNKKKQQKPELRDLVFSSPGDIPAEDFADHVRKNERDS
NCGFADKYQQLSLVGHSQSQMVASASENNAKNRYRNVLPYDWSRVPLKPIHEEPGSDYINASFMPGLWSPQEFIATQGPLPQTV
GDFWRLVWEQQSHTLVMLTNCMEAGRVKCEHYWPLDSQPCTHGHLRVTLVGEEVMENWTVRELLLLQVEEQKTLSVRQFHYQAW
PDHGVPSSPDTLLAFWRMLRQWLDQTMEGGPPIVHCSAGVGRTGTLIALDVLLRQLQSEGLLGPFSFVRKMRESRPLMVQTEAQ
YVFLHQCILRFLQQSAQAPAEKEVPYEDVENLIYENVAAIQAHKLEV

>PTPRH.mousePtprh-202 ENSMUST00000166650 [Mus musculus]

MARAGGNCGVWRSLVLLGLYGCSVVRAAGTSVTVDRHAPASSYEFSMWVEKDGVSSSPQIPVTTAAPNPVRNLRVEGQNNISIS
LSWEPPDQSSLQGLTYWTQCSRHGGQTETRNTTDTSVTVDGLDPGSSYECSVWVEKDGLYSKNETLNTSTA(6)PNPVRNLRVE
GQNNISISLSWEPPDQPSLQGLTYWAQCSRHGGQTETRNTADTSVTVDGLDPGSSYECSVWVEKDGVYSTNETLNTSTA(5)PN
PVRNLRVEGQNNISISLSWEPPDQPSLQGLTYWAQCSRHGGQTETRNTTDTSITVDGLDPGSSYECSVWVEKDGVYSTNETLSN
TTA(4)PNPVRNLRVKSQNNFSISLSWEPPDQPSLQGLIYWAQCSRHGGQTETRNTTDTSVTVDGLDPGFLYKCSVWVEKDGVY
STNETLNTSTV(3)PISASNPVRNLRVEGQNNFSISLSWEPPDQSSLQGLTYWAQCSRHGGQTETRNTADTSVTVDGLDPGSSY
ECSVWVEKDGVYSTNETLNTSTV(2)PAAVNITSCISTSGGYGVLLTWSCPSGGYESFEVKVGRKWRSENGSLCGKGVTVSDLE
PAQSYTATVTTVFKDLKAQSLSTTCHTESAA(1)IIAGAIVGILLLFILVGLLIVFLKRRRKKRQPKEVPKDLVCSCPGDILAK
DFADHVRENEKDSNCGFAEEYQQLALEGQGQSQITASALENRSKNRYRNVLPYDWSRVPLQPLQEEPGSDYINASFMPGLWSPK
EFIATQGPLPNTVGDFWRMVWEQQSHTLVMLTNCMESGRVKCEHYWPLDAQPCIHGQLQVMLISEEASENWTVRHLQLFHMKEQ
QTLSLRQFHYLAWPDHGVPYSPDPLLAFRKMLRQWMDQTTDGGPPIVHCSAGVGRTGTLIALDVLLRQLECEGLVGPFSFVKKM
RESRPLMVQTEAQYVFLHQCILKSLQKPAPALVPEEAMYENVASLVYENASAIMAHESEFSASGC

>PTPRH.frog xenopus XP_004916623.1| [Xenopus (Silurana) tropicalis]
MEYDEALTGICNVQILLKAMRTGCYVTLGYVPIRSHRFQKRLLSGVPSPVQSVSVSNITTTNVTLSWVPPNDINKDTYTYSITV
YNGSSPWDWPKNISGTSTQVTGLIPGVFYTFSVFTVTSNGTMSSQAGSTSGTTVPSPVQSVSISNIMTTNVTLSWVPPNDINKD
TYTYSITVYNGSTSWNGPTNISGTSTQVTGLIPGVYYTFSVFTVTSNGTKSSQAGSTSGTTV(7)PSPVQSVSISNIMTTNVTL
SWVPPNDINKDTYTYSITVSNGSSPWNGPKNISGTSTLVTGLIPGVFYTFSVFTVTSNGTMSSQNGSTSGTTG(6)PSLVTTVS
IIKQTNNSVTISWTPPQDINQATYMYSINVTNSAGSWNVNSSTNVSSKEVLDLLPGVTYTFCVYTVTSDGTKSSDCSPDSSTTR
(5)PTLVKDVTVKSQTTDSVTLSWTTPNDINKGSYYYLITTACGTTTCNVNSTVNANGATVASLLPGLTYQFSVYTVTSDGTNS
SAFASTSGTTA(4)PNAPTSVSGNPVNTTALSVNWTPPNDLNKNNYTYTVFWQRNNESLNQSITSNTTIIGGLTAGNQYNVSVI
SVINNVPSVKAVAYLQTN(3)PEVPGGFKIVTITNSSVNVSWTIPSGGSFSGIEINAVSGTSNLTKQFTGPSSEGILDGLIPGT
AYNLILRSFSSYPSGSSGRISRRATTNNIITYSNPIIEKVQLD(2)PNTVTGITCTKVGGGYQLKVAFVCPSGSYSSIKILADG
NEKETIPAQNCSQGATVQSLQPATKYIITVETVADSRQVSDFIICYTDNV(1)GVIVGSIFGVLLFLLVVGLIAYFVLRKRRGS
KNPFKKERGNMQKINIVRMPSVMKSAFPDYYQRQHADSDFGFAEEYQQLSNVGINQSKLAAELSENRSKNRFTNVLPYDHSRVR
LNRIDADETSDYINANYMPGYNSSKEFIASQGPLPNTSADFWRMIWENQVSTIVMLTNCMENGRVKCEHYWPLDYTPCTYGDIT
VTVTSEMILPDWTVRDFTLKHAKQQGNKHARHFHFTVWPDHGVPENTTTIVEFRNLVREYMDLKRSSGPTVVHCSAGVGRTGTL
IALDYLIQKMEKEQRIGIYSFVQKMRQNRPLMVQTESQYVFLNKCMLDLIQNPPDENIYENQIGGDLIYENAS

>PTPRH.fish ptprh-202 ENSDART00000114768 zebrafish Danio rerio

MELVLAFALLCVLSVSGQATLPDVNDVRITHRTETELTFQWDKAGGSNSYSYILRRDGIDSDIITAGTVNQVTYKVSDLSSGTN
YNWTLFTVFGDQRSEGKNFSAATVPPNVESVSVIDQSETTITLQWKKDKAEYSYNLTYGDKVDTPPSPQSTVEYTVTSLSPGTN
YSFTLYTVFEGLKSSGFSFFAVTV(5)PSKITGFSVIGRSETALTFQWTKEKDEYTYELDKSDANTQISFTDTTVVCLITSLSP
GTEYSYNLYAVFEGVKSQAKGISEVTV(4)PSNVENVSVVEANDTDVILQWNAVPNEQNKYNYILKYNDVEESVDFTTENEIKH
HVSGLIPATNYNFTLHTEFYSQNSTGYNFNHTTF(3)LSSVTEVRVNRSLTELTVEWNKLNQNNVYNYTLLRSDGAEIDFTGSA
VVGDVIKHKYSSLKPGTVYSFTLFTVSNNVRSSEHSFKSITA(2)LDCASFNWKVTNTSIVADVYGCTYVTAQNSSGSGKNASI
EGDKVNLRDLYPGESYTVSLFYNLESKMLTQCSQRLTLVPYVVSNLKCEYHSGGYGLAVMWDYPDGVVDVVEVDVNKKSFNHSH
GSDEPTQQLVTGLQAAQWYKVKATSFSGARRSETILINCQTDPAG(1)VIAGVLVFFLLVIIICAGVFVWFRYKSARNNGPDAS
ADLKVSKKNYKTIACDKFPEHFRNMSRDDNRAFSAEYDDLSSVGVEQSKVAALLPENKDKNRFSNVLPYDTSRVHLTINKAGDS
DYINANYMPGYGNASREYIAAQGPLPSTVNDFWRMIWEKKSSTIVMVTNCTEGGRVKCEQYWPLDYTPCLYENLLVTVKSENKS
QSWTLREFNVKNKMTSETRTVRHFHFTAWPDHGVPRGTEELIQFRDLIRQHIESHFSTGPTVVHCSAGVGRTGTLIALDVLLQQ
LNREKAVGVAAFVQEMRLNRPLMVQTESQYVFLHQCILDSLQTKFVQQSEPIYQNTDTIYVNAMALKDFENSSHT

PTPRG

>PTPRG.Hs ENSG00000144724

MRRLLEPCWWILFLKITSSVLHYVVCFPALTEGYVGALHENRHGSAVQIRRRKASGDPYWAYSGAYGPEHWVTSSVSCGGRHQS
PIDILDQYARVGEEYQELQLDGFDNESSNKTWMKNTGKTVAILLKDDYFVSGAGLPGRFKAEKVEFHWGHSNGSAGSEHSINGR

RFPVEMQIFFYNPDDFDSFQTAISENRIIGAMAIFFQVSPRDNSALDPIIHGLKGVVHHEKETFLDPFVLRDLLPASLGSYYRY
TGSLTTPPCSEIVEWIVFRRPVPISYHQLEAFYSIFTTEQQDHVKSVEYLRNNFRPQQRLHDRVVSKSAVRDSWNHDMTDFLEN
PLGTEASKVCSSPPIHMKVQPLNQTALQVSWSQPETIYHPPIMNYMISYSWTKNEDEKEKTFTKDSDKDLKATISHVSPDSLYL
FRVQAVCRNDMRSDFSQTMLFQANTTRIFQGTRIVKTGVPTASPASSADMAPISSGSSTWTSSGIPFSFVSMATGMGPSSSGSQ
ATVASVVTSTLLAGLGFGGGGISSFPSTVWPTRLPTAASASKQAARPVLATTEALASPGPDGDSSPTKDGEGTEEGEKDEKSES
EDGEREHEEDGEKDSEKKEKSGVTHAAEERNQTEPSPTPSSPNRTAEGGHQTIPGHEQDHTAVPTDQTGGRRDAGPGLDPDMVT
STQVPPTATEEQYAGSDPKRPEMPSKKPMSRGDRFSEDSRFITVNPAEKNTSGMISRPAPGRMEWIIPLIVVSALTFVCLILLI
AVLVYWRGCNKIKSKGFPRRFREVPSSGERGEKGSRKCFQTAHFYVEDSSSPRVVPNESIPIIPIPDDMEAIPVK**QFVKHIGEL**
YSNNQHGFSEDFEEVQRCTADMNITAEHSNHPENKHKNRYINILAYDHSRVKLRPLPGKDSKHSDYINANYVDGYNKAKAYIAT
QGPLKSTFEDFWRMIWEQNTGIIVMITNLVEKGRRKCDQYWPTENSEEYGNIIVTLKSTKIHACYTVRRFSIRNTKVKKGQKGN
PKGRQNERVVIQYHYTQWPDMGVPEYALPVLTFVRRSSAARMPETGPVLVHCSAGVGRTGTYIVIDSMLQQIKDKSTVNVLGFL
KHIRTQRNYLVQTEEQYIFIHDALLEAILGKETEVSSNQLHSYVNSILIPGVGGKTRLEKQFKLVTQCNAKYVECFSAQKECNK
EKNRNSSVVPSERARVGLAPLPGMKGTDYINASYIMGYYRSNEFIITQHPLPHTTKDFWRMIWDHNAQIIVMLPDNQSLAEDEF
VYWPSREESMNCEAFTVTLISKDRLCLSNEEQIIIHDFILEATQDDYVLEVRHFQCPKWPNPDAPISSTFELINVIKEEALTRD
GPTIVHDEYGAVSAGMLCALTTLSQQLENENAVDVFQVAKMINLMRPGVFTDIEQYQFIYKAMLSLVSTKENGNGPMTVDKNGA
VLIADESDPAESMESLV

PTPRF

>PTPRF.Hs ENSG00000142949

MAPEPAPGRTMVPLVPALVMLGLVAGAHGDSKPVFIKVPEDQTGLSGGVASFVCQATGEPKPRITWMKKGKKVSSQRFEVIEFD
DGAGSVLRIQPLRVQRDEAIYECTATNSLGEINTSAKLSVLEEEQLPPGFPSIDMGPQLKVVEKARTATMLCAAGGNPDPEISW
FKDFLPVDPATSNGRIKQLRSGALQIESSEESDQGKYECVATNSAGTRYSAPANLYVRVRRVAPRFSIPPSSQEVMPGGSVNLT
CVAVGAPMPYVKWMMGAEELTKEDEMPVGRNVLELSNVVRSANYTCVAISSLGMIEATAQVTVKALPKPPIDLVVTETTATSVT
LTWDSGNSEPVTYYGIQYRAAGTEGPFQEVDGVATTRYSIGGLSPFSEYAFRVLAVNSIGRGPPSEAVRARTGEQAPSSPPRRV
QARMLSASTMLVQWEPPEEPNGLVRGYRVYYTPDSRRPPNAWHKHNTDAGLLTTVGSLLPGITYSLRVLAFTAVGDGPPSPTIQ
VKTQQGVPAQPADFQAEVESDTRIQLSWLLPPQERIIMYELVYWAAEDEDQQHKVTFDPTSSYTLEDLKPDTLYRFQLAARSDM
GVGVFTPTIEARTAQSTPSAPPQKVMCVSMGSTTVRVSWVPPPADSRNGVITQYSVAYEAVDGEDRGRHVVDGISREHSSWDLV
GLEKWTEYRVWVRAHTDVGPGPESSPVLVRTDEDVPSGPPRKVEVEPLNSTAVHVYWKLPVPSKQHGQIRGYQVTYVRLENGEP
RGLPIIQDVMLAEAQWRPEESEDYETTISGLTPETTYSVTVAAYTTKGDGARSKPKIVTTTGAVPGRPTMMISTTAMNTALLQW
HPPKELPGELLGYRLQYCRADEARPNTIDFGKDDQHFTVTGLHKGTTYIFRLAAKNRAGLGEEFEKEIRTPEDLPSGFPQNLHV
TGLTTSTTELAWDPPVLAERNGRIISYTVVFRDINSQQELQNITTDTRFTLTGLKPDTTYDIKVRAWTSKGSGPLSPSIQSRTM
PVEQVFAKNFRVAAAMKTSVLLSWEVPDSYKSAVPFKILYNGQSVEVDGHSMRKLIADLQPNTEYSFVLMNRGSSAGGLQHLVS
IRTAPDLLPHKPLPASAYIEDGRFDLSMPHVQDPSLVRWFYIVVVPIDRVGGSMLTPRWSTPEELELDELLEAIEQGGEEQRRR
RRQAERLKPYVAAQLDVLPETFTLGDKKNYRGFYNRPLSPDLSYQCFVLASLKEPMDQKRYASSPYSDEIVVQVTPAQQQEEPE
MLWVTGPVLAVILIILIVIAILLFKRKRTHSPSSKDEQSIGLKDSLLAHSSDPVEMRRLNYQTPGMRDHPPIP**ITDLADNIERL**
KANDGLKFSQEYESIDPGQQFTWENSNLEVNKPKNRYANVIAYDHSRVILTSIDGVPGSDYINANYIDGYRKQNAYIATQGPLP
ETMGDFWRMVWEQRTATVVMMTRLEEKSRVKCDQYWPARGTETCGLIQVTLLDTVELATYTVRTFALHKSGSSEKRELRQFQFM
AWPDHGVPEYPTPILAFLRRVKACNPLDAGPMVVHCSAGVGRTGCFIVIDAMLERMKHEKTVDIYGHVTCMRSQRNYMVQTEDQ
YVFIHEALLEAATCGHTEVPARNLYAHIQKLGQVPPGESVTAMELEFKLLASSKAHTSRFISANLPCNKFKNRLVNIMPYELTR
VCLQPIRGVEGSDYINASFLDGYRQQKAYIATQGPLAESTEDFWRMLWEHNSTIIVMLTKLREMGREKCHQYWPAERSARYQYF
VVDPMAEYNMPQYILREFKVTDARDGQSRTIRQFQFTDWPEQGVPKTGEGFIDFIGQVHKTKEQFGQDGPITVHCSAGVGRTGV
FITLSIVLERMRYEGVVDMFQTVKTLRTQRPAMVQTEDQYQLCYRAALEYLGSFDHYAT

INVERTEBRATES

DEUTEROSTOMES

Tunicates

>Ciona   Ciona Savigny ENSCSAVT00000019606 plus GENSACAN and profiling profiling

MIKQLVILLFFRFLIHFRTLNKSIAESSTFSLEGQLQLDSLARTTTLLNLEPHRHYVLRIITISNHTTPRGWVTSESESTSLSF
STRKLDPDIKYRLPFITISPALAPPRSLHVNHTSITEESFEIAWKSAVGLVEKYRIVLKTKSADETKILVANNFATSYFVDGLS

PGESYEIAMISLKGIDQSSAVFVSQTTRAISDMAPPVNFRMTSRGETEITLGWDPAPGVQGVYMVRCEGFQEPCPAEVDIIRPD
EKRELRVGRLKPGARYNFTIQTQNGQDISKAKRLSLITV(15)PRTPLSLDTVSRSDTTISLRWDQPTGILDGYKISYTSRGGQ
LEILFVQPGIFEKTLNNLRPGTYYEISLVSTSSEIESEPIILTEITE(14)LREIENLSIARLGETWAFISWVPGEGGYSGFDY
IVQVATVLDKDRSVARNITIQTRPLPPNDVRMDNLTKSSVHVWWREPETFDQFLLVYKDDFNTKNLMTQQTSVDINDLTPGSSY
VIDICVIFGSVKSYNVTNEITTY(13)IIAEPGTPTALVLKPVSTTELLTEWEIPLNPNGIIRRYIIRFKQNYPHPSTNYTLIE
TNQTRYLLGNLEPGAEYVALFAAVNSAGVGNFTESYFFKMPESKPG(12)PVSDLHVSSVNAISATVAWQPPEQPNGEIIRYVL
NISTDHLPVLSVDLLKRSNSDKNLCLFDHKSKSDKTALVSVTSSLQNNSVTEALNDEIYFDPLCPVFVNVTSLLPFTLYRFRVT
AWTSSGEGDSRIREEMTLEDVPDDPPRHVRLTSATHTSLNVTWFPPNQPNGIVTYTIAVLQSDKMFQSNLTYYDINELRPFTKY
GVMVRAETSVGVSPWSEILFADTQQGRPGSVQNLTAQVETSRSVTLQWNPPINPNGVITGYWIFAKVKNVTQLTLWLEGVSL(1
1)NSGVNLRTLQDVPSSPPINLSYYNVSSTAVNITWDEPLEANGVIIKYVVYYMTKDTFLDVTVTERFALIKDLKIFSPYEVI(
10)RVRPYTLLGDGPASGTLLIHTDEDFPASPVRNVTSKNITSSVVELSWTPPLIINGRARSYTVYTVVLLNSYNAFNSYSNKT
SAFLTNLRVFTAYKVQVACQTKKGLGNILSKTIFIQTEEGQPATPPFNVSYQNLTSTKVRLTWRRPLVPNGIIQFYEISLTSKN
NKTIRATTENDVTAVTVDHLTAYSEYTATVRANTKFGDGSQQSHALTIHTLEDVPGSPVTNVTYVNLTSSSIGLEWSLPKEPNG
KILKYSIRYSMLEGNMNQYKAVNDTFVELFDLNKYTRYNVSVSAHTVAGEGPPAYISLHTDEDE(9)PESAPYDIIFQQYNSTT
IALTWRPPVKPNGIIVNYTVYYSNEDKVMTKTTTKPRTVLRNLEKFTEYEVYLTASTKLGNGGKISAIETFQTLEDA(8)PADP
PRDVVVKALSSTSISVGWSTPATPNGQIQFYTVFYTDKISAVHATNVTTVISMNRSTSSSKILLQITNGTNAVIKDLNKYSDYL
VWVSGSTALGDGNQLSDAKKVRTMEDV(7)PDNGVSDLSALAINATTIKVTWQPGIPLTGPTFFHIQYVLNNTPVHDLNFISFQ
PHTEYVIKVYPRVVGVYDAGPVSSIVVTTMESAPSSPVQNLHVVYVTETIVKLKWKPPSEANGVITSYTITVTKQNYNKTKTG(
6)NETETVIEDLRANSHYNVSVTSSTKIGEGPSTILLFRTKEGVPQDAVTDIQLINLTSDSALIKWLAPRQPNGVITHYTVHYG
RNSTIQTNVTEATLRDLHPFQLYWIEVYPWTSAGIGVKPSEKMAIKTKESA(5)PTAPTSLNCSVASNFSVKISWDLPMRTNGI
IRGYNLEYRSMDKNKVKFVQPSQTKQIISNLKPFANYLIKLRAINLAGKGQWTHCNVTTLEGY(4)ADAPPQLQLKNLSDRSIT
IEWSRPLQWNGRFHGYLITYKPPDSCPNPANDTQVCSFTSNLTSVTLEALSKYRAYNISVGSITGRGVGTMRDIFVSTLVGV(3
)PDSPVRDVHVTVVSSTSINVTWNLPSSYAGPTTYKLYSELVPLYDYFDHTAQYLRVHNHENNSPFSV(2)GLDEDTTYGVIVT
TMTTVGELSSIPVSIDTKEDAPSDFPESVGVATVLNNSSSVHVLFKEPLDPNGKLINYTIQHRRLEDSKLQSIVVSVDQLTAPT
TEHEPYSVSVSGLLGGRYYQFRVRAATAVGNGPWSLWTNKVLLPTTA(1)PPVPDTWPEVMNKIDDTSTMASVTSSTITVKKPC
MFSNENGPIASLSVIIAEDGGNIEAEPTYWSQAFPLQPSPPYKVLVSENPTDYCNTRKRRSINEKGFVIGTSDCPSELNTHCNG
PLKSSTDYRVKFRAETSNGLKTDTEFSEIIRTSPSFLATHMTLLIGIGAAIGLFTILVVISAVFLRLVMQYSIQYGFLNNVFPI
METSIHTNMAFIGEASMDDKVQEESVHGNEFSRPVFRDEFPNHVSSLSQNKNKGFSLEFDDIRGIPYAGTTAIAENSCNKTKNR
TTKLVPFDHCRVKIEGIPAIQGSNYINASYIPGLDSPEQYIATQTPLDHTKKDFWRMLWETGSTNIVMLCGSVNAGKKRCDEFW
PKKQTEYFGNLAVQMKEEIRHDEWIIRQFIVTMRDKVRHVTQHHFIKWPQLEQAENSLPLVRFIKNYRLLRDRSINPTIVMCSN
GSGRCGVFIGLLRILDNNGRDVDVFGTVAALRKYRPYMVQTLSEYIYLHQCVLKFIDD

>Ciona.1 *Ciona Intestinalis* genscan and curated by profiling gi|459174018
|ref|XP_002122888.2|] ENSCINT00000033578

MPSLYVISSYEISLTLATSGTAVTKTYTVIAPALTYTVTGLTPGESYTATVQAVSSSVSGSVSTPSLPQRTDVFENQVSSSVVS
TVSNLVDVPAEELAVSGEADSLLMTLDTVGEKVELDENGVYEDVTSNVGMAALLPSNKKGRSGLGVESLSINSEPGFRSDQVYL
IQGEIPNNVQSFITLPEEAGMLWVSCNMQIINACLFSVFGDSGNAKRIIFYAFPNDKLFLSPNNTYYDVNSMVVLSATVEQTSV
NNLQEPVIIQLVTNTHAVDAPSGITVDNIKSTEFTVSWTTPTDAISKMTVDIAGTASNENDAVDVVSPHKVINTTAGAAIVGNT
EYTITVYAVSSTDATDFKFATNQTTTIFSAPVLTSVTGTNSTIAVDLSWTYDNGGGANAVSEYLIKWDGGGSTGSPTAGSGSTT
ATISSLSANTEYTFSITAVSATVRGDTSAPSSATTAGATTTSIDLTWTAPAVGGGKNNVLAYTIQWTGGAGGSKETSGTTDTIS
SLSANTAYSFTVAAKSKAGTGEASTPLQAITLPSLPEQPTLTRSTTNPTTVIDVSWPAVTSGTETVDYVVEWTPDEGPAANKAV
TGTTTTIDSLVPGQQYNVTVRAHNSAGNSEPSPAARLRTN(11)PPTPTGVSLSNPTNGQTSLKVDWQITKNNFVISSYEISLT
PATSGAAVTKTYPANANSPFTYIVTGLTPGESYTATVQAFSSGVSGSISSASNSQTTV(10)PPTPTGVTLSQSAGDQTTSLKV
DWVMPSLYVVSSYNISLIPESSGSAIVTQTFTPSPASTTTDTVTGLTPGESYTATVQAFSSGVSGNNSDASASLRTV(9)SAVP
GTPNLYQPTDGSDKTTILYANWTVPTGVVDSYQLLVYLGSVGGTLVANQTVSTNFANITSLIPGKRYQATVRAFSAGVAGGVSG
NSNFAKTN(8)PPTPTGVTLSQPNVNQTTSLKVDWVMIPTSFVISSYIISLISTTSGAVVNKTYTASAGSAPTYTVTGLTPGES
YTATVQAVSSAYDPSTQSDVASSTNTQRTD(7)PPTPTGVQLFQPTVNQTTSLKVDWQITETDFVISSYDITLTPSTSGAAVTK
NYPANANSSFTYIVTGLTPGESYTATVQAVSSGVSSSVSTPSNPQRTD(96PPTPTNVTLSKPTVNQTTSLKVDWHITEAYFVV
SSYEISLKITGTTGAAVTKTYSANDASPFTYTVTGLTPGESYTATVQAVSSGVSSSVSTPSNPQRTD(5)PGVPGIPTLSQPTD
GSDKTTILYANWTAPTGVVDSYKLLVYRGDVGGTVDANLIVTTNFANITSLIPGKRYQATVTAYSVGVASNVSGNSNYGKTN(4
)PPTPTNVQLSQPTVDKTTSLVVNWQITPDSSVINYYNIATTAMSTGTITNFYYNLTEKSYTMPGLIPGESYAATVQAFSAKYP
PATQSFVGSSLINQRTAKQNPLQLIQYLQQHNLTYVQDGDASTKQTVTLENATTSYNITGLTSGASYTISVVTISNGVRSDEAK
QQFSTI(3)TKGVSNLMAQSQTTNSIYVTWNPPPMDVLFTNYFVEYSKMNIFLMPEDHKNENIYLRICITLLLISGYLSYGFYS
LWVNVTFTQPKNGANIYIATLTGVTINEQRNGSNSSSPVQVGGLSPGHTYSAVVSASASATILVEGVASSAIIHTIKPEPVTNF
RSTEQTSYSIYMSWDLPTLGIFQSQNVTYVPSDSSENAIVVSLGNETTSYNITGLTSGVKYTISVITISNNIESDPATDESMTG
PPVATNISITASNTTCVHGTIIYVGRGIIHFYSLTWGFHGSKLVPYHPHITHFNLCGLQAGVEYDINVVSLAGNVSSTYNISTS
AYTE(2)PNSVLSVMLSSVSITEISVQWTRNSGGVSGYEVIATNSNCVVEANITIAGNTTLSTTLSGLTPGTVFNVQVKALFSA
LKSFPVYQSAPTCELVNYTDPEGGDKSIQTNFYETSATLTVLPGYNYTVMVSVTAYGLNSVIQTMTKNTKFNVMIKSRINNVNG
VIENSDTFTELYKTNAGPPEEPNITQPVNTGSTSNMNTITIILPANTFNDKQGPIEAFGVYITKETQKKPGPQPDLSRNDSCTN
LMTECVAVWYTPAGVANTPSTSSRKKRSYDNPIGNNLITFVIGSGESTISPWKTLFVNLPLAVDTPYVVAVAAKTSNNVLVSTN
WSQPIRTE(1)TPVPPAPNVGLIVGLTVACVVIVVLLIVVAWFYRKRRKESKKDVSSENQIPLQQRKTKVIQLAEFLDLLKVMK
ADSDFKFSEEYEEFKTVGRDQATVAALLPENRGKNRYTNILPYDATRVKLSAIDDEQGTDYINANFIPGNNNRQREYIATQGPL
PGTKEDFWRMVWEQNSRNIVMVTQTVERGKIKCDHYWPFDNEPITVADYTLQMTSESILPEWTIREFKITHGSDTRRIRQFHYT
VWPDHGVPDTAETLVKFIRYVRRTIDREAKHSGPTVVHCSAGVGRTGTFIAMDRLLQHLPDNNYVDIFGIVHQMRIHRVFMVQT

ESQYILIHQMVQDILNRVYDEDDDDQEPVYENTTTISDPIYENAEFNGKQKNGGVVNPALISPSEDDQNKEDSEEEGEEESEEE
GEEESEEEGNEENDQNESKLISGSGGPNHDPSVIIKQDAVEETSKERGSKATVV

>Ciona2 Ciona intestinalis XP_002123247.2 partial
LFPHPVADYTNVKTNKTFLVLNQLEPGAGYYIRIAAINSAGLGTFIKDELIKVMEETPGPVNNLQVSSYNATTVTISWQPPLEP
NGEIIYYKLTVYKQNVLATIINFEKKQNENQFNCLLDNAPRDVLSSFMQLLLPNSLLQHNPSTTLTKESGDVTIQALSEIITSQ
TDNNSTKKGKQRTLRSIVTETNEYFEPLCPVATIVTSLKPFTNYRFNVVGWTAVGEGIGVDVSHTMPQEIPEGPPLNVRVDLAT
DSTIRVMWSSPAKPNGIVTYTINIELGNWVYQSNTTYYDVTNLKPFSTYAIKVKAITKVGEGPWSNWIIANTLEGSPESVNSFN
VTSKTSRNITLVWEPPLTPNGVLLGYYLIVEENNHIVRSYWLAENNGATREIPSQSDDVIQFDDDILGGKLVDYSMKIKTKTIS
DLTPHTAYAFNIFAATVGGNGSQLTLYDVTLEDKPDSPPYNLSYSNITSTSVNISWMEPLKANGVITKYTVYITKDKLQETSVT
ERYAVISGLNIYTTYEVYVRATTKVGDGPSSSKMQIHTDEDVPGSPVLDVNFQNISSSVVKVTWSPPLVTNGVILGYTIFYSNF
TTYNTTSKTTSVYLEKLKMFANYTVRIASQTSKGLSPMLSGAITIQTMEGQPSTPPFNLSYTNTTSSVIQLTWLKPLVPNGIIQ
FYEITLQTQNNNTIRKTTQSSETSKTIKGLKPFSLYLANVRANTKYGDGGQASATYLIYTMEDAPGSQVMNVSYVNLTSTSVNV
SWLQPAEPNGRITKYKIESKLKYTEYIISISAYTMAGEGPREVVKICTDEDE(7)PSSPPDGIFFHQHNSTSISLTWNPPLTPN
GIITLYSVHYRHGNKSLIRTTATPGITLNNLKKFTSYDVYIRASTKFGDGNQTSKIKSFKTLHDA(6)PADPPHDVRVAAISST
SINVTWSPPTTPNGLIQFYTVYYQHNPSTVQTKNVTKGMQVVIGNLRKFTNYLVWVTSSTALGDGNQMSDAIKVQTLEDV(5)P
EQKVQELSPRVVNSTAIRVTWLPGQPLTGVTYFMVNVSTKTSPYTEYIISVTPHVYGVDTPGPTSLVTVTTDEAKPSSPVRNIS
IITITQTTVTITFLPPTKSNGVITSYSIMTSLDHKQLVNGSHTKIKLENLKPYKVYNVTVMPYTKMGKGPKAILTFQTLEGVPE
DSPQKVQITNITDQSASLHWEPPTLPNGIIRTYTISYQSNESQRNFNSSKTQVQLTNLAPFTNYSLQLYAWTTRGMGKLPSRVL
YFTTHESEPSAPTNLECKIYDNYKILVTWDLPLLSNGRVIAYKLSHSVLNDTQTYFVKPPQINQTLQHLQPFTEYFISVNAKTS
AGWGPSATCNITTGEGY(4)ATAPQDLNINKTSDVTITIQWNKPNNVNGRLNGYVIMYIPFDACQPNSGILNKTRCILSTNKTW
TTLIGMKQHTEYKISVFATTGRGLGAPSNISVRTLVGV(3)PEHSVENLQAIVTSSTTVNVTWESPESFAGPTTYKVEAFHSTT
MQRVTSPLLTSSTHSYVMSGLDEDSIYGVTVSTMTSVGQLSSKPVVIMTYEDV(2)PSDFPRDVNVAIVMGNSSSVRVLFKPPR
DPNGVLTNYTIQFKRLENSESRNAIIPVSDLVEPTSNNVPYYVTINGLLGGRLYKFRVRAATAVGAGPWSLWTTDVQLPITA(1
)PPVPALLPEVIIQQDKAIVTSSSVIVKKPCVFSDDNGPLKSLSVIVAQEGATLDAEPTYWAKAYSEEPSPPYKVIVTEEPQSY
CNSRNKRSTQTNNGFVIGTSDCSRLSTSQCNGPLKSNTEYRFKYRAEANNGLMTDTEYSEVFRTNPSFIEAHMTLLVSIGAALG
IFIILLTISVALLRRRRRNQIPNGNESLKTMGPIETMGPIIEEKSKVPVMESSVHSNVAFVMDDSRDMKDTARSPSINEFSRPV
AV**TDFSLLVEKLSQNKNKGFSSEFDDIRGIPYAGTTSVAEKACNKTKNRTAKLVPFDHCRVKIEGIPAVEGSNYINASYVPGPH**
SPEQYVATQTPLDHTKKDFWRLIWETGSNTIVMLCNVMEGGKKRCSEYWPKKQTEYFGNLAIQMTKQDVCKDWIVRHFSVTMRD
KVRHVTQFQFMNWSVVEQGNPLPLVRFIKSYRTTRHNEYKPTIVMCSNGSGRSGVFIGLDKIVDTMTSYIDVFGTVASLRKYRP
FMVQTLTEYIVMHQCIVNFIDDVTIEQPTGDGLEETIF

>ciona.3 Ciona Savigny genscan and curated by profiling ENSCSAVG00000003069

MIGSIWTLVFILGLTCAPC(0)NAQPAAPTGLSVTSSKSNQLTISWTDTSGTTYKVYVGTGPANVADAVDKTVNSFIATSTTGGSPMSLVDNTE
YNVAVYAVDATNTTLVSAASTQPGTTVFVAPTLNPPTNPTSSSIDLSWTAPGNGGGAVTVTGYTITYTGGGGGTQDVTGSSVTSATINMLGSNT
AYSFTIAAKSSAGTGDSSTAQPGNTC(13)HTADQCNKHCDEQRLAKRQVSVSNAKRYVPDKVVLAANTTTATIDTIGLNWNPVSGGVPVTYTV
TWSLPTSGSPMTGISANSASVPGLTASTQYTFNVVAVNSAGSSADSGPDNSQYSTL(12)PNAPAAPPSLLPGSNATNMIDVSWTAPTGGNVIV
DYVVEWMAHSTTATENTTVTTGTSTTLTLLTAGELYSVNVKARNSAGAGAKSPSASDRTS(11)LKVKHNGTTPQSSAVRQFQKITVQNRIIVV
FFDNNLLKSIMLMRYNILCLGGVRSNLIPGKLYTATVVAYSEGLAGLMSDASDKIRTSEFLGKFDKLRKPFLTYFTDPPKPTAVTLSQPTSNKT
TSLEVTWSMIGTQFIVSSYIINLTPANSGSNAPSVTYIPTFTDPTNTIVGGLTPGESYTATVQAVSNNPTPESKSEISEPSAAQRTDPPKPTAV
TLSQPTSNKTTSLEVTWSMIGTEYIVSSYIINLTPANSASNAPTVAYRATSTSPTNKIVNGLTPGERYTATVQAVSNGVSGSISDASPSQRTD(
10)PTTSLVVTWVKPAGLVDTYGVTLYNEGSLNNPVTGFPKNETGLTATYGNLIPGKLYTATVVAYSGGLAGLMSDASDKIRTN(9)PPKPTAV
TLSQPTSDKTTSLEVTWSMIGTEYIVSSYIINLTPANSGSNAPTVTYTATSTSPTNTVVGGLTPGESYTATVQAVSNDPTPEIKSEISVSSAAQ
RTD(8)PPKPTAVTLSQPTSVKTTSLKVTWSMITTEYIVSSYIINLTPANSGSNAQIVTYTATSTDPTNTLVGGLTPGESYTATVQAVSNNPTP
ESRSEISASSAAQRTD(7)PNPPASFTVNPVAANNEITFSFTAPVTPNVFSHFKLTTNATGTTNTTIISASETTYTFTDAQPNLEHTFYLSAVS
GNSPSQTESTSLSATARTS(6)YPIQASVSFTPPQNGATMYQANLTDTSGGITYQQDSGSPINVTGLNPGETYQVVVIARNMMIGDGLPSTEQT
HTMK(5)PEAVTRLLKDSSTTESISITWTLPTAGLFDKQIVEVNKVNTPDTNVTEINDNSTTTFNITGLTSGASYNISVITVSSGVQSDKMNVV
FMTV(4)TLGVSNLRVATQSTVNIYVLWDAPPASVEFDSYLVEYSVVGMFGMENTINLTAEKKSEFTNITGLNPWEDVSGGSLHHQRGCKGTIC
IRVFCNSICDDCHFVLPPSPCIGGNCHPTTATHSAKRADLHHNPPPRIHRRLHSHPRPPPAHPHRGEVHQPHPHHHPSHPRPPIHYRNEEILPQ
LDLSTNHKESYNNTQHPLTVPYPPTKLQAAANSTSSIIVSWSPPISGAWWYYVLTYYNSTHPYTIQAPNTTTQLRLTHLLPGTIHFFNISSVAY
NTSSEILQISARTK(3)PNPVLSMMATPSNMNDIFVEWLNNTGGVEGYEVQASLSGIVRSNKTILGNSTLSTTLNGLVAGTFYNISVRVLFASL
KSSPLQKQSVPTY(2)PDSVKNLKVLKSSTKPTTELDLTWDQPAGDGENIT(0)IMYTQNPGGLVTNKIASFTDTTKQLTVEPGYNYTVALTVN
QFHLSSVTETQVMNTN(1)PAAPVISQALISTTKLVLTWTINGNSENIIIEWGQQGAAVK(0)TRPLPGTTTTQTFDLVKPSTIFNFKISSQIT
NVMGATESAE(0)PHLHTAPTEPNITQSISVGSATTMTGITIILPANTFNDKNGPIQFYAVYVVKVAQKMGDGTITRSPWNQNFPNQPLAADTE
YVVAIAAKTGNDQLVSTNWSTPIRTAPNVGLIAGLTVACIVIVILIIGLVYLYLKRRKDRKKD**EFLDVLKVMKADSDFKFSEEYEEFKTVGRDQ**
PTTVALLPENRGKNRYTNILPYDASRVKLSAIDDEPGTDYINANFIPVGNNNRQREYIATQGPLPGTKEDFWRMVWEQDSRNIIMVTQTVERGK
IKCDHYWPFDNEPITVADYTLQMTSESILPEWTIREFKITHGADTRRIRQFHYTVWPDHGVPDTAETLVKFIRYVRRTIDREAKHTGPTVVHCS
AGVGRTGTFIAMDRLLQHLVDNNYVDIFGIVHQMRMHRIFMVQTESQYILVHQMVQDFINRKYDEDDTTEEPVYENATQDDPIYENTSVKHTNG
GVVNPALISPSEDDKNNTEQHDYDCDDEEEEEEVEEEEECSDENSQTNSDGVQVKIVSGNTDDKAPPHVPCMIHSADSTPNMMRSHTTRTRRVD
PVITPSRCNSAINPLYSLETTPQEDSSDSEFDDDDDDDIPKA

Equinoderms

>SeaUrchin1 gi|390362950|ref|XP_790935.3| XP_011667085.1 [Strongylocentrotus purpuratus]
MEGNRPCRHLGLSILGLILICTLQDSVAIDAPDIVTVENEGNTDSLKVTWTPPTGATDQVVTWSPADGSGSDTSFGLSGTATEH
TIPGLTPGTLYNVTVTAQNGTDETSDDSEQGRTK(18)PSPVNLTESGATNDSISAEWTKPSGAIDQYTFSCSEGTEDPSSPIT
DVSSNMYEVSCVGLSAAGADYTITVTSLSGNEISNESTTTITAL(17)PNMVHIEAGDATNSSFVASWNHPEGEMDSFQVFCYE
NDTAPEEISDVGSNSTYMYEVTCESLPTAGTGYQFSVISISGSKNVTSETVLYTSKLGASFGIRLYYFHTSSCNIWLILGPFCN
ISLVPMQHNPVTHLQPLHVHLRQHFITL(16)PNPVELTASDSAVDSVRATWLKPAGEVTSYEVQCSNGTVVPPPVQTGDGPFT
ASCEDLPTPGDEYTMNVTSVSNGKSSDVKTIALRAL(15)PEAVTLNEERGMVTTTTIAASWTMPSGIVEYYDVFCLVGDPSPV
MGNPSGNLTASCVNLTMPGDDYNISVTAVSNGQRSETDTITITAL(14)PLSANLTAGNSTINSVSAMWPYPGGLVDVFQVNCS
NGTESNVTVLVNQTNPDPSYLVS(13)CDGVWNPGDDYTMVVTSVSNEQENSASIMLTALPESVMLNEERDRVNTSTITASWTM
ANGIVDYYQVSCSNGTASPAQVNPSEDLEASCTRLSTPGDNYTISVTTVSNGQNSAVSTITITAL(12)PSQVSDLAESGATTS
SVSATWTKPDGVVSSYTVTCPDGSIQTVPEDNDGSTHNVTCSILPDPGPGGDHIIMVVTLSGTKASDPATVTITAL(11)PSQV
TDLAESGATTSSVSATWTKPDGVVSSYTVTCPDGSIQTVPEDNDGSTHNVTCSILQDLGPGGDHIIMVVTLSGTKASDPATATI
TAL(10)PSQVTDLAESGATTSSVSATWTKPDGVVSSYTVTCPDGSIQTVPEDNDGSTHNVTCSILPDLGPGGDHIIMVVTLSG
TKASDPATVTITAF(9)PNSVVLREDGVTTRQITVQWVDPVGEEDSFIVDCGDDGSNDITLFPGDATDNTFEATCNTSAAGAPY
DITVTSVSGDKESPVTGTFTAAT(8)PDGVTFEEGSASTTAVTVTWIALARADGFRVNCSEGTPSHEGTPSPDSPLPGSATEVT
CNGVTPGGNHSVSVITLENNGESPAAVMYVVAV(7)PLSADLAADTSTINSVSAMWPYPGGLVDIFEVNCSNGTERNATLAANE
DLNSSYVVSCDGVWNPGDNYSMVVTSLSNGEENSASIILTALPVSVNLTAGGSTTSSVTATWDIPGGIVDEFEVECSNGTASTP
GNGSDQDYLASCDDVDSPGDNYTMTVKSLSNTQRNSATIVLTAL(6)PESVSLNEAIDMVTTTTITASWTKPNGIVDYYEVSCS
NGDPSPVMGNPSGNLTASCTGLSTPGDDYTISVTSVSNGQRSETDTITITAL(5)PEAVELSEGESNTTVISATWTVRNSVVDT
FNITCSDGTASPPSISVDSSQAGQQLTAYCVGLPTSGEQYDLSVVAISKEKLSAVSTVGVFAL(4)PASVESIAVTEPTTTTVD
VQWNLTQCSECVYNYFLLTFQPDSQEAIRVDYMAGVNEYSSQVSGLIQGRSYNFTVVSVSGVGVEDATLKTSEEKSVDQRTG(3
)DADKRLHGSIIDIVXXXXXXXXXXXXXXXXXGTRASSLINLVAVPEAVGNISVTQYATTTADVQWNLMQCSDCIYNYFLLTFE
PDSQEPIRVDYTTGVNEYSYQVSGLTEGRNYNFTVVSVSGVGVQDATLKTSEEESVDQRTV(2)PATPSDLSIVPGQRELNLVW
AYQGDADNFTITVTPNQGIAMFNGDFNDPAAEVTGLTPGTMYDIAIVTASGDERSEPITQTNRTLPDK(1)PSPVGNPQAEAVD
KNTITLTYESPLEPNGDITGYRISYIGTRDGNTDHEFSDFYSPPVLTVTYNDLYPGFSYTFTIIAVNDGGFESDPALTEPVETP
QEEPSPVPEDYPYEANTVFSETSTTSFAVVLPDDLFSHENGELLTFAVIITIDDNDPTVSSTELTYAARAENAYITAIEIPYPY
SPSFGSNRRRRATDPPGTVVIGDGTCAGSQNEYCNGDLDDNTEYYYAFRAYNGMGNVTSSTFGPVKTVKDNTAGIAAGVSVSLI
IIIVAVVVVVVFLKRRQPKEPSPRPSLEGRENQGYDGPDHEMRPTRRKTGGSMHRKSHSKPIVLNKFEGHYGRMKADSDFRFTE
EYDEIRLVGKDQAIVSALEMVNRAKNRFTNILPYEHSRVKLAALADDSDTDYINANYIPGYNSPREFMACQGPLPGTVDDMWRM
IWEKKTSIIVMLTQLVEKGKIKCHEYWPADYNPVTYGSIQVSVQALQQYDHWVIREFSISQGDEIRKLTQYHFMSWPDHGVPDK
TWTMLDFVRTVREAIQKTASDRPIVAHCSAGVGRTGTYIALDRLMQAMQENDYIDIFGIICEM
RMQRNHMVQTEKQYIFIHECVMDLLRRGEEEDTGESIYVNLPSNGFKDPDDVNKNLLDQAV

>SeaUrchin3 XP_792888.3 SPU_001461 LOC588094 [Strongylocentrotus purpuratus]
MEGGPNAITQLTSSLTTSTNITLNWNSALGVVSYYEVAYDPPNGNSPRRTDVRADEPRTLTLSGLFPDSQYTVTVVTWSGTGED
ATSSTPFSVMFDTAPILPLQVIVRFVNETEAQLTWGLVLGEDTYIITVDDSGANPIPSSETVPEGETPLSVRKGLTPGSLYTAN
VQTTTATGPDQQFRTS(49)PYPPLDLDVADSTPTSIDVAWAPPRAGGVFDEYILSYSIGDRIRTEVGRFAPDVTTATINNLNT
ATTYNIYLVTEANDGGFPQSSDVITREGRTE(48)IPAPGEIVIINRTPTSFRFQWGPSIVPDVTYITTYSPSDGSKLDTLSIR
QSDVTGAIPGRLYELTVFVLPGDQQETGLVRTL(47)PSSPGLLAVTAVDYVSVTLTWVPGTGEYTGFIISYKPTEGITYMDVE
EVDQDVRTWKVEGLSPDVSYDFQVVAVSGEHNTTYSEPTRRINTTTLSIAADAFVVLDYNSTSITVAWRDTNTNIIVNIEPQDD
EARNVNVGDRPVHTFTGLNPSTTYTITSLSQSQNLVTQVRTNPASPSNLEVSKTSTSFSIEWEAAESEVSQYKVDVKCADMSCS
FNDVIYPNERLVYELEGLVYLKEYLVTVSSCLDMDGQFAEQMGDEPLESSIITDDVPDDEIVVQETTTDNITIFYATGNRRGFV
EAIYLAGTPAQVGSNSFVTGAVVKKTFDGLVPGTLYTIRITELTNTIIPRTVNVRTR(46)PIAPSAIFGSPTSTVIPIFWFAS
PGSISFYEVTYSPPDGTTPAVSRIDASGPLSLDIVTLLPQKVYTVFVRAVTGVDNMPSISKAVSGMFETL(45)PAQPGEIILR
RITTDTITYTLSEVPSVLQYIVQLNIGTDLQSANPHVLPNVEGEFDELTPGQLYTLVLTVPGTQGDLATIQVRTD(44)PNPPG
SVSVELTSVPYSSLLAEWEAPPAGSLSGYEVSVRPADQVLTTMSEVVDSSTTELLLDNLLPEVNYTVEVRTISAGEGFSSTSDP
TEALGRTA(43)PFPEAILLVTGYDSTTISVTWRKKEGVDYLAQFIEVGQEIGEDFQGLVQSSSSTTTILSFRYTDLVPGRNYE
LIVRSNDQSEDTIRVSQRTL(42)PSPPMNAAAAPLSAESIRVTWTAATNDFEFYEVTFYPSADSTDSTAMTIPRDQFSVDIDS
LLADTEYVFDIRSVSGSGDSLQKSAAITASATTE(41)PIIMRLFSAVENVLLIDFTPIVGIFDDYRYGYRALPSGTITYEPLD
SGTSQFGIQGLRSGALYEITLDTRIGSIYTEVDRIQARTRPSAPRLISPVTDFDENSFTLEWLAPSTAEVDFDGFRVIYTPADG
ALESPLLLDKTRTSLFLTRLEPGTEYMVTLVSVSGQGSTQQVSFEEDVSVTTA(40)DGVPGGVNIKELTETSIRITWNSALGA
TYIPTITPDEGTTRILDTEAAIFEELTPGQLYTLIMTQIGGSSGPPTQQYTL(39)PNAPMDVAISRTTAFELDVSWSPPAEGF
YNSFSLTYSRVGTSEVTTIPVPASDAPSVTLPDLLPETSYAVSVVSVVGTINSQPAETSGTTG(38)RAPEGVPVLVERTTTTI
SISWGASTDALATGYQLRIRPEGGTSILFNLGQAITTYNFMNLESGTNYIISLQIFGLSEAPQELPVTTY(37)PQTPGAIQIL
RTTQTSITFAWGSAGGNFDRYSINLYPDGSQTAFLQDVPRGSDLTVSKNYLAPGTLYRIEIYAVTGTTRSALRETTASTA(36)
MELLVFITIDAFGVAVSWPNAPDFSNYYLEYDPSNGVPPSPVPTTQDSVFLNSLTPGQIYNFALSSGTPDTGTQSLVRATYVLA
(35)PLAPTIEIMILSPTTILIKILPASSGVLDYYRVGVTARQTGLGVGQNGVASFNIPREACPEVFITDLVPGGTYDVEVVAV
SGETDSAVSSMDVSLE(34)TMVAGQLYVNDRTTTSLEVVWGAVTDRTFTNYVLNLFLGNTRTDFQSQGINEERKVLFEGLLPG
TEYSLNLLVEGDAITQSTILTATA(33)PLTPGSLSFLTVEGQEVTLAWPARNSQVDSYELCWTPSSQTRSPTADATQELLTLE
QSDVEYTISLYALVSVGVGDDVKTTRSDAITRFITLS(32)EALEGELNVTDFDSNSISVEWSEVVSIEFENYRLQATPEGDGS
PIFAVIPPEDPRMHTFENLIPGVRYTVNNILQGSDPVVTRTVSQRTR(31)PNPVSGLTVVDTTSQSVTVNWQQATGSFNDYVV
TYTLADEPTTVIYADRIPSDGTRVSVIDGLLPDTSYVVTVYTSSGTDADETTSTEESVPATTT(30)ALPSCSIQVTETTYTEI
TVQWGSCAVVVNSYELTIEPNSANAPPGNVPIGGNFEYTFNSLVPGQRYIVGIRGTGSTEPYATDSVYTD(29)PNTPDNLVEV
EVSTSTLKVQWDPPVGEYERFEVNLNEQLQGTLRGDQIEAGELPSFTFDALRPGESYSVSIFAVVGSGNLEKRSPPRQNQFTTD
(28)SLDAGQLSVIDFDSFSITVVWGAVEGITFDVYIIQFQPPTGNPIYNRVERTQPRESTVTSLIPGQLQTISLILIDQVDPI
SVIQQRTA(27)PLPPRDLQTQPTVEGSTYSILLLWSMPVPSAIDQYELTYSPADGLSESFIVLEANDEFYTFRELTPSTTYEF

RMKTVAGEGDTRTESSEVEMTQRTLSAPLGQLLLRSKTTVTISVTWGVGNTANTIPEGNYVVRITPGDIADVLVNNEERNVDFT
LLNPATLYSIRLIEQGNDVSSIDVLTDPVAPNSISFESIESTSATVTWNRFVGHDVDNFEVWYRQTSSINDYTLAGEVDGDTES
IALSGLDPSALYDVQIFSKVTSGELTSKSEPAMDSFTTGALQEGVIEFRDKTTTLIEVLFGTAPLSSEVVQYRVTLTSEEGAST
SFNVAPTSEPFSVVTFDGLTPGRLYTVSLQAFGITSTYPTKDVRTF(26)PAPVRGLFPIEIGTTYVLFTWGLPIGDYDSYELS
YLKGTAREEEIISASETNVRIEFLDPNTMYTFNMVTLSAALRSEASDQLFTTTSLAVNEIAVRDVSTTEIEIVWGPEEGVDFWT
LTTVPAVSSNILYAQSPTYRFSGLTPGQEYVITVNVNSTSQRTLPEMPQNLESVGMQLFLELQWSPPIGVYDGYRITYTLDGTR
TVIPDLPSQPPRYTLRDLDPGTDYIVSVQALSGGLESEPVTRTLTTG(25)VFDQLELSVTTVSNTSIALVWGIYEDRDNLPEL
TNYELSITPPDAISLIRGLDVFRATFERLIPGQEYSIQLKLTTLDEPVSTVVQRTN(24)PDPPAFLRSTRSNFTEIDLAFEAP
LSRFDFFTLTYEKSDGSDLSPNMRTIAGNEDSVTVEELQPGTSYDFSLTAKSGTPTEFTASQAVEDTFTTRAVPALTVVAAAIT
CDTIQIFWAPDPDDMLYRIGIQPGPANDDFRRTTEYIFSGLNPATAYSISVTVVAFESNSQSEYLPLIQQTRPSGAGAITVIDK
TTTTIDVNWLPAQGNFDSYRVYVTSENGLPKAYPPISATEERRLSITELDANVLYTVSIETISFFGTQELTSCETSTLDVRTD(
23)ALDANEIAVTAFTSTSITVIFGKREDATGYSITLRNLDGNAPRDITVNLLPSESSRVTFDTLNPGVNYEITLDSQEDTTIE
QRTK(22)PVAPATVLTQNPTVTSFDVIWTQPQGVYHNFLLVITNPDGTKRPEIALGPLDAVREVTELLPGNQYDIQVYATVGT
GEDLIRSEPASAQGITN(21)SLGALEVIYIDRTTDSLEVAWGASPRPNPGVYRVSFNTEGLTDTTPLITQDRNIRGTSLVAGQ
LYNVFVVAENYQQDSGILNVRTI(20)PFVVRNLALTGEPTAISFSISWNQPASGMYDGYAVYIAGQDNVERLTSRLDDPDTTE
LLIEDLDPNQEYTVSVYAVAGFGQDSEVRSEVTEATVSTV(34)SLDALDLETYSVTETTIGVAFGPALVAPSGYTLSLTSDGI
TIGLIIIPVAGSTKFVFTGLTSGTLYRAQVQVTGSSVTSDPLDIRTI(18)PRQPSSFMDTLVTADEISLSWLRPPSGNFDSYE
LSYTPENGAKVTLPDVATDETSLVLENLATATEYVILLRTQAGEDTFSEPLELTVTTR(17)SALLALQALPDPTSDVSLDWDR
EAGSIQRYMLIYDPNNGDPISPIETGIPGPVAISGLMPGSQYTFRLFSVLTNNVQFLSSSVSHVTT(16)PQPPGNVAVTEEMT
STITLSWSAPSQEQFFGYILEYEGTGFSTELGRTPATSVLPRHQESVTLKGLTPGTSYSIKISAFVEYDDVTTRSNIVTKIGTT
VS(15)TSSLNIVAKFVNQTSFTIEWQPAVGASSYVTRVSEVTSGVEISSGTILNGDYERTEHRLVPGRNYQIEVSATGTGQSD
TVEQRTLPSAVSDVDVVPEAAALTLSWDPVFGDVDGYHASYQLKENPYQISDLFYVSDPSNPTIRLTGLSPLSEYIITLTSFSG
SGSDQEISSSTVEASTV(14)NYSPAEIVIETVTSTSIALSWGASTSPTASSYVIEASDAGNVVHSTSRALSQSRFIIISNLTP
GTLYTIRITVTGSSERDTEIALTV(13)PTAPRSLSTDSISQSIVTVSWSQGLGNVDEYDIYLRQAGESETPAQIVPPSQLTET
FENLEPDTYYSMRIVSVVRAQNLEQRSIPAVTNFTTQ(12)PRIINVVETGRNLRLSWANDFGNDILKYQLIYIEDKEGATSNS
EFVDPSDAPLVTLMNLMPGQTYTLILTAVDTLGNRAQIASTTYTLK(11)PLRPTFTVNRRSPSAVSLLGNLLGGILKFFRIRV
TPQVTGAGAGTPINIDVLIPVEACPEIELTGLIPNRDYQVTLQAESGVASSDPQIITFTTP(10)PLTQQGELSFGEITPSGFT
VYWTPFLGRTSYELSINNQAGLVVPASGDAVQSYVVDGLSSGTEYTVSLVDGGTGAIRTR(9)PLAPGTPLATQVLDTSIQLQW
AQSSDGNVAGYEICYSPDDGSSPVYIAGRTTTQYTLEGLDSDTTYIISISALTGVPQVKSQKQTIFVTTI(8)ADTTIPVASNF
AVTNTQAADHVIILEWDVPERASYDYFEVSYTPDVGYPSSPVRLPKAATSLSIMNALADKVYQFSLVSIRGDERTDPPLTTTGQ
IA(7)VNNLIVVIGERTTTSLEILWGASASGSVDEYQVSYVGSTPIVVDGSSDRRVVIPGLSPGTEYTVTVQAINAGAAVGDSM
ETASTL(6)HEINGLSAMVTDQASSMIHLTITPPFEDPMRYDGYNVRVRLGGVSSPVVQPVIPVAKSACPEIWIKKLEAATMYE
IDIEWYSGLDISEAFRVQTSTVAAAPLDITLDELETDSMKVSWQEGTGSFSNYLVTYSPVGSTQTPMLVAKGEPRVLDLSGLVP
GTAYTIMVQQQGQTPAQETHVQLTLPEAINIDSLSVTRSKNALDATWSAPSTGNYDGYRVCHYPRGTQNSPFTSGTTPSINLPG
LNSLTYYLVTVSSSEESTYSEPTTITQQTLIGDPGDINIALEDVDTNSVRITWVAINSITRYTVGGGTGASQTVTVNSPEEAEY
TFTNLVPGTSYTFFVTPLGTQRREREQYTRPDMPRNLLFNTITTNSISIGWEKPLTGGVDSYRVTYSPSSDGSPASPHTITDNG
QITQTLTVTNLLPGTTYTFNVYALVAELVSEPVTRGSG(5)TTIGMDIGAGYTSTQFTVVWNVPTSGDFNQFQVTYDPYDPTNA
QESPRIISLSSLNGAQEGSLLIYGLEPGQLYNVYVQTMLNGLQTDDGFGRISFRTP(4)PLPVTGVVITQRTPYSLSLDWSDVI
SADIQYRITYAPLEGALFPNNEDSAQVLSSNQVSESQISARSLAPGKEYVFTISPVSGDPSSFEQVGEPETVVTYTQ(3)PLSP
QNLVVSDVGVEEVSLSWNLPAEVNFPIAYYEIDLVPNEPTFPIRINGTRDKWEIHDGLVSGGEYQAVMRSVINDVNGQVIYSEP
LQSAFFLLEPTQPQRLSTPYVTTTELRVEWTLGNSNFTTFQVSFSKDSATILTTDTTEQSLLLTNLTVATVYDISIRTVVEGPQ
TNMIKTSAAALIMAVTTKSKPPQNLMQDEVTSDSITVSWGAPLNVVNMYHIIVKDEDGADVADATVPSSETSYTIENLNPYTKY
IITVAARFNGLDSDAIEIEQTTFQGK(2)PSVVRDPVVAINSPNSVFLTWTPPDSPNGILTGFVVEITGEDLTSRKRREAGFTR
TETYGVTVFSATIENLPTGCSYTFSIKAANSADQSDAVIAGPLDLPHSA(1)PPTPPADRVVRLVSHTGTTMSVSFDSSIFSDV
YGPVTRYALLVAQSSPDTIPSTPSDPAINWQVGSGQPYQTTLLTYAPFTNGNTRGFYTVGANNSCTQDSVGYCNGPVVPLTDYR
FALRAYGSDGKFTDSAWSPLYNSGINTTWYAAAVVSIIIITLVLLLVMLLISRQCCGEARSSALYERGNGQHLPLVNAAYPSAH
NQDSMHKTPLPEDAFIRMGPIPVRENAYVSMRDDRPKAPLPATLPRSAPPLTNPVQM**RKFPDHVNRMSAGNNTGFVEEYNSLSN**
VGRDKSTDASRLAVNATKNRYRNILPFDETAVHLQDLEEGQTSDYINANYIQGLHSMKEYIASQGPVPDSVNDFWEMVFENKST
TIAMITGLVEGGKTKCEHYWPDDDTPVNYGSVTVTMTHTQEMEQWTVRSFLLEKGMQQFETHQYAFKGWVDHDVPNNARPMIEF
IRTIDVAHDANLGPITVHCSAGIGRTGVFIALHKLIKQVETSKPNDYIDVCGTVARMREQRFNMVQTVKQYMFIHQCLLLLMR

>SeaUrchin4 SPU_016937.1 XP_783483. LOC578203, partial [Strongylocentrotus purpuratus]

MVVLLPYLPLVLIAGARILSVVNTANALTFLWDEQPNPETFTGYLITSASINEVIPKQSEETSYQKSISNLTPGQLYEVSLFLQ
PITTFPAPLDQLQVRTAPSPPESLRVVDETITTLRIEWDSPASGLYRGFEVNISPPTALQELPRIVGSDIRGFTFVGLMHNTEY
TVSVTTIMGEEGLEERSTTAEKTVMTA(23)RGDGDEVYLLSVSSTDAQIGWTIPPPTTVYLDGKQITVLSQPHTIPSLTPGRQ
YTFTVEDVDLAFTTV(22)PERPDGLELESVGPYSLDWKVDTSLNDLNYERLEVSYSPESSQTDSPFDISRCTDGTFTLEGLQP
DTEYSVSVTAISGDTRSDVLTRYGFTT(21)CALEGQLHVVSLSSDEIALAWGPVNNIQGYAVRLFNAIGINMRTLSLSANSMS
ATLDSLSPASNYSVQLIVMDTDGSRQTSVLQFVTD(20)PAPPGPITLQSSTQTRLSFTWTPATSNFDSYELSISGGDMPEPRP
VGVVYPNEGNEFEVGGLLPDTTYNVRIYSVFFESRSIASEASFNTDEQILEFNVGSNQNLTVSWQVQEGAESYRLIYTTTNRNT
NSIARPATSTSITIDNLLFGAIYNFRLATIAADGTEMEVANADFVQSPLTPSTNISDIKATTALLTFSLREGIFDDYVVMLSSP
TDSCGARQPVFTLSREACPELLLLDLYPNTMYTAQVFARTGERGLSLPSETTL(19)QNFTTATVVNNEVEISKVGTNSLEIIW
KTDNVDEYLLHYSGPDGPLMSDIVPYIEINYYTFGELTPGTQYSISVISLINRERQGDALEVMQYTK(18)PDPISNLVLTPQE
NTINLTWTSPSTMYDGFLICWYSATTEDNKAWLDGDATTYPWVISTPNEEYVVSVATFVGDREGDYVLSNKVTDFITLT(17)E
TSPLILKDIATDTISVSWVTIPGVVDYTLSYSPPDGNEQATQDIDESSAIAEGLNNIYYVNFTGLTPGREYTFTVVWPLAFNYS
TDAIRTN(16)PLPIYDPSVTNTTSCNLLYQWCLPDSDFDNISITIVPDVSMRTLSRGNGTGGMCDYQTVYLDGLEPATSYNVS
VEVMSGDKSSEVVMVYSTTE(15)PLPVNNVTLDDSTIFTLTISWENPIDGSFNMFLVEYAPVRSPDSVMALDPINMGTNTAII

PNLQPGTLYNIMVYALAGEERSDATEITAMTR(14)QQNSEELRLQTRTSTSISVSWGNTTRPEVTGFVLRITFQEGDAIDSIK
NVSLGDVLGVEFSDLIPGTNYTVYLLVEVEGQLDTAVHSLDVTTL(13)PGMPGNISCDPTKTTQSLLEIEWGSTQEGNFDGYA
ISTQTGDGEEIPLESVEKDEQLQATADYLMDNTIYTFIVRSTLGDLRGEPTTMTCRTD(12)PQNLMVSTSTSPSGGQEVFLDW
QDYGASSYKVEYTPQEAPLPVVQVIIVLRRPTSFTIQITLPRRGRYRFLRLRVRRRSQGLDAGPDGDEFLIPTDACPEYTFNNL
LPDTDYAVDVNTVAEDGRTESSPSTMDVTTPRMEEGDISFNEVTTTSISFAWGMVSADTNGPFDFYLLNLKNELSIDVQNVQVN
TSETREHTFIGLMPGELYRVELIVNSNRQRSAPMYTR(11)PDKPTDIMLSDLTTTSLTIDWKAPLTSYQGFRLCWAYGETNSL
ELGPAVMERTLSDLEAGAEYLVSLAAVVGTGANQITSEKFTTIVTLF(10)PPAELHVNITSFNTTAVSIVWGTDIPLLDVSSY
VVSYWETANNGSVQDISVDDPSQTDYTIDGLVPGTRYSFMVDALGTVQAVTSIGATQYTN(9)PETVAEIRVIPSITCSVRLEW
DRPEGNLDSYIFHYNVTRGTIVGAPLQLDVAINDTAETLTYLLEGLEKDSSSDFVSLEVGVTTVAGAGEDQVESLQQVVKDLDV
NVVTITILGRDQNSIQAEVNSSVSNVESITTGLYNAEMVTSSTTRNCSTMDCESFTVDFDTLNSGTLYTLYAAVVQSSGREVVP
LLAKAATIPESAVDLQFTSIGRDYVVLTWDNPAGMIDSYNISYYPVNDITKLMFEVVQAAAESNVLRVDDLNEGMNYSFTVVSL
LEVEADLQEMGAPVEVFAV(8)VGVLGSLNITAFDETTISIEWEQVDVEEYILSYDALEGGSGNVTVQSTDPNSYTFTGLTPGT
QYTIGVQPGIYAALEKTQSTV(7)PNPPTNLRTTMVAAQSITVSWDPPSEGGSDTYNITYNVTGGQPPSPHTVSTSETSVTFSN
LTPLTEYFFTIVNIWKSLPSRSELTRAT(6)TSAQTLSLAVTPEDNTEFTVSWEDLDTEAFEQYCVMYEPYEAQENRQQESFPV
ARNIRSARFYGLDPGQEYTVYVTSCSSVSDPTSHANERISFRLD(5)PAAIAEIRIMDGSLGPRAVMLEWDQPEGIRGLYEVTL
DPADEGSVDVDGDELPVNITGVMTSIKILNLAAGKGYTVGITAISGDKRSENTTFLFYTL(4)PDPPQNLVISEIGEDTATLSW
ADPAEMNFDILEVVMTTMPATKTTILKLNTNTVELTNLVPGQTYTVQLASVIRPDDALNQPEVIGEPQVSSPFLMK(3)PASPS
DISVSNNELTRVTVTWQPSEGVVARYSYTYGLPGSTVTASETTTTTDTSIQLSNLTPSTRYEFRLWAESDQTEGSETSSSDVVT
VNIVTT(2)VFFNVPSDPVSALPVTTLPSMPSAVGGLTITKTGIVDAEVSWSSPLLPNGVIANYSLTIHISHDNGTLSPYGDTV
TISAVAEQTEYSQDVIGLAAGQYYVFSVYATNQVASSNQRSTTRTLFAETA(1)PPSPPRGATPSLVSRGSTITITFFNLFDTA
FGRIVRFAIIVQERVDGGTVVITAKRQTTSSLTWAEARRTRPVPSYQTTPDDYNPFADGAGVTTFRVGSQSCDPDDLTAYCNGA
LYPGTSYRFAIRAYGVDGKFVDTEFSSPFRTDPDRGIFVIPAIVLAIMVVIILIIFMAMGFGCCGKPDDGMYSDDSNGYGNDYS
NGNGYSNGNGNGIMKRALINDGVNTYAYQQPQIMEHSFTNIPDYSTIPPNRGTTAQVHQPSTTHTRPTKKNMFAQHVAKMSARN
NAGFSDEYNNLPAMEKTRAVTAARQARNDDKNRYRNILPYDYTRVQLKGVAGTDYINASYIKDDHGQKKYIATQGPLPNTIEHF
WEMVWENQTSTIVMMTALVEGGKTKCEHYWPAGEEPQLHGNVTVTLVGSNQTDNFIERTLLLEKEGTERTVTHYQYLAWTDHGV
PESTAPLVGLLRQVKVTNQEDGAATGPIVVHCSAGVGRTGTFIAADMLMDAIQRSTATDYIDVAGTIAKIREQRALLVQTLYQY
IYLHRTVLSLIEEQ.

Hemichordates

>AcornWorm gi|291235644|ref|XP_002737754.1| [Saccoglossus kowalevskii]
MGTQDDTLTGRVLIIIQVFIFIGFSNAGYKCTDDCAYSHDNLCDDGGVDSYSNYCYLGSDCDDCGPREVPDTTTVAPPTTPTTP
TTPSSTTRTTAMHAMQTTFVHTTTAASEAEHAHTTLNTSTDASKTTDLQTEGHETSGAESTHTPEIVTDVIHSSRTEYEVMSMM
TTNENEENNVTMYNIRTEEQETEVVDSSTLVGTEIVITGFTPTEEQETEVVDSSTLVGTEIVTTDFTPTEEQETEVVDSSILVG
TEIVTTDFTPTEEQETEVVDSSTVVGTERVTTDFTPTEEQETEAVGSSTLVGTEIVIISFTPTEEQETEVVDSSKLVGTEIVTT
DFTPTEEQETECVDSSTLVSTEIATNGFKPTEEQETEVVDSSTLIGTETMTTDFTPTEEQETEVIDSSTVVGTEIVIISFTPTE
EQETEVVDKTLTTDFTPTEEQETEVVDSSTVVGTEIVTTDFTPTEELETEVVESSTLVGTEIVTTDFTPTEEQETEVVDSSTLV
GTETLTTGFTPTEERETEVVGSSTLVGTEIVTTDFTPTEKQETEFVGSSTLVGTETMTTDFTPTEEQETESVGSSVVVGTDVGS
TGGAQTEGPETEVVDISTVVHTEMVTTGGTPTEEQETEVVGSSTLVGTEIVTTGFAPTEEQETEVVDSSTVVGTEIVTTDFTPT
EELETGVVDSSTVVGTEILTTDFTPTEKQETEFVGSSTLVGTETMTTDFTPTEEQETEFVGSSTLAGTEVTTTGFTPTEEQETE
SVGSSVVVGTDVGSTGGAQTEGPETEVVDISTVVHTEMVTTGGTPTEEQETEVVGSSTVMGTGVMTTGVTQTEKQETEIVGSSK
VDGTEVVTSGLMPTVEDGTGVIDSSRFASTVKVATRSTLHSTTMVRPTEEDTESTTVVLSTLPTILAVYVTSTEVQMADDSYLR
EWSDSVALTFDLKLSTNESLQSVSLNNDWKLIIYIRDVSSTNETVNERLRPYEVEVDNFQISGSDLVFNDIIVNMNLSGLLCSE
MTYICIELIAYDGNGTDLVFIREPVDCVSILCRGVEIAPFVDVVTGSKLREGMDDQSFQFNLILYPDPEAGSIEGEYLWTLTSF
FSQSNDGYGNTDAGTVHSLTTDQSGMSLYSGIPASWTGFRSSAINITDKLCSDVSKYFCVNISKSSLSNPDFTFGNPVMTCTPT
PCTGVDVVDTRLSIVSGIVVKEHESSQYFNVFVTLLSSPDFGDIEGASLWNVTMYVNTQGNANVGDVSALAVADIPRDMRDLSL
LAGSNAILEGVEAFMDLAELNCRQIRYLCVDVSRDQTSVPAFTLTGKLQTCAEITCRGVEITDTELIVKSGTSIVERSGNHSVT
FDVLLSSNPAAASISGDRLWEMYVYLSDVEFDDTHSFAATYPVLSSVEDTDLTAGVTLALLGIEAFLDIDSLTCDQMRYICVTV
FKGRYSSPEYTLTGVPDQNILTACQPVTCTGVIISTDLQLSDDFYLLEGVDNQHISYTMTLFASSAGAGVSGMNLWDIRTQLRS
IEDAIVDTSNGTIQEVNTTLNAGGVLLMSNVKSVLSLEGVLCHQFFNICAILNKNSNANTEYTIEYDRSTLVSCRSVSCKGVEI
IEVAPTIMNGDIVAEADDAHAVSINVGMTSSIYGTNRTGAGSWALEVFVTSDDVRMPKLVNLYATLTAAQQDTELIGGIPSVLS
NIEVELDVSALQCSEFTQLCVVLHKGDNAEPDFTLGVLQDENALPGCAHLSCQRVAFNISAINVTTSSFTIRWDEAVGDFDGYY
IDVSPALTGLSSSFITERDEPREASFFDLESGQEYVISVGIAKNGFPKSTGVTQYTQRTF(6)PEKVDSLRVSMVTETEIHVVW
DRPDGVVSTYVISIQPSEGTATLPITLPSDSTTASFSGLSPMSQYVITIVTYAGSLQSKEVPVKVITG(5)TIPPVLEVTSFTM
DTITVATYVDNRLSHGMLHLSYFPTDAEPIPIELDLNKTSYVITDLVPGRLYTLEVKIVADIDGGRITSNSDRKYQQTE(4)PS
PPGSIVVSRITEFGAIVNWEPSSGDFNFYLVTYSSASVNTALPIRVNHGQTPQLSLSGLSPATVYTVHVSTRSGELTSQSVSTT
FNTL(3)LAPPGRLSVNGVTSSSVQISWLPAISGLAVEYYVTYAPFVEGATPASPFTLNGTSVNITNLYPFTIYTVTVQTRIDD
MYSRERGITATTYPDL(2)PSSVKGFNVTVESPYQVTLTIQQPSFANGILESYIVNVVGYKEGFPPHILDFEIPPDETDDITEY
LLEDVIAGYTYTFTVKPQNSLGIGPLRTAGQVIMPIYP(1)PPKPTASRITANVRFVETTAYTLRIHLSNNLFDDRFGIIVAYL
VIIAEDGEYSPLPEVLPSYDEVIDSSPWPPYQTSQPFNPFETLSIGRRRRNTLQTVNYIIGAKENCDVSLVYCNGALRPVTDYR
YVIRGYNELGNYTDTDWSLPQKTDLDPFWILYGVIAGLILIGIIILICICYCCCCRRRRSSTQDSDKLSEPPSYTPYACENGVF
FNGNEPTLEKTEKLYTLSLRRKRKSRPVPLKGFRDYVNELDEDDRKGFEEEYDDLRKLVVRQPTSVGQRMEHRSKNRYTNIVPY
DNNRVILSGSGNSYINASYISGSKGSRSYIATQGPLESTCGDFWKMIWQQRVTTVVMMTQCNELGKSKCHHYWPRDTQTQISHS
DLTIRLTSETRLPDWIIRDFSIESNGEIRALRQFHFTSWPITGTPYDADPLIRFIEAIRIQVLPNSGPILVHCSAGVGRTGVFI
ALYHLLEYFYTMIQVDIFGKVIKMRKQRPFMVQTQGQYEFLYYAIQQHIKEKPLGTNWSMRKTMDSNWTITYGGTVDPDDSGPG
FFDPYDPEEV

>Acorworm1. XP_006823994 curated profiling| partial [Saccoglossus kowalevskii]
MSLKHAFCFPANVNVIDYGTDYITVNWTHSGEHVDEFVITAIATENEAANGTETVNITRGTVDGLTPGAEYTITVVAVSQDDVE
SLPSDEVIQRT**I**PEPPYNVTVESYNTDSIHLRWEPPLSNVFTHYNVTYVADSDPGMTVTVNKSTTESSLTGLISGETYNITIKT
ISGDESSGASDAVVQTT**E(16)**PNSVVLARDETQITTDTIAVTWSSYSGDIVDTYTVQCSSNGTAEEATVDYVNGTDTYSAKCT
GYETAGKLENITVTAVSGTSGPSQSLSEPSMIEIHT**K(15)**PNSVVLARNDSQITNDTIAVIWNSNAGDEVSYYTVECSDGGTP
GQAMIIYDNTVTYSAKCTGYGTAGKLENITVTAVSGTSKPSQSESEPSKLAIHT**K(14)**PKSVTLARDETQITNDTIAVTWSSY
SGDIVNTYTVQCSSGGTAEEATVDYVNDTVTYSAKCTGYGTAGKLENITVTAVSGDINTTGSESEPSTMDIHT**K(13)**PNSVTL
ARDDSQITTDTIAVTWSSNSGDIVDTYTVQCSSGGTAEEATVDYVNDTVTYSAKCTGYGTAGKLESITVTAVSGDLNTTGSKSE
PSTMEINT**I(12)**PNQADPVRNDNLVTNTTIGVKWTDPGGNINHYEIGCPDGGTPSANVTHTPDKEMTAQCTALATPGGLYSIT
VTSVSGNSKSDPAVTSMNT**K(11)**PNKVRLERDDDNITNDTIAVTWNSNSGDEVTYYTVECSSGGTAEDSRVDYVNGTDTYSAK
CTGYETAGKLETITVTAVSGDLSTTGSESEPSMMEIHT**E(10)**SNAITLTKDSEYVTTTSIAVTWTNPGGAVAYYTVECSEGGT
AENGSIPYIDNDQHEYSGKCINYNNPGNLETINVTSINADNHRGETSSMEINT**E(9)**PLPVTDLTKVDATTAFIEFSWIEPESG
TWSGYDVGYLSEDGTQQFSEYLSDDVHTITSHTFNNLTAGVLYDITVITVSGEEMSTNETEPMRT**I(8)**PNAVTDLVLTTPNQT
NPEIVIMWSPVEDEVSHYRVKCNDSDDVTTEPDDQIIPHENVTSLTTECSDLFPGRLYNVSVSSISGYEGVDETESEPIVKAKR
T**H(7)**PKPVTSLSLTSPDNSVDTIHVSWTAPEDSDVDGYRVVCTETDSGDKIDDVEVTVTNYDFSGLTAGRLYTIDVYSYSGSD
NDEKLYSVNTTDVKRT**Y(6)**PKPVTSLSLTSPDNSVDTIHVSWTAPEDSDVDGYRVVCTETDSGDKIDDVEVTVTNYDFSGLTA
GRLYTIDVYSYSGSDNDEKLYSVNTTDVKRT**Y(5)**PNSVTITSTSTTTDSLNVTWSLPDGNWTGYRIEYVPQDNLANAVLRRED
ISDPEQKIVHHTIYDLYAGTLYNVTIFTVSSGIDQLESLPDTTEDRT**V(4)**PATAYNVTEVESSKTSYSLEFSWKINTTSLHDY
YEVYYRGQLDTSFRNTTSDSENVSLVDLRAGDTYNVTIITISGKKRSAPSEPAYGTT**T(3)**PLSPGAVSVIDVTETEIKIQWAQ
ACENCTYDYYVHHEPEDGEVDVDSSSLHAKLYDLNHGTLYNISVVSISNDVPSEPTYTLART**L(2)**PDTPENITIETGMRSLNV
TWDEPEGEYAGYNISIEPSTNRAGKDIIVDVPDKGIEECIITECDPYTNYKITIIVHSDEPNQTFSEPAVSHAMTETA**P(1)**PP
PPRNNIVPSSSESDIEVSQTTITVLFTDDYFNDSNGPLLNFTVIVTEDG**T**EEVDKHLNGEIPRVYCKDCENVPGIEEGEVKTWA
EVQNTPARYQIDYPYDYP**D**LSRRKRDTTGIEITIGSDDSCSTKDEGFCNGPLKDGTSY**R**YHFRAYTDVGYSDTVLSGPIRT**S**TN
MSWLWLLLGVLLGVLIAVAIILAIIYYKRRYYEETTTRTVEGTANPEFIGLRDENGKPPIAQKPKTQKHSKPVKL**AEYHGYFQI**
MSADMEFRFSEEYDELRPVGREQSWDAAELPENRAKNRYTNILPYDRTRVKLSQVEDDEATDYLNANWMPGFNSPREFIAVQGT
LPGTKDDFWRMIWEYNVSTIVMVTQCQERGRVKCERYWPTDDNPVYYGDVLVTVTHENELTDWVIREFTVENGKSLRRIRHFNF
TAWPDHGVPEETGSLIKFVRSVRAQIQNDGTPTVVHCSAGVGRTGTFIALDRLIQHIKEYDYVDIFGIACEMRMHRVYMIQTES
QYIFIHLCVDDLLKEREQPDEPDEPDSTPDTTEHIYGNVPSVTVMRNGPDVVTANLGDAYKRNSVIAQA

PROTOSTOMES

>Nematode.2 CeNTP10D C. elegans. F44G4.8a DEP-1 NP_001254192.1 [Caenorhabditis elegans]
MIRWKYELHSLIWLFLVLHLSKCQSDSLTTSAEQHELFAIKKDSLSPWSQILVSLPRRHPLYQSFAAKIQDVTENISDEVRDSN
KTFVSSDDAPYSIRIHALRAGHRYSIAIHGQKDGSTSLIKEESVVMDPRAPDFRSMDSDIQVAEHNITMRTIKNDSYLQDSFSI
EYRQINPDKKFPVLQILDIPEQKNLEFYLGNLNSGFDYSVRVIAHKDGMSSRPWISTLTTKPSPLKEVNINQNAGSCVEVSWQN
DEFSGADFYTIQYSLQSTPNNSTNMTIPSTESSISICDSMLQGEAYQIIATVQKGGQVSEPLITKFQLRPLPPIDFRVRADLKR
GKYKLLAELPTSSKIDKCQITVAGDEAERSVNYANIEQTKSGHKICWFNFALSPGERFDFSISSMANESASQKLQKSIVLTPAF
DFNAFGLTLQESNGGIELIWPKSEVFMTRVKDIWNKVVGAESLLNMRITPIGNNDETDKTLKFETSPKNIDPVFAKNLVKGACY
RVQLFTVTKTGIISETRHNETIRMSSPAVNVSLESVTRSSATLRIVFSTHHDSTSISNCQMHIVVRDMNGKSVFDKRMQLTATF
APLLNLDGLSPFHKYTVNTQIICGSGSSETPQCPAATRTMRQLSFSTRQDKPAAVQDLKVEPLNSYSVMLTWLPPALPNGILTH
YAVNVTKIGSDETRTIDVGVSSNRSDHTVQVVIDELFGGHTYSFSVRAVTEAGFGENSPVVPTVSMPLMAPPVPTVAPMIMKES
VGSHNMIVRFPTTMFDNRNGEIKQFAIIVSETTADESINRWIESDNGTYTWQQVQRFDVWPSYVAKLQDIQKVKQDVDVSIFEE
LGEDETCLEVRADRICNGPLRSASKYRVRIRLFTSPTLFTDSPPSQVMTTGSATPAIPLLTVVAVLIVIAFVGIVGTIFLFFWN
RTKKARLAAAAFKNGPSKEKESQWEALKMMMAERAADCLAKLGLDATTPPPSSTTSSNSPTSTSTTMTDCGSNPHLGAPNAGGH
RRTRSLRERTGVEHRLERLSSGPVHRTPLYTVVTGANTNKSRPVRI**EDFADHVRLMSADSDFRFSEEYDMMRNVGVGQSVAASE**
LPINRPKNRFTNIPSYDHSRVKLSNPNNIEGGDYINANYVPGFSSRREFIAAQGPLPTTRDHFWQMTWEQQCPAIIALTKCVEK
GRDKCHQYWPDHENVPVLYGDIEVTIVAEKEFDEFVIRDIRLEKSGPDGRVTRFVRHWHYMAWPDFGAPSHPNGIIQFSRMFRH
HLPHSPHNAPTIVHCSAGVGRSGTFISIDRLLQSSSFGDPIDVFGTVCEMRYERCQMVQNEQQYIFIHYCILQVLQGSSPSPTS
TSTGAHHNAGFVQDGQMIVESGF

>Nematode.3 HcNTP10D Barber.Pole.Worm gi|560121775|emb|CDJ93597.1| [*Haemonchus contortus*]
MVGLDVALEVVMVIIRTVLTLATAIVFATKELRAASNAESSEDTHEKKLDLLEFKIEKDQKLPDSQLTVKLPYNHPSYDQFIAK
VVDISSTIDPSIRDVNRTFLSSADGSGTIRIHTLHPGHKYSVSIVGRKGENSSVIKEELVVMDPRVPEFTTQNSDITMHNITLR
AVKNEKFLEDSFLIEYRQLEPEQGYPVLEVLDIPEQKNLEVYLGNLNPGRDYSVQVVAVKTGLKSRPWSTTLSTKPGAVSDLIV
SENSSCLTVEWDVPPNSGADSFLLRYRQLNAAVNLSVTIPGNDRFVDLCDGIVPGTAYALGVVAKKGKSVSDETIKTYTVRPAS
PTDFQVSPDITKGKYRLTFDLPPNSNYDGCHVSVVSETLEALEDDGEVNEKEGNKSCSILMPLVPGERFEFTLSTYVENVTSTK
LHRSVVLTPAFDMTGFGLSLQEYQSGVKLSWPQSDVFMSRMRDIWNKVVGPKSQLQMRLFPADGLEKGSRLQGDPHEATPLIVG
ALKKGSCYKVQIFTVTKSGIVSEKRYNDFFRMSAPLVNITVKSITRSSAVIHAAFVSPGEADPECLLNAIVLDMHSHVVLDKTL
KAQSHNFSPIELGGLRPFHKYTVNSKITCASGPSDCISTSRTIRPMTFSTMQDKPGPVLSLSARPLNPYSAQLMWLPPALPNGI
LTHYVVDVKSEDDPNDSRSLNVAIGTDRADHFVETVIDGLSGGERYTFTVRAVTEAGTGELPTALLEPVRMPILAPPRTSTVPL
VIAESITSHSLTVKYSPTMFNNKHGRIIRSTLLVAEVTEFGQISETWMNAENETYTWMQVQRFDVWPLYAAAVDEISARGSPSL
SQAVGIDQTCEDLPSDVICNGPLKAGTNYKFKLRLFTATNLFSDTDFSEIITTEAAARGAILKIAVLALAIAVLLGILGVLFTS

YWNRSKKARAAYASSKESQWAALKMIMAERAADCLAKLGLDGSMHNGEIPSQPSHLQPTFGHHRRCRSLRERTGVDHRLERLPS
GPINKTPLYTVITGVNTNKSRPVKI**ADFAEHVRMMSADSDFRFSEEYEILRNVGCGQSYTAAELPVNKPKNRFTNILPYDHSRV**
RLMQAGDQEGADYINANFMPGYSSRREFIAAQGPLPTTRDAFWQMAWEQYCPAIIALTKCVEKGRDKCHQYWPDSEHSSVVYSD
IEVTLLNESSYDDFTIRELRLKKLHESTAPRIIWHFHYMAWPDFGVPDHPQGIIRFALMFRSRLPHSALNKPTIVHCSAGVGRS
GTFIAIDRLLQTIQADRPIDVFGIVHEMRYERCHMVQNEQQYIFIHHCILHAIESIASERTGMTTELHQNPVFEDDDTIAESDF

>Annelid gi|443734862|gb|ELU18718.1| CAPTEDRAFT_229306 [Capitella teleta]
MSQPVTNQLAGFGVLAALALICVSRSESSSTTTAAVPTTNFSTNIPPTTQGLEVTEVTTSMVTLVWSYDGSEQLTFVVEYVEKD
ADNATSFVLEQGVTSCVVEGLQAGWTYTFEVSVQLERDMLGEPAYANATTIPGKPINFRTVDNSVGATFFTLTWESAAGSQQDD
FVVVYRKIGSSDVVQVFTGGATSVTLHGLDAGVLYEVKVMAVRGQQLSTPAIITEVTR**(12)**PPTPRIHNITAVDTKSVTVQYS
AGAGGSQNSFVVTYVNQEGQTNEPVCSYSNTCTFDVSVFGVKPGEHFTVKIKATQDMVSSAWASMSGNTKPLPPSAPKLFERSE
TTLTVIGDVPSTNSTVFDGCKASVTPAIQNGPYVFKRNGGYEIRLQNPTPGSKYAINIWTYSREEESAHVSGDFYSMPAQPGVI
SRHTGIATTTKQLAVSWGAPNSGQVGGYVAKLYEHGKSVAIESKTLTTNMRQATFHNLTPGQVYDVSVSSYVGTGVYSAFSTIE
LSTK**(11)**PESASNLELKTTTSSTLVVQWDEPSTGFYDEFLVRIQSHGETEKITKSSQSTYEKEFDNLSAGTEFTIVVITVSHG
QQSTATIGIFYTK**(10)**PERPSNLKKESTTSDSITVKWNAPVSANFDSYRITINSTDHENEVLDSSESSHTFSGLMPAQQYDIT
LISLLNSLESSMLSIQVYTN**(9)**PAAPTDLRILSRNTNMMSFTWSPPTNPGTIDDYKYSITISGAATPEIERSGTINEYSHQLS
AGKTYIIEVRAVTNNQRGLPVSLTFTMI**(8)**PNDPGNVREMERGQSYLKIEFDAPWDTYDGFEVTWVEKGSGPSSVADLLVTSY
NDDSITVEWEKPTGTTVNGFTISIDPPPSGQSGSKDLTENIQEYTWTGLSAGREYNLIIEVIFADTKSEKVERRQTTK**(7)**PKR
ALSLSANYVSTDSISLSWKVDQSSSQDNFKITYEVTGSSTEWTRETTFTQGQLQYSYLIEGLSAGSTYSISVIAKKSSVQSDPL
EITQTI**(6)**QPKSVRELKAEVIPSGISLSWLPGFDSTQNSYRYQYQGKNVKLNIVPWVVINSESVDLQNLFPGERYQFYVDAIS
NDQYSPSNQSTLATTY**(5)**PLPPTDLTVDRSATTVSSVRVQWKDDVTRSYITSWDIKIADRGTDNIRRVGSTHERTNLNYEIPN
LTAGKNYTVYVYGKSGNQHSRDASTMDVTAK**(4)**PVIRSTLSEDTEKTTEDTIAVTYSESKAGVFDHYLFSLLNSSDTVIKQRS
NTDRNIRFENLVAGVKYTILARTVSGVEESMTTQKSITTK**(3)**PNKPKHKCNQGTDKLTIILIKPSGFVDEYILKCLNEDCNST
EISNVSFEQDFNNLKPYTTYHFEAYTKRFEKLSNKVEFPCKTGEGP**(2)**PGPVQEFSYSENSLKPFEIRLTWMEPRNPNGKIIK
YHIKYNGIKENQVPHTGSEVDVEPDMHEQLIKGLKPGFEYTFEIAAETNAIGKKTTLVKTMPIKA**(1)**PIVMPNSPPPQAITTA
ISHDKIRIILTNPFLNTNGDVVAFSVFVTTDPNERFMANSPLRTWADVKGPSPMASYFAVYKCANLFDGNDQCSSGPARKRRVA
QPRNTVEFTVGGDSSCTTNADDYCNGPLDAESTYYVALVGYTENDLYSSGPSSEPIRTDTAPTNLLLIIIVVVVVLVLAIVAAI
GVIVYMRKRSSDNNEDKLVCCLVLIGVKPASPEWPPGSNNGMVIIPSSKPVKK**VDFVQHVASMAADSEFKYSEEYEELSLVGKD**
QPKEASDLPCNRSKNRFINILPYDHSRVKLLPIDDEDGGDYINANWIPGYNSRREYIASQGPLPSTRDDHWRMIWECNCQCIVM
LTKCVEAARQRCDHYWPIDSEAIYYGDLQVQILNETKSTDWSISEFIISKGDQSRRLKHFHYHSWPDMKAPNKTGSIVPFVRLI
RAKVQLDGGPLLVHCSAGVGRTGTFIALDHLMQHIEEHDDIDIYGTVYQMRKHRCMMVQTESQYIFIHKCLEFVLEGGEDDEYA
NTGAIGTENRAFEGLRSESDLVAEIFFNLPRQALTNFDDDEGIDVKSDP

>Fruit Fly DPTP4e L20894 FBtr0301793 Drosophila melanogaster
MDCATRKQQQLRAHHQQQQIQIQTHGRKRQQLQKQRHNHHHYYQNSQQQQKHFVWLVVGILTIFLAQHANAADLVINVPNASSN
ANAFYRIDYSPPFGFPEPNTTIPASDIGKDIKFSRALPGTEYNFWLYYTNSTHREQLTWTVNITTAPDPPANLSVQLRSSKSAF
ITWRPPGSGRYSGFRIRVLGLTDLPFERSYSLEGNETLQLSAKELTPGGSYQVQAYSVYQGKESVAYTSRNFTTK**(1)**PNTPGK
FIVWFRNETTLLVLWQPPFPAGIYTHYRVSITPDDAIQSVLYVEREGEPPGPAQAAFKGLVPGREYNISVQTVSEDETSSVPTT
ARYLTVPERVLNVTFDEAYTTSSSFRVRWEPPRTYSEFDAYQVMLSTSRRIFNVPRAANGDSVYFDYPDILEPGRTYEVVVKTI
ADNVNSWPASGEVTLRPRPVRSLGGFLDDRSNALHISWEPAETGRQDSYRISYHEQTNASEVPAPFPVAAESQITTNLTEYTLD
SLLAGRRYLIAVQALSKGVASNASDITRYTRPAAPLIQELRSIDQGLMLSWRSDVNSRQDRYEVHYQRNGTREERTMATNETSL
TIHYLHPGSGYEVKVHAISHGVRSEPHSYFQAVFPKPPQNLTLQTVHTNLVVLHWQAPEGSDFSEYVVRYRTDASPWQRISGLH
ENEARIKDMHYGERYLVQVNTVSFGVESPHPLELNVTMPPQPVSNVVPLVDSRNLTLEWPRPDGHVDFYTLKWWPTDEEDRVEF
KNVTQLEDLSSPSVRIPIEDLSPGRQYRFEVQASSNGIRSGTTHLSTRTMPLIQSDVFIANAGHEQGQDETITLSYTPTPADST
RFDIYRFSMGDPTIKDKEKLANDTERKLSFSGLTPGKLYNVTVWTVSGGVASLPVQRLYRLHPLPISDLKAIQVAAREITLHWT
APAGEYTDFELQYLSADEEAPQLLQNVTKNTEITLQGLRPYHNYTFTVVVRSGSIQGTDFADVSVSTLMRSSAPISASYQTLTA
PPGKVDYFQPSDVQPGEVTFEWSLEPAEQHGPIDYFRITCQNADDAADVSSYEFPVNATQGKIDGLVPGNHYIFRIQAKSALGY
GAEREHIQTMPILAPPVPEPSVTPLEVSRTSSTIEISFRQGYFSNAHGMVRSYTIIIAEDVGKNASGLEMPSWQDVQAYTVWLP
YQAIEPYNPFLTSNGSRKSSLEAEHFTIGTANCDKHQAGYCNGPLRAGTTYRIKIRAFTDEDKFTDTVYSSPITTERSDTVIVA
ATVSAVLLVAMVLVVVYCQHRCQLIRRASKLARMQDELAALPEGYITPNRPVHV**KDFSEHYRIMSADSDFRFSEEFEELKHVGR**
DQACSFANLPCNRPKNRFTNILPYDHSRFKLQPVDDDDGSDYINANYMPGHNSPREFIVTQGPLHSTREEFWRMCWESNSRAIV
MLTRCFEKGREKCDQYWPVDRVAMFYGDIKVQLIIDTHYHDWSISEFMVSRNCESRIMRHFHFTTWPDFGVPEPPQSLVRFVRA
FRDVIGTDMRPIIVHCSAGVGRSGTFIALDRILQHIHKSDYVDIFGIVFAMRKERVFMVQTEQQYVCIHQCLLAVLEGKEHLLA
DSLELHANDGYEVTKIYLERQPQTKMGTLPIRASLAMAEKLDADLMTNKDEDEDQEQQQQQLQLATEVKPKGSNDDEEDEEDD
DDDDDDQQPLNNETTATLSSASCSSSTHDVHVVLQEAIEKPKQEQERICAGTQSHADTESDNTDSDDDDEDGDGKVAKDGAVAD
EDGWWY

>Fruit Fly PTP10D M80465 FBpp0303419 Drosophila melanogaster
MLYQLSKATTRIRLKRQKAVPQHRWLWSLAFLAAFTLKDVRCADLAISIPNNPGLDDGASYRLDYSPPFGYPEPNTTIASREIG
DEIQFSRALPGTKYNFWLYYTNFTHHDWLTWTVTITTAPDPPSNLSVQVRSGKNAIILWSPPTQGSYTAFKIKVLGLSEASSSY
NRTFQVNDNTFQHSVKELTPGATYQVQAYTIYDGKESVAYTSRNFTTK**(1)**PNTPGKFIVWFRNETTLLVLWQPPYPAGIYTHY
KVSIEPPDANDSVLYVEKEGEPPGPAQAAFKGLVPGRAYNISVQTMSEDEISLPTTAQYRTVPLRPLNVTFDRDFITSNSFRVL
WEAPKGISEFDKYQVSVATTRRQSTVPRSNEPVAFFDFRDIAEPGKTFNVIVKTVSGKVTSWPATGDVTLRPLPVRNLRSINDD
KTNTMIITWEADPASTQDEYRIVYHELETFNGDTSTLTTDRTRFTLESLLPGRNYSLSVQAVSKKMESNETSIFVVTRPSSPII
EDLKSIRMGLNISWKSDVNSKQEQYEVLYSRNGTSDLRTQKTKESRLVIKNLQPGAGYELKVFAVSHDLRSEPHAYFQAVYPNP
PRNMTIETVRSNSVLVHWSPPESGEFTEYSIRYRTDSEQQWVRLPSVRSTEADITDMTKGEKYTIQVNTVSFGVESPVPQEVNT

TVPPNPVSNIIQLVDSRNITLEWPKPEGRVESYILKWWPSDNPGRVQTKNVSENKSADDLSTVRVLIGELMPGVQYKFDIQTTS
YGILSGITSLYPRTMPLIQSDVVVANGEKEDERDTITLSYTPTPQSSSKFDIYRFSLGDAEIRDKEKLANDTDRKVTFTGLVPG
RLYNITVWTVSGGVASLPIQRQDRLYPEPITQLHATNITDTEISLRWDLPKGEYNDFDIAYLTADNLLAQNMTTRNEITISDLR
PHRNYTFTVVVRSGTESSVLRSSSPLSASFTTNEAVPGRVERFHPTDVQPSEINFEWSLPSSEANGVIRQFSIAYTNINNLTDA
GMQDFESEEAFGVIKNLKPGETYVFKIQAKTAIGFGPEREYRQTMPILAPPRPATQVVPTEVYRSSSTIQIRFRKNYFSDQNGQ
VRMYTIIVAEDDAKNASGLEMPSWLDVQSYSVWLPYQAIDPYYPFENRSVEDFTIGTENCDNHKIGYCNGPLKSGTTYRVKVRA
FTGADKFTDTAYSFPIQTDQDNTSLIVAITVPLTIILVLLVTLLFYKRRRNNCRKTTKDSRANDNMSLPDSVIEQNRPILI**KNF**
AEHYRLMSADSDFRFSEEFEELKHVGRDQPCTFADLPCNRPKNRFTNILPYDHSRFKLQPVDDDEGSDYINANYVPGHNSPREF
IVTQGPLHSTRDDFWRMCWESNSRAIVMLTRCFEKGREKCDQYWPNDTVPVFYGDIKVQILNDSHYADWVMTEFMLCRGSEQRI
LRHFHFTTWPDFGVPNPPQTLVRFVRAFRDRIGAEQRPIVVHCSAGVGRSGTFITLDRILQQINTSDYVDIFGIVYAMRKERVW
MVQTEQQYICIHQCLLAVLEGKENIVGPAREMHDNEGYEGQQVQLDENGDVVATIEGHLSHHDLQQAEAEAIDDENAAILHDDQ
QPLTSSFTGHHTHMPPTTSMSSFGGGG

>Fruit Fly.Ptp52F-RB FBtr0289985 Drosophila melanogaster
MEATNWKQSHRRIHALILILWIAGVQGQTVGSLVAVSESQELAEKVEVTVVSTVYTLGFSISNEADYSIDKVICRNGSGSETEA
NAAHVCENLNPCTFYTSVVSFRSNVSGKPPPSDQTIYAYTEYKQPKTTVTSVVATANTIKVTWQTNDRACVESFGITAKATDYT
KSFQLLNDKSSQTFVNLSACLTHTITLDTRNNASVVVDTDATDVDTQYAEPGDLVMNVTNLASGITMITWGDPSEKNCISNYVF
KWQRNDCGTDQNQDTTTDVPSTETTNEIDYVTTTPMETTPDTPSDEVKCEWTDVSSDGKLREYLLTDLQGCDLYTFQVFINENS
TAKASQTFTSAEKL**(3)**VSAVYEPSPTAYPTQLHWTWFSPQNHPKCVANYSVTLTGPIQRSENKTMNVITEETFAIFDDLDPCG
IYLVEIVPNQLNGSAGTKYQEQSTVGEDQPSVIQDPVVEAEAYSMEVSWKTPEYADLCIDGYRLSGWMEDDKLVEVEALSITTQ
NTTVVFDKNLLACQVYIIQIIPYTKENLDGQLRQVGVETKAAIVDYTKVKLEMKNAGSEFIDLIAFNADYNNSCPTIFALFTCN
ATTQVRNPYAERYVEGHSKQGFNASLSPLSPYATYVCKVILYNVAGPSEPVVDADMHTTTYFPEQPESVMLEKSTVSSLLFNWQ
PPTYTNGPIKYYQAFLMRHEASYFVPADCAIVEQDTKSETKGDPSVNFTGLAPAVRYMMQVAAQNDFGMGVYTEPVIGITLPAV
(2)SDSVTQLTVLTQGPVNNNAVYEANVTITWKVPCKSNGDIEYFQLAFNGTRNNFAPVSFERRVELDTGNKQGRMSYTETEMQ
PQFDYTVEVSVKNRDVEQLSSSVPGSWQSPAGL**(1)**PTIPSDELIKQMRANVEETSNPTKTAIVRLPADIMTSASGDIKWMALM
ISQKNCAGVPHLKYDVSSDWPKVLSYQEAGADGTGDCSLEYQTTEERWHPEPVQRQRRDGEVTSDEEIVFTIGLDKCSEVQKTY
CNGPLLPDTDYNVVVRLFTASGYSDAAVLNFKTKAAIKVTLILVSVCSCLLLAFVLGLTVLWVRKRLAWKRDSGQGIEDPFGNV
IA**KNFAIFYTEVAKPEKLAREFKEITVVALELSYSASELGCHKNRYADIFPYDKNRVILDIDAEGSDYINASFIDGHTRKKEYI**
ATQGPKPESVMDFWRMILQYNVRVIVQVTQFREGNTIKCHEYYPYNVRGLTVTIKSKEVLELYDRTELTVVHDKYGLKEKVIHY
YFKKWPDHGVPEDPMHLIMFVKKVKAEKRPSYSPIVVHCSAGVGRTGTFIGLDLIMQRLKSESKINIFETVKKLRFQRMKMVQT
QQQYTFLYACTYELVKHKIPRAALKMDGRPKSVTVPAIPSPKKVSFPDVDVGSEYVSSAPITDLDDGRPIVQLPSRFSGLRRNS
PPGENDNPTSSSNM

PORIFERA

>Sponge XP_003383454.1 XP_011409595.1curated by ptotein profofiling [Amphimedon queenslandica]
MFFIIDILIKNFYLGFAPDVINFTVNSGNSTSLALSWQLRYPLVPPDMYYIAYNYTELSGANPRSDNSTIMIDEDDVTTTTDYI
LYTLDDLLPFTSYYVTLFPQYGEEEGEGLSDSGNTGEGMM**(8)**SSQVNDTNVDIDRDNMRLNISWAPPSRPAGNILRYEVTLSI
RGKICLTINTTELSVIAEFKFNVTYTIRITPFNSFGSGTTRELDMFATPEGA**(7)**PTPPLNFTLTELSLSSLKASWEEPSTLNG
VLANYTVYCNLSSLQFYSQQLMLSFNAPPTTVDPDSTTANITGLYPFTNYDCYATASTGGGESSRSNNDSARTSEEEPAGPPEG
FNVTDVTATSVSLEWRRPSVPNGVILHYFLQYSAYNVTIPVTFNTSSDEYSVTSYTVESLNEYTNYTFTISAVTSGGAGPLATT
STRTSEAVPGTNLDGFNTSSMSLRITWTELLEDDQNGVIIGYNISYFSLPAVGQPINNFTSDTSYNISGLDVYTDYNVSVAAYT
SAGTGPFDSVIRRTDSTV**(6)**PTSPSDVSYINISSTSIEVSWNPPTDFNGPNEGYVITYIRLESDTESMSTNRLTGTSFVIENL
EKYEQYSVTVVAFTDKGPGASSDVLSVLTDEDL**(5)**PGPPSNVSTMSTNTSISITWSPPLDPNGLLLSYSINVTLNSTYAQYLS
FDVMTMSVSQNIFSYTLFDLLPFAGYDISLQASTSVGLGTPAIETETTLQAA**(4)**PAAPVANPMASPVSSTAVNVSWLPPNLSN
WNGLITNYTIEYRTNDEYIRPSIEVTATLSSFANSDDPRAATTPLQLESIIIPSLHEFVNYSFIITLSNSIGQISSLPVHIVTL
EST**(3)**PSGPPLDVSVIVDSPSTARISWSPPMYIDRNGIIVNYTVRIITTVRGTIRETNISNVSGNYYDASDLPQFASFNVTVA
AATSVGLGPYSPSVSNMTFEGV**(2)**PSAPDPVIVKRRNDTAIQVNWTRPAEPNGIILGYLIYYIGTKNNTGTEYSNINVLIIIN
VTDPNTLSYLITNLLADTQYFINVTAYTSAGLGAVGDGDSILLPTGRP**(1)**PTVPPNVTIGTPPTGGTSDPTTATTIRIEVTIP
EELNANGPLTRIRILIRIFLSRNDTISTWYESQKFPNSVAPPWQATQLPLNQGNRRKRQAGGETVAETIGTNNSCGPNDIVCNG
PLKPGTQYQFKYRVYNSDDDDSYVESQYSGPIRTG**D**PIAEENNTGTTIVIAVVVVLLVIILLIAILVIVVIIVLKRRKRKAYSF
A**A**SFNGKEELITAGEGKGATLSKAIQLTPTDPTIVPLTENTIVNPTAVRTASVKATPNSRKVYL**RDFPSYVEDMLNDAGFKFSE**
EYEKVSAVGLDHSKDASLLPENRAKNRYTNILAYDHSRVKLESIDDEPGSDYINANYIPGYRMRRAYIATQGPLPSTFDDFWRM
TWEQNSHVIVMLTQLVERGRTKCHRYWPGAQPEVYGEINVDMLSETEKSDWIVRKFKITKEKRSRTITHYQFVSWPDHGVPDEA
GPALDFVREVHEVASSAFGPVIVHCSAGVGRTGTFIALGTLLQHIKDHDWVDLFGLASEMRQHRNHMIQTEPQYVFIHKAMVDA
CKSSMSKFGGGIKVLPSFPI**Y**S**N**IGPASQLSDF**Y**GDMLSEEAS**Y**QYQKTSGNGKTYDESTF

PROTISTS

>capsaspora PTPc1 Capsaspora owczarzaki ATCC 30864]
PTPcapsaspora .2| EFW39745.GENScan ref|XP_004365141.1|

MKPRLLLLAVALLAAVGSVRGADLDITFSAATGNSITLNWVAYGSFASPMEYRVTRTPGSATPVYIGTALTFADSSLTKNTAYT
YTVSVYSNSGAGGTLVGATAAPPSQTTANDVTLNADSILMYATSNVNEYVLSWPSLVANTGASTAGPPATITYTVEYKKVASGS
YIGWTGYSSGTSAPALVLAPGTNYNVRITVSSGILGTASVNQVFTGRTYSVAPVIGTALAAGTITATQVPLSWTVTSSGQESNL
ALAQVLSRNGVDLTTLPAGTASYTGSFPQPFSFTDSGLSPYTPYTYSIRATTVAGNSTSSPVSALSVTTASAPPTVAFLTTAPY
ITQNSVT(0)DLDPCTLYSYTLTATTNDGQTFTTSAKSFTTLADKAVLSPTVTSLNYTYNAFSFAWTNSALSPCPGSGGTSGYQ
LSLSVNSGAATLVNPTTTTSYSLSAGVLPSSTYAFYLRFTNTNNNVSADALLTTFTTFANTPTVTALGVTANSSNSLTFQWTGT
ANGGGPLFYKVDRTSPSVLSIKNFADSLTSATDNTGLLPFTDYTYSVQARNSQTPTANLSTVATATFKTAASQATFSSSTVSST
PAANSILFSWTAATWNSLTGDYSWTISGNPTPSPSSGVVTTNSVTVSNLRAYNQYTFTVYARSGSGNTYDSAVLTASNIQTLAA
APTIVAGQFTSTGQTYNSVSLEWNGVLTLINGPTVNSASQWLVERRLNNPQGSFVSVGTFAAATLSTTDNDASLAPYTVYDYRL
TVRNDQYSTTATLSSIRTAAAPPTIDPSQIEPSFSANGPTSYAVTFDLAGAYYRGTPASVSATFLLRYKTTGSGSFTSAPTFTT
DTTTVTMQASQVYDLEWTITNAGPLSSTPSTGSVSVPALAPYFNAPTITTSDVTTTTADISWPAPTTKNGNDPIVYWVQYTAGQ
PNPGNQTIQTSSDILQTTSTTVSGLYPGVIYTWTVSAKNANSGFSSPFSTVSSLRQDPADPIWQTAPTLSASGQTTSAFVFNWA
APVYAQDDPNNLKYRVVATQITASPAFPDPTLFSKTVSINSAAVTVAAASNTGYEPYVQYNVTVHVKSSTDNFVPSNTFLTVTT
AGSKPSWGALTQSSLTGDRSSRQITLEWPDVPYPQDASTLTYSVSRGGTTIISGLTTSSYVDTGLTPFTDYAYIITATNSKGST
ALGTAVTFKTTEATPLFAGSETLTMTPNANGASITYAAPNFPYGTLSGYTVSVVDTSFTDAHVTPTQSSNSAANVFTRTITSLF
PFRTYAVTIQATNGAGTSPPSDSRTGTFTTSPSAPILDESLLDAQVTSNTSITVSWKGPQPWERNSVLSSYRVECYMQEEDGAW
TFVNAPTRTPLPADNAAYNTATQQSYQEVFSGLLPYRNYTFNVFVIGSSLTSSAIEVLARTLPGLPPTPLQATVIPISDTQARV
SWPAVDDINGPIVSYVVEGQRLLPGYNETWPVPVVYAELTNGRNVTTVSGLAAESKYRFRVRTFTAAGFSLGPFGTSATTLEAA
AGSQGWVAAVVLIPLILIALVALLLVRRRQGKPMPWEPLQQRWKQRQNKDANNSMMLMGVQHQQDNMAGMPSRIVPAEELADCV
AQLHANSDLGFAEEYEALNVNEEYATNAALMPANKSKNRYANILPYDHSRVRLSVIPGVEGSDYVNANYIDGYRKSHAYIACQG
PLPDTFDDFWRMVWEQRSAVIVMVTNEEEKGRVKCHRYWPDDSEVVFGDVEVRMTRQEELSEFITRVFSLKNIRTGETRTVHHL
QFTGWPDHGVPHSTSSLIKFVKQAKAVQPNDAGPMVIHCSAGVGRTGTFIVTDMSLERTKVENNVDIFGCVSALRRNRMYMVQT
EEQYMFIYQTLADAVANGDSEVSARDLAQHVARLAQHNKATDSSGFEEEFRLFQQPDKKKPTTDAATLSANKSKNRFATLLPCM
HLNDHTRVKLNAIPGVNGSDYINASYIDGFRRKRAYIATQGPLPATVNDFWRMIWEQRVEGIVMISREEESGRVKCHRYWPDQD
VLQAGAIEVTLLSQLDRTNYILRRFSITNETTKETRQIDQFQYLAWPDTSLPASTANFIDFMTQVKIWQSNANPNNPVVVHCST
GAGRTGAYIVIDMAMDRLASEGVVDVFHTVKMLRSQRPNMVQALEHYEFVYSAVLEYIRSNSG

DNA sequences catalytic domain

>Spongue

AGGGATTTCCCAAGCTACGTTGAGGACATGCTTAATGATGCTGGATTTAAGTTCTCTGAGGAATACGAGAAAGT
ATCAGCTGTTGGACTAGATCATTCTAAAGATGCGTCACTCCTTCCTGAGAACAGAGCTAAGAACAGATACACTA
ATATATTGGCCTATGATCACTCGAGAGTCAAACTTGAGAGCATCGACGATGAACCAGGATCAGATTACATCAAC
GCAAACTACATACCAGGTTACAGGATGCGTAGAGCCTATATTGCAACACAAGGACCTTTACCATCAACCTTTGA
TGATTTCTGGAGGATGACATGGGAACAGAATTCGCACGTTATTGTAATGCTGACTCAGCTCGTTGAAAGAGGAA
GGACGAAATGTCATCGCTATTGGCCCGGAGCACAGCCGGAAGTTTATGGAGAAATAAACGTAGACATGTTAAGC
GAGACTGAGAAGAGTGACTGGATAGTGCGGAAGTTCAAGATCACAAAAGAGAAGAGAAGCCGTACAATAACACA
CTACCAGTTTGTATCATGGCCTGATCATGGAGTCCCCGATGAAGCCGGACCTGCTCTGGATTTTGTACGGGAAG
TACATGAAGTAGCTTCATCAGCTTTCGGACCTGTTATAGTCCACTGCAGTGCTGGTGTAGGTCGTACAGGAACA
TTCATAGCACTGGGTACACTGCTCCAGCATATTAAGGACCATGACTGGGTTGACCTCTTTGGACTGGCTAGTGA
AATGAGACAGCATCGTAACCACATGATCCAGACCGAGCCTCAATACGTCTTCATTCACAAAGCAATGGTTGATG
CTTGCAAGAGCAGCATGA

>Nematode.2

GAAGACTTTGCCGACCACGTGAGACTAATGTCAGCGGACTCTGACTTTAGATTCTCAGAAGAATATGATATGAT
GCGAAATGTTGGAGTTGGACAATCTGTAGCTGCATCGGAATTGCCAATTAACAGACCGAAAAATCGATTTACAA
ACATTCCATCCTACGATCACTCGCGTGTTAAACTATCGAATCCAAATAATATCGAAGGTGGAGATTATATAAAT
GCGAACTATGTACCTGGATTCTCATCACGTCGAGAGTTTATTGCTGCACAAGGTCCTCTACCAACAACACGTGA
TCATTTCTGGCAGATGACGTGGGAGCAGCAGTGTCCAGCAATTATAGCACTCACGAAATGTGTTGAAAAGGGAA
GAGATAAATGTCATCAATATTGGCCTGATCATGAGAATGTACCTGTTTTGTATGGAGATATTGAAGTGACAATA
GTAGCAGAAAAGGAATTCGATGAATTCGTGATCCGAGATATTCGACTTGAAAAATCAGGTCCCGATGGTCGCGT
AACACGATTTGTCAGGCACTGGCATTATATGGCATGGCCTGATTTTGGAGCCCCGTCTCATCCAAACGGTATCA
TTCAATTCTCAAGAATGTTCCGTCACCATCTTCCACATTCACCTCATAATGCTCCAACGATAGTGCATTGCAGT
GCTGGAGTTGGTCGTTCTGGAACATTTATCAGTATTGATCGTCTCCTACAATCAAGTTCATTTGGTGATCCAAT
TGATGTATTTGGAACTGTTTGTGAAATGCGATACGAGAGATGTCAAATGGTACAGAATGAGCAACAATACATAT
TCATTCACTATTGTATCCTTCAAGTTCTACAAGGATCA

>annelid

GTGGACTTCGTGCAGCATGTTGCAAGCATGGCAGCTGACAGTGAATTCAAATACTCAGAGGAGTATGAGGAGTT
GAGCTTAGTGGGCAAAGATCAGCCTAAGGAGGCGTCCGACCTGCCCTGCAACCGTTCCAAGAACCGCTTCATCA
ACATCCTACCATATGACCACAGCAGGGTGAAGTTACTCCCAATTGATGACGAAGACGGCGGAGATTATATCAAT
GCTAACTGGATCCCTGGCTACAATTCGCGTCGAGAATACATCGCATCACAAGGACCTCTGCCATCCACTCGAGA
TGACCACTGGCGCATGATTTGGGAGTGCAATTGTCAGTGTATTGTAATGCTCACCAAATGCGTGGAGGCAGCAA
GACAACGGTGTGACCATTACTGGCCTATTGATTCTGAGGCTATCTACTACGGTGATCTTCAGGTGCAAATTCTG
AATGAAACGAAATCCACAGATTGGAGCATCTCAGAATTCATCATTTCCAAAGGCGATCAATCACGACGTCTGAA
GCATTTTCACTACCACTCTTGGCCCGACATGAAAGCCCCGAATAAAACGGGAAGCATCGTGCCCTTCGTGCGCC
TCATTCGTGCCAAGGTTCAGTTGGATGGTGGACCGCTGCTCGTGCACTGCAGTGCTGGTGTGGGTCGCACTGGA
ACCTTTATAGCTCTGGATCATTTGATGCAGCACATCGAAGAGCATGATGACATTGACATCTATGGAACCGTTTA
CCAGATGCGCAAACATCGGTGTATGATGGTCCAGACTGAGTCACAATACATCTTCATACACAAGTGCTTGGAGT
TCGTTCTTGAAGGGGG

>FlyDPTP4e

AAAGACTTCTCCGAGCACTACAGGATCATGTCGGCCGATTCGGACTTTCGTTTCAGCGAGGAGTTCGAAGAGCT
GAAGCATGTGGGCCGCGATCAGGCCTGCAGCTTTGCCAATCTGCCCTGCAATCGGCCCAAGAACCGATTTACCA
ATATCCTGCCCTACGACCATTCCCGTTTTAAGCTCCAGCCAGTGGACGACGACGATGGTTCGGATTATATTAAT
GCGAACTACATGCCGGGACACAATTCGCCGCGCGAGTTTATCGTCACCCAGGGACCGTTCCACTCGACGCGCGA
GGAATTCTGGCGGATGTGCTGGGAGAGCAACTCCAGGGCGATTGTCATGCTGACGCGCTGCTTTGAGAAGGGTC
GCGAGAAATGCGATCAGTACTGGCCCGTGGATCGGGTGGCCATGTTCTACGGGGACATCAAGGTGCAGTTGATT
ATCGACACGCACTACCACGACTGGAGCATATCAGAGTTTATGGTCTCAAGGAACTGCGAATCGCGTATAATGCG
TCACTTTCACTTCACCACATGGCCGGACTTTGGGGTTCCAGAGCCGCCGCTGTCGCTGGTTCGCTTTGTGCGCG
CCTTCCGCGATGTCATCGGCACAGATATGCGTCCCATCATCGTCCATTGCAGCGCTGGAGTGGGCAGATCGGGC
ACGTTTATAGCCCTGGATCGCATCCTGCAGCACATTCACAAGTCAGACTACGTGGACATTTTTGGCATCGTGTT
TGCCATGCGAAAGGAGCGCGTTTTTATGGTGCAGACGGAACAGCAGTATGTGTGCATCCATCAGTGTCTGCTGG
CGGTGCTGGAGGGCAAG

>Fly PTP10D

AAGAACTTTGCCGAGCACTATCGCCTAATGTCCGCCGATTCGGACTTCCGTTTCAGCGAGGAATTCGAGGAACT
GAAGCACGTTGGCCGGGATCAGCCGTGCACTTTTGCCGATTTACCCTGCAATCGTCCCAAAAACAGGTTCACCA
ATATACTGCCCTACGATCACTCACGTTTCAAGCTTCAGCCGGTGGACGATGATGAGGGTAGTGATTATATCAAT
GCCAATTACGTGCCGGGTCACAATTCACCGCGCGAGTTCATCGTGACCCAGGGACCATTGCATTCGACACGCGA
TGACTTCTGGCGAATGTGCTGGGAGAGCAACTCGCGGGCCATAGTCATGCTGACCAGGTGCTTTGAGAAGGGGC
GCGAGAAGTGCGACCAGTATTGGCCAAATGATACGGTGCCCGTCTTTTACGGTGACATCAAGGTGCAGATACTC
AACGACAGTCACTATGCCGACTGGGTGATGACCGAGTTCATGCTATGCAGAGGCAGCGAACAGCGCATCCTGCG
ACACTTCCACTTCACCACCTGGCCGGACTTCGGTGTTCCCAATCCGCCACAGACACTGGTGCGCTTTGTGCGCG
CCTTCCGCGATCGAATTGGTGCGGAACAGCGACCCATTGTGGTCCATTGTAGCGCCGGTGTGGGAAGGTCTGGC
ACCTTCATCACCCTGGATCGCATCCTGCAACAGATCAACACGTCTGACTATGTGGACATATTTGGCATAGTATA
TGCCATGCGCAAGGAGCGCGTTTGGATGGTGCAGACGGAGCAGCAGTATATCTGCATCCACCAGTGCCTGCTGG
CGGTGCTCGAGGGGAAG

>Fly.Ptp52F

AAGAACTTTGCCATCTTTTATACCGAAGTTGCCAAGCCGGAGAAACTGGCCAGGGAGTTCAAGGAGATCACCGT
TGTGGCCTTGGAGCTCAGCTATTCCGCCTCCGAATTGGGTTGCCACAAGAATCGCTATGCGGACATATTTCCTT
ATGACAAGAATCGCGTGATTCTGGACATCGATGCGGAGGGATCGGACTACATCAATGCCTCCTTCATAGATGGT
CACACGCGTAAGAAGGAGTACATAGCCACCCAGGGACCAAAACCAGAGAGTGTGATGGACTTTTGGCGCATGAT
CCTGCAGTACAACGTGCGGGTTATTGTTCAAGTTACCCAGTTTCGCGAGGGCAATACGATCAAGTGTCACGAGT
ACTATCCGTATAATGTGCGTGGATTGACTGTGACTATCAAGTCCAAGGAGGTTCTGGAGCTGTACGATCGAACT
GAGTTGACTGTTGTGCACGACAAGTACGGACTGAAGGAGAAGGTGATCCACTACTATTTCAAAAAGTGGCCCGA
TCACGGCGTGCCCGAGGATCCCATGCATTTGATTATGTTTGTGAAGAAGGTGAAGGCGGAGAAGCGACCCAGTT
ACTCGCCCATTGTCGTCCATTGCAGTGCGGGAGTGGGCAGGACGGGCACGTTTATTGGCCTGGATCTGATCATG
CAGCGATTGAAGAGCGAGTCGAAAATCAACATTTTCGAAACGGTCAAAAAGCTGCGATTCCAGCGCATGAAAAT
GGTGCAAACGCAGCAGCAGTACACATTCCTCTACGCCTGCACCTATGAGCTGGTCAAGCATAAGATA

>AcornWorm

AAAGGTTTTCGTGATTATGTGAATGAATTAGACGAAGACGACAGGAAAGGATTTGAAGAGGAATATGACGATCT
CCGAAAACTAGTAGTGAGACAGCCAACATCGGTGGGACAGCGAATGGAACACAGATCAAAGAACAGATACACTA
ACATTGTACCCTATGATAATAACAGAGTTATATTGTCAGGCAGTGGGAATAGTTACATTAATGCCAGTTATATT
TCGGGTTCCAAAGGCTCACGATCGTATATTGCAACACAAGGACCACTGGAAAGCACTTGTGGCGATTTCTGGAA
AATGATTTGGCAACAACGCGTGACCACTGTCGTCATGATGACGCAATGCAACGAACTTGGAAAGAGTAAATGTC
ACCACTACTGGCCACGAGATACCCAGACACAGATTTCGCACAGTGATCTCACAATAAGACTAACATCTGAAACC
AGGCTTCCCGATTGGATAATACGAGACTTTTCAATAGAATCTAATGGTGAAATTCGTGCATTACGCCAGTTTCA
CTTTACATCGTGGCCAATCACAGGGACGCCGTACGATGCTGACCCATTGATCCGATTTATTGAAGCTATTCGAA
TTCAAGTCTTGCCAAATAGCGGACCAATACTAGTACACTGCAGTGCTGGTGTTGGTCGAACAGGTGTCTTTATT
GCATTGTATCACCTTCTAGAATATTTCTACACTATGATCCAGGTTGATATATTTGGTAAAGTCATAAAAATGAG
GAAACAAAGACCATTTATGGTTCAGACACAGGGTCAGTATGAATTCCTCTACTATGCAATTCAACAACATATAA
AAGAGAAG

>SeaUrchin.1

AATAAGTTTGAGGGGCATTACGGCCGGATGAAGGCTGATTCAGATTTCAGATTCACAGAGGAATATGATGAAAT
CCGTCTAGTGGGTAAGGACCAAGCCATTGTATCAGCTTTAGAGATGGTGAACAGAGCCAAAAACCGATTCACTA
ATATCCTACCATATGAGCATTCCCGCGTTAAGCTTGCGGCTCTCGCCGACGATTCAGACACGGACTACATCAAT
GCAAATTACATCCCCGGTTACAACTCGCCACGCGAATTCATGGCTTGCCAGGGCCCGTTGCCGGGGACTGTGGA
TGATATGTGGAGGATGATCTGGGAGAAGAAGACCTCCATCATCGTCATGCTGACGCAGCTCGTTGAAAAGGGCA
AGATAAAATGTCATGAATATTGGCCTGCCGATTACAACCCCGTCACATATGGATCGATACAGGTCTCCGTACAA
GCACTTCAGCAGTATGACCACTGGGTCATTAGGGAGTTCAGTATATCACAGGGAGATGAAATTCGAAAGCTTAC
GCAGTATCACTTTATGTCATGGCCTGACCACGGGGTCCCGGAGAAGACCTGGACCATGCTGGACTTTGTCCGAA
CGGTCCGAGAAGCAATTCAGAAAACAGCCAGCGATCGGCCTATTGTAGCGCACTGCAGCGCCGGTGTTGGTCGG
ACAGGGACGTACATCGCTCTCGATCGGTTGATGCAGGCCATGCAGGAGAACGACTACATCGATATCTTTGGGAT
TATATGTGAGATGAGGATGCAGAGAAATCACATGGTCCAGACGGAAAAACAGTACATCTTCATTCACGAATGTG
TGATGGATCTCTTGCGGAGAGG

>SeaUrchin.3

AGGAAGTTCCCTGACCATGTCAATCGCATGAGTGCTGGCAACAATACTGGATTTGTGGAAGAGTACAATTCTCT
ATCAAATGTGGGCAGAGACAAGAGCACCGATGCTTCAAGGCTTGCTGTCAATGCAACCAAGAACAGATACAGGA
ACATCCTACCATTTGATGAGACTGCCGTTCATCTACAAGATTTGGAGGAAGGACAGACCAGTGACTACATCAAT
GCAAACTACATCCAGGGCTTGCACTCTATGAAGGAATACATTGCTTCCCAGGGCCCCGTACCAGACTCTGTCAA
CGATTTCTGGGAGATGGTGTTTGAGAATAAGAGCACAACCATCGCAATGATCACTGGGCTTGTTGAAGGAGGAA
AGACCAAGTGTGAGCACTACTGGCCAGATGACGATACCCCAGTGAACTACGGCAGTGTGACGGTCACCATGACC

CATACACAGGAGATGGAACAATGGACCGTCAGGTCATTCCTGCTAGAGAAGGGCATGCAACAGTTTGAGACGCA
TCAGTACGCCTTCAAGGGCTGGGTCGATCATGATGTACCCAACAATGCTCGTCCTATGATTGAATTCATCCGGA
CCATAGACGTCGCCCATGATGCTAATCTAGGACCCATCACCGTGCACTGCAGTGCTGGTATTGGTCGAACCGGT
GTGTTCATTGCCCTTCACAAGCTGATCAAGCAGGTTGAGACTTCCAAGCCCAATGACTACATCGATGTGTGCGG
CACTGTGGCACGCATGAGGGAGCAACGTTTCAACATGGTCCAGACAGTCAAACAATACATGTTCATCCACCAGT
GTCTGCTACTTCTTATGAGA

>SeaUrchin.4

AATATGTTTGCCCAACACGTTGCCAAGATGTCTGCTCGCAACAATGCTGGATTCAGTGATGAATATAATAATTT
GCCAGCGATGGAAAAGACTCGAGCGGTGACTGCGGCTCGACAGGCCAGAAATGATGATAAGAACAGATACAGGA
ACATCTTGCCTTATGACTACACCAGAGTCCAACTGAAAGGAGTAGCCGGTACCGATTACATCAATGCAAGTTAC
ATTAAGGATGATCATGGACAGAAGAAGTACATTGCGACCCAAGGACCCTTGCCAAACACCATTGAACACTTCTG
GGAGATGGTTTGGGAAAACCAGACCAGTACCATTGTGATGATGACTGCATTGGTGGAAGGAGGAAAGACCAAAT
GCGAGCACTACTGGCCAGCTGGAGAAGAGCCTCAACTGCATGGCAACGTGACTGTCACCCTTGTTGGCAGTAAC
CAGACCGATAACTTCATCGAACGCACACTTCTCCTAGAAAAGGAGGGTACAGAACGCACCGTGACCCACTACCA
GTACCTCGCCTGGACCGACCATGGCGTGCCCGAGTCCACTGCCCCTCTAGTGGGGCTACTCCGTCAGGTCAAAG
TGACTAACCAGGAAGATGGGGCAGCAACCGGTCCGATCGTCGTCCACTGCAGTGCTGGTGTTGGCAGGACAGGA
ACGTTCATCGCTGCTGATATGTTAATGGATGCCATACAACGATCCACTGCTACCGACTACATTGACGTTGCTGG
TACTATAGCGAAGATCAGAGAGCAAAGAGCCCTCTTGGTTCAAACACTGTACCAATACATCTATCTTCATCGTA
CCGTGCTTTCCTTGATCGAGGAGCAA

>Ciona

GACGAATTTCCAAATCACGTTTCATCCTTAAGTCAAAATAAAAATAAAGGATTCAGTCTTGAATTTGATGATAT
TAGGGGAATTCCTTATGCTGGTACTACGGCTATTGCCGAAAACTCTTGTAATAAAACAAAGAACAGAACAACGA
AATTAGTCCCATTTGACCACTGTCGGGTTAAAATTGAAGGAATCCCAGCGATACAAGGTTCAAATTATATCAAC
GCATCGTACATACCAGGTCTAGACAGTCCGGAGCAATACATAGCAACACAGACTCCGTTAGACCACACAAAGAA
AGATTTCTGGCGAATGCTTTGGGAGACGGGCTCAACTAACATTGTTATGTTGTGCGGTTCTGTGAATGCAGGCA
AAAAACGCTGCGACGAGTTCTGGCCCAAGAAACAAACTGAATATTTTGGAAACCTCGCTGTTCAGATGAAGGAA
GAGATACGACACGATGAATGGATTATTCGGCAATTCATCGTCACAATGCGAGACAAAGTTCGACACGTGACACA
ACATCACTTTATAAAATGGCCGCAACTGGAACAAGCTGAAAACTCTTTGCCACTGGTTCGATTCATAAAGAACT
ACCGATTGTTACGGGACAGAAGCATCAATCCAACTATTGTCATGTGCAGCAACGGGAGTGGTCGATGCGGAGTC
TTCATCGGATTACTTCGAATATTAGACAACAACGGACGTGACGTCGATGTGTTCGGAACGGTTGCTGCATTGAG
GAAATACAGACCATATATGGTGCAGACACTGTCCGAGTACATATACCTGCACCAATGCGTGCTCAAGTTTATCG
ATGACGTCAC

>PTPRQhuman

AAATCCTTCCTGCAACATGTTGAAGAGCTTTGCACAAACAACAACCTAAAGTTTCAAGAAGAATTTTCGGAATT
ACCAAAATTTCTTCAGGATCTTTCTTCAACTGATGCTGATCTGCCTTGGAATAGAGCAAAAAACCGCTTCCCAA
ACATAAAACCATATAATAATAACAGAGTAAAGCTGATAGCTGACGCTAGTGTTCCAGGTTCGGATTATATTAAT
GCCAGCTATATTTCTGGTTATTTATGTCCAAATGAATTTATTGCTACTCAAGGTCCACTACCAGGAACAGTTGG
AGATTTTTGGAGAATGGTGTGGGAAACCAGAGCAAAAACATTAGTAATGCTAACACAGTGTTTTGAAAAAGGAC
GGATCAGATGCCATCAGTATTGGCCAGAGGACAACAAGCCAGTTACTGTCTTTGGAGATATAGTGATTACAAAG
CTAATGGAGGATGTTCAAATAGATTGGACTATCAGGGATCTGAAAATTGAAAGGCATGGGGATTGCATGACTGT
TCGACAGTGTAACTTTACTGCCTGGCCAGAGCATGGGGTTCCTGAGAACAGCGCCCCTCTAATTCACTTTGTGA
AGTTGGTTCGAGCAAGCAGGGCACATGACACCACACCTATGATTGTTCACTGCAGTGCTGGAGTTGGAAGAACT
GGAGTTTTTATTGCTCTGGACCATTTAACACAACATATAAATGACCATGATTTTGTGGATATATATGGACTAGT
AGCTGAACTGAGAAGTGAAAGAATGTGCATGGTGCAGAATCTGGCACAGTATATCTTTTTACACCAGTGCATTC
TGGATCTCTTATCAAATAAG

>PTPRQ.mouse

AAATCCTTCCTGCAACATGTTGAAGAGCTTTGCACAAACAACAACCTAAAGTTTCAAGAGGAATTTTCGGAATT
ACCAAAATTTCTTCAAGATCTCTCCTCAACTGATGCTGATCTCCCTTGGAACAGAGCAAAAAATCGCTTTCCCA
ACATAAAACCATACAATAATAACAGAGTGAAGCTGATAGCTGATGTAAGCATCCCGGGATCAGATTACATTAAT
GCCAGCTATGTTTCTGGATACTTATGTCCCAATGAGTTTATTGCCACCCAAGGCCCATTACCGGGAACAGTTGG
AGACTTTTGGAGAATGGTGTGGGAAACCAGAGCGAAAACATTGGTAATGCTCACACAGTGTTTTGAGAAAGGAC
GGATCAGATGCCATCAGTATTGGCCCGAGGACAACAAGCCAGTGACAGTCTTTGGAGACATACTGATTACAAAG
CTTATGGAGGACATTCAGATCGACTGGACCATCAGGGATCTGAAAATTGAAAGGCATGGAGACTGCATGACTGT
CAGGCAGTGTAACTTCACGGGTTGGCCTGAGCATGGGGTTCCTGAAAACACCACCCCTCTAATTCACTTTGTGA
AGTTGGTACGAACAAGCAGAGCACACGACGCTACCCCTATGGTTGTGCATTGCAGCGCTGGGGTTGGAAGGACG
GGAGTTTTCATTGCTCTGGACCACTTAACACAGCACATACATGACCATGACTTTGTGGACATATATGGACTAGT
AGCTGAACTGAGGAGTGAGAGAATGTGCATGGTGCAGAACCTGGCACAGTATATCTTCTTACACCAGTGCATTC
TGGATCTTTTATCAAATAAA

>PTPRQ.chiken

AAATCCTTCCTACAACACGTTGAAGAGCTTTGTACAAACAACAACCTAAAGTTTCAGGAAGAATTTTCGGAACT
TCCTAAGTTTCTTGAAGACCTTGCTTCAACTGATGCTGATCTGCCTTGGAACAGGTCTAAGAACCGTTTCCCAA
ACATTAAGCCATATAATAACAACAGAGTAAAGCTGATGCCTGATGCTGGTATTCCTGGATCTGACTACATTAAT
GCCAGCTATGTATCTGGCTACTTATGTCCAAATGAATTTATTGCAACTCAGGGACCGTTACCAGGAACAGTGGG
TGATTTCTGGAGAATGGTGTGGGAGACTAGAGCAAAAACTCTAGTGATGCTTACACAGTGTTTTGAAAAAGGAC
GGATAAGATGCCATCAGTACTGGCCAGAAGATAATAAACCAGTGACTGTGTTTGGGGATATAGTAATTACTAAA
TTGGTGGAAGATATTCAAATAGACTGGACCATCAGAGATCTAAAAATTGAAAGGCATGGAGACTGCATGATGGT
CAGGCAGTGTAACTTCACTTCATGGCCAGAACATGGAGTCCCTGAAACTACTGCACCAATTATCCACTTTGTGA
AACTTATCCGTGCAAGTCGAGCACACGATAATACGCCGATGGTGGTTCACTGCAGTGCTGGTGTTGGAAGAACA
GGTGTTTATATTGCTCTTGACCATTTAACGCAACATATAAATGACCATGATTTTGTGGATATCTATGGCCTTGT
AGCTGAGCTAAGAAGTGAAAGAATGTGCATGGTGCAGAATCTGGCACAGTACATCTTTTTGCATCAGTGTGTCC
TGGATCTGCTGACAAGCAGG

>PTPRQ.fish

AAATCGTTTCTTCAGCATGTGGAAGATCTTTGTGCCAACGACAACGCCAAGTTTCAGGAGGAGTTTGCTGAGTT
GCCGAAGCTGCTGCAGGATCTGGCCACATCAGACGCTGACCTGCCTTGGAATCGCTCCAAAAATCGCTTCACAA
ACATCAAGCCCTACAACAATAGCCGAGTGAAGTTGCTGTCTGAGCCAGGAATGCCGGGTTCTGACTACATCAAT
GCCAGCTTCGTCTCTGGCTATCTGTGTCCTAATGAGTTTATAGCCACTCAGGGCCCTCTGCCGAGCACCGTCGC
AGATTTCTGGAGGATGATTTGGGAAACTGGGACTAAAACCATCGTCATGCTTACTCAATGCTTTGAGAAGGGCC
GGATCCGCTGTCATCAGTACTGGCCGGAGGACAATAAACCAGTCACTGTTTTTGCTGACATCATAATCACTAAA
CTCACAGAGGACGTGCGTCCTGATTGGACGGTGCGGGCACTCAAAGTGGAGCGGCATGGCAGCTACATGATTGT
CCATCACTTTAACTACACCTCATGGCCAGAACACGGCGTTCCCGAATCCAGCTCCACACTTGTCCAGTTTGTCA
AAGCCGTTCGCTCCAACAGAGGCCATGAAAACACTACCATCGTGGTCCACTGCAGTGCTGGTGTGGGCAGGACT
GGAGTGTTTATAGCACTGGATCATCTCATCCAGCATTTGCGAGATCATGAATTTGTTGACATATACGGCCTGGT
CGCAGAGCTGCGCAGTGAGAGGATGTGTATGGTGCAAAACCTGGCTCAATACATGTTTCTCCATCAGAGCACTC
TGGATCTGCTGTCGGCCAAA

>PTPRB.human

AATCAGTTTGAAGGGCATTTCATGAAGCTACAGGCTGACTCCAACTACCTTCTATCCAAGGAATACGAGGAGTT
AAAAGACGTGGGCCGAAACCAGTCATGTGACATTGCACTCTTGCCGGAGAATAGAGGGAAAAATCGATACAACA
ATATATTGCCCTATGATGCCACGCGAGTGAAGCTCTCCAATGTAGATGATGATCCTTGCTCTGACTACATCAAT
GCCAGCTACATCCCTGGCAACAACTTCAGAAGAGAATACATTGTCACTCAGGGACCGCTTCCTGGCACCAAGGA
TGACTTCTGGAAAATGGTGTGGGAACAAAACGTTCACAACATCGTCATGGTGACCCAGTGTGTTGAGAAGGGCC
GAGTAAAGTGTGATCATTACTGGCCAGCGGACCAGGATTCCCTCTACTATGGGGACCTCATCCTGCAGATGCTC
TCAGAGTCCGTCCTGCCTGAGTGGACCATCCGGGAGTTTAAGATATGCGGTGAGGAACAGCTTGATGCACACAG
ACTCATCCGCCACTTTCACTATACGGTGTGGCCAGACCATGGAGTCCCAGAAACCACCCAGTCTCTGATCCAGT
TTGTGAGAACTGTCAGGGACTACATCAACAGAAGCCCGGGTGCTGGGCCCACTGTGGTGCACTGCAGTGCTGGT
GTGGGTAGGACTGGAACCTTTATTGCATTGGACCGAATCCTCCAGCAGTTAGACTCCAAAGACTCTGTGGACAT
TTATGGAGCAGTGCACGACCTAAGACTTCACAGGGTTCACATGGTCCAGACTGAGTGTCAGTATGTCTACCTAC
ATCAGTGTGTAAGAGATGTCCTCAGAGCAAGA

>PTPRB.mouse

AATCAGTTTGAAGGGCATTTCATGAAGCTGCAGGCAGACTCCAACTACCTTCTATCCAAGGAATATGAGGACTT
AAAAGACGTGGGTAGAAGCCAGTCATGTGACATTGCCCTCTTGCCTGAGAATCGAGGGAAAAATCGATACAACA
ACATATTGCCTTATGATGCCTCAAGAGTGAAGCTCTGCAATGTCGATGACGACCCTTGCTCTGACTACATCAAC
GCCAGCTACATCCCCGGTAACAACTTCAGACGAGAATACATCGCCACTCAGGGACCGCTTCCAGGCACCAAGGA
TGACTTCTGGAAGATGGCGTGGGAGCAGAACGTTCACAACATCGTCATGGTGACCCAGTGTGTTGAAAAGGGCC
GAGTGAAGTGTGACCATTACTGGCCAGCAGACCAGGACCCCCTCTACTACGGTGATCTCATCCTACAGATGGTC
TCGGAGTCCGTGCTCCCCGAGTGGACCATCAGGGAGTTTAAGATATGCAGTGAAGAACAGTTGGATGCACACAG
ACTCATCCGTCACTTTCACTACACGGTGTGGCCAGACCATGGGGTCCCAGAGACCACCCAGTCCCTGATCCAAT
TTGTGAGGACAGTCAGGGACTACATCAACAGAAGCCCCGGGGCTGGGCCCACCGTAGTGCACTGCAGCGCTGGT
GTGGGCAGAACAGGGACGTTCGTTGCCCTGGACCGGATCCTCCAGCAGTTGGACTCTAAGGACTCCGTGGACAT
TTATGGGGCAGTGCATGACCTAAGACTCCACAGGGTTCACATGGTCCAGACCGAGTGTCAATATGTGTATCTGC
ATCAGTGTGTAAGAGACGTCCTCAGAGCAAAG

>PTPRBgallus

AGTCATTTTGAAGCACACTTCACCAAACTCCAAGCAGACTCCAACTACCTCCTGTCCAAGGAATATGAGGACTT
AAAAGATGTGGGTCGAAACCAGACATGTGACATAGCACTTCTGCCAGAGAACAGAGGGAAAAATCGATACAATA
ATATATTGCCTTATGATACATCAAGAGTGAAGCTTTCCAATGTGGATGATGACCCTTGCTCAGACTACATTAAT
GCAAGCTATATACCAGGCAATAATTTCCGCAGGGAATACATAGCAACTCAGGGCCCTTTGCCTGGCACTAAAGA
TGAGTTCTGGAAGATGGCATGGGAACAAAATGTGCACAATATTGTCATGGTAACCCAGTGTGTTGAGAAGGGCC
GGGTAAAGTGTGATCACTACTGGCCCCTTGACCAGGATTCCTTGTACTATGGAGACCTGATAGTTGAGATGCTG
TCTGAATCAGTGCTCCCAGAATGGACAATACGAGAGTTTAAAATCTGCAGTGAGGAGCAGCTTGACTCAACGAG
GCTCATTCGTCACTTCCACTACACTGTGTGGCCAGATCACGGAGTGCCAGAGACCACCCAGTCCCTGATCCAAT
TTGTCAGAACTGTAAGGGATTATATCAACAGGACCCCAGACACGGGACCAACTGTTGTGCACTGCAGTGCTGGT
GTTGGCAGAACTGGCACATTCATTGCACTGGACCGAATTCTGCAGCAACTGGATTCAAAGGACACTGTTGACAT
TTATGCAGCAGTGCACGATCTAAGGCTTCACCGCGTTCACATGGTTCAAACAGAGTGTCAATACGTGTATCTAC
ATCAGTGTGTGAGAGATGTGTTAAGAGCCAGA

>PTPRB.frog

GCCCAGTTTGAAGAACATTTCAGCAAGCTTCAGACCGATTCCAATTACTTGCTTTCTCGGGAGTATGAGAACCT
TAAAGATTTTGGTCGAGACCAGTCTTCTGATACTGCCCTTCTCCCAGAAAACAGAGGAAAGAATAGATATAGCA
ACATATTGCCCTATGATTCCACAAGAGTGAAACTTGCCAACGTTGACGATGACCCCTGTTCAGACTATATTAAC
GCCAGCTATATGCCAGGCATCAACTTTCGGAGAGAATATATTGCTACACAAGGGCCTTTACCAGCAACCAAAGA
TGATTTCTGGAAAATGGTGTGGGAACAAAATGTTCACATCATTGTGATGGTGACACAGTGTACAGAGAGAGGAC
GGGCAAAATGCGATCATTATTGGCCCATGGACCAAGACTCATACTATTACGGAGACCTGATAGTACAAATGCTG
TCAGAATCTGTGCTTCCTGAATGGACAATAAGGGAATTTAAAATCTGCAGTGAAGATCAAATTGATGCACCACG
ACTAGTCCGTCATTTCCACTATACAGTGTGGCCAGATCATGGGGTGCCAGAAACCACCCAATCACTGATCCAGT
TTGTAAGAACAGTCAGAGACTATATTAACAGAACTCCTGGAAGTGGCCCTACTGTGGTGCACTGCAGTGCTGGT
GTTGGCAGAACTGGCACATTCATTGTACTGGACCGAATGCTTCAACAAGTTGATACAGTGGATTCAGTGGATAT
ATTTGGAGCTGTTAGGGACCTCCGAATTCACCGGATGTACATGGTGCAGACCGAGTGTCAGTACGTCTATTTGT
ATCAATGTGTAAGAGACGTCTTAAGAGCCAGG

>PTPRB.fish

GCCAATTTCGAGTCCCACCTTGCTAAACTTCAATCCGATTCCAGTTTCCTTCTGTCAGAAGAGTTTGAGGGCCT
AAAGGACGTGGGACGGATCCAAACTCAAAATGCTGCACGTCTGCTTGGGAACCGCAACAAAAACCGATACAACA
ATATACTTCCCTACGACTCCACGCGGGTGAGACTGTCCTGTCTGGAGGATGACCCCTGCTCAGACTACATCAAC
GCTAACTATATACCTGGAAACAATTTCAGATGGGAGTATATCGCCACACAAGGGCCTTTGCCAGGTACCAAGGA
CGATTTTTGGCGAATGGTGTGGGAACAAAATGTGCACAGCCTAGTAATGGTGACGCAGTGTGTGGAAAGAGGCA
TGGTAAAGTGTGACCACTACTGGCCGACGAACAGCGAGCCTTTGTGCTATGGAGACATTGTGGTGCAGCTGCTT
TCTGAGAAGGTCTTTCCAGAGTGGACCATTCGGGACTTCAAAATATCATGCGAGAGTCAGCTGAGATACCCCAG
GATGGTGCGTCAGTTCCATTACACCATCTGGCCAGATCACGGCGTCCCGGACACCACTCAGTCTCTTATCCAGT
TTGTGCGAACAGTCCGAGATTTCATCAACAGGACAAACAGTCCTGGAATCTCCGTAGTACACTGCAGTGCTGGT
GTGGGTCGAACTGGGACTTTCATAGTGCTGGATCGTGCTTTACAGCAACTAGACAGGAACTGCACAGTGGACAT
TTACGGCTGTGTGTTTGACCTGCGCCTGCATCGCTCATACATGGTACAAACCGAGTGTCAGTATGCTTATATCC
ATCAGTGTGTGCGGGATGTTTTGAGGGCTCGC

>PTPRJ.human

GAGAATTTTGAGGCCTACTTCAAGAAGCAGCAAGCTGACTCCAACTGTGGGTTCGCAGAGGAATACGAAGATCT
GAAGCTTGTTGGAATTAGTCAACCTAAATATGCAGCAGAACTGGCTGAGAATAGAGGAAAGAATCGCTATAATA
ATGTTCTGCCCTATGATATTTCCCGTGTCAAACTTTCGGTCCAGACCCATTCAACGGATGACTACATCAATGCC
AACTACATGCCTGGCTACCACTCCAAGAAAGATTTTATTGCCACACAAGGACCTTTACCGAACACTTTGAAAGA
TTTTTGGCGTATGGTTTGGGAGAAAAATGTATATGCCATCATTATGTTGACTAAATGTGTTGAACAGGGAAGAA
CCAAATGTGAGGAGTATTGGCCCTCCAAGCAGGCTCAGGACTATGGAGACATAACTGTGGCAATGACATCAGAA
ATTGTTCTTCCGGAATGGACCATCAGAGATTTCACAGTGAAAAATATCCAGACAAGTGAGAGTCACCCTCTGAG
ACAGTTCCATTTCACCTCCTGGCCAGACCACGGTGTTCCCGACACCACTGACCTGCTCATCAACTTCCGGTACC
TCGTTCGTGACTACATGAAGCAGAGTCCTCCCGAATCGCCGATTCTGGTGCATTGCAGTGCTGGGGTCGGAAGG
ACGGGCACTTTCATTGCCATTGATCGTCTCATCTACCAGATAGAGAATGAGAACACCGTGGATGTGTATGGGAT
TGTGTATGACCTTCGAATGCATAGGCCTTTAATGGTGCAGACAGAGGACCAGTATGTTTTCCTCAATCAGTGTG
TTTTGGATATTGTCAGATCCCAG

>PTPRJmouse

GAGAATTTTGAGGCCTACTTTAAGAAGCAGCAAGCTGACTCTAACTGTGGGTTTGCAGAGGAATATGAGGACCT
GAAGCTGATTGGGATAAGTTTACCTAAATACACAGCTGAGATAGCCGAGAACAGAGGGAAGAACCGCTACAACA
ATGTTCTGCCCTATGATATTTCTCGAGTCAAACTTTCAGTCCAGACCCATTCGACAGATGACTACATCAATGCC
AACTATATGCCTGGCTACCATTCCAAGAAAGATTTCATTGCCACACAAGGACCTTTACCCAACACTTTGAAAGA
TTTCTGGCGTATGGTTTGGGAGAAAAACGTATATGCCATTGTTATGTTGACCAAATGCGTGGAGCAGGGAAGGA

CCAAATGTGAGGAGTACTGGCCTTCCAAGCAGGCTCAGGACTACGGGGACATAACTGTGGCGATGACATCAGAA
GTCGTTCTTCCAGAATGGACCATCAGAGATTTTGTGGTGAAAAATATGCAGAATAGCGAGAGCCATCCTCTGCG
GCAGTTCCATTTCACCTCCTGGCCTGACCACGGTGTTCCTGACACCACTGACCTGCTCATCAACTTTCGGTACC
TGGTCCGGGATTACATGAAGCAGATACCCCCCGAGTCACCAATTCTGGTGCATTGCAGTGCTGGGGTTGGAAGG
ACGGGCACTTTCATCGCCATCGATCGCCTGATCTATCAGATAGAGAATGAGAACACCGTGGACGTGTATGGGAT
TGTCTATGACCTTCGGATGCACAGGCCTCTGATGGTGCAGACAGAGGACCAGTATGTTTTCCTCAATCAGTGTG
TTTTGGATATTATCAGAGCCCAG

>PTPRJ.chiken

GAGAACTTTGAGTCCTACTTTAAGAAGCAGCAAGCAGACTCCAACTGTGGTTTTGCTGAAGAGTATGAGGAACT
CAAGTCTGCTGGTGTTCATCAGCCCAAATTTGCTGCTGAAATCGCTGAGAACAGGGGGAAAAATAGATACAACA
ATGTCTTACCATATGATATTTCTCGTGTTAAACTTTCAAATCCAAGCTGTACAACTGATGATTATATTAATGCA
AACTATATGCCTGGATACAGCTCAAAGAAGGCATTTATTGCTGCACAAGGCCCTTTACCCAATACTATAGAAGA
CTTCTGGCGCATGATTTGGGAAAAGAATATCTACTCTATTGTCATGTTGACAAAATGTGTTGAACAGGCCCGGA
CGAAATGTGAGCAATATTGGCCAGACAAACAATCCAAGAGTTATGGTGACATTATTGTGACAATGGTCTCAGAA
GTTGTTCTTCCAGAATGGACGATAAGGGACTTCAATGTAGAGAACGCTGACACAATGGAGAGCCACACAGTGCG
TCAGTTCCATTTCACATCCTGGCCAGATCATGGAGTGCCAGAGACAACAGACTTGCTAATCAACTTCAGACACC
TTGTTCATGAATACAGCAGTCAGAATCCTATTGACTCTCCTATTCTAGTGCACTGCAGTGCTGGTGTCGGGAGG
ACAGGTACGTTTATTGCCATTGACCGCCTGATTCAGCAGATTGAAATGGAGAATACTGTGGATGTGTATGGAGT
GGTGTACGATCTTCGAATGCATCGACCTTTAATGGTGCAAACTGAGGACCAGTATGTCTTCCTAAACCAGTGTG
TCATGGATATTATTAGATCACAG

>PTPRJ.fish

GAAGATTATGAGGCTTACTATAAGAGACAAAGAGCTGATTCTTTCTGTGGATTTGCTGAAGAATTTGAGGATCT
AAGGCCGGTTGGGATAAATCAATCAAAGACTGTTGCTGTGTTTCCTGAAAACAAAGCCAAGAATCGATATAATA
ATGTGCTGCCATACGATTCTTCTCGTGTCAAGCTCTCTGTTCTCAGCAGCCCATTTGATGACTACATCAATGCC
AACTACATGCCGGGTTACATTACAAAGAAGGAGTTTATCGCAGCACAAGGTCCATTGCCCGGCACTGTTAATGA
CTTCTGGAGGCTGATCTGGGAGAAGAATGTGCACACTATTGTCATGCTCACCAAATGCAATGAACAAGGCCGGG
TAAAATGTGAGGAATACTGGCCAGCAGAGATGAAGACTTTTAGCAATCTGACAGTGACAACAATTTCTGATATC
CCATTGGAGGATTGGACTTTACGTGACTTTGAAGTGAAAAATATGAAAACAGCAGAAATTCGATCGGTTCGTCA
CTTCCATTTCACTGCATGGCCAGACCATGGAGTTCCTGAGACCACTGAGCTTCTCATCAACTTCAGACATCTGG
TCCGAGAGCACATGGATGAGTACTCAAGACATTCCCCCACACTTGTGCACTGCAGTGCGGGTGTGGGCCGGACA
GGTACCTTCATTGCCATTGATAGGTTAATCTTCCAGATCGAGCGGGATGGTGTAGTGGATGTATATGGGATCAT
CCATGACTTACGCATGCACAGACCCCTCATGGTGCAAACAGAGGATCAATATGTCTTTCTTAACCAGTGTGCCA
TGGACATCATCAAGTCTCGA

>PTPRO.human

GATGACTTTGATGCCTATATTAAGGATATGGCCAAAGACTCTGACTATAAATTTTCTCTTCAGTTTGAGGAGTT
GAAATTGATTGGACTGGATATCCCACACTTTGCTGCAGATCTTCCACTGAATCGATGTAAAAACCGTTACACAA
ACATCCTACCATATGACTTCAGCCGTGTGAGATTAGTCTCCATGAATGAAGAGGAAGGTGCAGACTACATCAAT
GCCAACTATATTCCTGGATACAACTCACCCCAGGAGTATATTGCCACCCAGGGGCCACTGCCTGAAACCAGAAA
TGACTTCTGGAAGATGGTCCTGCAACAAAAGTCTCAGATTATTGTCATGCTCACTCAGTGTAATGAGAAAAGGA
GGGTGAAATGTGACCATTACTGGCCATTCACGGAAGAACCTATAGCCTATGGAGACATCACTGTGGAGATGATT
TCAGAGGAAGAGCAGGACGACTGGGCCTGTAGACACTTCCGGATCAACTATGCTGACGAGATGCAGGATGTGAT
GCATTTTAACTACACTGCATGGCCTGATCATGGTGTGCCCACAGCAAATGCTGCAGAAAGTATCCTGCAGTTTG
TACACATGGTCCGACAGCAAGCTACCAAGAGCAAAGGTCCCATGATCATTCACTGCAGTGCTGGCGTGGGACGG
ACAGGAACATTCATTGCCCTGGACAGGCTCTTGCAGCACATTCGGGATCATGAGTTTGTTGACATCTTAGGGCT
GGTGTCAGAAATGAGGTCATACCGGATGTCTATGGTACAGACAGAGGAGCAGTACATTTTTATCCATCAGTGTG
TGCAACTGATGTGGATGAAGAAG

>PTPRO.mouse

GATGACTTCGATTCTTACATCAAGGATATGGCCAAGGACTCGGACTATAAATTCTCTCTTCAGTTTGAGGAGTT
GAAGTTGATTGGACTGGATATTCCGCACTTTGCTGCAGATCTACCGCTGAACCGATGTAAAAACCGCTACACAA
ACATCCTGCCGTATGACTTTAGCCGGGTGAGGCTAGTCTCCATGAACGAAGAGGAAGGAGCAGACTACATTAAT
GCCAACTATATTCCTGGATACAACTCACCCCAGGAGTACATTGCCACCCAGGGTCCCCTGCCAGAAACCAGAAA
TGACTTCTGGAAGATGGTCCTACAACAGAAGTCCCACATCATCGTCATGCTCACTCAATGCAATGAGAAAAGGA
GGGTAAAATGTGACCACTACTGGCCATTCACAGAAGAACCCATTGCTTATGGGGACATCACCGTGGAGATGGTC
TCTGAGGAAGAGGAGGAGGACTGGGCCAGTAGACACTTCCGGATCAACTATGCGGACGAAGCGCAGGACGTGAT
GCATTTTAACTACACAGCCTGGCCCGATCACGGCGTGCCTCCAGCAAACGCCGCCGAGAGCATCCTGCAGTTTG
TGTTCACAGTGCGACAGCAAGCCGCCAAGAGCAAAGGGCCCATGATCATCCACTGCAGTGCGGGTGTGGGACGG
ACAGGAACCTTCATTGCCCTGGACAGGCTCCTGCAACACATTCGAGATCATGAATTTGTGGACATCTTAGGGCT
GGTATCAGAGATGCGCTCATACCGAATGTCAATGGTACAGACAGAGGAGCAGTACATTTTTATCCATCAGTGTG
TGCAGCTGATGTGGCTGAGGAAG

>PTPRO.chiken

GATGATTTTGATGGCTACATCAAGGATATGGCCAAAGATTCTGATTATAAATTTTCTCTGCAATTTGAGGAGCT
AAAACTGATTGGGCTTGATATACCTCACTTTGCCGCTGATCTTCCCATGAATCGCTGCAAAAAATCGCTACACAA
ATATCCTGCCATATGACTTTAGTCGCGTCCGGTTAGTCTCAATGAATGAAGAAGAGGGATCTGACTACATTAAT
GCCAACTATATTCCTGGTTATAATTCACCCCAGGAATACATAGCAACCCAAGGACCACTGCCAGAAACTAGGAA
TGATTTTTGGAAGATGGTTCTTCAGCAAAAATCTCAAATTATTGTTATGCTCACTCAGTGCAATGAGAAAAGGC
GGGTGAAATGTGATCATTACTGGCCTTTTACAGAAGATCCTATTGCATACGGGGACATTACTGTCGAGATGCTG
TCTGAGGAAGAACACACAGATTGGGTTTATAGGAATTTCCGAATTAGCTATGCTGATGAGGTGCAAGACGTGAT
GCATTTTAACTACACTGCCTGGCCAGACCATGGTGTCCCCACAGCCAATGCCGCCGAAAGCATCCTGCAGTTCG
TGCAGATGGTCAGGCAGAAGTCAGTAAAGAGTAAAGGCCCCATGATCATACACTGCAGTGCTGGAGTGGGACGA
ACAGGAACATTCATAGCCCTGGACTGGCTCTTACAGCACATCCGTGACCATGAGTTTGTAGATATATTGGGCCT
GGTGTCTGACATGAGGTCCTACAGAATGTCCATGGTGCAGACAGAGGAACAATACATTTTTATTCACCAGTGTG
TGCAATTGATGTGGCAAAAGAAG

>PTPRO.frog

GATGACTTTGATTTATACATAAAGGAAATGTCCAAAGACTCTGATTACAAATTCTCCCTCCAGTTTGAAGAACT
AAAGATGGTCGGACTGGATATTCCGCACTTTGCCGCCGACCTTCCATCAAACAGAGGCAAAAACCGCTACACGA
ATATCCTGCCATATGATTGCAGCCGCGTGAAACTGATTTCTCTAGAAGCCGATGAAGGAGCCGATTATATCAAC
GCCAATTACATTCCGGGCTACAGTGCCGCCCAGGAATATATCGCCACTCAGGGGCCCCTGCCGGAGACCAGGAA
CGACTTCTGGAAAATGATCCTTCAGCAGAAGTGCCAAGTTATTGTCATGCTGACCCAGTGCAACGAGAAGCGGC
GGATTAAATGTGACCACTACTGGCCATTCACTCCGGAACCGGTAAACTATGGCGATATCACCGTGGAGATGGCG
TCAGAGGAGGAGCAATCGGACTGGGCGCAGCGAGTATTTAGGGTCTCGTATGCGGATGAGACCCAGTGCGTCAC
ACACTTTAACTTCACGGCGTGGCCTGACCATGGGGTTCCGACAGTCAATGCGGCCGAGAGTGTCCTGCAGTTTG
TCCAGGTCGTCAGGCAGAAGGCGCTGAAGTCTAAGGGACCCGTAACGGTGCATTGCAGCGCCGGCGTGGGTCGG
ACCGGAACCTTCATAGCGCTGGACTGGCTGATGCAGCACATCCGGGACCATGAATTTGTCGATATCTTGGGGCT
CGTGAGTGAGTTGCGCTCTCACAGGATGTCTATGGTGCAGACAGAGGAGCAGTTTGTGTTCATCCATCAGTGCG
CGCAGTTGATGTGGAAGAAGAAAACCCCGTGCAGCTT

>PTPRO.fish

AGTGATTTCGAGGCCTACCTCAAAGACATGGGCAAAGACTCAGCCTACAAGTTCTCCCTGCAGTTCGAGGAGCT
GAAGAGTGTAGGTCTGGATTTGTCCCATGAAGCCGCTGATCTGCCCATCAACAGACCCAAGAACCGCTACACCA
ACATCCTGCCCTATGACTTCAGTCGAGTGAAGCTGATCTCTCTGCACAATGATGAAGGCTCTGATTACATCAAT
GCTAACTATATTCCCGGCTATAACTCTCCACGCGAGTACATCGCCACGCAGGGCCCACTGCCCGACACCAGGAA
CGACTTCTGGAAGATGGTGCTGCAGCAGAAAGTGCACATCATCGTGATGCTAACACAGTGCAACGAGCGCCGCA
GGGTGAAGTGTGACCACTACTGGCCGTTCAGTGATGAGCCGGTGGCGTATGGAGAGATCAGTGTGGAGATGCTG
GCGGAGACAGACTCGCCCGAGTGGACCATCAGGAGCTTCAGACTCGCATACGCTGATGAGACTCAGGACGTCCT
GCACTTTAACTACACCTCGTGGCCGGATCACGGAGTGCCGACGGTGAACGCCATCGAGAGCATCCTACAGTTCG
TGCAGATCGTCCGGCAGCAGGTCAACAGGAGCAAAGGACCCATCGTAGTGCACTGCAGTGCTGGCGTGGGCCGC
ACCGGCACCTTCATCTCTCTGGACCGGCTGATGCAGCACATTCAGGAGCACGAGTATGTGGACGTGCTGGGGCT
GGTGTCCGACATGCGCTCGCACCGACTGTCCATGGTGCAGACTGAGGAGCAGTACGTGTTCATCCATCAGTGTG
TTCTGCTGATGTGGAAGAAGAAG

>PTPRH.human

GAAGACTTCGCTGACCACGTCAGGAAGAATGAGAGGGACAGCAACTGTGGTTTTGCAGACAAGTACCAGCAACT
CTCCCTGGTGGGCCACAGCCAGTCTCAGATGGTGGCTTCGGCTTCAGAGAACAACGCCAAGAACCGCTACAGAA
ATGTGCTGCCCTATGACTGGTCCCGGGTGCCCCTGAAGCCCATCCATGAGGAGCCAGGCTCTGACTACATCAAT
GCCAGCTTCATGCCCGGTCTCTGGAGCCCCCAGGAGTTCATTGCAACCCAGGGTCCCCTGCCACAGACAGTGGG
TGACTTCTGGCGCCTGGTGTGGGAACAGCAGAGCCACACCCTGGTCATGCTGACCAACTGCATGGAGGCCGGCC
GGGTGAAGTGTGAGCATTACTGGCCTCTGGACTCGCAGCCCTGCACCCATGGGCACCTGCGGGTAACCCTGGTA
GGTGAGGAAGTGATGGAGAACTGGACGGTGCGGGAACTGCTGCTCCTCCAGGTGGAGGAGCAGAAGACACTGTC
TGTGCGCCAATTCCACTACCAGGCCTGGCCGGATCACGGCGTTCCCTCCTCCCCAGACACCTTGCTGGCTTTCT
GGAGGATGCTTCGGCAGTGGCTGGATCAGACCATGGAGGGAGGCCCACCCATTGTGCACTGCAGTGCTGGCGTG
GGTCGCACAGGAACCCTCATTGCCCTGGACGTCCTGCTCCGGCAGCTGCAGTCCGAGGGTCTCCTTGGGCCCTT
CAGCTTTGTAAGGAAGATGAGAGAGAGTCGGCCGTTGATGGTGCAGACTGAGGCTCAGTACGTATTCCTGCATC
AGTGCATCCTGCGGTTCCTCCAACAGTCA

>PTPRH.mouse

AAAGACTTTGCTGACCATGTCAGGGAGAATGAGAAGGACAGCAACTGTGGATTTGCTGAGGAGTATCAGCAACT
GGCTCTGGAGGGCCAAGGCCAGTCTCAAATAACGGCATCGGCTCTAGAGAACAGATCCAAGAACCGCTACAGGA
ATGTACTGCCCTATGACTGGTCCCGGGTTCCCCTGCAGCCCCTCCAAGAGGAACCAGGCTCTGACTATATCAAC

GCCAGCTTTATGCCTGGTCTCTGGAGCCCCAAGGAGTTCATTGCAACCCAGGGTCCCCTGCCTAACACTGTGGG
TGATTTTTGGAGAATGGTCTGGGAACAGCAGAGCCACACCCTGGTTATGCTGACCAACTGCATGGAGTCTGGAC
GGGTGAAGTGTGAGCACTACTGGCCCTTGGATGCTCAGCCTTGCATTCATGGGCAGCTGCAGGTAATGTTGATT
AGTGAGGAGGCGTCAGAAAACTGGACAGTGAGGCATCTTCAACTCTTCCATATGAAAGAACAGCAGACTCTCTC
GTTGCGCCAATTCCACTACCTGGCCTGGCCAGACCATGGAGTTCCTTACTCCCCAGACCCCCTGCTGGCTTTCC
GAAAGATGCTTCGGCAATGGATGGATCAGACCACGGATGGAGGTCCACCCATTGTGCATTGCAGTGCTGGTGTG
GGCCGGACGGGCACCCTAATTGCACTGGATGTCCTGCTGCGGCAGCTGGAGTGTGAGGGGCTGGTGGGTCCCTT
CAGCTTTGTGAAGAAGATGAGAGAGAGCCGGCCACTGATGGTGCAGACAGAGGCCCAGTATGTGTTCCTCCACC
AGTGCATCTTGAAATCCCTCCAGAAGCCA

>PTPRH.frog

TCAGCGTTTCCAGATTATTACCAGAGACAGCACGCGGATAGTGACTTTGGATTTGCAGAGGAGTATCAGCAACT
CAGCAATGTAGGCATTAACCAGTCTAAACTGGCAGCAGAACTCTCAGAAAACAGATCCAAGAACAGATTCACCA
ATGTCTTGCCTTATGACCATTCCAGAGTCAGACTCAACCGTATTGATGCGGATGAGACCTCTGATTATATCAAT
GCCAACTACATGCCTGGCTACAACTCTTCTAAGGAATTCATAGCTTCCCAAGGCCCACTGCCCAACACAAGTGC
AGACTTCTGGAGGATGATTTGGGAGAATCAAGTCAGTACCATTGTAATGCTGACCAACTGCATGGAGAATGGCA
GGGTGAAATGTGAACATTATTGGCCCTTGGATTACACCCCATGCACATACGGAGACATCACCGTGACTGTAACC
TCCGAAATGATCCTTCCTGATTGGACAGTACGGGATTTTACCCTTAAACATGCCAAGCAGCAAGGCAATAAGCA
TGCTCGTCATTTTCACTTTACGGTCTGGCCCGACCACGGTGTTCCAGAAAACACAACAACCATTGTCGAGTTCC
GGAACCTTGTTCGAGAATACATGGACCTGAAGAGGAGCAGCGGACCCACGGTTGTTCACTGCAGTGCTGGTGTT
GGGAGGACAGGGACACTGATTGCCCTGGATTACCTGATCCAGAAGATGGAGAAGGAGCAGCGTATTGGAATATA
TAGCTTTGTTCAGAAGATGAGGCAGAACCGTCCCTTAATGGTGCAGACAGAGAGTCAGTACGTATTTCTGAATA
AGTGTATGCTGGATCTAATCCAGAATCCTCCC

>PTPRH.fish

GATAAATTTCCTGAACATTTCCGAAACATGAGTCGTGATGATAACAGAGCTTTCAGTGCTGAATATGATGACTT
GAGTTCAGTGGGTGTAGAACAGTCAAAGGTTGCGGCTCTTCTCCCTGAAAACAAGGATAAAAATCGATTCTCCA
ATGTTTTACCATATGACACCTCTAGAGTGCATCTTACCATAAATAAAGCGGGTGATTCTGATTACATCAATGCT
AACTACATGCCTGGATATGGAAATGCAAGCAGAGAGTACATAGCTGCTCAAGGTCCACTGCCATCCACTGTCAA
TGACTTCTGGAGAATGATCTGGGAGAAAAAGTCATCGACTATTGTCATGGTTACCAACTGTACTGAGGGTGGAA
GGGTCAAGTGTGAACAGTACTGGCCACTGGACTACACACCGTGTCTTTATGAAAACCTGCTTGTCACAGTGAAA
TCAGAGAACAAATCCCAAAGTTGGACTTTGCGAGAATTTAATGTCAAAAATAAAATGACCTCTGAGACGCGAAC
AGTGCGTCACTTCCACTTCACAGCATGGCCGGATCATGGTGTTCCCCGGGGCACAGAAGAGCTGATCCAATTCA
GAGACCTTATCCGTCAGCACATAGAGAGCCACTTCTCTACAGGACCAACAGTAGTGCACTGCAGTGCTGGAGTG
GGCCGGACAGGGACGCTGATCGCGCTGGACGTCCTGCTGCAGCAGCTGAACAGAGAGAAGGCAGTGGGTGTAGC
AGCATTCGTCCAGGAAATGAGACTCAACCGGCCACTTATGGTGCAAACGGAGTCTCAGTATGTGTTCCTGCACC
AGTGCATCCTGGACTCTTTGCAAACCAAG

>Ciona.1

GCTGAGTTTCTTGACTTACTTAAAGTCATGAAAGCAGATTCTGATTTTAAATTCTCAGAAGAGTATGAGGAATT
CAAGACCGTTGGGCGAGACCAGGCAACAGTTGCTGCTCTGTTGCCCGAAAATCGTGGCAAGAACCGTTACACCA
ACATTCTGCCATATGATGCAACTCGTGTAAAGTTATCTGCAATTGACGACGAACAAGGAACGGATTACATCAAT
GCTAACTTTATCCCGGGCAACAACAATCGTCAGCGCGAGTACATCGCCACTCAAGGTCCTCTTCCAGGAACAAA
GGAAGATTTTTGGAGGATGGTTTGGGAGCAAAACTCGAGGAACATTGTGATGGTGACACAGACTGTGGAGAGAG
GCAAGATAAAGTGCGACCACTACTGGCCATTCGACAACGAACCAATCACAGTGGCTGATTACACGCTACAGATG
ACATCAGAATCCATTCTTCCTGAGTGGACGATCCGTGAATTCAAGATCACACACGGGTCTGACACTCGTAGGAT
CCGACAGTTCCATTACACAGTATGGCCCGATCATGGTGTGCCAGACACCGCTGAAACACTCGTTAAGTTCATTC
GTTACGTTCGTCGAACTATTGACAGGGAAGCGAAACATAGCGGACCAACCGTTGTTCACTGCAGTGCTGGAGTT
GGTCGTACAGGAACATTTATCGCAATGGATCGACTTCTTCAACATCTTCCCGACAATAATTACGTCGATATCTT
TGGGATCGTTCATCAAATGCGAATTCATCGTGTGTTTATGGTGCAGACAGAGAGCCAGTATATCCTCATCCACC
AGATGGTTCAAGATATTCTCAACCGAGTT

>AcornWorm.1

GCTGAATATCATGGATATTTCCAAATTATGAGTGCAGACATGGAGTTCCGCTTCTCAGAAGAATATGATGAACT
GCGACCAGTTGGGAGAGAACAGTCTTGGGATGCAGCTGAGTTGCCAGAAAATAGAGCTAAAAATAGATACACCA
ACATTCTACCATATGATCGTACAAGAGTTAAACTCTCACAGGTGGAAGATGATGAAGCCACTGATTATCTCAAT
GCTAACTGGATGCCAGGTTTCAATTCTCCCAGGGAATTCATCGCTGTACAAGGAACACTGCCAGGTACCAAGGA
TGATTTTTGGAGAATGATTTGGGAATATAATGTGAGCACCATTGTCATGGTAACACAATGTCAGGAAAGAGGAC
GGGTGAAATGTGAGCGTTATTGGCCAACTGATGATAACCCAGTTTACTATGGTGATGTGTTGGTGACAGTCACT
CATGAAAATGAACTGACTGATTGGGTTATCAGGGAATTCACAGTGGAAAACGGCAAGTCACTGAGACGAATACG
TCACTTTAATTTCACTGCGTGGCCTGATCATGGTGTACCTGAAGAAACAGGTTCTCTGATCAAGTTTGTACGAT
CTGTCAGGGCGCAGATTCAGAATGATGGTACACCAACTGTTGTACATTGCAGTGCTGGTGTTGGTAGGACTGGT
ACTTTCATTGCACTAGATAGACTTATACAGCATATAAAGGAGTATGACTACGTGGATATATTTGGTATCGCATG

TGAAATGAGAATGCATAGAGTCTATATGATCCAAACTGAGTCCCAGTACATATTCATTCATCTTTGTGTGGATG
ACCTATTAAAAGAGCGA

>Nematode.3

GCGGATTTCGCTGAACATGTTCGTATGATGTCTGCCGATTCCGATTTCCGTTTTTCGGAAGAATATGAGATTCT
GCGAAACGTTGGCTGTGGGCAATCATACACGGCTGCCGAATTGCCCGTAAATAAACCAAAGAACAGATTCACAA
ATATTCTCCCATATGACCATTCCCGTGTTCGATTAATGCAAGCGGGTGATCAAGAAGGCGCCGACTATATCAAT
GCCAACTTCATGCCGGGTTACTCTTCACGACGAGAATTCATCGCTGCCCAAGGACCTTTACCAACAACCAGAGA
CGCCTTTTGGCAGATGGCATGGGAACAATACTGCCCGGCAATCATTGCGTTAACAAAGTGCGTGGAAAAAGGTC
GAGATAAATGTCATCAGTATTGGCCAGACAGTGAACACTCAAGCGTCGTCTATTCCGATATCGAGGTAACCTTG
TTGAATGAATCGTCCTATGACGATTTCACGATCCGTGAGCTTCGTCTAAAGAAGTTGCACGAATCGACGGCACC
TCGGATCATCTGGCACTTCCACTATATGGCTTGGCCAGATTTCGGCGTACCAGATCATCCACAGGGAATTATTC
GATTCGCTCTGATGTTCCGCTCGAGACTTCCGCACTCTGCCCTGAACAAACCGACCATTGTCCACTGCAGTGCT
GGAGTCGGAAGGTCTGGTACATTCATAGCGATAGATCGCCTTCTTCAAACGATACAAGCTGATCGACCTATAGA
TGTCTTTGGAATTGTGCACGAAATGCGATATGAGCGATGTCATATGGTCCAAAATGAACAACAATACATCTTCA
TCCATCATTGCATTCTTCATGCCATCGAGTCTATC

>Nematode.1

GAAGACTTCGCCGAGCACGTGCGACTGATGTCAGCAGATTCTGATTTCCGATTCTCTGAGGAATACGATATGAT
GCGAAACGTGGGTGTTGGACAATCAGTCGCCGCTTCAGAATTGACTGTAAATCGACCGAAGAATCGTTTCACCA
ACATTCCTGCCTATGATCATTCCCGTGTGAAGCTGTCAAATCCGAATAATATCGAAGGAGGCGACTACATCAAT
GCCAATTATGTTCCCGGATTCTCGTCTCGTCGAGAGTTCATTGCCGCCCAAGGTCCTTTACCATCGACACGTGA
TCACTTCTGGCAGATGACGTGGGAGCAGCAGTGTCCAGCGATCATTGCATTGACGAAGTGTGTGGAAAAGGGAC
GAGATAAGTGTCATCAGTACTGGCCGGATCATGAGAATGTGCCTGTTTTGTATGGAGATATTGAGGTAACGATA
ATGGCTGAAAAAGAGTACGAAGAGTTTGTGATCCGAGATATTCGATTGGAAAAAGTGGGTCCAGAAGGTCGAGC
GACCCGATACGTTCGTCATTGGCATTACATGGCGTGGCCAGATTTTGGAGCACCAGCCCATCCGAACGGAATCA
TTCAATTCTCTCGAATGTTCCGACATCAGCTACCACATTCACCTCACAACGAACCAACGATAGTACATTGCAGT
GCTGGAGTTGGTCGTTCTGGAACCTTCATCAGCATCGATCGTCTCCTACAAACTAGTTCACTTGGAGAGCCAAT
CGATGTATTTGGAACAGTTTGTGAGATGCGATATGAGCGGTGTCAGATGGTGCAGAATGAACAACAATACATAT
TTATTCATTATTGTGTTCTTCAAGTCCTTCAAGGATCT

>Ciona.2

ACCGACTTTTCGTTGTTGGTGGAAAAATTATCACAAAACAAAAATAAAGGTTTTAGTTCAGAATTCGATGACAT
TAGGGGAATACCCTATGCTGGTACTACATCTGTAGCAGAAAAAGCTTGTAATAAAACGAAAAACAGAACGGCTA
AGCTAGTACCTTTTGATCATTGTAGAGTCAAGATAGAAGGAATACCTGCTGTGGAAGGTTCAAATTACATCAAT
GCTTCCTATGTACCGGGTCCCCATAGTCCCGAACAGTACGTGGCCACCCAAACACCTCTGGATCACACAAAGAA
AGATTTCTGGAGGTTAATTTGGGAGACGGGTTCAAATACCATTGTGATGTTATGCAACGTCATGGAGGGAGGAA
AGAAGAGATGCAGTGAGTATTGGCCGAAAAAACAAACTGAATATTTTGGAAACTTGGCAATTCAGATGACCAAG
CAGGATGTATGCAAGGATTGGATTGTTCGACACTTTTCTGTAACAATGCGGGACAAAGTTCGTCATGTGACGCA
GTTTCAATTCATGAATTGGTCAGTAGTGGAACAGGGAAACCCCCTTCCATTGGTGCGATTTATTAAAAGTTATC
GGACAACGCGTCACAATGAGTACAAACCAACCATTGTTATGTGCAGCAACGGTAGTGGTCGAAGCGGCGTTTTC
ATTGGTTTGGACAAAATCGTTGATACTATGACATCATACATCGACGTGTTTGGGACAGTTGCGTCCCTGAGGAA
GTATCGGCCGTTTATGGTTCAAACCCTGACTGAATACATCGTAATGCACCAATGCATCGTGAACTTCATTGATG
ATGTC
