# Supplementary material for: Phosphotyrosine phosphatase R3 receptors: Origin, evolution and structural diversification
Source: PLoS One. 2017 Mar 3;12(3):e0172887. doi: 10.1371/journal.pone.0172887 (PMC5336234; doi:10.1371/journal.pone.0172887)
Supplement: S2 File — Each individual matrix appears in increasing gap cost order from top to bottom as labelled. (PDF) [file pone.0172887.s006.pdf]

S2 File

```
#NEXUS
[written Sun Feb 09 09:13:10 EST 2014 by Mesquite  version 2.75 (build 564) at 
Robs-MacBook-Air.local/10.0.1.3]

BEGIN TAXA;
	TITLE Taxa;
	DIMENSIONS NTAX=31;
	TAXLABELS
		capsasporaPTPc1 Sponge Acornworm Seaurchin4 seaurchin3 
seaurchin1 CElegans Ciona Fly10D FlyDPTP4E PTPRB_Hs PTPRB_Mm PTPRB_Gg PTPRB_Xt 
PTPRB_Dr PTPRJ_Hs PTPRJ_Mm PTPRJ_Gg PTPRQ_Hs PTPRQ_Mm PTPRQ_Gg PTPRQ_Dr PTPRH_Hs 
PTPRH_Mm PTPRH_Xt PTPRH_Dr PTPRO_Hs PTPRO_Mm PTPRO_Gg PTPRO_Xt PTPRO_Dr 
	;

END;


BEGIN CHARACTERS;
	TITLE  Character_Matrix;
	DIMENSIONS  NCHAR=1357;
	FORMAT DATATYPE = Protein GAP = - MISSING = ? interleave;
	MATRIX
	capsasporaPTPc1      
IVPAEEL--ADCVAQLHANSDLGFAEEYEAL-NVNEEYATNAALMPANKSKNRYANILPYDHSRVRLSVIPGVEGSDYVN
ANYIDGY-RKSHAYIACQGPLPDTFDDFWRMVWEQRSAVIVMVTNEEEKGRVKCHRYWPDD--SEV-VFGDVEVRMTRQE
ELSEFITRVFSLKNIRTGE----TRTVHHLQFTGWPDHGVP--HSTSSLIKFVKQAKAV---QPNDAGPMVIHCSAGVGR
TGTFIVTDMSLERTKV---ENNVDIFGCVSALRRN
	Sponge               
-VYLRDF--PSYVEDMLNDAGFKFSEEYEKVSAVGLDHSKDASLLPENRAKNRYTNILAYDHSRVKLESIDDEPGSDYIN
ANYIPGY-RMRRAYIATQGPLPSTFDDFWRMTWEQNSHVIVMLTQLVERGRTKCHRYWP-G-AQPE-VYGEINVDMLSET
EKSDWIVRKFKIT--KEKR----SRTITHYQFVSWPDHGVP--DEAGPALDFVREVHEV---ASSAFGPVIVHCSAGVGR
TGTFIALGTLLQHIKD---HDWVDLFGLASEMRQH
	Acornworm            
PVPLKGF--RDYVNELDEDDRKGFEEEYDDLRKLVVRQPTSVGQRMEHRSKNRYTNIVPYDNNRVILSG----SGNSYIN
ASYISGS-KGSRSYIATQGPLESTCGDFWKMIWQQRVTTVVMMTQCNELGKSKCHHYWPRDTQTQI-SHSDLTIRLTSET
RLPDWIIRDFSIE--SNGE----IRALRQFHFTSWPITGTP--YDADPLIRFIEAIRIQ---VLPNSGPILVHCSAGVGR
TGVFIALYHLLEYFYT---MIQVDIFGKVIKMRKQ
	Seaurchin4           
PTKKNMF--AQHVAKMSARNNAGFSDEYNNLPAMEKTRAVTAARQARNDDKNRYRNILPYDYTRVQLKGV---AGTDYIN
ASYIKDD-HGQKKYIATQGPLPNTIEHFWEMVWENQTSTIVMMTALVEGGKTKCEHYWPAG-EEPQ-LHGNVTVTLVGSN
QTDNFIERTLLLE--KEGT----ERTVTHYQYLAWTDHGVP--ESTAPLVGLLRQVKVTNQEDGAATGPIVVHCSAGVGR
TGTFIAADMLMDAIQRSTATDYIDVAGTIAKIREQ
	seaurchin3           
PVQMRKF--PDHVNRMSAGNNTGFVEEYNSLSNVGRDKSTDASRLAVNATKNRYRNILPFDETAVHLQDLEEGQTSDYIN
ANYIQGL-HSMKEYIASQGPVPDSVNDFWEMVFENKSTTIAMITGLVEGGKTKCEHYWPDD-DTPV-NYGSVTVTMTHTQ
EMEQWTVRSFLLE--KGMQ----QFETHQYAFKGWVDHDVP--NNARPMIEFIRTIDVA---HDANLGPITVHCSAGIGR
TGVFIALHKLIKQVETSKPNDYIDVCGTVARMREQ
	seaurchin1           
PIVLNKF--EGHYGRMKADSDFRFTEEYDEIRLVGKDQAIVSALEMVNRAKNRFTNILPYEHSRVKLAALADDSDTDYIN
ANYIPGY-NSPREFMACQGPLPGTVDDMWRMIWEKKTSIIVMLTQLVEKGKIKCHEYWPAD-YNPV-TYGSIQVSVQALQ
QYDHWVIREFSIS--QGDE----IRKLTQYHFMSWPDHGVP--DKTWTMLDFVRTVREA-IQKTASDRPIVAHCSAGVGR
TGTYIALDRLMQAMQE---NDYIDIFGIICEMRMQ
	CElegans             
PVRIEDF--ADHVRLMSADSDFRFSEEYDMMRNVGVGQSVAASELPINRPKNRFTNIPSYDHSRVKLSNPNNIEGGDYIN
ANYVPGF-SSRREFIAAQGPLPTTRDHFWQMTWEQQCPAIIALTKCVEKGRDKCHQYWPDHENVPV-LYGDIEVTIVAEK
EFDEFVIRDIRLE--KSGPDGRVTRFVRHWHYMAWPDFGAP--SHPNGIIQFSRMFRHH-LPHSPHNAPTIVHCSAGVGR
SGTFISIDRLLQSSSF---GDPIDVFGTVCEMRYE
	Ciona                
PVFRDEF--PNHVSSLSQNKNKGFSLEFDDIRGIPYAGTTAIAENSCNKTKNRTTKLVPFDHCRVKIEGIPAIQGSNYIN
ASYIPGL-DSPEQYIATQTPLDHTKKDFWRMLWETGSTNIVMLCGSVNAGKKRCDEFWPKK-QTE--YFGNLAVQMKEEI
RHDEWIIRQFIVT--MRDK----VRHVTQHHFIKWPQLEQA--ENSLPLVRFIKNYRLL---RDRSINPTIVMCSNGSGR
CGVFIGLLRILDNN-----GRDVDVFGTVAALRKY
	Fly10D               
PILIKNF--AEHYRLMSADSDFRFSEEFEELKHVGRDQPCTFADLPCNRPKNRFTNILPYDHSRFKLQPVDDDEGSDYIN
ANYVPGH-NSPREFIVTQGPLHSTRDDFWRMCWESNSRAIVMLTRCFEKGREKCDQYWPND-TVPV-FYGDIKVQILNDS
HYADWVMTEFMLC--RGSE----QRILRHFHFTTWPDFGVP--NPPQTLVRFVRAFRDR---IGAEQRPIVVHCSAGVGR
SGTFITLDRILQQINT---SDYVDIFGIVYAMRKE
	FlyDPTP4E
```

PVHVKDF--SEHYRIMSADSDFRFSEEFEELKHVGRDQACSFANLPCNRPKNRFTNILPYDHSRFKLQPVDDDDGSDYIN
ANYMPGH-NSPREFIVTQGPLHSTREEFWRMCWESNSRAIVMLTRCFEKGREKCDQYWPVD-RVAM-FYGDIKVQLIIDT
HYHDWSISEFMVS--RNCE----SRIMRHFHFTTWPDFGVP--EPPQSLVRFVRAFRDV---IGTDMRPIIVHCSAGVGR
SGTFIALDRILQHIHK---SDYVDIFGIVFAMRKE
PTPRB_Hs
PIKINQF--EGHFMKLQADSNYLLSKEYEELKDVGRNQSCDIALLPENRGKNRYNNILPYDATRVKLSNVDDDPCSDYIN
ASYIPGN-NFRREYIVTQGPLPGTKDDFWKMVWEQNVHNIVMVTQCVEKGRVKCDHYWPAD-QDSL-YYGDLILQMLSES
VLPEWTIREFKIC--GEEQ-LDAHRLIRHFHYTVWPDHGVP--ETTQSLIQFVRTVRDY-INRSPGAGPTVVHCSAGVGR
TGTFIALDRILQQLDS---KDSVDIYGAVHDLRLH
PTPRB_Mm
PIKINQF--EGHFMKLQADSNYLLSKEYEDLKDVGRSQSCDIALLPENRGKNRYNNILPYDASRVKLCNVDDDPCSDYIN
ASYIPGN-NFRREYIATQGPLPGTKDDFWKMAWEQNVHNIVMVTQCVEKGRVKCDHYWPAD-QDPL-YYGDLILQMVSES
VLPEWTIREFKIC--SEEQ-LDAHRLIRHFHYTVWPDHGVP--ETTQSLIQFVRTVRDY-INRSPGAGPTVVHCSAGVGR
TGTFVALDRILQQLDS---KDSVDIYGAVHDLRLH
PTPRB_Gg
PIKVSHF--EAHFTKLQADSNYLLSKEYEDLKDVGRNQTCDIALLPENRGKNRYNNILPYDTSRVKLSNVDDDPCSDYIN
ASYIPGN-NFRREYIATQGPLPGTKDEFWKMAWEQNVHNIVMVTQCVEKGRVKCDHYWPLD-QDSL-YYGDLIVEMLSES
VLPEWTIREFKIC--SEEQ-LDSTRLIRHFHYTVWPDHGVP--ETTQSLIQFVRTVRDY-INRTPDTGPTVVHCSAGVGR
TGTFIALDRILQQLDS---KDTVDIYAAVHDLRLH
PTPRB_Xt
PILTAQF--EEHFSKLQTDSNYLLSREYENLKDFGRDQSSDTALLPENRGKNRYSNILPYDSTRVKLANVDDDPCSDYIN
ASYMPGI-NFRREYIATQGPLPATKDDFWKMVWEQNVHIIVMVTQCTERGRAKCDHYWPMD-QDSY-YYGDLIVQMLSES
VLPEWTIREFKIC--SEDQ-IDAPRLVRHFHYTVWPDHGVP--ETTQSLIQFVRTVRDY-INRTPGSGPTVVHCSAGVGR
TGTFIVLDRMLQQVDT---VDSVDIFGAVRDLRIH
PTPRB_Dr
PVQAANF--ESHLAKLQSDSSYLLSEEFEGLKDVGRIQTQNAARLLGNRNKNRYNNILPYDSTRVRLSCLEDDPCSDYIN
ANYIPGN-NFRWEYIATQGPLPGTKDDFWRMVWEQNVHSLVMVTQCVERGMVKCDHYWPTD-SEPL-CYGDIVVQLLSEK
VFPEWTIRDFKIS--CESQ-LRYPRMVRQFHYTIWPDHGVP--DTTQSLIQFVRTVRDF-INRTNSPGISVVHCSAGVGR
TGTFIVLDRALQQLDR---NCTVDIYGCVFDLRLH
PTPRJ_Hs
LIRVENF--EAYFKKQQADSNCGFAEEYEDLKLVGISQPKYAAELAENRGKNRYNNVLPYDISRVKLS-VQTHSTDDYIN
ANYMPGY-HSKKDFIATQGPLPNTLKDFWRMVWEKNVYAIIMLTKCVEQGRTKCEEYWP-S-KQAQ-DYGDITVAMTSEI
VLPEWTIRDFTVK--NIQT--SESHPLRQFHFTSWPDHGVP--DTTDLLINFRYLVRDY-MKQSPPESPILVHCSAGVGR
TGTFIAIDRLIYQIEN---ENTVDVYGIVYDLRMH
PTPRJ_Mm
LIRVENF--EAYFKKQQADSNCGFAEEYEDLKLIGISLPKYTAEIAENRGKNRYNNVLPYDISRVKLS-VQTHSTDDYIN
ANYMPGY-HSKKDFIATQGPLPNTLKDFWRMVWEKNVYAIVMLTKCVEQGRTKCEEYWP-S-KQAQ-DYGDITVAMTSEV
VLPEWTIRDFVVK--NMQN--SESHPLRQFHFTSWPDHGVP--DTTDLLINFRYLVRDY-MKQIPPESPILVHCSAGVGR
TGTFIAIDRLIYQIEN---ENTVDVYGIVYDLRMH
PTPRJ_Gg
MIKVENF--ESYFKKQQADSNCGFAEEYEELKSAGVHQPKFAAEIAENRGKNRYNNVLPYDISRVKLS-NPSCTTDDYIN
ANYMPGY-SSKKAFIAAQGPLPNTIEDFWRMIWEKNIYSIVMLTKCVEQARTKCEQYWP-D-KQSK-SYGDIIVTMVSEV
VLPEWTIRDFNVE--NADT--MESHTVRQFHFTSWPDHGVP--ETTDLLINFRHLVHEY-SSQNPIDSPILVHCSAGVGR
TGTFIAIDRLIQQIEM---ENTVDVYGVVYDLRMH
PTPRQ_Hs
PISKKSF--LQHVEELCTNNNLKFQEEFSELPKFLQDLSSTDADLPWNRAKNRFPNIKPYNNNRVKLIADASVPGSDYIN
ASYISGY-LCPNEFIATQGPLPGTVGDFWRMVWETRAKTLVMLTQCFEKGRIRCHQYWPED-NKPVTVFGDIVITKLMED
VQIDWTIRDLKIE--RHGD----CMTVRQCNFTAWPEHGVP--ENSAPLIHFVKLVRAS---RAHDTTPMIVHCSAGVGR
TGVFIALDHLTQHIND---HDFVDIYGLVAELRSE
PTPRQ_Mm
PVSKKSF--LQHVEELCTNNNLKFQEEFSELPKFLQDLSSTDADLPWNRAKNRFPNIKPYNNNRVKLIADVSIPGSDYIN
ASYVSGY-LCPNEFIATQGPLPGTVGDFWRMVWETRAKTLVMLTQCFEKGRIRCHQYWPED-NKPVTVFGDILITKLMED
IQIDWTIRDLKIE--RHGD----CMTVRQCNFTGWPEHGVP--ENTTPLIHFVKLVRTS---RAHDATPMVVHCSAGVGR
TGVFIALDHLTQHIHD---HDFVDIYGLVAELRSE
PTPRQ_Gg
PISKKSF--LQHVEELCTNNNLKFQEEFSELPKFLEDLASTDADLPWNRSKNRFPNIKPYNNNRVKLMPDAGIPGSDYIN
ASYVSGY-LCPNEFIATQGPLPGTVGDFWRMVWETRAKTLVMLTQCFEKGRIRCHQYWPED-NKPVTVFGDIVITKLVED
IQIDWTIRDLKIE--RHGD----CMMVRQCNFTSWPEHGVP--ETTAPIIHFVKLIRAS---RAHDNTPMVVHCSAGVGR
TGVYIALDHLTQHIND---HDFVDIYGLVAELRSE
PTPRQ_Dr
PISKKSF--LQHVEDLCANDNAKFQEEFAELPKLLQDLATSDADLPWNRSKNRFTNIKPYNNSRVKLLSEPGMPGSDYIN
ASFVSGY-LCPNEFIATQGPLPSTVADFWRMIWETGTKTIVMLTQCFEKGRIRCHQYWPED-NKPVTVFADIIITKLTED
VRPDWTVRALKVE--RHGS----YMIVHHFNYTSWPEHGVP--ESSSTLVQFVKAVRSN---RGHENTTIVVHCSAGVGR
TGVFIALDHLIQHLRD---HEFVDIYGLVAELRSE
PTPRH_Hs
DIPAEDF--ADHVRKNERDSNCGFADKYQQLSLVGHSQSQMVASASENNAKNRYRNVLPYDWSRVPLKPIHEEPGSDYIN
ASFMPGL-WSPQEFIATQGPLPQTVGDFWRLVWEQQSHTLVMLTNCMEAGRVKCEHYWPLD-SQPC-THGHLRVTLVGEE
VMENWTVRELLLL--QVEE--QKTLSVRQFHYQAWPDHGVP--SSPDTLLAFWRMLRQW-LDQTMEGGPPIVHCSAGVGR

TGTLIALDVLLRQLQS---EGLLGPFSFVRKMRES
PTPRH_Mm
DILAKDF--ADHVRENEKDSNCGFAEEYQQLALEGQGQSQITASALENRSKNRYRNVLPYDWSRVPLQPLQEEPGSDYIN
ASFMPGL-WSPKEFIATQGPLPNTVGDFWRMVWEQQSHTLVMLTNCMESGRVKCEHYWPLD-AQPC-IHGQLQVMLISEE
ASENWTVRHLQLF--HMKE--QQTLSLRQFHYLAWPDHGVP--YSPDPLLAFRKMLRQW-MDQTTDGGPPIVHCSAGVGR
TGTLIALDVLLRQLEC---EGLVGPFSFVKKMRES
PTPRH_Xt
SVMKSAF--PDYYQRQHADSDFGFAEEYQQLSNVGINQSKLAAELSENRSKNRFTNVLPYDHSRVRLNRIDADETSDYIN
ANYMPGY-NSSKEFIASQGPLPNTSADFWRMIWENQVSTIVMLTNCMENGRVKCEHYWPLD-YTPC-TYGDITVTVTSEM
ILPDWTVRDFTLK--HAKQ--QGNKHARHFHFTVWPDHGVP--ENTTTIVEFRNLVREY-MDLKRSSGPTVVHCSAGVGR
TGTLIALDYLIQKMEK---EQRIGIYSFVQKMRQN
PTPRH_Dr
TIACDKF--PEHFRNMSRDDNRAFSAEYDDLSSVGVEQSKVAALLPENKDKNRFSNVLPYDTSRVHLT-INKAGDSDYIN
ANYMPGYGNASREYIAAQGPLPSTVNDFWRMIWEKKSSTIVMVTNCTEGGRVKCEQYWPLD-YTPC-LYENLLVTVKSEN
KSQSWTLREFNVK--NKMT--SETRTVRHFHFTAWPDHGVP--RGTEELIQFRDLIRQH-IESHFSTGPTVVHCSAGVGR
TGTLIALDVLLQQLNR---EKAVGVAAFVQEMRLN
PTPRO_Hs
PVQLDDF--DAYIKDMAKDSDYKFSLQFEELKLIGLDIPHFAADLPLNRCKNRYTNILPYDFSRVRLVSMNEEEGADYIN
ANYIPGY-NSPQEYIATQGPLPETRNDFWKMVLQQKSQIIVMLTQCNEKRRVKCDHYWPFT-EEPI-AYGDITVEMISEE
EQDDWACRHFRIN--YADE----MQDVMHFNYTAWPDHGVPTANAAESILQFVHMVRQQ---ATKSKGPMIIHCSAGVGR
TGTFIALDRLLQHIRD---HEFVDILGLVSEMRSY
PTPRO_Mm
PVQLDDF--DSYIKDMAKDSDYKFSLQFEELKLIGLDIPHFAADLPLNRCKNRYTNILPYDFSRVRLVSMNEEEGADYIN
ANYIPGY-NSPQEYIATQGPLPETRNDFWKMVLQQKSHIIVMLTQCNEKRRVKCDHYWPFT-EEPI-AYGDITVEMVSEE
EEEDWASRHFRIN--YADE----AQDVMHFNYTAWPDHGVPPANAAESILQFVFTVRQQ---AAKSKGPMIIHCSAGVGR
TGTFIALDRLLQHIRD---HEFVDILGLVSEMRSY
PTPRO_Gg
PVQLDDF--DGYIKDMAKDSDYKFSLQFEELKLIGLDIPHFAADLPMNRCKNRYTNILPYDFSRVRLVSMNEEEGSDYIN
ANYIPGY-NSPQEYIATQGPLPETRNDFWKMVLQQKSQIIVMLTQCNEKRRVKCDHYWPFT-EDPI-AYGDITVEMLSEE
EHTDWVYRNFRIS--YADE----VQDVMHFNYTAWPDHGVPTANAAESILQFVQMVRQK---SVKSKGPMIIHCSAGVGR
TGTFIALDWLLQHIRD---HEFVDILGLVSDMRSY
PTPRO_Xt
PPQ--RFFKKKGGKEMSKDSDYKFSLQFEELKMVGLDIPHFAADLPSNRGKNRYTNILPYDCSRVKLISLEADEGADYIN
ANYIPGY-SAAQEYIATQGPLPETRNDFWKMILQQKCQVIVMLTQCNEKRRIKCDHYWPFT-PEPV-NYGDITVEMASEE
EQSDWAQRVFRVS--YADE----TQCVTHFNFTAWPDHGVPTVNAAESVLQFVQVVRQK---ALKSKGPVTVHCSAGVGR
TGTFIALDWLMQHIRD---HEFVDILGLVSELRSH
PTPRO_Dr
PVQLSDF--EAYLKDMGKDSAYKFSLQFEELKSVGLDLSHEAADLPINRPKNRYTNILPYDFSRVKLISLHNDEGSDYIN
ANYIPGY-NSPREYIATQGPLPDTRNDFWKMVLQQKVHIIVMLTQCNERRRVKCDHYWPFS-DEPV-AYGEISVEMLAET
DSPEWTIRSFRLA--YADE----TQDVLHFNYTSWPDHGVPTVNAIESILQFVQIVRQQ---VNRSKGPIVVHCSAGVGR
TGTFISLDRLMQHIQE---HEYVDVLGLVSDMRSH

[DIMENSIONS NCHAR=271;
FORMAT DATATYPE = Protein GAP = - MISSING = ?;
MATRIX]
capsasporaPTPc1
IVPAEELADCVAQLHANSDLGFAEEYEAL-NVNEEYATNAALMPANKSKNRYANILPYDHSRVRLSVIPGVEGSDYVNAN
YIDGY-RKSHAYIACQGPLPDTFDDFWRMVWEQRSAVIVMVTNEEEKGRVKCHRYWP--DDSEV-VFGDVEVRMTRQEEL
SEFITRVFSLKNIRT--GETRTVHHLQFTGWPDHGVP--HSTSSLIKFVKQAKAVQ---PNDAGPMVIHCSAGVGRTGTF
IVTDMSLERTKV---ENNVDIFGCVSALRRN
Sponge
-VYLRDFPSYVEDMLNDAGFKFSEEYEKVSAVGLDHSKDASLLPENRAKNRYTNILAYDHSRVKLESIDDEPGSDYINAN
YIPGY-RMRRAYIATQGPLPSTFDDFWRMTWEQNSHVIVMLTQLVERGRTKCHRYWP--GAQPE-VYGEINVDMLSETEK
SDWIVRKFKITKEKR----SRTITHYQFVSWPDHGVP--DEAGPALDFVREVHEVA---SSAFGPVIVHCSAGVGRTGTF
IALGTLLQHIKD---HDWVDLFGLASEMRQH
Acornworm
PVPLKGFRDYVNELDEDDRKGFEEEYDDLRKLVVRQPTSVGQRMEHRSKNRYTNIVPYDNNRVILSG----SGNSYINAS
YISGS-KGSRSYIATQGPLESTCGDFWKMIWQQRVTTVVMMTQCNELGKSKCHHYWPR-DTQTQISHSDLTIRLTSETRL
PDWIIRDFSIESNGE----IRALRQFHFTSWPITGTP--YDADPLIRFIEAIRIQV---LPNSGPILVHCSAGVGRTGVF
IALYHLLEYFYT---MIQVDIFGKVIKMRKQ
Seaurchin4
PTKKNMFAQHVAKMSARNNAGFSDEYNNLPAMEKTRAVTAARQARNDDKNRYRNILPYDYTRVQLKGV---AGTDYINAS
YIKDD-HGQKKYIATQGPLPNTIEHFWEMVWENQTSTIVMMTALVEGGKTKCEHYWPA-GEEPQ-LHGNVTVTLVGSNQT
DNFIERTLLLEKEGT----ERTVTHYQYLAWTDHGVP--ESTAPLVGLLRQVKVTNQEDGAATGPIVVHCSAGVGRTGTF
IAADMLMDAIQRSTATDYIDVAGTIAKIREQ
seaurchin3
PVQMRKFPDHVNRMSAGNNTGFVEEYNSLSNVGRDKSTDASRLAVNATKNRYRNILPFDETAVHLQDLEEGQTSDYINAN
YIQGL-HSMKEYIASQGPVPDSVNDFWEMVFENKSTTIAMITGLVEGGKTKCEHYWPD-DDTPV-NYGSVTVTMTHTQEM

EQWTVRSFLLEKGMQ----QFETHQYAFKGWVDHDVP--NNARPMIEFIRTIDVAH---DANLGPITVHCSAGIGRTGVF
IALHKLIKQVETSKPNDYIDVCGTVARMREQ
seaurchin1
PIVLNKFEGHYGRMKADSDFRFTEEYDEIRLVGKDQAIVSALEMVNRAKNRFTNILPYEHSRVKLAALADDSDTDYINAN
YIPGY-NSPREFMACQGPLPGTVDDMWRMIWEKKTSIIVMLTQLVEKGKIKCHEYWPA-DYNPV-TYGSIQVSVQALQQY
DHWVIREFSISQGDE----IRKLTQYHFMSWPDHGVP--DKTWTMLDFVRTVREAIQ-KTASDRPIVAHCSAGVGRTGTY
IALDRLMQAMQE---NDYIDIFGIICEMRMQ
CElegans
PVRIEDFADHVRLMSADSDFRFSEEYDMMRNVGVGQSVAASELPINRPKNRFTNIPSYDHSRVKLSNPNNIEGGDYINAN
YVPGF-SSRREFIAAQGPLPTTRDHFWQMTWEQQCPAIIALTKCVEKGRDKCHQYWPDHENVPV-LYGDIEVTIVAEKEF
DEFVIRDIRLEKSGPDGRVTRFVRHWHYMAWPDFGAP--SHPNGIIQFSRMFRHHLP-HSPHNAPTIVHCSAGVGRSGTF
ISIDRLLQSSSF---GDPIDVFGTVCEMRYE
Ciona
PVFRDEFPNHVSSLSQNKNKGFSLEFDDIRGIPYAGTTAIAENSCNKTKNRTTKLVPFDHCRVKIEGIPAIQGSNYINAS
YIPGL-DSPEQYIATQTPLDHTKKDFWRMLWETGSTNIVMLCGSVNAGKKRCDEFWP--KKQTE-YFGNLAVQMKEEIRH
DEWIIRQFIVTMRDK----VRHVTQHHFIKWPQLEQA--ENSLPLVRFIKNYRLLR---DRSINPTIVMCSNGSGRCGVF
IGLLRILDNN-----GRDVDVFGTVAALRKY
Fly10D
PILIKNFAEHYRLMSADSDFRFSEEFEELKHVGRDQPCTFADLPCNRPKNRFTNILPYDHSRFKLQPVDDDEGSDYINAN
YVPGH-NSPREFIVTQGPLHSTRDDFWRMCWESNSRAIVMLTRCFEKGREKCDQYWPN-DTVPV-FYGDIKVQILNDSHY
ADWVMTEFMLCRGSE----QRILRHFHFTTWPDFGVP--NPPQTLVRFVRAFRDRI---GAEQRPIVVHCSAGVGRSGTF
ITLDRILQQINT---SDYVDIFGIVYAMRKE
FlyDPTP4E
PVHVKDFSEHYRIMSADSDFRFSEEFEELKHVGRDQACSFANLPCNRPKNRFTNILPYDHSRFKLQPVDDDDGSDYINAN
YMPGH-NSPREFIVTQGPLHSTREEFWRMCWESNSRAIVMLTRCFEKGREKCDQYWPV-DRVAM-FYGDIKVQLIIDTHY
HDWSISEFMVSRNCE----SRIMRHFHFTTWPDFGVP--EPPQSLVRFVRAFRDVI---GTDMRPIIVHCSAGVGRSGTF
IALDRILQHIHK---SDYVDIFGIVFAMRKE
PTPRB_Hs
PIKINQFEGHFMKLQADSNYLLSKEYEELKDVGRNQSCDIALLPENRGKNRYNNILPYDATRVKLSNVDDDPCSDYINAS
YIPGN-NFRREYIVTQGPLPGTKDDFWKMVWEQNVHNIVMVTQCVEKGRVKCDHYWPA-DQDSL-YYGDLILQMLSESVL
PEWTIREFKICGEEQ-LDAHRLIRHFHYTVWPDHGVP--ETTQSLIQFVRTVRDYIN-RSPGAGPTVVHCSAGVGRTGTF
IALDRILQQLDS---KDSVDIYGAVHDLRLH
PTPRB_Mm
PIKINQFEGHFMKLQADSNYLLSKEYEDLKDVGRSQSCDIALLPENRGKNRYNNILPYDASRVKLCNVDDDPCSDYINAS
YIPGN-NFRREYIATQGPLPGTKDDFWKMAWEQNVHNIVMVTQCVEKGRVKCDHYWPA-DQDPL-YYGDLILQMVSESVL
PEWTIREFKICSEEQ-LDAHRLIRHFHYTVWPDHGVP--ETTQSLIQFVRTVRDYIN-RSPGAGPTVVHCSAGVGRTGTF
VALDRILQQLDS---KDSVDIYGAVHDLRLH
PTPRB_Gg
PIKVSHFEAHFTKLQADSNYLLSKEYEDLKDVGRNQTCDIALLPENRGKNRYNNILPYDTSRVKLSNVDDDPCSDYINAS
YIPGN-NFRREYIATQGPLPGTKDEFWKMAWEQNVHNIVMVTQCVEKGRVKCDHYWPL-DQDSL-YYGDLIVEMLSESVL
PEWTIREFKICSEEQ-LDSTRLIRHFHYTVWPDHGVP--ETTQSLIQFVRTVRDYIN-RTPDTGPTVVHCSAGVGRTGTF
IALDRILQQLDS---KDTVDIYAAVHDLRLH
PTPRB_Xt
PILTAQFEEHFSKLQTDSNYLLSREYENLKDFGRDQSSDTALLPENRGKNRYSNILPYDSTRVKLANVDDDPCSDYINAS
YMPGI-NFRREYIATQGPLPATKDDFWKMVWEQNVHIIVMVTQCTERGRAKCDHYWPM-DQDSY-YYGDLIVQMLSESVL
PEWTIREFKICSEDQ-IDAPRLVRHFHYTVWPDHGVP--ETTQSLIQFVRTVRDYIN-RTPGSGPTVVHCSAGVGRTGTF
IVLDRMLQQVDT---VDSVDIFGAVRDLRIH
PTPRB_Dr
PVQAANFESHLAKLQSDSSYLLSEEFEGLKDVGRIQTQNAARLLGNRNKNRYNNILPYDSTRVRLSCLEDDPCSDYINAN
YIPGN-NFRWEYIATQGPLPGTKDDFWRMVWEQNVHSLVMVTQCVERGMVKCDHYWPT-DSEPL-CYGDIVVQLLSEKVF
PEWTIRDFKISCESQ-LRYPRMVRQFHYTIWPDHGVP--DTTQSLIQFVRTVRDFIN-RTNSPGISVVHCSAGVGRTGTF
IVLDRALQQLDR---NCTVDIYGCVFDLRLH
PTPRJ_Hs
LIRVENFEAYFKKQQADSNCGFAEEYEDLKLVGISQPKYAAELAENRGKNRYNNVLPYDISRVKLS-VQTHSTDDYINAN
YMPGY-HSKKDFIATQGPLPNTLKDFWRMVWEKNVYAIIMLTKCVEQGRTKCEEYWP--SKQAQ-DYGDITVAMTSEIVL
PEWTIRDFTVKNIQT--SESHPLRQFHFTSWPDHGVP--DTTDLLINFRYLVRDYMK-QSPPESPILVHCSAGVGRTGTF
IAIDRLIYQIEN---ENTVDVYGIVYDLRMH
PTPRJ_Mm
LIRVENFEAYFKKQQADSNCGFAEEYEDLKLIGISLPKYTAEIAENRGKNRYNNVLPYDISRVKLS-VQTHSTDDYINAN
YMPGY-HSKKDFIATQGPLPNTLKDFWRMVWEKNVYAIVMLTKCVEQGRTKCEEYWP--SKQAQ-DYGDITVAMTSEVVL
PEWTIRDFVVKNMQN--SESHPLRQFHFTSWPDHGVP--DTTDLLINFRYLVRDYMK-QIPPESPILVHCSAGVGRTGTF
IAIDRLIYQIEN---ENTVDVYGIVYDLRMH
PTPRJ_Gg
MIKVENFESYFKKQQADSNCGFAEEYEELKSAGVHQPKFAAEIAENRGKNRYNNVLPYDISRVKLS-NPSCTTDDYINAN
YMPGY-SSKKAFIAAQGPLPNTIEDFWRMIWEKNIYSIVMLTKCVEQARTKCEQYWP--DKQSK-SYGDIIVTMVSEVVL
PEWTIRDFNVENADT--MESHTVRQFHFTSWPDHGVP--ETTDLLINFRHLVHEYSS-QNPIDSPILVHCSAGVGRTGTF
IAIDRLIQQIEM---ENTVDVYGVVYDLRMH
PTPRQ_Hs

```
PISKKSFLQHVEELCTNNNLKFQEEFSELPKFLQDLSSTDADLPWNRAKNRFPNIKPYNNNRVKLIADASVPGSDYINAS
YISGY-LCPNEFIATQGPLPGTVGDFWRMVWETRAKTLVMLTQCFEKGRIRCHQYWPE-DNKPVTVFGDIVITKLMEDVQ
IDWTIRDLKIERHGD----CMTVRQCNFTAWPEHGVP--ENSAPLIHFVKLVRASR---AHDTTPMIVHCSAGVGRTGVF
IALDHLTQHIND---HDFVDIYGLVAELRSE
        PTPRQ_Mm
PVSKKSFLQHVEELCTNNNLKFQEEFSELPKFLQDLSSTDADLPWNRAKNRFPNIKPYNNNRVKLIADVSIPGSDYINAS
YVSGY-LCPNEFIATQGPLPGTVGDFWRMVWETRAKTLVMLTQCFEKGRIRCHQYWPE-DNKPVTVFGDILITKLMEDIQ
IDWTIRDLKIERHGD----CMTVRQCNFTGWPEHGVP--ENTTPLIHFVKLVRTSR---AHDATPMVVHCSAGVGRTGVF
IALDHLTQHIHD---HDFVDIYGLVAELRSE
        PTPRQ_Gg
PISKKSFLQHVEELCTNNNLKFQEEFSELPKFLEDLASTDADLPWNRSKNRFPNIKPYNNNRVKLMPDAGIPGSDYINAS
YVSGY-LCPNEFIATQGPLPGTVGDFWRMVWETRAKTLVMLTQCFEKGRIRCHQYWPE-DNKPVTVFGDIVITKLVEDIQ
IDWTIRDLKIERHGD----CMMVRQCNFTSWPEHGVP--ETTAPIIHFVKLIRASR---AHDNTPMVVHCSAGVGRTGVY
IALDHLTQHIND---HDFVDIYGLVAELRSE
        PTPRQ_Dr
PISKKSFLQHVEDLCANDNAKFQEEFAELPKLLQDLATSDADLPWNRSKNRFTNIKPYNNSRVKLLSEPGMPGSDYINAS
FVSGY-LCPNEFIATQGPLPSTVADFWRMIWETGTKTIVMLTQCFEKGRIRCHQYWPE-DNKPVTVFADIIITKLTEDVR
PDWTVRALKVERHGS----YMIVHHFNYTSWPEHGVP--ESSSTLVQFVKAVRSNR---GHENTTIVVHCSAGVGRTGVF
IALDHLIQHLRD---HEFVDIYGLVAELRSE
        PTPRH_Hs
DIPAEDFADHVRKNERDSNCGFADKYQQLSLVGHSQSQMVASASENNAKNRYRNVLPYDWSRVPLKPIHEEPGSDYINAS
FMPGL-WSPQEFIATQGPLPQTVGDFWRLVWEQQSHTLVMLTNCMEAGRVKCEHYWPL-DSQPC-THGHLRVTLVGEEVM
ENWTVRELLLLQVEE--QKTLSVRQFHYQAWPDHGVP--SSPDTLLAFWRMLRQWLD-QTMEGGPPIVHCSAGVGRTGTL
IALDVLLRQLQS---EGLLGPFSFVRKMRES
        PTPRH_Mm
DILAKDFADHVRENEKDSNCGFAEEYQQLALEGQGQSQITASALENRSKNRYRNVLPYDWSRVPLQPLQEEPGSDYINAS
FMPGL-WSPKEFIATQGPLPNTVGDFWRMVWEQQSHTLVMLTNCMESGRVKCEHYWPL-DAQPC-IHGQLQVMLISEEAS
ENWTVRHLQLFHMKE--QQTLSLRQFHYLAWPDHGVP--YSPDPLLAFRKMLRQWMD-QTTDGGPPIVHCSAGVGRTGTL
IALDVLLRQLEC---EGLVGPFSFVKKMRES
        PTPRH_Xt
SVMKSAFPDYYQRQHADSDFGFAEEYQQLSNVGINQSKLAAELSENRSKNRFTNVLPYDHSRVRLNRIDADETSDYINAN
YMPGY-NSSKEFIASQGPLPNTSADFWRMIWENQVSTIVMLTNCMENGRVKCEHYWPL-DYTPC-TYGDITVTVTSEMIL
PDWTVRDFTLKHAKQ--QGNKHARHFHFTVWPDHGVP--ENTTTIVEFRNLVREYMD-LKRSSGPTVVHCSAGVGRTGTL
IALDYLIQKMEK---EQRIGIYSFVQKMRQN
        PTPRH_Dr
TIACDKFPEHFRNMSRDDNRAFSAEYDDLSSVGVEQSKVAALLPENKDKNRFSNVLPYDTSRVHLT-INKAGDSDYINAN
YMPGYGNASREYIAAQGPLPSTVNDFWRMIWEKKSSTIVMVTNCTEGGRVKCEQYWPL-DYTPC-LYENLLVTVKSENKS
QSWTLREFNVKNKMT--SETRTVRHFHFTAWPDHGVP--RGTEELIQFRDLIRQHIE-SHFSTGPTVVHCSAGVGRTGTL
IALDVLLQQLNR---EKAVGVAAFVQEMRLN
        PTPRO_Hs
PVQLDDFDAYIKDMAKDSDYKFSLQFEELKLIGLDIPHFAADLPLNRCKNRYTNILPYDFSRVRLVSMNEEEGADYINAN
YIPGY-NSPQEYIATQGPLPETRNDFWKMVLQQKSQIIVMLTQCNEKRRVKCDHYWPF-TEEPI-AYGDITVEMISEEEQ
DDWACRHFRINYADE----MQDVMHFNYTAWPDHGVPTANAAESILQFVHMVRQQA---TKSKGPMIIHCSAGVGRTGTF
IALDRLLQHIRD---HEFVDILGLVSEMRSY
        PTPRO_Mm
PVQLDDFDSYIKDMAKDSDYKFSLQFEELKLIGLDIPHFAADLPLNRCKNRYTNILPYDFSRVRLVSMNEEEGADYINAN
YIPGY-NSPQEYIATQGPLPETRNDFWKMVLQQKSHIIVMLTQCNEKRRVKCDHYWPF-TEEPI-AYGDITVEMVSEEEE
EDWASRHFRINYADE----AQDVMHFNYTAWPDHGVPPANAAESILQFVFTVRQQA---AKSKGPMIIHCSAGVGRTGTF
IALDRLLQHIRD---HEFVDILGLVSEMRSY
        PTPRO_Gg
PVQLDDFDGYIKDMAKDSDYKFSLQFEELKLIGLDIPHFAADLPMNRCKNRYTNILPYDFSRVRLVSMNEEEGSDYINAN
YIPGY-NSPQEYIATQGPLPETRNDFWKMVLQQKSQIIVMLTQCNEKRRVKCDHYWPF-TEDPI-AYGDITVEMLSEEEH
TDWVYRNFRISYADE----VQDVMHFNYTAWPDHGVPTANAAESILQFVQMVRQKS---VKSKGPMIIHCSAGVGRTGTF
IALDWLLQHIRD---HEFVDILGLVSDMRSY
        PTPRO_Xt
PPQRFFKKKGGKEMSKDSDYKFSLQFEELKMVGLDIPHFAADLPSNRGKNRYTNILPYDCSRVKLISLEADEGADYINAN
YIPGY-SAAQEYIATQGPLPETRNDFWKMILQQKCQVIVMLTQCNEKRRIKCDHYWPF-TPEPV-NYGDITVEMASEEEQ
SDWAQRVFRVSYADE----TQCVTHFNFTAWPDHGVPTVNAAESVLQFVQVVRQKA---LKSKGPVTVHCSAGVGRTGTF
IALDWLMQHIRD---HEFVDILGLVSELRSH
        PTPRO_Dr
PVQLSDFEAYLKDMGKDSAYKFSLQFEELKSVGLDLSHEAADLPINRPKNRYTNILPYDFSRVKLISLHNDEGSDYINAN
YIPGY-NSPREYIATQGPLPDTRNDFWKMVLQQKVHIIVMLTQCNERRRVKCDHYWPF-SDEPV-AYGEISVEMLAETDS
PEWTIRSFRLAYADE----TQDVLHFNYTSWPDHGVPTVNAIESILQFVQIVRQQV---NRSKGPIVVHCSAGVGRTGTF
ISLDRLMQHIQE---HEYVDVLGLVSDMRSH


        [DIMENSIONS  NCHAR=271;
        FORMAT DATATYPE = Protein GAP = - MISSING = ?;
```

MATRIX]
capsasporaPTPc1
IVPAEELADCVAQLHANSDLGFAEEYEAL-NVNEEYATNAALMPANKSKNRYANILPYDHSRVRLSVIPGVEGSDYVNAN
YIDGY-RKSHAYIACQGPLPDTFDDFWRMVWEQRSAVIVMVTNEEEKGRVKCHRYWP--DDSEV-VFGDVEVRMTRQEEL
SEFITRVFSLKNIRT--GETRTVHHLQFTGWPDHGVP--HSTSSLIKFVKQAKAVQ---PNDAGPMVIHCSAGVGRTGTF
IVTDMSLERTKV---ENNVDIFGCVSALRRN
Sponge
-VYLRDFPSYVEDMLNDAGFKFSEEYEKVSAVGLDHSKDASLLPENRAKNRYTNILAYDHSRVKLESIDDEPGSDYINAN
YIPGY-RMRRAYIATQGPLPSTFDDFWRMTWEQNSHVIVMLTQLVERGRTKCHRYWP--GAQPE-VYGEINVDMLSETEK
SDWIVRKFKITKEKR----SRTITHYQFVSWPDHGVP--DEAGPALDFVREVHEVA---SSAFGPVIVHCSAGVGRTGTF
IALGTLLQHIKD---HDWVDLFGLASEMRQH
Acornworm
PVPLKGFRDYVNELDEDDRKGFEEEYDDLRKLVVRQPTSVGQRMEHRSKNRYTNIVPYDNNRVILSG----SGNSYINAS
YISGS-KGSRSYIATQGPLESTCGDFWKMIWQQRVTTVVMMTQCNELGKSKCHHYWP-RDTQTQISHSDLTIRLTSETRL
PDWIIRDFSIESNGE----IRALRQFHFTSWPITGTP--YDADPLIRFIEAIRIQV---LPNSGPILVHCSAGVGRTGVF
IALYHLLEYFYT---MIQVDIFGKVIKMRKQ
Seaurchin4
PTKKNMFAQHVAKMSARNNAGFSDEYNNLPAMEKTRAVTAARQARNDDKNRYRNILPYDYTRVQLKGV---AGTDYINAS
YIKDD-HGQKKYIATQGPLPNTIEHFWEMVWENQTSTIVMMTALVEGGKTKCEHYWP-AGEEPQ-LHGNVTVTLVGSNQT
DNFIERTLLLEKEGT----ERTVTHYQYLAWTDHGVP--ESTAPLVGLLRQVKVTNQEDGAATGPIVVHCSAGVGRTGTF
IAADMLMDAIQRSTATDYIDVAGTIAKIREQ
seaurchin3
PVQMRKFPDHVNRMSAGNNTGFVEEYNSLSNVGRDKSTDASRLAVNATKNRYRNILPFDETAVHLQDLEEGQTSDYINAN
YIQGL-HSMKEYIASQGPVPDSVNDFWEMVFENKSTTIAMITGLVEGGKTKCEHYWP-DDDTPV-NYGSVTVTMTHTQEM
EQWTVRSFLLEKGMQ----QFETHQYAFKGWVDHDVP--NNARPMIEFIRTIDVAH---DANLGPITVHCSAGIGRTGVF
IALHKLIKQVETSKPNDYIDVCGTVARMREQ
seaurchin1
PIVLNKFEGHYGRMKADSDFRFTEEYDEIRLVGKDQAIVSALEMVNRAKNRFTNILPYEHSRVKLAALADDSDTDYINAN
YIPGY-NSPREFMACQGPLPGTVDDMWRMIWEKKTSIIVMLTQLVEKGKIKCHEYWP-ADYNPV-TYGSIQVSVQALQQY
DHWVIREFSISQGDE----IRKLTQYHFMSWPDHGVP--DKTWTMLDFVRTVREAIQ-KTASDRPIVAHCSAGVGRTGTY
IALDRLMQAMQE---NDYIDIFGIICEMRMQ
CElegans
PVRIEDFADHVRLMSADSDFRFSEEYDMMRNVGVGQSVAASELPINRPKNRFTNIPSYDHSRVKLSNPNNIEGGDYINAN
YVPGF-SSRREFIAAQGPLPTTRDHFWQMTWEQQCPAIIALTKCVEKGRDKCHQYWPDHENVPV-LYGDIEVTIVAEKEF
DEFVIRDIRLEKSGPDGRVTRFVRHWHYMAWPDFGAP--SHPNGIIQFSRMFRHHLP-HSPHNAPTIVHCSAGVGRSGTF
ISIDRLLQSSSF---GDPIDVFGTVCEMRYE
Ciona
PVFRDEFPNHVSSLSQNKNKGFSLEFDDIRGIPYAGTTAIAENSCNKTKNRTTKLVPFDHCRVKIEGIPAIQGSNYINAS
YIPGL-DSPEQYIATQTPLDHTKKDFWRMLWETGSTNIVMLCGSVNAGKKRCDEFWP--KKQTE-YFGNLAVQMKEEIRH
DEWIIRQFIVTMRDK----VRHVTQHHFIKWPQLEQA--ENSLPLVRFIKNYRLLR---DRSINPTIVMCSNGSGRCGVF
IGLLRILDNN-----GRDVDVFGTVAALRKY
Fly10D
PILIKNFAEHYRLMSADSDFRFSEEFEELKHVGRDQPCTFADLPCNRPKNRFTNILPYDHSRFKLQPVDDDEGSDYINAN
YVPGH-NSPREFIVTQGPLHSTRDDFWRMCWESNSRAIVMLTRCFEKGREKCDQYWP-NDTVPV-FYGDIKVQILNDSHY
ADWVMTEFMLCRGSE----QRILRHFHFTTWPDFGVP--NPPQTLVRFVRAFRDRI---GAEQRPIVVHCSAGVGRSGTF
ITLDRILQQINT---SDYVDIFGIVYAMRKE
FlyDPTP4E
PVHVKDFSEHYRIMSADSDFRFSEEFEELKHVGRDQACSFANLPCNRPKNRFTNILPYDHSRFKLQPVDDDDGSDYINAN
YMPGH-NSPREFIVTQGPLHSTREEFWRMCWESNSRAIVMLTRCFEKGREKCDQYWP-VDRVAM-FYGDIKVQLIIDTHY
HDWSISEFMVSRNCE----SRIMRHFHFTTWPDFGVP--EPPQSLVRFVRAFRDVI---GTDMRPIIVHCSAGVGRSGTF
IALDRILQHIHK---SDYVDIFGIVFAMRKE
PTPRB_Hs
PIKINQFEGHFMKLQADSNYLLSKEYEELKDVGRNQSCDIALLPENRGKNRYNNILPYDATRVKLSNVDDDPCSDYINAS
YIPGN-NFRREYIVTQGPLPGTKDDFWKMVWEQNVHNIVMVTQCVEKGRVKCDHYWP-ADQDSL-YYGDLILQMLSESVL
PEWTIREFKICGEEQ-LDAHRLIRHFHYTVWPDHGVP--ETTQSLIQFVRTVRDYIN-RSPGAGPTVVHCSAGVGRTGTF
IALDRILQQLDS---KDSVDIYGAVHDLRLH
PTPRB_Mm
PIKINQFEGHFMKLQADSNYLLSKEYEDLKDVGRSQSCDIALLPENRGKNRYNNILPYDASRVKLCNVDDDPCSDYINAS
YIPGN-NFRREYIATQGPLPGTKDDFWKMAWEQNVHNIVMVTQCVEKGRVKCDHYWP-ADQDPL-YYGDLILQMVSESVL
PEWTIREFKICSEEQ-LDAHRLIRHFHYTVWPDHGVP--ETTQSLIQFVRTVRDYIN-RSPGAGPTVVHCSAGVGRTGTF
VALDRILQQLDS---KDSVDIYGAVHDLRLH
PTPRB_Gg
PIKVSHFEAHFTKLQADSNYLLSKEYEDLKDVGRNQTCDIALLPENRGKNRYNNILPYDTSRVKLSNVDDDPCSDYINAS
YIPGN-NFRREYIATQGPLPGTKDEFWKMAWEQNVHNIVMVTQCVEKGRVKCDHYWP-LDQDSL-YYGDLIVEMLSESVL
PEWTIREFKICSEEQ-LDSTRLIRHFHYTVWPDHGVP--ETTQSLIQFVRTVRDYIN-RTPDTGPTVVHCSAGVGRTGTF
IALDRILQQLDS---KDTVDIYAAVHDLRLH
PTPRB_Xt
PILTAQFEEHFSKLQTDSNYLLSREYENLKDFGRDQSSDTALLPENRGKNRYSNILPYDSTRVKLANVDDDPCSDYINAS

YMPGI-NFRREYIATQGPLPATKDDFWKMVWEQNVHIIVMVTQCTERGRAKCDHYWP-MDQDSY-YYGDLIVQMLSESVL
PEWTIREFKICSEDQ-IDAPRLVRHFHYTVWPDHGVP--ETTQSLIQFVRTVRDYIN-RTPGSGPTVVHCSAGVGRTGTF
IVLDRMLQQVDT---VDSVDIFGAVRDLRIH
PTPRB_Dr
PVQAANFESHLAKLQSDSSYLLSEEFEGLKDVGRIQTQNAARLLGNRNKNRYNNILPYDSTRVRLSCLEDDPCSDYINAN
YIPGN-NFRWEYIATQGPLPGTKDDFWRMVWEQNVHSLVMVTQCVERGMVKCDHYWP-TDSEPL-CYGDIVVQLLSEKVF
PEWTIRDFKISCESQ-LRYPRMVRQFHYTIWPDHGVP--DTTQSLIQFVRTVRDFIN-RTNSPGISVVHCSAGVGRTGTF
IVLDRALQQLDR---NCTVDIYGCVFDLRLH
PTPRJ_Hs
LIRVENFEAYFKKQQADSNCGFAEEYEDLKLVGISQPKYAAELAENRGKNRYNNVLPYDISRVKLS-VQTHSTDDYINAN
YMPGY-HSKKDFIATQGPLPNTLKDFWRMVWEKNVYAIIMLTKCVEQGRTKCEEYWP--SKQAQ-DYGDITVAMTSEIVL
PEWTIRDFTVKNIQT--SESHPLRQFHFTSWPDHGVP--DTTDLLINFRYLVRDYMK-QSPPESPILVHCSAGVGRTGTF
IAIDRLIYQIEN---ENTVDVYGIVYDLRMH
PTPRJ_Mm
LIRVENFEAYFKKQQADSNCGFAEEYEDLKLIGISLPKYTAEIAENRGKNRYNNVLPYDISRVKLS-VQTHSTDDYINAN
YMPGY-HSKKDFIATQGPLPNTLKDFWRMVWEKNVYAIVMLTKCVEQGRTKCEEYWP--SKQAQ-DYGDITVAMTSEVVL
PEWTIRDFVVKNMQN--SESHPLRQFHFTSWPDHGVP--DTTDLLINFRYLVRDYMK-QIPPESPILVHCSAGVGRTGTF
IAIDRLIYQIEN---ENTVDVYGIVYDLRMH
PTPRJ_Gg
MIKVENFESYFKKQQADSNCGFAEEYEELKSAGVHQPKFAAEIAENRGKNRYNNVLPYDISRVKLS-NPSCTTDDYINAN
YMPGY-SSKKAFIAAQGPLPNTIEDFWRMIWEKNIYSIVMLTKCVEQARTKCEQYWP--DKQSK-SYGDIIVTMVSEVVL
PEWTIRDFNVENADT--MESHTVRQFHFTSWPDHGVP--ETTDLLINFRHLVHEYSS-QNPIDSPILVHCSAGVGRTGTF
IAIDRLIQQIEM---ENTVDVYGVVYDLRMH
PTPRQ_Hs
PISKKSFLQHVEELCTNNNLKFQEEFSELPKFLQDLSSTDADLPWNRAKNRFPNIKPYNNNRVKLIADASVPGSDYINAS
YISGY-LCPNEFIATQGPLPGTVGDFWRMVWETRAKTLVMLTQCFEKGRIRCHQYWP-EDNKPVTVFGDIVITKLMEDVQ
IDWTIRDLKIERHGD----CMTVRQCNFTAWPEHGVP--ENSAPLIHFVKLVRASR---AHDTTPMIVHCSAGVGRTGVF
IALDHLTQHIND---HDFVDIYGLVAELRSE
PTPRQ_Mm
PVSKKSFLQHVEELCTNNNLKFQEEFSELPKFLQDLSSTDADLPWNRAKNRFPNIKPYNNNRVKLIADVSIPGSDYINAS
YVSGY-LCPNEFIATQGPLPGTVGDFWRMVWETRAKTLVMLTQCFEKGRIRCHQYWP-EDNKPVTVFGDILITKLMEDIQ
IDWTIRDLKIERHGD----CMTVRQCNFTGWPEHGVP--ENTTPLIHFVKLVRTSR---AHDATPMVVHCSAGVGRTGVF
IALDHLTQHIHD---HDFVDIYGLVAELRSE
PTPRQ_Gg
PISKKSFLQHVEELCTNNNLKFQEEFSELPKFLEDLASTDADLPWNRSKNRFPNIKPYNNNRVKLMPDAGIPGSDYINAS
YVSGY-LCPNEFIATQGPLPGTVGDFWRMVWETRAKTLVMLTQCFEKGRIRCHQYWP-EDNKPVTVFGDIVITKLVEDIQ
IDWTIRDLKIERHGD----CMMVRQCNFTSWPEHGVP--ETTAPIIHFVKLIRASR---AHDNTPMVVHCSAGVGRTGVY
IALDHLTQHIND---HDFVDIYGLVAELRSE
PTPRQ_Dr
PISKKSFLQHVEDLCANDNAKFQEEFAELPKLLQDLATSDADLPWNRSKNRFTNIKPYNNSRVKLLSEPGMPGSDYINAS
FVSGY-LCPNEFIATQGPLPSTVADFWRMIWETGTKTIVMLTQCFEKGRIRCHQYWP-EDNKPVTVFADIIITKLTEDVR
PDWTVRALKVERHGS----YMIVHHFNYTSWPEHGVP--ESSSTLVQFVKAVRSNR---GHENTTIVVHCSAGVGRTGVF
IALDHLIQHLRD---HEFVDIYGLVAELRSE
PTPRH_Hs
DIPAEDFADHVRKNERDSNCGFADKYQQLSLVGHSQSQMVASASENNAKNRYRNVLPYDWSRVPLKPIHEEPGSDYINAS
FMPGL-WSPQEFIATQGPLPQTVGDFWRLVWEQQSHTLVMLTNCMEAGRVKCEHYWP-LDSQPC-THGHLRVTLVGEEVM
ENWTVRELLLLQVEE--QKTLSVRQFHYQAWPDHGVP--SSPDTLLAFWRMLRQWLD-QTMEGGPPIVHCSAGVGRTGTL
IALDVLLRQLQS---EGLLGPFSFVRKMRES
PTPRH_Mm
DILAKDFADHVRENEKDSNCGFAEEYQQLALEGQGQSQITASALENRSKNRYRNVLPYDWSRVPLQPLQEEPGSDYINAS
FMPGL-WSPKEFIATQGPLPNTVGDFWRMVWEQQSHTLVMLTNCMESGRVKCEHYWP-LDAQPC-IHGQLQVMLISEEAS
ENWTVRHLQLFHMKE--QQTLSLRQFHYLAWPDHGVP--YSPDPLLAFRKMLRQWMD-QTTDGGPPIVHCSAGVGRTGTL
IALDVLLRQLEC---EGLVGPFSFVKKMRES
PTPRH_Xt
SVMKSAFPDYYQRQHADSDFGFAEEYQQLSNVGINQSKLAAELSENRSKNRFTNVLPYDHSRVRLNRIDADETSDYINAN
YMPGY-NSSKEFIASQGPLPNTSADFWRMIWENQVSTIVMLTNCMENGRVKCEHYWP-LDYTPC-TYGDITVTVTSEMIL
PDWTVRDFTLKHAKQ--QGNKHARHFHFTVWPDHGVP--ENTTTIVEFRNLVREYMD-LKRSSGPTVVHCSAGVGRTGTL
IALDYLIQKMEK---EQRIGIYSFVQKMRQN
PTPRH_Dr
TIACDKFPEHFRNMSRDDNRAFSAEYDDLSSVGVEQSKVAALLPENKDKNRFSNVLPYDTSRVHLT-INKAGDSDYINAN
YMPGYGNASREYIAAQGPLPSTVNDFWRMIWEKKSSTIVMVTNCTEGGRVKCEQYWP-LDYTPC-LYENLLVTVKSENKS
QSWTLREFNVKNKMT--SETRTVRHFHFTAWPDHGVP--RGTEELIQFRDLIRQHIE-SHFSTGPTVVHCSAGVGRTGTL
IALDVLLQQLNR---EKAVGVAAFVQEMRLN
PTPRO_Hs
PVQLDDFDAYIKDMAKDSDYKFSLQFEELKLIGLDIPHFAADLPLNRCKNRYTNILPYDFSRVRLVSMNEEEGADYINAN
YIPGY-NSPQEYIATQGPLPETRNDFWKMVLQQKSQIIVMLTQCNEKRRVKCDHYWP-FTEEPI-AYGDITVEMISEEEQ
DDWACRHFRINYADE----MQDVMHFNYTAWPDHGVPTANAAESILQFVHMVRQQA---TKSKGPMIIHCSAGVGRTGTF
IALDRLLQHIRD---HEFVDILGLVSEMRSY

PTPRO_Mm
PVQLDDFDSYIKDMAKDSDYKFSLQFEELKLIGLDIPHFAADLPLNRCKNRYTNILPYDFSRVRLVSMNEEEGADYINAN
YIPGY-NSPQEYIATQGPLPETRNDFWKMVLQQKSHIIVMLTQCNEKRRVKCDHYWP-FTEEPI-AYGDITVEMVSEEEE
EDWASRHFRINYADE----AQDVMHFNYTAWPDHGVPPANAAESILQFVFTVRQQA---AKSKGPMIIHCSAGVGRTGTF
IALDRLLQHIRD---HEFVDILGLVSEMRSY
PTPRO_Gg
PVQLDDFDGYIKDMAKDSDYKFSLQFEELKLIGLDIPHFAADLPMNRCKNRYTNILPYDFSRVRLVSMNEEEGSDYINAN
YIPGY-NSPQEYIATQGPLPETRNDFWKMVLQQKSQIIVMLTQCNEKRRVKCDHYWP-FTEDPI-AYGDITVEMLSEEEH
TDWVYRNFRISYADE----VQDVMHFNYTAWPDHGVPTANAAESILQFVQMVRQKS---VKSKGPMIIHCSAGVGRTGTF
IALDWLLQHIRD---HEFVDILGLVSDMRSY
PTPRO_Xt
PPQRFFKKKGGKEMSKDSDYKFSLQFEELKMVGLDIPHFAADLPSNRGKNRYTNILPYDCSRVKLISLEADEGADYINAN
YIPGY-SAAQEYIATQGPLPETRNDFWKMILQQKCQVIVMLTQCNEKRRIKCDHYWP-FTPEPV-NYGDITVEMASEEEQ
SDWAQRVFRVSYADE----TQCVTHFNFTAWPDHGVPTVNAAESVLQFVQVVRQKA---LKSKGPVTVHCSAGVGRTGTF
IALDWLMQHIRD---HEFVDILGLVSELRSH
PTPRO_Dr
PVQLSDFEAYLKDMGKDSAYKFSLQFEELKSVGLDLSHEAADLPINRPKNRYTNILPYDFSRVKLISLHNDEGSDYINAN
YIPGY-NSPREYIATQGPLPDTRNDFWKMVLQQKVHIIVMLTQCNERRRVKCDHYWP-FSDEPV-AYGEISVEMLAETDS
PEWTIRSFRLAYADE----TQDVLHFNYTSWPDHGVPTVNAIESILQFVQIVRQQV---NRSKGPIVVHCSAGVGRTGTF
ISLDRLMQHIQE---HEYVDVLGLVSDMRSH

[DIMENSIONS NCHAR=270;
FORMAT DATATYPE = Protein GAP = - MISSING = ?;
MATRIX]
capsasporaPTPc1
IVPAEELADCVAQLHANSDLGFAEEYEAL-NVNEEYATNAALMPANKSKNRYANILPYDHSRVRLSVIPGVEGSDYVNAN
YIDGY-RKSHAYIACQGPLPDTFDDFWRMVWEQRSAVIVMVTNEEEKGRVKCHRYWP--DDSEVVFGDVEVRMTRQEELS
EFITRVFSLKNIRT--GETRTVHHLQFTGWPDHGVP--HSTSSLIKFVKQAKAVQ---PNDAGPMVIHCSAGVGRTGTFI
VTDMSLERTKV---ENNVDIFGCVSALRRN
Sponge
-VYLRDFPSYVEDMLNDAGFKFSEEYEKVSAVGLDHSKDASLLPENRAKNRYTNILAYDHSRVKLESIDDEPGSDYINAN
YIPGY-RMRRAYIATQGPLPSTFDDFWRMTWEQNSHVIVMLTQLVERGRTKCHRYWP--GAQPEVYGEINVDMLSETEKS
DWIVRKFKITKEKR----SRTITHYQFVSWPDHGVP--DEAGPALDFVREVHEVA---SSAFGPVIVHCSAGVGRTGTFI
ALGTLLQHIKD---HDWVDLFGLASEMRQH
Acornworm
PVPLKGFRDYVNELDEDDRKGFEEEYDDLRKLVVRQPTSVGQRMEHRSKNRYTNIVPYDNNRVILS----GSGNSYINAS
YISGS-KGSRSYIATQGPLESTCGDFWKMIWQQRVTTVVMMTQCNELGKSKCHHYWPRDTQTQISHSDLTIRLTSETRLP
DWIIRDFSIESNGE----IRALRQFHFTSWPITGTP--YDADPLIRFIEAIRIQV---LPNSGPILVHCSAGVGRTGVFI
ALYHLLEYFYT---MIQVDIFGKVIKMRKQ
Seaurchin4
PTKKNMFAQHVAKMSARNNAGFSDEYNNLPAMEKTRAVTAARQARNDDKNRYRNILPYDYTRVQLKGV---AGTDYINAS
YIKDD-HGQKKYIATQGPLPNTIEHFWEMVWENQTSTIVMMTALVEGGKTKCEHYWP-AGEEPQLHGNVTVTLVGSNQTD
NFIERTLLLEKEGT----ERTVTHYQYLAWTDHGVP--ESTAPLVGLLRQVKVTNQEDGAATGPIVVHCSAGVGRTGTFI
AADMLMDAIQRSTATDYIDVAGTIAKIREQ
seaurchin3
PVQMRKFPDHVNRMSAGNNTGFVEEYNSLSNVGRDKSTDASRLAVNATKNRYRNILPFDETAVHLQDLEEGQTSDYINAN
YIQGL-HSMKEYIASQGPVPDSVNDFWEMVFENKSTTIAMITGLVEGGKTKCEHYWP-DDDTPVNYGSVTVTMTHTQEME
QWTVRSFLLEKGMQ----QFETHQYAFKGWVDHDVP--NNARPMIEFIRTIDVAH---DANLGPITVHCSAGIGRTGVFI
ALHKLIKQVETSKPNDYIDVCGTVARMREQ
seaurchin1
PIVLNKFEGHYGRMKADSDFRFTEEYDEIRLVGKDQAIVSALEMVNRAKNRFTNILPYEHSRVKLAALADDSDTDYINAN
YIPGY-NSPREFMACQGPLPGTVDDMWRMIWEKKTSIIVMLTQLVEKGKIKCHEYWP-ADYNPVTYGSIQVSVQALQQYD
HWVIREFSISQGDE----IRKLTQYHFMSWPDHGVP--DKTWTMLDFVRTVREAIQ-KTASDRPIVAHCSAGVGRTGTYI
ALDRLMQAMQE---NDYIDIFGIICEMRMQ
CElegans
PVRIEDFADHVRLMSADSDFRFSEEYDMMRNVGVGQSVAASELPINRPKNRFTNIPSYDHSRVKLSNPNNIEGGDYINAN
YVPGF-SSRREFIAAQGPLPTTRDHFWQMTWEQQCPAIIALTKCVEKGRDKCHQYWPDHENVPVLYGDIEVTIVAEKEFD
EFVIRDIRLEKSGPDGRVTRFVRHWHYMAWPDFGAP--SHPNGIIQFSRMFRHHLP-HSPHNAPTIVHCSAGVGRSGTFI
SIDRLLQSSSF---GDPIDVFGTVCEMRYE
Ciona
PVFRDEFPNHVSSLSQNKNKGFSLEFDDIRGIPYAGTTAIAENSCNKTKNRTTKLVPFDHCRVKIEGIPAIQGSNYINAS
YIPGL-DSPEQYIATQTPLDHTKKDFWRMLWETGSTNIVMLCGSVNAGKKRCDEFWP--KKQTEYFGNLAVQMKEEIRHD
EWIIRQFIVTMRDK----VRHVTQHHFIKWPQLEQA--ENSLPLVRFIKNYRLLR---DRSINPTIVMCSNGSGRCGVFI
GLLRILDNN-----GRDVDVFGTVAALRKY
Fly10D
PILIKNFAEHYRLMSADSDFRFSEEFEELKHVGRDQPCTFADLPCNRPKNRFTNILPYDHSRFKLQPVDDDEGSDYINAN
YVPGH-NSPREFIVTQGPLHSTRDDFWRMCWESNSRAIVMLTRCFEKGREKCDQYWP-NDTVPVFYGDIKVQILNDSHYA

DWVMTEFMLCRGSE----QRILRHFHFTTWPDFGVP--NPPQTLVRFVRAFRDRI---GAEQRPIVVHCSAGVGRSGTFI
TLDRILQQINT---SDYVDIFGIVYAMRKE
FlyDPTP4E
PVHVKDFSEHYRIMSADSDFRFSEEFEELKHVGRDQACSFANLPCNRPKNRFTNILPYDHSRFKLQPVDDDDGSDYINAN
YMPGH-NSPREFIVTQGPLHSTREEFWRMCWESNSRAIVMLTRCFEKGREKCDQYWP-VDRVAMFYGDIKVQLIIDTHYH
DWSISEFMVSRNCE----SRIMRHFHFTTWPDFGVP--EPPQSLVRFVRAFRDVI---GTDMRPIIVHCSAGVGRSGTFI
ALDRILQHIHK---SDYVDIFGIVFAMRKE
PTPRB_Hs
PIKINQFEGHFMKLQADSNYLLSKEYEELKDVGRNQSCDIALLPENRGKNRYNNILPYDATRVKLSNVDDDPCSDYINAS
YIPGN-NFRREYIVTQGPLPGTKDDFWKMVWEQNVHNIVMVTQCVEKGRVKCDHYWP-ADQDSLYYGDLILQMLSESVLP
EWTIREFKICGEEQ-LDAHRLIRHFHYTVWPDHGVP--ETTQSLIQFVRTVRDYIN-RSPGAGPTVVHCSAGVGRTGTFI
ALDRILQQLDS---KDSVDIYGAVHDLRLH
PTPRB_Mm
PIKINQFEGHFMKLQADSNYLLSKEYEDLKDVGRSQSCDIALLPENRGKNRYNNILPYDASRVKLCNVDDDPCSDYINAS
YIPGN-NFRREYIATQGPLPGTKDDFWKMAWEQNVHNIVMVTQCVEKGRVKCDHYWP-ADQDPLYYGDLILQMVSESVLP
EWTIREFKICSEEQ-LDAHRLIRHFHYTVWPDHGVP--ETTQSLIQFVRTVRDYIN-RSPGAGPTVVHCSAGVGRTGTFV
ALDRILQQLDS---KDSVDIYGAVHDLRLH
PTPRB_Gg
PIKVSHFEAHFTKLQADSNYLLSKEYEDLKDVGRNQTCDIALLPENRGKNRYNNILPYDTSRVKLSNVDDDPCSDYINAS
YIPGN-NFRREYIATQGPLPGTKDEFWKMAWEQNVHNIVMVTQCVEKGRVKCDHYWP-LDQDSLYYGDLIVEMLSESVLP
EWTIREFKICSEEQ-LDSTRLIRHFHYTVWPDHGVP--ETTQSLIQFVRTVRDYIN-RTPDTGPTVVHCSAGVGRTGTFI
ALDRILQQLDS---KDTVDIYAAVHDLRLH
PTPRB_Xt
PILTAQFEEHFSKLQTDSNYLLSREYENLKDFGRDQSSDTALLPENRGKNRYSNILPYDSTRVKLANVDDDPCSDYINAS
YMPGI-NFRREYIATQGPLPATKDDFWKMVWEQNVHIIVMVTQCTERGRAKCDHYWP-MDQDSYYYGDLIVQMLSESVLP
EWTIREFKICSEDQ-IDAPRLVRHFHYTVWPDHGVP--ETTQSLIQFVRTVRDYIN-RTPGSGPTVVHCSAGVGRTGTFI
VLDRMLQQVDT---VDSVDIFGAVRDLRIH
PTPRB_Dr
PVQAANFESHLAKLQSDSSYLLSEEFEGLKDVGRIQTQNAARLLGNRNKNRYNNILPYDSTRVRLSCLEDDPCSDYINAN
YIPGN-NFRWEYIATQGPLPGTKDDFWRMVWEQNVHSLVMVTQCVERGMVKCDHYWP-TDSEPLCYGDIVVQLLSEKVFP
EWTIRDFKISCESQ-LRYPRMVRQFHYTIWPDHGVP--DTTQSLIQFVRTVRDFIN-RTNSPGISVVHCSAGVGRTGTFI
VLDRALQQLDR---NCTVDIYGCVFDLRLH
PTPRJ_Hs
LIRVENFEAYFKKQQADSNCGFAEEYEDLKLVGISQPKYAAELAENRGKNRYNNVLPYDISRVKLS-VQTHSTDDYINAN
YMPGY-HSKKDFIATQGPLPNTLKDFWRMVWEKNVYAIIMLTKCVEQGRTKCEEYWP--SKQAQDYGDITVAMTSEIVLP
EWTIRDFTVKNIQT--SESHPLRQFHFTSWPDHGVP--DTTDLLINFRYLVRDYMK-QSPPESPILVHCSAGVGRTGTFI
AIDRLIYQIEN---ENTVDVYGIVYDLRMH
PTPRJ_Mm
LIRVENFEAYFKKQQADSNCGFAEEYEDLKLIGISLPKYTAEIAENRGKNRYNNVLPYDISRVKLS-VQTHSTDDYINAN
YMPGY-HSKKDFIATQGPLPNTLKDFWRMVWEKNVYAIVMLTKCVEQGRTKCEEYWP--SKQAQDYGDITVAMTSEVVLP
EWTIRDFVVKNMQN--SESHPLRQFHFTSWPDHGVP--DTTDLLINFRYLVRDYMK-QIPPESPILVHCSAGVGRTGTFI
AIDRLIYQIEN---ENTVDVYGIVYDLRMH
PTPRJ_Gg
MIKVENFESYFKKQQADSNCGFAEEYEELKSAGVHQPKFAAEIAENRGKNRYNNVLPYDISRVKLS-NPSCTTDDYINAN
YMPGY-SSKKAFIAAQGPLPNTIEDFWRMIWEKNIYSIVMLTKCVEQARTKCEQYWP--DKQSKSYGDIIVTMVSEVVLP
EWTIRDFNVENADT--MESHTVRQFHFTSWPDHGVP--ETTDLLINFRHLVHEYSS-QNPIDSPILVHCSAGVGRTGTFI
AIDRLIQQIEM---ENTVDVYGVVYDLRMH
PTPRQ_Hs
PISKKSFLQHVEELCTNNNLKFQEEFSELPKFLQDLSSTDADLPWNRAKNRFPNIKPYNNNRVKLIADASVPGSDYINAS
YISGY-LCPNEFIATQGPLPGTVGDFWRMVWETRAKTLVMLTQCFEKGRIRCHQYWPEDNKPVTVFGDIVITKLMEDVQI
DWTIRDLKIERHGD----CMTVRQCNFTAWPEHGVP--ENSAPLIHFVKLVRASR---AHDTTPMIVHCSAGVGRTGVFI
ALDHLTQHIND---HDFVDIYGLVAELRSE
PTPRQ_Mm
PVSKKSFLQHVEELCTNNNLKFQEEFSELPKFLQDLSSTDADLPWNRAKNRFPNIKPYNNNRVKLIADVSIPGSDYINAS
YVSGY-LCPNEFIATQGPLPGTVGDFWRMVWETRAKTLVMLTQCFEKGRIRCHQYWPEDNKPVTVFGDILITKLMEDIQI
DWTIRDLKIERHGD----CMTVRQCNFTGWPEHGVP--ENTTPLIHFVKLVRTSR---AHDATPMVVHCSAGVGRTGVFI
ALDHLTQHIHD---HDFVDIYGLVAELRSE
PTPRQ_Gg
PISKKSFLQHVEELCTNNNLKFQEEFSELPKFLEDLASTDADLPWNRSKNRFPNIKPYNNNRVKLMPDAGIPGSDYINAS
YVSGY-LCPNEFIATQGPLPGTVGDFWRMVWETRAKTLVMLTQCFEKGRIRCHQYWPEDNKPVTVFGDIVITKLVEDIQI
DWTIRDLKIERHGD----CMMVRQCNFTSWPEHGVP--ETTAPIIHFVKLIRASR---AHDNTPMVVHCSAGVGRTGVYI
ALDHLTQHIND---HDFVDIYGLVAELRSE
PTPRQ_Dr
PISKKSFLQHVEDLCANDNAKFQEEFAELPKLLQDLATSDADLPWNRSKNRFTNIKPYNNSRVKLLSEPGMPGSDYINAS
FVSGY-LCPNEFIATQGPLPSTVADFWRMIWETGTKTIVMLTQCFEKGRIRCHQYWPEDNKPVTVFADIIITKLTEDVRP
DWTVRALKVERHGS----YMIVHHFNYTSWPEHGVP--ESSSTLVQFVKAVRSNR---GHENTTIVVHCSAGVGRTGVFI
ALDHLIQHLRD---HEFVDIYGLVAELRSE
PTPRH_Hs

DIPAEDFADHVRKNERDSNCGFADKYQQLSLVGHSQSQMVASASENNAKNRYRNVLPYDWSRVPLKPIHEEPGSDYINAS
FMPGL-WSPQEFIATQGPLPQTVGDFWRLVWEQQSHTLVMLTNCMEAGRVKCEHYWP-LDSQPCTHGHLRVTLVGEEVME
NWTVRELLLLQVEE--QKTLSVRQFHYQAWPDHGVP--SSPDTLLAFWRMLRQWLD-QTMEGGPPIVHCSAGVGRTGTLI
ALDVLLRQLQS---EGLLGPFSFVRKMRES
PTPRH_Mm
DILAKDFADHVRENEKDSNCGFAEEYQQLALEGQGQSQITASALENRSKNRYRNVLPYDWSRVPLQPLQEEPGSDYINAS
FMPGL-WSPKEFIATQGPLPNTVGDFWRMVWEQQSHTLVMLTNCMESGRVKCEHYWP-LDAQPCIHGQLQVMLISEEASE
NWTVRHLQLFHMKE--QQTLSLRQFHYLAWPDHGVP--YSPDPLLAFRKMLRQWMD-QTTDGGPPIVHCSAGVGRTGTLI
ALDVLLRQLEC---EGLVGPFSFVKKMRES
PTPRH_Xt
SVMKSAFPDYYQRQHADSDFGFAEEYQQLSNVGINQSKLAAELSENRSKNRFTNVLPYDHSRVRLNRIDADETSDYINAN
YMPGY-NSSKEFIASQGPLPNTSADFWRMIWENQVSTIVMLTNCMENGRVKCEHYWP-LDYTPCTYGDITVTVTSEMILP
DWTVRDFTLKHAKQ--QGNKHARHFHFTVWPDHGVP--ENTTTIVEFRNLVREYMD-LKRSSGPTVVHCSAGVGRTGTLI
ALDYLIQKMEK---EQRIGIYSFVQKMRQN
PTPRH_Dr
TIACDKFPEHFRNMSRDDNRAFSAEYDDLSSVGVEQSKVAALLPENKDKNRFSNVLPYDTSRVHLT-INKAGDSDYINAN
YMPGYGNASREYIAAQGPLPSTVNDFWRMIWEKKSSTIVMVTNCTEGGRVKCEQYWP-LDYTPCLYENLLVTVKSENKSQ
SWTLREFNVKNKMT--SETRTVRHFHFTAWPDHGVP--RGTEELIQFRDLIRQHIE-SHFSTGPTVVHCSAGVGRTGTLI
ALDVLLQQLNR---EKAVGVAAFVQEMRLN
PTPRO_Hs
PVQLDDFDAYIKDMAKDSDYKFSLQFEELKLIGLDIPHFAADLPLNRCKNRYTNILPYDFSRVRLVSMNEEEGADYINAN
YIPGY-NSPQEYIATQGPLPETRNDFWKMVLQQKSQIIVMLTQCNEKRRVKCDHYWP-FTEEPIAYGDITVEMISEEEQD
DWACRHFRINYADE----MQDVMHFNYTAWPDHGVPTANAAESILQFVHMVRQQA---TKSKGPMIIHCSAGVGRTGTFI
ALDRLLQHIRD---HEFVDILGLVSEMRSY
PTPRO_Mm
PVQLDDFDSYIKDMAKDSDYKFSLQFEELKLIGLDIPHFAADLPLNRCKNRYTNILPYDFSRVRLVSMNEEEGADYINAN
YIPGY-NSPQEYIATQGPLPETRNDFWKMVLQQKSHIIVMLTQCNEKRRVKCDHYWP-FTEEPIAYGDITVEMVSEEEEE
DWASRHFRINYADE----AQDVMHFNYTAWPDHGVPPANAAESILQFVFTVRQQA---AKSKGPMIIHCSAGVGRTGTFI
ALDRLLQHIRD---HEFVDILGLVSEMRSY
PTPRO_Gg
PVQLDDFDGYIKDMAKDSDYKFSLQFEELKLIGLDIPHFAADLPMNRCKNRYTNILPYDFSRVRLVSMNEEEGSDYINAN
YIPGY-NSPQEYIATQGPLPETRNDFWKMVLQQKSQIIVMLTQCNEKRRVKCDHYWP-FTEDPIAYGDITVEMLSEEEHT
DWVYRNFRISYADE----VQDVMHFNYTAWPDHGVPTANAAESILQFVQMVRQKS---VKSKGPMIIHCSAGVGRTGTFI
ALDWLLQHIRD---HEFVDILGLVSDMRSY
PTPRO_Xt
PPQRFFKKKGGKEMSKDSDYKFSLQFEELKMVGLDIPHFAADLPSNRGKNRYTNILPYDCSRVKLISLEADEGADYINAN
YIPGY-SAAQEYIATQGPLPETRNDFWKMILQQKCQVIVMLTQCNEKRRIKCDHYWP-FTPEPVNYGDITVEMASEEEQS
DWAQRVFRVSYADE----TQCVTHFNFTAWPDHGVPTVNAAESVLQFVQVVRQKA---LKSKGPVTVHCSAGVGRTGTFI
ALDWLMQHIRD---HEFVDILGLVSELRSH
PTPRO_Dr
PVQLSDFEAYLKDMGKDSAYKFSLQFEELKSVGLDLSHEAADLPINRPKNRYTNILPYDFSRVKLISLHNDEGSDYINAN
YIPGY-NSPREYIATQGPLPDTRNDFWKMVLQQKVHIIVMLTQCNERRRVKCDHYWP-FSDEPVAYGEISVEMLAETDSP
EWTIRSFRLAYADE----TQDVLHFNYTSWPDHGVPTVNAIESILQFVQIVRQQV---NRSKGPIVVHCSAGVGRTGTFI
SLDRLMQHIQE---HEYVDVLGLVSDMRSH

[DIMENSIONS NCHAR=270;
FORMAT DATATYPE = Protein GAP = - MISSING = ?;
MATRIX]
capsasporaPTPc1
IVPAEELADCVAQLHANSDLGFAEEYEAL-NVNEEYATNAALMPANKSKNRYANILPYDHSRVRLSVIPGVEGSDYVNAN
YIDGY-RKSHAYIACQGPLPDTFDDFWRMVWEQRSAVIVMVTNEEEKGRVKCHRYWP--DDSEVVFGDVEVRMTRQEELS
EFITRVFSLKNIRT--GETRTVHHLQFTGWPDHGVP--HSTSSLIKFVKQAKAVQ---PNDAGPMVIHCSAGVGRTGTFI
VTDMSLERTKV---ENNVDIFGCVSALRRN
Sponge
-VYLRDFPSYVEDMLNDAGFKFSEEYEKVSAVGLDHSKDASLLPENRAKNRYTNILAYDHSRVKLESIDDEPGSDYINAN
YIPGY-RMRRAYIATQGPLPSTFDDFWRMTWEQNSHVIVMLTQLVERGRTKCHRYWP--GAQPEVYGEINVDMLSETEKS
DWIVRKFKITKEKR----SRTITHYQFVSWPDHGVP--DEAGPALDFVREVHEVA---SSAFGPVIVHCSAGVGRTGTFI
ALGTLLQHIKD---HDWVDLFGLASEMRQH
Acornworm
PVPLKGFRDYVNELDEDDRKGFEEEYDDLRKLVVRQPTSVGQRMEHRSKNRYTNIVPYDNNRVILS----GSGNSYINAS
YISGS-KGSRSYIATQGPLESTCGDFWKMIWQQRVTTVVMMTQCNELGKSKCHHYWPRDTQTQISHSDLTIRLTSETRLP
DWIIRDFSIESNGE----IRALRQFHFTSWPITGTP--YDADPLIRFIEAIRIQV---LPNSGPILVHCSAGVGRTGVFI
ALYHLLEYFYT---MIQVDIFGKVIKMRKQ
Seaurchin4
PTKKNMFAQHVAKMSARNNAGFSDEYNNLPAMEKTRAVTAARQARNDDKNRYRNILPYDYTRVQLK---GVAGTDYINAS
YIKDD-HGQKKYIATQGPLPNTIEHFWEMVWENQTSTIVMMTALVEGGKTKCEHYWP-AGEEPQLHGNVTVTLVGSNQTD
NFIERTLLLEKEGT----ERTVTHYQYLAWTDHGVP--ESTAPLVGLLRQVKVTNQEDGAATGPIVVHCSAGVGRTGTFI

AADMLMDAIQRSTATDYIDVAGTIAKIREQ
seaurchin3
PVQMRKFPDHVNRMSAGNNTGFVEEYNSLSNVGRDKSTDASRLAVNATKNRYRNILPFDETAVHLQDLEEGQTSDYINAN
YIQGL-HSMKEYIASQGPVPDSVNDFWEMVFENKSTTIAMITGLVEGGKTKCEHYWP-DDDTPVNYGSVTVTMTHTQEME
QWTVRSFLLEKGMQ----QFETHQYAFKGWVDHDVP--NNARPMIEFIRTIDVAH---DANLGPITVHCSAGIGRTGVFI
ALHKLIKQVETSKPNDYIDVCGTVARMREQ
seaurchin1
PIVLNKFEGHYGRMKADSDFRFTEEYDEIRLVGKDQAIVSALEMVNRAKNRFTNILPYEHSRVKLAALADDSDTDYINAN
YIPGY-NSPREFMACQGPLPGTVDDMWRMIWEKKTSIIVMLTQLVEKGKIKCHEYWP-ADYNPVTYGSIQVSVQALQQYD
HWVIREFSISQGDE----IRKLTQYHFMSWPDHGVP--DKTWTMLDFVRTVREAIQ-KTASDRPIVAHCSAGVGRTGTYI
ALDRLMQAMQE---NDYIDIFGIICEMRMQ
CElegans
PVRIEDFADHVRLMSADSDFRFSEEYDMMRNVGVGQSVAASELPINRPKNRFTNIPSYDHSRVKLSNPNNIEGGDYINAN
YVPGF-SSRREFIAAQGPLPTTRDHFWQMTWEQQCPAIIALTKCVEKGRDKCHQYWPDHENVPVLYGDIEVTIVAEKEFD
EFVIRDIRLEKSGPDGRVTRFVRHWHYMAWPDFGAP--SHPNGIIQFSRMFRHHLP-HSPHNAPTIVHCSAGVGRSGTFI
SIDRLLQSSSF---GDPIDVFGTVCEMRYE
Ciona
PVFRDEFPNHVSSLSQNKNKGFSLEFDDIRGIPYAGTTAIAENSCNKTKNRTTKLVPFDHCRVKIEGIPAIQGSNYINAS
YIPGL-DSPEQYIATQTPLDHTKKDFWRMLWETGSTNIVMLCGSVNAGKKRCDEFWP--KKQTEYFGNLAVQMKEEIRHD
EWIIRQFIVTMRDK----VRHVTQHHFIKWPQLEQA--ENSLPLVRFIKNYRLLR---DRSINPTIVMCSNGSGRCGVFI
GLLRILDNN-----GRDVDVFGTVAALRKY
Fly10D
PILIKNFAEHYRLMSADSDFRFSEEFEELKHVGRDQPCTFADLPCNRPKNRFTNILPYDHSRFKLQPVDDDEGSDYINAN
YVPGH-NSPREFIVTQGPLHSTRDDFWRMCWESNSRAIVMLTRCFEKGREKCDQYWP-NDTVPVFYGDIKVQILNDSHYA
DWVMTEFMLCRGSE----QRILRHFHFTTWPDFGVP--NPPQTLVRFVRAFRDRI---GAEQRPIVVHCSAGVGRSGTFI
TLDRILQQINT---SDYVDIFGIVYAMRKE
FlyDPTP4E
PVHVKDFSEHYRIMSADSDFRFSEEFEELKHVGRDQACSFANLPCNRPKNRFTNILPYDHSRFKLQPVDDDDGSDYINAN
YMPGH-NSPREFIVTQGPLHSTREEFWRMCWESNSRAIVMLTRCFEKGREKCDQYWP-VDRVAMFYGDIKVQLIIDTHYH
DWSISEFMVSRNCE----SRIMRHFHFTTWPDFGVP--EPPQSLVRFVRAFRDVI---GTDMRPIIVHCSAGVGRSGTFI
ALDRILQHIHK---SDYVDIFGIVFAMRKE
PTPRB_Hs
PIKINQFEGHFMKLQADSNYLLSKEYEELKDVGRNQSCDIALLPENRGKNRYNNILPYDATRVKLSNVDDDPCSDYINAS
YIPGN-NFRREYIVTQGPLPGTKDDFWKMVWEQNVHNIVMVTQCVEKGRVKCDHYWP-ADQDSLYYGDLILQMLSESVLP
EWTIREFKICGEEQ-LDAHRLIRHFHYTVWPDHGVP--ETTQSLIQFVRTVRDYIN-RSPGAGPTVVHCSAGVGRTGTFI
ALDRILQQLDS---KDSVDIYGAVHDLRLH
PTPRB_Mm
PIKINQFEGHFMKLQADSNYLLSKEYEDLKDVGRSQSCDIALLPENRGKNRYNNILPYDASRVKLCNVDDDPCSDYINAS
YIPGN-NFRREYIATQGPLPGTKDDFWKMAWEQNVHNIVMVTQCVEKGRVKCDHYWP-ADQDPLYYGDLILQMVSESVLP
EWTIREFKICSEEQ-LDAHRLIRHFHYTVWPDHGVP--ETTQSLIQFVRTVRDYIN-RSPGAGPTVVHCSAGVGRTGTFV
ALDRILQQLDS---KDSVDIYGAVHDLRLH
PTPRB_Gg
PIKVSHFEAHFTKLQADSNYLLSKEYEDLKDVGRNQTCDIALLPENRGKNRYNNILPYDTSRVKLSNVDDDPCSDYINAS
YIPGN-NFRREYIATQGPLPGTKDEFWKMAWEQNVHNIVMVTQCVEKGRVKCDHYWP-LDQDSLYYGDLIVEMLSESVLP
EWTIREFKICSEEQ-LDSTRLIRHFHYTVWPDHGVP--ETTQSLIQFVRTVRDYIN-RTPDTGPTVVHCSAGVGRTGTFI
ALDRILQQLDS---KDTVDIYAAVHDLRLH
PTPRB_Xt
PILTAQFEEHFSKLQTDSNYLLSREYENLKDFGRDQSSDTALLPENRGKNRYSNILPYDSTRVKLANVDDDPCSDYINAS
YMPGI-NFRREYIATQGPLPATKDDFWKMVWEQNVHIIVMVTQCTERGRAKCDHYWP-MDQDSYYYGDLIVQMLSESVLP
EWTIREFKICSEDQ-IDAPRLVRHFHYTVWPDHGVP--ETTQSLIQFVRTVRDYIN-RTPGSGPTVVHCSAGVGRTGTFI
VLDRMLQQVDT---VDSVDIFGAVRDLRIH
PTPRB_Dr
PVQAANFESHLAKLQSDSSYLLSEEFEGLKDVGRIQTQNAARLLGNRNKNRYNNILPYDSTRVRLSCLEDDPCSDYINAN
YIPGN-NFRWEYIATQGPLPGTKDDFWRMVWEQNVHSLVMVTQCVERGMVKCDHYWP-TDSEPLCYGDIVVQLLSEKVFP
EWTIRDFKISCESQ-LRYPRMVRQFHYTIWPDHGVP--DTTQSLIQFVRTVRDFIN-RTNSPGISVVHCSAGVGRTGTFI
VLDRALQQLDR---NCTVDIYGCVFDLRLH
PTPRJ_Hs
LIRVENFEAYFKKQQADSNCGFAEEYEDLKLVGISQPKYAAELAENRGKNRYNNVLPYDISRVKLS-VQTHSTDDYINAN
YMPGY-HSKKDFIATQGPLPNTLKDFWRMVWEKNVYAIIMLTKCVEQGRTKCEEYWP--SKQAQDYGDITVAMTSEIVLP
EWTIRDFTVKNIQT--SESHPLRQFHFTSWPDHGVP--DTTDLLINFRYLVRDYMK-QSPPESPILVHCSAGVGRTGTFI
AIDRLIYQIEN---ENTVDVYGIVYDLRMH
PTPRJ_Mm
LIRVENFEAYFKKQQADSNCGFAEEYEDLKLIGISLPKYTAEIAENRGKNRYNNVLPYDISRVKLS-VQTHSTDDYINAN
YMPGY-HSKKDFIATQGPLPNTLKDFWRMVWEKNVYAIVMLTKCVEQGRTKCEEYWP--SKQAQDYGDITVAMTSEVVLP
EWTIRDFVVKNMQN--SESHPLRQFHFTSWPDHGVP--DTTDLLINFRYLVRDYMK-QIPPESPILVHCSAGVGRTGTFI
AIDRLIYQIEN---ENTVDVYGIVYDLRMH
PTPRJ_Gg
MIKVENFESYFKKQQADSNCGFAEEYEELKSAGVHQPKFAAEIAENRGKNRYNNVLPYDISRVKLS-NPSCTTDDYINAN

YMPGY-SSKKAFIAAQGPLPNTIEDFWRMIWEKNIYSIVMLTKCVEQARTKCEQYWP--DKQSKSYGDIIVTMVSEVVLP
EWTIRDFNVENADT--MESHTVRQFHFTSWPDHGVP--ETTDLLINFRHLVHEYSS-QNPIDSPILVHCSAGVGRTGTFI
AIDRLIQQIEM---ENTVDVYGVVYDLRMH
PTPRQ_Hs
PISKKSFLQHVEELCTNNNLKFQEEFSELPKFLQDLSSTDADLPWNRAKNRFPNIKPYNNNRVKLIADASVPGSDYINAS
YISGY-LCPNEFIATQGPLPGTVGDFWRMVWETRAKTLVMLTQCFEKGRIRCHQYWPEDNKPVTVFGDIVITKLMEDVQI
DWTIRDLKIERHGD----CMTVRQCNFTAWPEHGVP--ENSAPLIHFVKLVRASR---AHDTTPMIVHCSAGVGRTGVFI
ALDHLTQHIND---HDFVDIYGLVAELRSE
PTPRQ_Mm
PVSKKSFLQHVEELCTNNNLKFQEEFSELPKFLQDLSSTDADLPWNRAKNRFPNIKPYNNNRVKLIADVSIPGSDYINAS
YVSGY-LCPNEFIATQGPLPGTVGDFWRMVWETRAKTLVMLTQCFEKGRIRCHQYWPEDNKPVTVFGDILITKLMEDIQI
DWTIRDLKIERHGD----CMTVRQCNFTGWPEHGVP--ENTTPLIHFVKLVRTSR---AHDATPMVVHCSAGVGRTGVFI
ALDHLTQHIHD---HDFVDIYGLVAELRSE
PTPRQ_Gg
PISKKSFLQHVEELCTNNNLKFQEEFSELPKFLEDLASTDADLPWNRSKNRFPNIKPYNNNRVKLMPDAGIPGSDYINAS
YVSGY-LCPNEFIATQGPLPGTVGDFWRMVWETRAKTLVMLTQCFEKGRIRCHQYWPEDNKPVTVFGDIVITKLVEDIQI
DWTIRDLKIERHGD----CMMVRQCNFTSWPEHGVP--ETTAPIIHFVKLIRASR---AHDNTPMVVHCSAGVGRTGVYI
ALDHLTQHIND---HDFVDIYGLVAELRSE
PTPRQ_Dr
PISKKSFLQHVEDLCANDNAKFQEEFAELPKLLQDLATSDADLPWNRSKNRFTNIKPYNNSRVKLLSEPGMPGSDYINAS
FVSGY-LCPNEFIATQGPLPSTVADFWRMIWETGTKTIVMLTQCFEKGRIRCHQYWPEDNKPVTVFADIIITKLTEDVRP
DWTVRALKVERHGS----YMIVHHFNYTSWPEHGVP--ESSSTLVQFVKAVRSNR---GHENTTIVVHCSAGVGRTGVFI
ALDHLIQHLRD---HEFVDIYGLVAELRSE
PTPRH_Hs
DIPAEDFADHVRKNERDSNCGFADKYQQLSLVGHSQSQMVASASENNAKNRYRNVLPYDWSRVPLKPIHEEPGSDYINAS
FMPGL-WSPQEFIATQGPLPQTVGDFWRLVWEQQSHTLVMLTNCMEAGRVKCEHYWP-LDSQPCTHGHLRVTLVGEEVME
NWTVRELLLLQVEE--QKTLSVRQFHYQAWPDHGVP--SSPDTLLAFWRMLRQWLD-QTMEGGPPIVHCSAGVGRTGTLI
ALDVLLRQLQS---EGLLGPFSFVRKMRES
PTPRH_Mm
DILAKDFADHVRENEKDSNCGFAEEYQQLALEGQGQSQITASALENRSKNRYRNVLPYDWSRVPLQPLQEEPGSDYINAS
FMPGL-WSPKEFIATQGPLPNTVGDFWRMVWEQQSHTLVMLTNCMESGRVKCEHYWP-LDAQPCIHGQLQVMLISEEASE
NWTVRHLQLFHMKE--QQTLSLRQFHYLAWPDHGVP--YSPDPLLAFRKMLRQWMD-QTTDGGPPIVHCSAGVGRTGTLI
ALDVLLRQLEC---EGLVGPFSFVKKMRES
PTPRH_Xt
SVMKSAFPDYYQRQHADSDFGFAEEYQQLSNVGINQSKLAAELSENRSKNRFTNVLPYDHSRVRLNRIDADETSDYINAN
YMPGY-NSSKEFIASQGPLPNTSADFWRMIWENQVSTIVMLTNCMENGRVKCEHYWP-LDYTPCTYGDITVTVTSEMILP
DWTVRDFTLKHAKQ--QGNKHARHFHFTVWPDHGVP--ENTTTIVEFRNLVREYMD-LKRSSGPTVVHCSAGVGRTGTLI
ALDYLIQKMEK---EQRIGIYSFVQKMRQN
PTPRH_Dr
TIACDKFPEHFRNMSRDDNRAFSAEYDDLSSVGVEQSKVAALLPENKDKNRFSNVLPYDTSRVHLT-INKAGDSDYINAN
YMPGYGNASREYIAAQGPLPSTVNDFWRMIWEKKSSTIVMVTNCTEGGRVKCEQYWP-LDYTPCLYENLLVTVKSENKSQ
SWTLREFNVKNKMT--SETRTVRHFHFTAWPDHGVP--RGTEELIQFRDLIRQHIE-SHFSTGPTVVHCSAGVGRTGTLI
ALDVLLQQLNR---EKAVGVAAFVQEMRLN
PTPRO_Hs
PVQLDDFDAYIKDMAKDSDYKFSLQFEELKLIGLDIPHFAADLPLNRCKNRYTNILPYDFSRVRLVSMNEEEGADYINAN
YIPGY-NSPQEYIATQGPLPETRNDFWKMVLQQKSQIIVMLTQCNEKRRVKCDHYWP-FTEEPIAYGDITVEMISEEEQD
DWACRHFRINYADE----MQDVMHFNYTAWPDHGVPTANAAESILQFVHMVRQQA---TKSKGPMIIHCSAGVGRTGTFI
ALDRLLQHIRD---HEFVDILGLVSEMRSY
PTPRO_Mm
PVQLDDFDSYIKDMAKDSDYKFSLQFEELKLIGLDIPHFAADLPLNRCKNRYTNILPYDFSRVRLVSMNEEEGADYINAN
YIPGY-NSPQEYIATQGPLPETRNDFWKMVLQQKSHIIVMLTQCNEKRRVKCDHYWP-FTEEPIAYGDITVEMVSEEEEE
DWASRHFRINYADE----AQDVMHFNYTAWPDHGVPPANAAESILQFVFTVRQQA---AKSKGPMIIHCSAGVGRTGTFI
ALDRLLQHIRD---HEFVDILGLVSEMRSY
PTPRO_Gg
PVQLDDFDGYIKDMAKDSDYKFSLQFEELKLIGLDIPHFAADLPMNRCKNRYTNILPYDFSRVRLVSMNEEEGSDYINAN
YIPGY-NSPQEYIATQGPLPETRNDFWKMVLQQKSQIIVMLTQCNEKRRVKCDHYWP-FTEDPIAYGDITVEMLSEEEHT
DWVYRNFRISYADE----VQDVMHFNYTAWPDHGVPTANAAESILQFVQMVRQKS---VKSKGPMIIHCSAGVGRTGTFI
ALDWLLQHIRD---HEFVDILGLVSDMRSY
PTPRO_Xt
PPQRFFKKKGGKEMSKDSDYKFSLQFEELKMVGLDIPHFAADLPSNRGKNRYTNILPYDCSRVKLISLEADEGADYINAN
YIPGY-SAAQEYIATQGPLPETRNDFWKMILQQKCQVIVMLTQCNEKRRIKCDHYWP-FTPEPVNYGDITVEMASEEEQS
DWAQRVFRVSYADE----TQCVTHFNFTAWPDHGVPTVNAAESVLQFVQVVRQKA---LKSKGPVTVHCSAGVGRTGTFI
ALDWLMQHIRD---HEFVDILGLVSELRSH
PTPRO_Dr
PVQLSDFEAYLKDMGKDSAYKFSLQFEELKSVGLDLSHEAADLPINRPKNRYTNILPYDFSRVKLISLHNDEGSDYINAN
YIPGY-NSPREYIATQGPLPDTRNDFWKMVLQQKVHIIVMLTQCNERRRVKCDHYWP-FSDEPVAYGEISVEMLAETDSP
EWTIRSFRLAYADE----TQDVLHFNYTSWPDHGVPTVNAIESILQFVQIVRQQV---NRSKGPIVVHCSAGVGRTGTFI
SLDRLMQHIQE---HEYVDVLGLVSDMRSH

```
;
end;

Begin assumptions;
Charset op1=1-275;
Charset op2=276-546;
Charset op4=547-817;
Charset op8=818-1087;
Charset op16=1088-1357;

end;
```
