# Supplementary material for: Phosphotyrosine phosphatase R3 receptors: Origin, evolution and structural diversification
Source: PLoS One. 2017 Mar 3;12(3):e0172887. doi: 10.1371/journal.pone.0172887 (PMC5336234; doi:10.1371/journal.pone.0172887)
Supplement: S3 File — (PDF) [file pone.0172887.s007.pdf]

```
#NEXUS

BEGIN data;
        DIMENSIONS Ntax=40 NCHAR=347;
        FORMAT DATATYPE = Protein GAP = - MISSING = ? interleave;
        MATRIX
upk3A_human
-----------------VNLQPQLASVTF-------ATNNPTLTTVALEKP--------LCMFDSKEALTGTHEVYLYVL
-----------------VDSAISRNASV-QDSTNTPLGSTFLQTE------------------------GGRTGPYKAV-
-AF-------DLIPCSDLPSLDAIGDVSKA--SQILNAYLVRVGAN-----GTCLWD------PNFQGLCNAPLSAATEY
RFKYVLVNM----------STGL---VEDQ-TLWSDPIRTNQLTPYSTIDTWPGRRSGGMIVITSILGSLPFFLLVGFAG
AIALSL---------------
upk3A_mouse
-----------------VNLQPQLASVTF-------ATNNPTLTTVALEKP--------LCMFDSSEPLSGSYEVYLYAM
-----------------VDSAMSRNVSV-QDSAGVPLSTTFRQTQ------------------------GGRSGPYKAA-
-AF-------DLTPCGDLPSLDAVGDVTQA--SEILNAYLVRVGNN-----GTCFWD------PNFQGLCNPPLTAATEY
RFKYVLVNM----------STGL---VQDQ-TLWSDPIWTNRPIPYSAIDTWPGRRSGGMIVITSILGSLPFFLLVGFAG
AIILSF---------------
Upk3A_chiken
-----------------QSMKPQLAAPEL-------ATNNPTLTTVALEKP--------FCMFDSSLHPNKSYAIYLYVM
-----------------KSSANTISSVV-TDSSSKPLDSTFQQTH------------------------GGHLGPYKAA-
-SF-------DVPNCVSPPRLADAGDINKV--SDVLKQYLFRVGDD-----GTCLYD------PNFLDVCNPPLAPDTTY
RFKYVLVDN----------TEGI---VKDQ-TLWSDPIKTRKAKLPMKIDIWPGRRSGSMIVITSILSVSVFLLLAGLLA
SVFSAL---------------
upk3A_frog
-----------------GADMPLLANSDF-------FSLNPTQTTIALEQP--------ICMFKSA--------VNVYLI
-----------------GIVAGAPNTPL-YDGNKKVNASTYSGTQ------------------------GGKTGPYIVA-
-KL-------PNQQCINIQALSNMADPTQV--QSILSKYVVRVGAD-----VTCLTN------PNFVGYCNAPLQGNTQY
SFKYLFTDS----------GD-I---VQSE-TSWSLGITTVNGKASSTIDTWPGRRSGGMIVLTSILSTLMFFVFIAYVI
GFAYSI---------------
UPK3A_fish
---------------EALKVKPEAVSPRL-------LRFNPTQSTVSLAKP--------LCVFDSVK-PTNEMMVDVYVV
-------------------HSLSATLT-------FETGKTYKETN------------------------GGTETPYKAT-
-SF-------GIPNCTSPPNPADLSVPQRI--DKTLDEYLVRIGSN-----PTCVGE------PEAEAFCNAPLSDGTSY
RFKYLLVNG----------TT-----TIAE-TEWSESILTRKALSPDEIDTWIGKRSGGGIVVTVILSLLLFLLLGAAIF
MGVLDV---------------
lamprey1
-----------------SGTKPQLVSSTA-------VPYNPTETTIVWSKP--------FCVFQKPV-PTTQYVVDVY--
-------------------ASITNNSYA-FDNSIGAVLSSYWTN-------------------------AVSPSPYLAA-
-TF-------KVPDCASQPSIYDA--------MAVKTNATFRLGGD-----TACVNSI-----GPSTSVCNGPLVPGMKY
RVKYTLSEE----------SPQFPRTIVDQ-TPWSDPVSTKKSPAASTINTWPGKRTGGMVVVTAVLSTLLFLLLAALLL
VVIFKA--------------C
lamprey2
-----------------SQVVPTVVNPN--------LLGAVTQTTVALQAPFCSALDAEVVALSSVSADLRLFVMATAQR
-------------------NVSNEMIT---SSSTIGLDKGYAGSG------------------------AGTSSWYLAL-
GGR-------PLQNCTISTP------------SLSTPTSYYRVGAD-----SKC----------SVAVTCNGPLNAGTIY
WFKYIMGTVAT--------NGAIDR-SYLE-SSWSKPIRLNKAGELNAIGVTPGPRSGGMVVVTVILVVLLFIAV-----
-------DRARPALG------
Upk3B_human
----------------MVPYTPQITAWD--------LEGKVTATTFSLEQP--------RCVFDGLASASDTVWLVVAFS
-------------------NASRGFQNP-ETLADIPASPQLLTDGH-----------------------------YMTLP
LSP-------DQLPCGDPM-------------AGSGGAPVLRVGHD-----HGC----------HQQPFCNAPLPGPGPY
RVKFLLMDT----------RGS----PRAE-TKWSDPITLHQGKTPGSIDTWPGRRSGSMIVITSILSSLAGLLLLAFLA
ASTMR----------------
Upk3B_mouse
----------------MIAYVPQITAWD--------LEGKITATTFSLEQP--------RCVFDEHVSTKDTIWLVVAFS
-------------------NASRDFQNP-QTAAKIPTFPQLLTDGH-----------------------------YMTLP
LSL-------DQLPCEDLT-------------GGSGGVPVLRVGND-----FGC----------YQRPYCNAPLPSQGPY
SVKFLVMDA----------AGP----PKAE-TKWSNPIYLHQGKNPNSIDTWPGRRSGCMIVITSILSALAGLLLLAFLA
ASTTR----------------
upk3B_chiken
----------------MLPYVPRVAPGA--------MPGKVTATTFVLERP--------RCIFDPFANASDAVWLAVAFA
-------------------DAPR-----------CPHDEGLPAARA-----------------------------YMTLQ
MAA-------AAYGC------------------SAPGAAVLRVGGD-----TAC----------HGRAPCNGPLPSPGPY
RVKFLLMGC----------GG-----PKAE-TKWSDPILLRRARSLSTIDPTPARRSSTAVVIAAILASLGAALAMAVLG
AV-------------------
Upk3B_frog
```

```
-----------------ITSYVPQLTLSP--------IVGTVTSTTFVLDKP--------QCVFGNTG---NQVWLLVARS
-------------------NVSANVVL-----TPPSMYSSFATK-------------------------GYYHVPFGTE-
----------SLYHC-------------------SNTAEYIRVGDT-----AQC----------NDNTNCNGPLPDPGPY
RVKYLVMNN----------NA-----LVSQ-SLWSQQITLLTGKSSSQLDTWPGRRSGGMIVLTSILSVLMGILTLCLFA
AFFVG----------------
PTPRB_human
-----------------RPPPPPPHIRV------NEKDVLISKSSINFTVN--------CSWFSDTNGAVKYFTVVVREA
-------------------DGSDELKP-----EQQHPLPSYLEYRH-----------------------NASIRVYQTN-
--Y-------FASKC--------AENPN-----SNSKSFNIKLGAEMESLGGKC--D------PTQQKFCDGPLKPHTAY
RISIRAFTQLFDEDLKEFTKP-----LYSD-TFFSLPITTESEPLF-------------GAIEGVSAGLFLIGMLVAVVA
LLIC-----------------
PTPRB_mouse
-----------------RPPQPPPHIRV------NEKDVLISKSSINFTVN--------CSWFSDTNGAVKYFAVVVREA
-------------------DSMDELKP-----EQQHPLPSYLEYRH-----------------------NASIRVYQTN-
--Y-------FASKC--------AESPD-----SSSKSFNIKLGAEMDSLGGKC--D------PSQQKFCDGPLKPHTAY
RISIRAFTQLFDEDLKEFTKP-----LYSD-TFFSMPITTESEPLF-------------GVIEGVSAGLFLIGMLVALVA
FF-------------------
PTPRB_chiken
-----------------RPPQPPPDIRV------NKKEVLITKSSINFTFN--------CSWFSDTNGAVKYFTVVVREA
-------------------DGSEGPKP-----DEQHPLPSYLEYKH-----------------------NDSIRIYQTN-
--Y-------FASRC--------AENPD-----SDYKSFDIKLGGEMENLGGKC--D------PDQQKFCDGPLKPRTAY
RISIRAFTQLFSEDPKELPQP-----LFAD-TFFSLPITTEAEPLF-------------GVIEGVSAGLFLIVMLVAVTA
LFV------------------
PTPRB_frog
-----------------RPPPPPLLIRV------NKKDTFISKSSIHFRFN--------CSWFSDTNGAVKYFTVIVSEA
-------------------DGNDNQRP-----EASLPLPSYADYKT-----------------------NKSTKIYQTS-
--Y-------FPSRC--------AENPD-----YNIQSYKIKLGTGMELLGGKC--D------QNENKYCDGPLSPRTSY
RISVRAFTQLFTEEMRTFPEP-----LYSD-TFFSLPITTEAGSLFFNKNITDLP----LQIFPQTKAMILV--------
---------------------
PTPRB_fish
-----------------RPPVPPVTVRV------NEHSAVITHFTIRFKFN--------CSWFSDANGAIRYFTIIATES
-------------------NDVDNGLP-----EQRHPLPSYLDYRQ-----------------------NHSIKAYQTG-
--Y-------FHSTC--------AEGSD-----GKVQVFEINLGAGMKHLGGAC--KLDPESIQHGSHLCDGPLRSRTSY
RLSVRAFTQLFDEENREFPHP-----LYTD-TYLSLPLLTQSAPRS-------------GLTGGITAALFLITMVLALTA
LLI------------------
PTPRQ_human
-----------------APARPKTKPTPIY---DATGKLLVTSTTITIRMP--------ICYYSDDHGPIKNVQVLVTET
-------------------GAQHDGNV-----------TKWYDAY------------------------FNKARPYFTN-
EGF-------PNPPC--------TEGKTK----FSGNEEIYIIGAD-----NACMIP------GNEDKICNGPLKPKKQY
LFKFRATNI----------MG-----QFTD-SDYSDPVKTLGEGLSERTV---------EIILSVTLCILSIILLGTAIF
A--------------------
PTPRQ_mouse
-----------------APARPKTKPIPIH---DATGKLLVTSTTITIRMP--------ICYYNDDHGPIRNVQVLVAEA
-------------------GAQQDGNV-----------TKWYDAY------------------------FNKARPYFTN-
EGF-------PNPPC--------IEGKTK----FSGNEEIYVIGAD-----NACMIP------GNEEKICNGPLKPKKQY
LFKFRATNV----------MG-----QFTD-SEYSDPIKTLGEGLSERTV---------EIILSVTLCILSIILLGTAIF
AF-------------------
PTPRQ_chiken
-----------------EPPRPKKKPAPVY---DTNGALLVTATTITIRMP--------VCYYSDDHGPIKKIQVLVVEA
-------------------GAQHDGNV-----------TKWHDAY------------------------FNRPRPYFTN-
EGF-------PNPPC--------IEGKED----LSGKEEIYVIGAD-----TTCMIS------GSQDKICNGPLKPRKQY
LFKFRATNV----------KG-----QFTD-SDYSDPVKTLGEGRSGGSV---------EVILAVTLCILSVVLLVAAVY
AF-----AR------------
PTPRQ_fish
-----------------APPKPKKTPRAAL---NSAGVIISTSKTITIEMP--------ECFFTDDHGPIQKVQVIVSEP
-------------------AVMDYGNL-----------SNWKSVF------------------------LHPTAPYLTD-
DGF-------LNPEC--------PKNSER----MSSSTKTYVIGED-----EGCL-S------EDAETLCNGPLKPKTHY
VFKFRATNI----------RG-----QFTD-SEYSDKVRTADDRLLTRDE---------QIILGVLLSFFLALFLILIIY
GS-------------------
CionaNocat_1
------------PTIISGAPGPILSPTQA-------LFGASPTHAFQIENP----CNRKSSLFSEIGGVITAIEVIVWQT
-------------------GAASSKWE-------AATPAIWADAIN-----------------------QNPIPPYVAG-
-----------TIQCS-----TASGRKKR---ALTPNGSGYVIGAE------TC--T------AANRVTCNGPLISGRQY
NVAYRGVNG----------LG-----TPSDMTASNGPFSTSTQRGLEA-----------GEIAAIVISSIVVLLLIICLV
YYCV---KR------------
CionaNocat_2
-----------APTLIAGAAPPTAGPTTN-------ALGVSPTVAFQVENP----CLRKAQLFSEIGGTINQIQLIVWQI
-------------------QAPTPTTN----------ANVWANVIN-----------------------QNPIVAYQAG-
```

-----------TITCTG----TASGRKKR---ALTANNDGYVVGAD-----STC--T------TTTVAVCNGPLPSGRQF
NVSYIGVNG----------GG-----QTSGMTAPGGPFSTSTPTGLEA-----------GEIAAIVISCIVVLLLIISLI
YYCV---KR------------
Sponge
-----------------PPTVPPNVTIGTPP--TGGTSDPTTATTIRIEVT--------IPEELNANGPLTRIRILIRIF
-------------------------------LSRNDTISTWYESQKFP----------------------NSVAPPWQATQ
LPLN-----------------QGNRRKRQ----AGGETVAETIGTN-----NSC---------GPNDIVCNGPLKPGTQY
QFKYRVYNS--------DDDD-----SYVE-SQYSGPIRTGNPIAEENNT-------GTTIVIAVVVVLLVIILLIAILV
IVVIIVLKRRKRKAYSFAA--
Nematode_2
----------------MAPPVPTVAPMI--------MKESVGSHNMIVRFP--------TTMFDNRNGEIKQFAIIVSET
-------------------TADESINR---WIESDNGTYTWQQVQR-----------------------FDVWPSYVAK-
--------------------LQDIQKVKQ----DVDVSIFEELGED-----ETCL-E------VRADRICNGPLRSASKY
RVRIRLFTS----------PT-----LFTD-SPPSQVMTTGSATPA-------------IPLLTVVAVLIVIAFVGIVGT
IFLFFW-NRTKKAR-------
annelid
--------------PIVMPNSPPPQAIT----------TAISHDKIRIILT---------NPFLNTNGDVVAFSVFVTTD
PNER------------F---------------MANSPLRTWADVKG-----------------------PSPMASYFAV-
--YKCANLFDGNDQCS-----SGPARKRRV--AQPRNTVEFTVGGD-----SSC--T------TNADDYCNGPLDAESTY
YVALVGYTE----------ND-----LYSS-GPSSEPIRTDTAPTNLL-----------LIIIVVVVVLVLAIVAAIGVI
VYM----RKRSSDNNE-----
ly_DPTP4e
---------------ILAPPVPEPSVTP--------LEVSRTSSTIEISFR--------QGYFSNAHGMVRSYTIIIAED
-------------------VGKNASGL---------EMPSWQDVQA-----------------------YTVWLPYQAI-
EPY--------NPFLT-------SNGSRK----SSLEAEHFTIGTA------NC--D------KHQAGYCNGPLRAGTTY
RIKIRAFTD----------ED-----KFTD-TVYSSPITTERSDT---------------VIVAATVSAVLLVAMVLVVV
YC-----QHRCQ---------
fly_PTP10D
---------------ILAPPRPATQVVP--------TEVYRSSSTIQIRFR--------KNYFSDQNGQVRMYTIIVAED
-------------------DAKNASGL---------EMPSWLDVQS-----------------------YSVWLPYQAI-
DPY--------YPF------------------ENRSVEDFTIGTE------NC--D------NHKIGYCNGPLKSGTTY
RVKVRAFTG----------AD-----KFTD-TAYSFPIQTDQDNT--------------SLIVAITVPLTIILVLLVTLL
FY-----KRRR----------
fly_Ptp52F
-----------------MPTIPSDELIKQMR--ANVEETSNPTKTAIVRLP--------ADIMTSASGDIKWMALMISQK
-------------------NCAGVPHL------KYDVSSDWPKVLSYQ-----EAGADGT---------GDCSLEYQTTE
ERW--------HPEP--------VQRQRRDGEVTSDEEIVFTIGLD------KC--S------EVQKTYCNGPLLPDTDY
NVVVRLFTA----------------------SGYSDAAVLNFKTKAAIKVT--------LILVSVCSCLLLAFVLGLTVL
WV-----RKRLA--------W
SeaUrchin1
E----------------PSPVPEDYPYE-----ANTVFSETSTTSFAVVLP--------DDLFSHENGELLTFAVIITID
---------------------DNDPTV------SSTELTYAARAEN--------------A------YITAIEIPYP---
--Y--------SPSF-------GSNRRRR----ATDPPGTVVIGDG------TC--A------GSQNEYCNGDLDDNTEY
YYAFRAYNG----------MG-----NVTS-STFG-PVKTVKDNTA-------------GIAAGVSVSLIIIIVAVVVVV
VFL----KRRQPKEPSPRPS-
SeaUrchin3
-----------------APPTPPADRVV--------RLVSHTGTTMSVSFD--------SSIFSDVYGPVTRYALLVAQS
-------------------SPDTIPST------PSDPAINWQ--------------------------VGSGQPYQTTL
LTY--------APFT------------------NGNTRGFYTVGAN-----NSC--T------QDSVGYCNGPVVPLTDY
RFALRAYGS----------DG-----KFTD-SAWSPLYNSGINTT--------------WYAAAVVSIIIITLVLLLVML
LI-----SRQC----------
SeaUrchin4
-----------------APPSPPRGATP---------SLVSRGSTITITFF---------NLFDTAFGRIVRFAIIVQER
-----------------VDGGTVVITA----KRQTTSSLTWAEARR------------------------TRPVPSYQTTP
DDY--------NPFA------------------DGAGVTTFRVGSQ------SC--D--P---DDLTAYCNGALYPGTSY
RFAIRAYGV----------DG-----KFVD-TEFSSPFRTDPDRGI-------------FVIPAIVLAIMVVIILIIFMA
MGFGC----------------
facornWorm
-----------------PPPKPTASRIT-----ANVRFVETTAYTLRIHLS--------NNLFDDRFGIIVAYLVIIAED
-------------------GGEYSPLP--------EVLPSYDEVID-----------------------SSPWPPYQTS-
QPF--------NPFET-----LSIGRRRR----NTLQTVNYIIGAK-----ENC---------DVSLVYCNGALRPVTDY
RYVIRGYNE----------LG-----NYTD-TDWSLPQKTDLDPF--------------WILYGVIAGLILIGIIILICI
CYCCCC-RRRRSSTQDSDK--
acorworm1
-----------------PPPPPRNNIVPS----SSESDIEVSQTTITVLFT--------DDYFNDSNGPLLNFTVIVTED
GTEEVDKHLNGEIPRVYCKDCENVPGI------EEGEVKTWAEVQN-------------------------TPARYQID-
YPY-------DYPDL---------SRRKR-----DTTGIEITIGSD-----DSC--S------TKDEGFCNGPLKDGTSY
RYHFRAYTD----------VG------YSD-TVLSGPIRTSTNMSWL------------WLLLGVLLGVLIAVAIILAII

```
YY-----KRRYYEETT-----
ciona
-----------------APPVPDTWPEVMNKIDDTSTMASVTSSTITVKKP---------CMFSNENGPIASLSVIIAED
-------------------GGNIEAEP-----------TYWSQAFP-----------------------LQPSPPYKVL-
VSEN------PTDYC--------NTRKRR-----SINEKGFVIGTS------DC--P------SELNTHCNGPLKSSTDY
RVKFRAETS----------NG-----LKTD-TEFSEIIRTSPSFLATHM----------TLLIGIGAAIGLFTILVVISA
VFLRLVMQYSIQYGFLNNV-F
Ciona2
-----------------APPVPALLPEVIIQQ----DKAIVTSSSVIVKKP---------CVFSDDNGPLKSLSVIVAQE
-------------------GATLDAEP-----------TYWAKAYS-----------------------EEPSPPYKVI-
VTEE------PQSYC--------NSRNKR----STQTNNGFVIGTS------DC--S------RLSTSQCNGPLKSNTEY
RFKYRAEAN----------NG-----LMTD-TEYSEVFRTNPSFIEAHM----------TLLVSIGAALGIFIILLTISV
ALLR-----------------
PTPRF_human
----------HLVSIRTAPDLLPHKPLP--------ASAYIEDGRFDLSMP--------H---VQDPSLVRWFYIVVVPI
-----------------------------DRVGGSMLTPRWSTPEELELDELLEAIEQGGEEQRRRRRQAERLKPYVAA-
--------------------------------QLDVLPETFTLGDK-----------------KNYRGFYNRPLSPDLSY
QCFVLASLKEP------MDQK-----RYAS-SPYSDEIVVQVTPAQQQEE--PEM----LWVTGPVLAVILIILIVIAIL
LF-----KR------------
PTPRF_mouse
----------HLVSIRTAPDLLPQKPLP--------ASAFIEDGRFSLSMP--------Q---VQDPSLVRWFYIVVVPI
-----------------------------DRVGGNLLAPRWNTPEELELDELLEAIEQGEEKQRRRRRQAERLKPYVAA-
--------------------------------QVDVLPDTFTLGDK-----------------KSYRGFYNRPLSPDLSY
QCFVLASLKEP------MDQK-----RYAS-SPYSDEIVVQVTPAQQQEE--PEM----LWVTGPVLAVILIILIVIAIL
LF-----KR------------
PTPRF_chiken
---------QHLVSIRTAPDVLQSKPIA--------TNKYIQEGKFTLTLP--------K---VQTTVPVRWYYIVVVPA
-----------------------------EQ-SPSSPTARWRTPDEMELDQLLEAISQGSQ-SRRQRRQADRLKPYIAA-
--------------------------------QVDVLPETFTLGDE-----------------KNYKGFYNKPLSQDLSY
RCFVLASLE-D------GDTK-----RYAA-SPYSDEIVMELASAKQQDE--PEM----LWVMGPVLAVILIIIIVIAIL
LF-----KR------------
PTPRF_fish
---------QQVVSIRTAPDLLKSKPVI--------YRGQEEDGKLTISLN--------RVCISDRSSCLKLIITCFFIT
------------------------------------ESYQWTDGVSQPYNKGLKSSKEGAC-----PRVKESRKPYSAPL
SSL-----------------------------------QLVTAGPE-----NTFP-SL---SRRNYLKFCNNKVKTNHFK
SLEILKKNL---PYFYTLFQK-----TFAA-SPYSDPITVEVVNRMPRQPEEPEM----LWVMGPVLAVILIIIIVIAIL
LF-----K-------------
PTPRF_monosiga
VILRGPGRVAERRTLSARPEAPPAVTSAVL------PDGYSSQSAFAVSWVAP----------STYSGPIRRLSLVVEPQ
-------------------GSSTHAVASLDCASSSCDFGTWSEAQA-----------------------ASSPMAYIAYS
QTF-------TESEG-----------------LGDLSRSSFVIGAER----EKV--D------ADGDGYVNGPLKAGATY
TFRLLTCTQ----------NGND---ELCQ-AASLNPTTALASTAAPAD--------NTGIIVGVVIVIIVLIVAVLVFV
MW-----RRRQNKAAAPDEDF
```

upk3A_human	GGGGGG	
upk3A_mouse		GGGGGG
Upk3A_chiken	GGGGGG	
upk3A_frog		GGGGGG
UPK3A_fish		GGGGGG
lamprey1		GGGGGG
lamprey2		GGGGGG
Upk3B_human		GGGGGG
Upk3B_mouse		GGGGGG
upk3B_chiken	GGGGGG	
Upk3B_frog		GGGGGG
PTPRB_human		GGGGGG
PTPRB_mouse		GGGGGG
PTPRB_chiken	GGGGGG	
PTPRB_frog		GGGGGG
PTPRB_fish		GGGGGG
PTPRQ_human		GGGGGG
PTPRQ_mouse		GGGGGG
PTPRQ_chiken	GGGGGG	
PTPRQ_fish		GGGGGG
CionaNocat_1	GGGTGG	
CionaNocat_2	GGGTGG	
Sponge		TGTTGG
Nematode_2		TGTTTG

annelid TGTTGG
ly_DPTP4e TGTTTT
fly_PTP10D TGTTTG
fly_Ptp52F TGTTGT
SeaUrchin1 TGGGGG
SeaUrchin3 TGGGGG
SeaUrchin4 TGGGGG
facornWorm TGGGGG
acorworm1 TGGGGG
ciona GGGGGT
Ciona2 GGGGGT
PTPRF_human TTTTTT
PTPRF_mouse TTTTTT
PTPRF_chiken TTTTTT
PTPRF_fish TTTTTT
PTPRF_monosiga TTTTTT

;

END;

[DNA sequence matrix follows; to use copy and paste from this file to a new file and remove brackets.
#NEXUS

BEGIN DATA;
DIMENSIONS NTAX=40 NCHAR=949;
FORMAT DATATYPE = Protein GAP = - MISSING = ? interleave;
MATRIX

upk3A_human
--gtgaacctgcagccccaactggccag
tgtgactttcgccac---------------caacaaccccacacttaccactgtggccttggaaaagcctct--------
------------------ctgcatgtttgacagcaaagaggccctcactggcacccacgaggtctacctgtatgtcctgg
tcgactcagccatttccaggaatgcctcagtgcaagacagcaccaacaccccactgggctcaacgttcctac--------
----------------------aaacagagggtgggaggacaggtccctacaaagctgtggcctttg-------------
---------------acctgatcccctgcagt-----------------------------gacctgcccagcctggatg
ccatt---------------------------ggggatgtgtccaaggcctcacagatcctgaatgcctacctggtcagg
gtgggtgccaacgggacctgcc------tgtgggatcccaacttccagggcctctgtaacgcacccctgtcggcagccac
ggagtacaggttcaagtatgtcctggtcaatatgtccacgggcttgg------------------------tagaggacc
aga---ccctgtggtcagaccccatccgcacca-accagctcaccccatactcgacgatcgacacgtggccaggccggcg
gagcggaggcat-gatcgtcatcacttccatcctgggctccctgcccttctttctacttgtgggttttgct-ggcgccat
tgccctcagcctc--
upk3A_mouse
--gtgaacctccagccccaactggccag
tgtgacctttgccac---------------caacaaccctaccctcaccaccgtggccttggagaagcctct--------
------------------gtgcatgtttgatagctcagagccactcagcggctcttacgaggtttacctctatgctatgg
tcgactcagccatgtccaggaatgtgtctgtacaggacagcgctggcgtcccactgagcaccactttccggc--------
----------------------aaacccagggtgggaggtcaggcccctataaagctgcggcctttg-------------
---------------acctgaccccttgtggt-----------------------------gacttgcccagcctggatg
ctgtt---------------------------ggagatgtgacccaggcctcagagatcctgaacgcatacctagtcagg
gtgggcaacaacgggacctgtt------tttgggaccccaacttccagggcctctgcaacccacccctgacagcggccac
tgagtacagattcaagtatgtcctggtcaacatgtccacaggcttgg------------------------tgcaggacc
aga---cactatggtcagatcccatctggacca-accggcccatcccctactcggccatcgacacgtggcccggccggcg
gagtggaggcat-gattgtcatcacgtccattctgggctccctgcccttcttcctgctcgtgggtttcgct-ggagccat
catcctcagcttt--
Upk3A_chiken
--cagagcatgaaacctcagcttgcagc
ccccgagcttgcaac---------------aaacaatcccactctcactacagttgccctagagaaaccttt--------
------------------ctgtatgtttgatagctcactacatccaaacaaatcttatgccatctacttgtatgtgatga
aaagttcagccaacacaataagctccgtggtgactgacagcagcagcaagccactggacagcacgttccagc--------
----------------------aaacacatgggggacatcttggaccttacaaggctgcctcattcg-------------
---------------atgtacccaactgtgtg-----------------------------tcacccccaggcttgctg
atgca---------------------------ggagacatcaacaaagtttccgatgtcctgaaacaatacctcttcaga
gttggggatgatgggacttgtt------tgtatgacccaaacttcctagatgtctgcaacccacctcttgcaccagacac
aacatacaggtttaaatacgtgttggtcgataacactgaaggtatcg------------------------tgaaagacc

```
aaa---ctctttggtctgatccaatcaaaacca-gaaaagctaaacttcccatgaaaattgatatctggcctggtcgaag
gagtggaagcat-gattgtcattacatcaattctaagtgtgtcagtgttccttctgcttgctggcttgctt-gcttctgt
gttttctgctctc--------------------------------------------------
upk3A_frog
------------------------------------------------gga------gcagacatgccgttgctggccaa
ctcggatttctttag---------------cctcaatcccacccagactacgattgcgctggagcagccgat--------
------------------ctgcatgtttaaatcggccg------------------------ttaacgtctatctaatcg
ggatcgtggcaggtgccccaaacaccccgctttatgacggcaataagaaggttaatgcctcaacctacagcg--------
----------------------gaacccaggggggcaagacgggaccctatatcgtggccaaacttc-------------
---------------ccaaccaacaatgtatc-----------------------------aacatacaggccctgagca
atatg---------------------------gccgaccccacgcaggtgcagtccatccttagcaaatacgtcgtgaga
gtgggggccgacgtgacctgct------taaccaatcccaactttgtggggtactgcaacgcccccctgcaggggaacac
tcaatacagtttcaagtatctgtttacagacagtggggatat---------------------------tgtgcagtccg
aga---ccagctggtccctgggcatcactaccg-tcaatggcaaagcttctagtaccatagacacctggccgggcaggag
gagtggtggcat-gattgtcctgacctccattctcagcactctgatgttcttcgttttcattgcctacgtc-attggctt
tgcttactccata--------------------------------------------------
UPK3A_fish
------------------------------------------------gaggctctgaaggtgaagcccgaggctgtgtc
tcccaggctgctgcg---------------gtttaacccgacacagagcacggtgtccctcgcgaagccgct--------
------------------ttgcgttttcgacagcgtgaagccaacgaatgaaat---gatggtagatgtatacgttgtcc
acagcctctcggccactctgacctttgagactgggaa------------------------aacgtacaagg--------
----------------------agacgaatggagggacagagactccttataaggctacatccttcg-------------
---------------ggatcccaaactgcacc-----------------------------tctccaccaaaccctgcag
acctg---------------------------tctgttccccagagaatcgataagactttagatgagtacttggtccgt
attggcagcaacccgacatgcg------tgggggaaccggaggctgaagccttttgtaatgcgccgctctccgatggcac
ttcgtacaggtttaaatatttgcttgtaaatgggactacaac------------------------------tatagcag
aaa---ccgaatggtcagagtcaatcctaacaa-gaaaggctctgagccctgatgaaattgacacctggattgggaagag
aagtggaggcgg-gatagttgtcactgtcatcctgagtctcctgctgtttctgctgcttggggctgccatt-ttcatggg
agtactggatgtg--------------------------------------------------
lamprey1
------------agc------------------------------------------ggcaccaagcctcaactcgtgag
ctcgacggccgttcc---------------gtacaacccgacggagacaacgatcgtgtggtccaagccctt--------
------------------ctgcgtgttccaaaagcccgttcccaccactc---------------agtacgtcgtggacg
tctacgcgtccatcaccaacaacagttatgcattcgacaactcaatcggcgcggtgctctcctcctact-----------
----------------------ggactaacgcggtgtcgcccagtccgtacctggcagccacattca-------------
---------------aagttccggactgtgca-----------------------------agtcaacccagcatttacg
acgccatggctgtca---------------------------------------------aaacgaacgcgacattccgt
ctgggcggcgacacggcctgcg---tgaacagcatagggcccagtacgtccgtatgcaacggaccgctcgtacccggaat
gaagtacagagtgaaatatacactttcggaagaatctcccca---------------atttcccagaactattgtggatc
aaa---ctccctggtcagaccctgtctccacaa-agaaatcgccagcggccagcaccatcaacacgtggcccggcaaacg
cacgggtgggat-ggtggtggttaccgccgtcctcagcacgctgctcttcctgctgctcgccgcactcctc-ctggtggt
catcttcaaggcg-----------------------------------------------tgc
lamprey2
------------------------------------------------------tcacaggtggtgcccaccgtggtgaa
ccccaacctgctggg------------------agccgtgacgcagacgacggtagcgctccaggcgccctt--------
------------------ctgcagcgccctggatgcggaggtggtggctctcag---ctcggtctccgccgacctgcggc
tcttcgtgatggccacggcgcaacgcaacgtgagtaacgagatgatcacgagctcgtcgacgatt---------------
---------------------------gggctcgacaagggatacgcgggctcgggtgcggggaccagcagctgg-----
--------tacttggctctgggaggccgcccc------------------------ctacaaaactgcaccatctccacg
ccgtctctgtcga-----------------------------------------------cgcccaccagctactaccgc
gtgggcgccgactccaagtgca------------------gcgtggcggtcacgtgcaacgggccactcaatgccggcac
catctactggttcaagtacatcatgggaacggtggctaccaacggggccat------------cgaccgctcttacctcg
agt---cttcctggtcgaaacccatccggctga-acaaagccggtgaactcaacgcgattggcgtgacccctgggccgcg
?tcgggcggcat-ggtggtggtgacagtcatcctggtcgtgctcctcttcatcgcggttgatcgggcccgg-cctgctct
tggt-----------------------------------------------------------
Upk3B_human
---------------------------------------------------ctggtgccctacacaccacagataacagc
ttgggacctggaagg------------------gaaggtcacagccaccaccttctccctggagcagccgcg--------
------------------ctgtgtcttcgatgggcttg------------------------ccagcgccagcgataccg
tctggctcgtggtggccttcagcaatgcctccaggggcttccagaacccggagacac-----------------------
-----------------------------tggctgacattccggcctccccacagctgctga------------------
---------------ccgatggccactacatg-----------------------------acgctgcccctgtctccgg
accagctgccctg------------------tggcgaccccatggcgggcagcg------gaggcgcccccgtgctgcgg
gtgggccatgaccacggctgcc------------------accagcagcccttctgcaacgcgcccctccctggccctgg
accctatcgggtgaagttcctcctgatggacaccaggggctc---------------------------acccagggctg
aga---ccaagtggtcagaccccatcactctcc-accaagggaagacccccggatccatcgacacctggccagggcggcg
aagtggcagcat-gatcgtcattacctccatcctctcttctctggccggcctcctactcttggccttcttg-gcagcctc
taccatgcgc-----------------------------------------------------
```

Upk3B_mouse
---ctgattgcctacgtgccgcagataacagc
ctgggacctggaagg------------------gaagatcacagccactacattctctctggagcagcctcg--------
------------------gtgcgtctttgatgagcatg------------------------tctcaactaaggacacca
tctggctagtggtggctttcagcaatgcctccagggactttcagaacccacagactg-----------------------
-----------------------------ctgctaagatcccgaccttcccacagctgctga------------------
---------------ctgacggccactatatg-----------------------------acattacccctgtccctgg
atcagctgccatg------------------tgaggacctgaccggtggcagtg------gaggtgtccccgtgcttcgg
gtgggcaatgattttggctgtt------------------accagcgaccctattgcaacgcccccctccccagccaggg
cccttacagtgtgaagttccttgtaatggatgccgccggccc---------------------------acccaaggctg
aga---cgaagtggtccaaccccatttatctcc-accaaggaaagaatcccaactccattgacacatggcctggccgacg
gagcggctgtat-gatcgtcataacttccatcctctctgccctggccggcctcttgctcctggctttcctg-gcagcttc
cactacgcgt---
upk3B_chiken
---ctgctgccctacgtgccccgcgtggcccc
cggtgccatgccggg------------------gaaggtgacggccaccaccttcgtgctggagagaccccg--------
------------------ctgcatcttcgacccctttg------------------------ccaacgcctccgatgccg
tctggctggcggtggcttttgccgacgcgccgaggtgcccccatgacgaggggctgc-----------------------
---ctgccgcccgcgc------------
---------------ctacatgacactgcaga-----------------------------tggcggcagccgcctacgg
atgctcagctc---ccggtgcagccgtgctgcgg
gtcggcggggacacggcgtgcc------------------acggccgggcaccctgcaatgggccgctgccctccccggg
gccctacagggtgaagttcctgctgatgggctgcggtggccc------------------------------caaagcgg
aga---cgaagtggtccgaccccatcctcctgc-ggagagctcgcagcctgagcaccatcgaccccacacccgcacgtcg
cagcagcaccgc-ggtcgtcatcgccgccatcctggccagcctgggtgccgcgctggccatggccgtgctg-ggagctgt
g--
Upk3B_frog
---atcacttcatatgttccacaattgaccct
ctcgcctatcgtggg------------------cacagtaacaagtacaacatttgtactagacaaaccaca--------
------------------atgcgtatttggtaacacag---------------------------------ggaatcagg
tttggttgcttgttgctagaagtaacgtgtcagcgaatg---
-----------------------------tggtgctcactccaccctccatgtattcatcatttgccacta---------
---------------aaggatactaccatgtt----------------------ccctttggcactgaatctttatacca
ctgct---ctaacacagctgaatatatc
cgagttggagatactgcgcagt---------------gtaatgataatacaaactgcaatggtcccttacctgatcctgg
gccttaccgggtgaaatatcttgtgatgaacaataatgctct------------------------------agtctcac
aat---cactttggtctcaacaaataactttgc-tgacagggaaaagttcatctcaactcgatacttggcctgggaggag
aagtggaggaat-gattgttctaaccagcattctatcagttcttatgggaattttgacactttgcctattt-gctgcttt
ctttgttgga---
PTPRB_human
---cgcccccctcctccacccccacacattcg
tgtgaatgaaaagga---------------tgtgctaattagcaagtcttccatcaactttactgtcaactg--------
------------------cagctggttcagcgacacca---------------------------atggagctgtgaaat
acttcacagtggtggtgagagaggctgatggcagtgatgagctgaagccagaacagcagcaccctctcc-----------
---cttcctacctggagtacaggcacaatgc---------
---------------ctccattcgggtgtatc-----------------------------agactaattattttgccag
caaatgtgccgaaaat----------cctaacagcaactccaagagttttaacattaagcttggagcagagatggagagc
ctaggtggaa------------------aatgcgatcccactcagcaaaaattctgtgatggaccactgaagccacacac
tgcctacagaatcagcattcgagcttttacacagctctttgatgaggacctgaaggaattcacaaagccactctattcag
aca---catttttttctttacccatcactactg----------------------aatcagagcccttgtttgg------
-------------agctattgaaggtgtgagtgctggtctgtttttaattggcatgctagtggctgttgtt---------
------------------------------------gccttattgatctgc------------
PTPRB_mouse
---cgcccgcctcaaccgcctccacacatccg
tgtgaatgaaaagga---------------tgtgctaatcagcaaatcttccatcaactttactgtcaactg--------
------------------cagctggttcagcgacacca---------------------------acggagcggttaaat
actttgctgtggtggtgagagaggccgacagcatggatgagttgaagccagaacagcagcaccctctcc-----------
---cttcctacctggagtacagacacaacgc---------
---------------ctccatccgagtctacc-----------------------------agaccaattattttgccag
caaatgtgctgaaagt----------cccgacagcagttctaaaagtttcaacattaagcttggagcagagatggacagc
ctcggtggca------------------aatgtgatcccagtcagcagaaattctgtgatggaccgctgaagccacacac
cgcctacagaatcagcatccgggcttttacacagctatttgacgaggacttgaaagagttcaccaaacctctctactcgg
ata---cgttcttctctatgcccatcaccacag----------------------agtcagagcccttgtttgg------
-------------agttattgaaggtgtgagtgctggcctgtttctaattggcatgctggtggcccttgtt---------
------------------------------------gccttcttc------------------
PTPRB_chiken
---cgtccccctcagccacctccagacatacg
agtaaacaaaaaaga---------------ggtgctgatcaccaaatcctccattaactttacttttaactg--------

------------------cagctggtttagtgatacta---------------------------atggagctgtgaagt
acttcactgtggttgtcagggaggctgatggtagtgaaggaccaaagcctgatgagcaacacccgttac-----------
---cttcctacctggagtacaaacacaatga---------
---------------ctccatacgcatctacc-----------------------------agacaaattatttcgccag
tagatgtgctgaaaac----------cctgacagtgactataaaagctttgacattaagcttggaggagaaatggaaaat
ctgggaggaa------------------agtgtgatccggatcagcaaaaattctgcgatggacccctgaagcctcggac
tgcttataggatcagcatacgggcttttacccagctcttcagtgaagacccaaaggaacttcctcaaccgctctttgcag
aca---ccttcttctccttaccaatcactacag----------------------aggcagagcctctctttgg------
-------------agttattgaaggtgtgagtgctggtttgtttctgatcgtgatgttagtggctgttact---------
------------------------------------gctttatttgtc---------------
PTPRB_frog
---cgccctcctccgccacctcttttaatcag
agtgaataaaaaaga---------------tacattcattagcaagtcatcaatccacttcagatttaattg--------
------------------cagttggttcagtgacacca---------------------------atggagctgtcaaat
atttcactgtgattgtttcggaagcagatggcaatgacaaccagaggcctgaggcaagccttcctttgc-----------
---catcatatgcagattataaaacaaataa---------
---------------gtccactaaaatttacc-----------------------------agactagctactttccaag
tcgatgtgcagagaac----------cctgactacaatatccagagttacaaaattaagctgggtacaggaatggaactt
ttgggtggca------------------aatgtgatcagaatgaaaataaatactgtgatggaccactgagtccaaggac
atcctacaggataagtgtcagagcttttacccaactatttactgaagaaatgaggacattccctgagccactgtatagtg
aca---ctttcttctccttgccaatcacaacgg----------------------aagcaggttctttattt--------
--------------tttaacaaaaacataacagatttgcctttacagatttcccacagacaaaagccatg---------
------------------------------------atcctggta------------------
PTPRB_fish
---aggccccctgtcccaccagtgaccgtacg
ggtcaatgagcattc---------------agcggtcatcacccatttcaccatccggtttaagttcaactg--------
------------------cagctggttcagtgatgcta---------------------------acggagccattcgct
acttcacaataattgccacggagtcaaatgatgttgataatggcttgccggaacagagacatcctctgc-----------
---catcttatctggactatagacagaacca---------
---------------ctccatcaaagcctacc-----------------------------agacgggctactttcacag
cacgtgtgccgagggg----------tccgacggcaaggttcaggttttcgagataaacctgggagcaggaatgaaacat
ttgggcggagcttgcaaattggatccagaatctatccagcacggatcacatctttgtgacgggccccttagatccaggac
atcgtatcgattaagcgtccgtgccttcactcaactgtttgatgaagagaacagagaatttccacacccgctttatacag
aca---cctatctctcgctccctttattaactc----------------------aatcagcaccacgaagcgg------
-------------cctgacaggagggatcacggctgccctgtttctcatcactatggtgctcgccctgaca---------
------------------------------------gccttactgatt---------------
PTPRQ_human
--gctccagcacgaccaaaaaccaaaccaacccctattta
tgatgccacaggaaa---------------actgcttgtgacttcaacaacaattacaatcagaatgccaat--------
------------------atgttactacagtgatgatc---------------------------atggaccaataaaaa
atgtacaagtgcttgtgacagaaacaggagctcagcatgatggaaatgtaa-----------------------------
---caaagtggtatgatgcatattttaataa---------
---------------agcaaggccatatttta-----------------------------caaatgaaggctttcctaa
ccctccatgtaca-------------------gaaggaaagacaaagtttagtg------gcaatgaagaaatctacatc
ataggtgctgataatgcatgca------tgattcctggcaatgaagacaaaatttgcaatggaccactgaaaccaaaaaa
gcaatacttatttaaatttagagctacaaatattatgggaca------------------------------atttactg
act---ctgattattctgaccctgttaagactt----------------------taggggaaggactttcagaaagaac
cgtaga-------gatcattctttccgtcactttgtgtatcctttcaataattctccttggaacagctatt-tttgca--
----
PTPRQ_mouse
--gctccagcacgaccaaaaaccaagccaattcctattca
tgatgccacaggaaa---------------actgcttgtgacgtcaacaacaattacaatcagaatgccaat--------
------------------ctgctactacaatgatgacc---------------------------acgggccaatcagaa
atgtgcaagtccttgtggcagaagcaggagctcagcaagacggaaatgtga-----------------------------
---caaagtggtatgatgcatattttaacaa---------
---------------agcaaggccatatttta-----------------------------caaacgaaggattccctaa
tcccccgtgtata-------------------gagggaaagactaagttcagcg------gtaatgaagaaatctatgtg
ataggtgctgataatgcctgca------tgatccctggaaatgaggagaaaatttgcaatggacctctgaaacccaaaaa
gcagtatttgtttaaatttagagccacaaatgtcatgggaca------------------------------atttactg
act---ctgagtactctgaccctattaaaactt----------------------taggtgaaggactttcagaaagaac
cgtgga-------gatcatactgtcagtcactttgtgtatcctctcaatcatcctccttggaacagctatt-tttgcatt
t--
PTPRQ_chiken
--gaaccaccacggcctaaaaagaaaccagcacctgttta
tgataccaatggggc---------------cttacttgtcacagcaacaacaattacaatcagaatgccagt--------
------------------atgttattacagcgatgatc---------------------------atggacctatcaaga
agatacaagttcttgttgtggaagctggagctcagcatgatggaaacgtta-----------------------------
---ctaagtggcatgatgcatacttcaacag---------

---------------accaaggccatattta------------------------------caaatgaaggctttccaaa
cccaccatgtata-------------------gaaggaaaggaagatctgagtg------gtaaagaagaaatatatgtt
ataggtgctgatactacatgta------tgatatcaggcagtcaagacaaaatatgtaatggcccacttaaaccaagaaa
gcagtacctattcaagttcagagcaactaatgttaaaggaca------------------------------gtttacag
att---ctgactattctgaccctgtcaaaactt----------------------taggtgaagggcggtcaggaggatc
tgtgga-------agttatccttgcagttactttgtgtatactttcagttgttcttctggtggctgcggtc-tatgcttt
tgcaa--ga
PTPRQ_fish
--gctcctcccaaaccgaagaagacgccgcgggcagctct
gaacagtgctggtgt---------------catcatctcaacctccaagaccatcaccatcgaaatgcctga--------
------------------atgtttcttcactgatgacc---------------------------atggaccaattcaga
aagtccaggtcatagtttctgaacccgcagtgatggactacggtaatctat-----------------------------
---ccaactggaagagtgttttcctccaccc---------
---------------cactgctccatacctga-----------------------------cagacgatggcttcctgaa
cccagagtgccct-------------------aaaaattctgagcgcatgagct------caagcactaagacgtatgtt
ataggagaagacgagggctgcc------tg---tctgaagatgcagagacgctttgtaacggacctctgaaacccaaaac
acattatgtgttcaaatttcgagcgacaaacatacgtggcca------------------------------gttcacag
act---ctgagtactcagacaaagtcagaactg----------------------cagatgaccgcttgctgaccagaga
tgagca-------gatcatccttggagtcttgctgtcattttttctggcgttgtttttaattctgattata-tatggatc
t--
CionaNocat_1
--ccaactattatatctggtgcacccggcccaatacttag
cccaacccaagcactctt------------tggggcttccccaacccatgctttccaaatagagaacccttgtaac----
----------agaaagtcgagtttgttcagtgagattg---------------------------gtggagtgataaccg
cgatagaagtcatcgtgtggcagactggagctgcaagcagta--------------------------------------
----------------------agtgggaggctgccacacctgcaatatgggctgatgccatcaaccaaaa---------
---------------cccaattcctccatacg-----------------------------ttgctggcactatacaatg
ttcta--ctgcatct--------------ggtcgtaagaagagagctttaacac------ccaacggatctggatatgtg
attggtgcggaaa---------------cttgcactgcggcaaacagagttacatgcaatggaccacttatatctggtcg
acaatataatgttgcttatcgtggggttaacggtctcggcac------------------------------accctcag
atatgactgcctccaacggccccttttcaacaa----------------------------gtacgcaacgaggtttgga
ggctgggga----aattgcagcgattgttatctcaagcattgtggtgttacttcttataatttgccttgtc---------
-------------------------------------tactactgtgtgaaa--------cgg
CionaNocat_2
---------------------------------------gctcctacattaattgctggagctgccccccctactgctgg
ccccacaacaaacgctct------------tggagtttcacccacagttgccttccaagttgaaaacccatgcctc----
----------agaaaagcgcagttattttcagaaatag---------------------------gcggcactattaatc
aaatccaacttattgtttggcaaattcaagcacctact--
---------------------------ccaacaactaacgccaacgtttgggcaaatgttatcaatcagaa---------
---------------tcctattgttgcatacc-----------------------------aagctggcactataacatg
cactggaacagcgagt-------------ggtagaaagaagagagctttaactg------ctaacaatgatggctatgtt
gttggtgctgattcca------------catgtacaacaacaactgtagccgtttgtaatggcccgcttcctagtggtcg
tcagttcaatgtcagctacataggagttaatggtggtggtca------------------------------aacctcag
ggatgactgcaccaggtggtcccttttcaacaa----------------------------gcaccccaactggattgga
agctggcga----aattgctgctattgtcatttcttgcattgtagttttgttgctaatcatatcacttatt---------
-------------------------------------tactactgcgtgaaa--------agg
Sponge
---cctccaacagtaccacctaatgttactat
aggaacaccccctac---
------------------tggtggcaccagtgatcccacaa------------------------ctgccacaacgataa
gaatagaagtcacaataccagaggaacttaatgctaatggtccactgacgaggattcgtatattgatacgtattt-----
-------------------ttctatctcgaaatgatacaatatccacctggtatgagagtcaaaaattc-----------
---------------ccaaattctgtggcacc--------------------------accatggcaggcgactcaacta
ccattaaatcaagga--------------aataggaggaagagacaggctggcg------gtgagactgtagctgagacc
ataggtactaaca---------------actcgtgtggtcctaatgacatcgtttgtaacggcccattaaaaccagggac
ccagtaccagttcaagtacagggtgtataatagtgatgatgatga------------------------ttcctatgtgg
aga---gtcagtactctggccccattagaacag---gtaatccaatagctgaggaaaataatactggtacaac------
-------------aatagtgattgcagtggttgtggttctattggtcattatattattgattgctattctt-gttattgt
tgttattattgtccttaagagaaggaaaagaaaagcatactcttttgctgca-----------
Nematode_2
---------------------------------------atggcaccaccagtgcctacagtagctccaatgattatgaa
ggaaagtgttggaagtca------------------------------taatatgattgtgagatttccaac--------
------------------aacaatgttcgataatagaa---------------------------acggagaaattaaac
aatttgctataattgtctcggaaaccacagctgacgaatctataaatagatggatagagagc------------------
------------------------------gacaacggaacttatacatggcaacaagtacaacgatttga---------
---------------tgtttggccatcttacgtggcaaaactt-------------------------------------
--------------------------------caagatattcagaaagtgaaac------aagatgtagatgtgtcgatt
ttcgaagaattaggggaggatgagacttgtctagaagtgagagccgatcgtatatgtaacggaccacttcgttccgcatc

```
gaaatatcgtgtcagaatccgcttgttcacgtctccgacttt------------------------------gttcacag
att---cacctcccagccaagtgatgaccaccg----------------------------gctcagcaactcc------
-agcaatcccattgctaacagttgtcgctgttctgattgtgatcgcattcgtcgggatcgtcggaaccatattcctcttc
ttctggaatcgaacaaagaaagcgaga------------------------------------
annelid
------------------------------------------cccattgtaatgcccaactcgccaccaccacaagctat
tacaactgcgatttc---------------acacgacaaaatccgaatcattctgac-----------------------
------------------aaatccgttttgaatacta----------------------------atggggatgtcgtcg
cgttttcggttttgtgaccaccgatccgaatgaacgttttatggctaatagtccgc------------------------
----------------------------------------tacgaacatgggcagacgtcaagggtccatc---------
---------------tccgatggcttcgtatttcgctgtctata-----------------aatgcgccaacttatttga
tggcaatgatcagtgctcaagcggcccggcgaggaaacgccgcgtagctcaaccaa----gaaacaccgttgagttcact
gtgggtggcgactcaa------------gctgcacaacaaatgctgatgactactgcaatggacccttggatgctgagtc
tacgtactatgttgcacttgttggttataccgagaatgatct------------------------------atattcaa
gtg---gtccctcttccgaacccattcgcactg----------------------------acactgcgcctac------
-gaacttgttgctgattataattgtggtcgtggttgttctggtgttggccatcgtggctgccattggtgtc--attgttt
acatgagaaagcgatcttcagacaataatgag-------------------------------
ly_DPTP4e
---------------------------------------------------atactagcacctccggttccggagccaag
tgtaacaccactgga---------------agtgagcaggaccagtagcaccatcgagattagtttccgtca--------
------------------gggttacttctccaacgctc---------------------------atggcatggtaagat
cctatacgataatcatagccgaggatgtgggcaaaaatgcctccggactggagatgc-----------------------
-------------------------------------------ccagctggcaggatgtgcaggcatatac---------
---------------cgtgtggctgccttatc-----------------------------aagccatagagccatacaa
tccattcctgaccagc----------------aatggcagcagaaagagcagcc------tggaggcagagcattttacg
ataggaacggcga---------------actgcgataaacatcaggcgggctactgcaatggtccgctgcgggctggaac
cacctataggattaagattcgtgcctttacggacgaggacaa------------------------------gttcacgg
aca---cggtgtacagttcaccgataaccaccg----------------------aacgcagtgataccgtcat------
-------------agtggcggctaccgtttcggctgtgttactggtggcaatggtgcttgtggtgg------tgtactgt
cagcaccgctgccaa------------------------------------------------
fly_PTP10D
---------------------------------------------------atattagcgccaccacgtcctgccaccca
agtggtgcccaccga---------------ggtctatcgcagctcatcgaccatccagattcggtttaggaa--------
------------------gaactacttctcggatcaaa---------------------------acggccaggtgcgca
tgtacacgatcatcgtggccgaggatgatgccaagaatgcatccggcctggagatgc-----------------------
-------------------------------------------ccagctggctggatgtgcagtcgtacag---------
---------------cgtttggttgccctatc-----------------------------aggccatagatccgtacta
tccattcgagaat----------------------------------------------cgatccgtagaggacttcacc
atcggtacggaga---------------actgtgacaaccacaagatcggctactgcaacggaccactgaaatcgggaac
cacctatcgggttaaggtgcgggcgttcaccggagcggataa------------------------------gttcacgg
ata---ccgcctacagttttcccattcagacag----------------------atcaagacaacacctc---------
-------------actgattgtggccattacggtgccgttaactatcatcttggtgctcctggtgacacttttgttctac
aaacgacgtcgc---------------------------------------------------
fly_Ptp52F
---------------------------------------ctgcccaccattccgagtgatgagcttatcaagcaaatgcg
cgccaatgtcgaggagac------------atctaatccaacgaaaacggccattgt-----------------------
------------------tcgccttccagccgacatcatgacatccgcat---------------ccggcgacatcaagt
ggatggcactgatgatctcgcaaaagaactgtgctggagttccacacctcaaatacgat---------------------
------------------gtcagcagcgattggccaaaggttctatcctatcaagaggccggcgcagatgg---------
------cacaggtgactgcagtctggagtacc-------------------aaaccaccgaggagcgctggcatcccgaa
cccgttcaacgtcagc-----------------gaagggatggagaggtgacatccg---atgaggaaattgtttttacc
atcggattggaca---------------agtgttcggaggttcagaaaacgtattgcaatggacctttgttaccagacac
ggattacaatgttgtggtgagactgttcaccgcatctggttatag-----------------------------------
------cgatgccgccgtactcaactttaagac-----------------caaggcggccatcaaggtgaccct------
-------------gatcctggtgagcgtttgcagttgcctgctgctggccttcgtacttggtttgacggtt---------
------------------------------------ctctgggtgcgcaagcgattggcctgg
SeaUrchin1
---------------------------------------gagccatccccagttccggaagactatccctatgaagccaa
tacagttttctctga---------------------gacctctaccacctcgttcgctgtggtgcttccaga--------
------------------cgatttattcagccatgaaa---------------------------acggtgaactttga
cctttgctgtcatcatcacaatagatgataatgatccaactgttagta--------------------------------
--------------------------------------------------------------------------------
--------------gcacagaactgacttatgcggccagagccgagaatgcttaca--tcacagccattgaaatcccata
tccatattcaccatcg----------tttggctctaatcgtagaagacgagcaacgg---atcctccaggtacggtagtc
ataggtgatggga---------------cctgtgcaggtagtcaaaatgaatattgcaatggagatttggatgacaacac
agaatactactatgcgttcagagcatacaatggcatgggtaatgtga----------------------cat--------
------ccagtacatttggacctgtgaaaacag----------------------ttaaagataacacagcggg------
-------------gattgctgctggggtatccgtctcactgatcatcatcatcgtggctgtagtggtagtt--gtcgtgt
```

tcttaaaaagaagacaaccaaaagaaccttcac----cacgcccaagc---------------
SeaUrchin3
---gcacctccaactcctcctgcaga
cagggtggtacgtct---------------tgtttctcatacaggtactaccatgagtgtatcctttgacag--------
------------------tagcatattcagcgatgtct---------------------------acggtcctgtgacta
gatatgccctcctggtagcacaaagcagtccagacaccattccttcgacaccaagtgaccctgccatcaac---------
----
----tggcaagttggctcaggccagccatacc-----------------------------aaaccacgctccttactta
cgctcctttcacaaacggaa---acacgaggggttctatacc
gttggcgctaacaaca------------gctgcacgcaggattcagtgggatactgtaacggacctgtcgtccctctcac
tgattacaggtttgctctacgtgcctacgggtctgatgggaa------------------------------gttcactg
aca---gtgcttggtcaccactctacaactcag----------------------gaattaacaccacttg---------
-------------gtacgctgctgctgtggtttccatcatcatcatcactctcgttctcttactcgtcatgcttctcatc
tcccgtcaatgc---
SeaUrchin4
---gctccaccgtctccacctcgtgg
tgcaactccctcgttggtc------tcccgaggcagcactatcaccatcactttttt-----------------------
------------------caatctctttgatactgcgt---------------------------ttggacgaatcgtgc
gctttgcaatcattgtgcaggaacgtgttgatggcggtaccgtcgtcatcacagcca-----------------------
----------------------aacgtcagaccacatcttcactcacctgggctgaagccagaaggacacg---------
---------------ccctgttccctcctacc-----------------------------agaccacgcccgatgacta
taacccttttgctg--atggggcaggtgtaaccacc
ttcagagttgggtcacagtcct------gtgatcctgatgatcttactgcctattgcaatggggctctaccctggtac
ctcttacagatttgctattagagcgtatggtgtggacggcaa------------------------------gtttgtgg
ata---ctgaattcagctctccatttagaacag----------------------atcccgacagaggaatatttgt---
-------------catcccggcaattgtccttgccatcatggtagtcatcatcctcatcatcttcatggccatgggattt
ggatgc---
facornWorm
--cctccaccgaaaccaactgcttcacgtattac
ggcaaatgttcggtt---------------tgttgagaccaccgcctacacattgcgtattcacctaagtaa--------
------------------taatttatttgatgacagat---------------------------ttggaataattgttg
cttacttagttatcattgctgaggacggaggtgagtattcaccattgccagaggttc-----------------------
--ttccatcatatgatgaagtcattgacagctc---------
---------------gccgtggccaccatacc-----------------------------aaaccagtcagccattcaa
tccatttgagacttta----------tcgattggtagacggcgacgcaatacac------tgcagactgttaattacatc
ataggcgccaagg---------------agaattgtgatgtcagtcttgtttactgtaatggggccttaagaccagttac
agactacaggtatgtcattcgtggctacaatgaacttggtaa------------------------------ctacaccg
ata---cggactggtcacttccacaaaaaacag--att
tagatccattctggattctgtatggtgtcatagcaggattaatattaattggcatcattatactgatatgtatatgttat
tgttgctgttgtagaagaagacgatcatcga----cgcaagactcagacaag-----------
acorworm1
--ccaccacctcctcccagaaa
taacatagttccttcatcctctgagtctgacattgaagtttctcaaacaacaatcacagtattattcacaga--------
------------------tgattattttaatgattcca---------------------------atggaccactcttaa
atttcactgtcattgtgacagaagatggcactgaggaagttgataagcatctgaatggtgaaataccacgtgtatattgt
aaagattgtgaaaatgtaccaggtatagaagaaggagaagtgaagacctgggcagaagtacaaaa---------------
---------------cactccagccagatatc-----------------------------agattgattatccttatga
ctatccagattta-------------------tccagaagaaaacgtgaca---------caacagggattgagattact
attggttctgacgata------------gctgttctacaaaggatgaaggtttctgcaatggtcctttgaaggacggcac
atcatatagatatcattttagagcatacacagatgttgg---------------------------------atactcag
ata---ctgtactttcaggtccaattcgaacaa----------------------gcactaacatgtcatggttgtg---
-------------gttacttcttggtgtattgcttggtgtattgatagctgttgctatcattcttgctatcatttattat
aaacgaagatattatgaagaaactact------------------------------------
ciona
--gcgcccccagtgcctgacacttggcccgaggttatgaa
caaaatcgatgatacttca------acaatggcatctgtgacatcatcgacaataactgttaagaaacc-----------
------------------atgcatgttctccaatgaga---------------------------atggaccgattgcaa
gtctcagtgttataatagcggaggacggaggaaacattgaagccgaaccga-----------------------------
---cgtattggtcgcaggctttccctctgca---------
---------------accttctccaccataca-----------------------------aagtcctagtgagtgaaaa
cccgactgattattgc-------------aacacaagaaaacgtcgatcaa---------taaatgagaaaggcttcgtc
attggtacttcag---------------actgtccgagtgaactcaacacacattgtaacggcccgcttaaatcaagcac
ggattacagggtaaaattccgagccgaaactagtaatggatt------------------------------aaagacag
ata---cggaattttccgaaattatccgaactt-------ctccgagtttcctcgcaacccatatgacgttgtt------
-------------gatcgggattggggctgcaattggattgtttacaattcttgtcgtgatatcagctgta--tttctac
ggttagttatgcaatattcgattcaatatg-------gatttcttaacaatgtattc------
Ciona2
--gcgcctccagtgccggcgttgctaccggaagttattat

ccaacaagacaaagc------------------gattgtgacgtcatcatctgttattgtgaagaaacc-----------
------------------atgcgttttctccgatgaca---------------------------atggaccgcttaaga
gtctaagtgtaatagttgcacaagagggggcgacgcttgacgctgaaccaa-----------------------------
---cttattgggcgaaggcttatagtgagga---------
---------------gccttcaccaccgtata-----------------------------aggttattgtaactgaaga
acctcaaagttattgc-------------aactccagaaacaaacgatcaacac------aaaccaacaatggattcgtc
atcggaacttccg---------------attgttctcgtttgtcgacgtcacaatgcaatggaccactgaaatcaaacac
agaatatcggtttaaatatcgagccgaggccaacaacggcct------------------------------gatgacag
aca---ccgaatattccgaagttttccgaacta-------atccgagctttatcgaagcgcatatgacgttgtt------
-------------agtgtcgattggggcagcacttgggatatttattatacttctgactatatcggtcgca--ttgttac
ga---

PTPRF_human

---------------------cacctggtgtccatccgcacagcccccgacctcctgcctcacaagccgctgcctgcctc
tgcctacatagagga---------------cggccgcttcgatctctccatgccccatgtgcaagacccc----------
------------------tcgcttgtcaggtggttcta--
-cattgttgtggtgcccattgaccgtgtgggcgggagcatgctgacgccaaggtggagcacaccc---------------
---------------------------gaggaactggagctggacgagcttctagaagccatcgagcaaggcggagagga
gcagcggc-------ggcggcggcggcaggca--------------------------gaacgtctgaagccatatgtgg
ctgctcaactggatg---tgctcccggagacctttacc
ttgggggacaaga------------------------agaactaccggggcttctacaaccggcccctgtctccggactt
gagctaccagtgctttgtgcttgcctccttgaaggaa--------------------------------cccatggacca
gaagcgctatgcctccagcccctactcggatga------gatcgtggtccaggtgacaccagcccagcagcaggaggagc
cggagatgctgtgggtgacgggtcccgtgctggcagtcatcctcatcatcctcattgtcatcgccatcctc---------
---------------------------------------ttgttcaaaagg------------

PTPRF_mouse

---------------------cacctggtgtccatccgcactgccccggacctcctaccccagaagccactgcctgcctc
cgcctttatagagga---------------tggccgcttctccctctccatgcctcaagtgcaggacccc----------
------------------tcgctagtcaggtggttcta--
-cattgtggtggtgcccattgaccgtgtgggcgggaacttgctggcaccaagatggaacacacca---------------
---------------------------gaggagttggagctggacgagcttctggaggccatcgagcagggcgaggagaa
acagcgga-------ggcgccggcgccaagca--------------------------gagcggctgaagccttatgtgg
cggcccaagtggatg---tgctccctgacaccttcacc
ctgggggacaaga------------------------agagctaccgcggcttctacaaccggcccctgtctccggatct
gagttaccagtgcttcgttctcgcctccctcaaggaa--------------------------------cccatggacca
gaagcgctacgcctccagcccctactcggacga------gattgtagtccaggtgacgccagcacagcagcaggaggagc
ccgagatgctgtgggtgacaggccctgtcctggcggtcattctcatcatactcattgtcatcgccatcctc---------
---------------------------------------ctgttcaagagg------------

PTPRF_chiken

cag------------------cacctcgtctccatccgcactgcgcctgacgtcttgcaaagcaaacccattgccaccaa
caagtatatccagga---------------aggaaagttcacgcttacccttcccaaagtgcagaccact----------
------------------gtgccagttcggtggtacta--
-cattgtggtcgtgccggcagagca---gagccccagcagcccgacagcgcggtggcggacgcct---------------
---------------------------gatgagatggagctggaccagctgctggaggccataagccagggcagtcagag
caggc----------gccagagacgccaagca--------------------------gacagactcaagccctacattg
ctgcccaagtggacg---tgctgcccgagaccttcacc
ctgggggacgaga------------------------agaactacaagggtttctacaacaagcccctctctcaggacct
gagctaccgctgcttcgtgctggcctcgctggaggat-----------------------------------ggggacac
gaagagatacgcagccagcccctactcagatga------gattgtgatggaattggcttcagcgaagcagcaggatgagc
cagagatgctgtgggtgatgggacctgtcctggctgtgatattaatcatcatcatcgtcatcgctatactc---------
---------------------------------------ctcttcaaaagg------------

PTPRF_fish

cag------------------caggtggtgtccatccgcaccgcgcccgacttgctcaaatccaagccggtcatctacag
ggggcaggaggagga---------------cggcaagctcaccatctctctgaacagagtttgtatctctgatcgctcca
gttgcttaaaattaattataacatgtttttttataacagaa------------------------tcgtatcagtggaca
gatggcgtgtcacaaccttacaacaaaggtctaaagtcatca--------------------------------------
------------------------------------aaagaaggcgcttgcccacgggtgaaagagagcagaaag-----
---------------ccttactctgctcctct-------------------------ctctagcctgcaacttgtcacag
caggacctgaaaatacatttccttcac---
----------------------------tttcccgcaggaattatttaaagttttgcaataataaagttaaaactaatca
ttttaaaagcctggaaatcctgaaaaagaacttgccatatttta------------------------caccctcttcca
gaagacgtttgcggccagcccttactcagaccccataacggtggaggtggtcaacaggatgccgagacagccggaggagc
cggagatgttgtgggtgatggggccggtcttggccgtcatcctcatcatcatcattgtcatcgccatcctg---------
---------------------------------------ctgtttaag---------------

PTPRF_monosiga

gtgatcttgcgtggtccagggcgcgtggcagagcgccgcacgctttcagcgcggcctgaagcaccgcccgcggtcacgag
tgccgttctgcctga---------------tggatatagttcacaatcagcctttg------------------------
------------------ctgtcagctgggtcgcaccc--------------tc---cacgtactcgggtcccatccgtc
ggctctcgctcgtggtcgagccacaaggcagtagcacccatgccgtcgcctcgttggactgcgcctctagc---------

```
------------------------------agctgtgatttcgggacgtggtccgaagcccaggctgccag---------
---------------cagcccgatggcttaca----------------------ttgcgtacagccagacctttaccgag
tcggagggattgggggatc-----------------------------------------tgtctcgaagctcttttgtc
attggtgccgagcgag---------agaaggtcgacgctgacggtgatggttatgtcaatggtcctctcaaagcaggtgc
cacctacaccttccgtctcctcacctgcacgcaaaatggcaacgatgaactgtgccagg---------------------
------ccgccagcctcaacccgaccacggcct-tggcctcgacggcggctgcccccgctgacaatactggcat------
-------------catcgttggagttgtcattgtcatcatcgttctcattgtggccgttcttgtctttgtcatgtggcgg
cgtcgccagaacaaagc-----------------cgctgcgcctgatgaagatttctg-----

;

end;]
```
