# Supplementary material for: Phosphotyrosine phosphatase R3 receptors: Origin, evolution and structural diversification
Source: PLoS One. 2017 Mar 3;12(3):e0172887. doi: 10.1371/journal.pone.0172887 (PMC5336234; doi:10.1371/journal.pone.0172887)
Supplement: S1 Table — The NCBI PBLAST E values were plotted in the table, E values lower than e-80 are shaded. (PDF) [file pone.0172887.s008.pdf]

	PTPRO	PTPRO	PTPRO	PTPRB	PTPRB	PTPRB	PTPRJ	PTPRJ	PTPRJ	PTPRH	PTPRH	PTPRQ	PTPRQ	PTPRQ	LAR PTPRF	PTPRγ PTPRG
	human	chicken	fish	human	chicken	fish	human	chicken	fish	human	fish	human	chicken	fish	human	human
Capsapora	8e-79	2e-74	6e-72	1e-70	2e-71	1e-68	2e-77	2e-75	2e-69	5e-64	2e-74	6e-75	9e-71	2e-72	9e-106	3e-82
sponge	1e-105	9e-106	2e-99	8e-100	9e-92	2e-84	1e-87	8e-84	2e-82	3e-74	7e-80	2e-85	3e-77	2e-78	6e-89	3e-88
annelid	9e-108	2e-108	3e-101	5e-102	3e-95	6e-88	3e-88	5e-86	2e-80	8e-81	2e-83	9e-84	7e-81	3e-77	4e-74	8e-75
Fly PTP10D	5e-100	2e-101	6e-97	1e-109	2e-99	1e-90	9e-88	3e-81	1e-79	3e-81	4e-83	2e-85	4e-82	2e-85	4e-72	1e-76
Fly DPTP4e	1e-96	2e-96	6e-95	3e-107	4e-103	2e-93	1e-87	1e-83	1e-81	4e-81	3e-83	2e-85	9e-85	5e-85	1e-73	3e-75
Fly PTP52F	1e-59	1e-59	6e-60	6e-60	2e-71	---	4e-55	---	---	1e-54	---	5e-60	9e-71	---	3e-57	3e-55
Nematode2	8e-84	8e-83	1e-80	3e-87	3e-82	3e-74	3e-80	2e-85	5e-75	1e-72	2e-78	4e-74	6e-70	---	8e-71	6e-65
Nematode3	7e-90	5e-88	4e-85	2e-93	4e-87	9e-79	3e-86	1e-83	5e-84	3e-77	4e-84	2e-76	3e-73	3e-73	7e-73	5e-69
Sea urchin1	1e-96	2e-96	1e-92	1e-103	2e-93	6e-92	2e-93	1e-88	2e-89	1e-80	3e-89	2e-84	3e-80	6e-76	3e-75	3e-77
Sea urchin3	7e-75	6e-74	1e-73	8e-75	4e-66	---	5e-70	---	---	8e-72	1e-70	2e-69	---	---	7e-67	1e-59
Sea urchin4	2e-66	6e-66	---	8e-74	---	2e-65	1e-65	---	---	3e-73	9e-72	9e-72	5e-67	2e-68	1e-67	5e-64
Acorn worm	6e-68	8e-70	---	4e-76	6e-69	1e-63	3e-76	2e-71	1e-67	7e-62	2e-67	1e-75	2e-69	1e-68	5e-63	2e-56
Acorn worm1	3e-103	1e-103	2e-101	1e-117	3e-107	3e-98	6e-95	2e-92	7e-97	4e-78	1e-93	4e-94	4e-87	4e-85	8e-74	7e-69
ciona	6e-58	2e-58	1e-50	3e-60	3e-55	1e-50	5e-55	3e-51	---	1e-51	1e-51	1e-57	2e-52	5e-52	2e-47	9e-50
Ciona2	1e-55	5e-56	1e-50	2e-55	4e-51	---	7e-56	1e-52	4e-48	1e-51	5e-48	1e-60	6e-56	8e-57	3e-49	7e-49
Ciona1	3e-107	2e-107	3e-101	2e-131	5e-118	1e-111	1e-98	2e-90	5e-89	6e-84	5e-91	3e-86	8e-80	1e-81	3e-72	2e-71
Ciona3	1e-84	3e-78	1e-79	2e-107	3e-97	2e-88	3e-81	8e-75	4e-74	5e-65	2e-72	5e-71	7e-66	2e-66	2e-52	2e-54
PTPRO	0.0	0.0	3e-163	5e-95	4e-89	5e-87	2e-87	4e-85	4e-81	2e-83	7e-84	3e-91	1e-83	9e-87	4e-70	1e-68
PTPRB	5e-95	6e-97	4e-99	0.0	0.0	2e-146	4e-113	4e-111	7e-103	1e-85	2e-94	2e-82	4e-78	3e-80	7e-70	7e-74
PTPRJ	1e-88	9e-81	2e-87	2e-114	7e-105	4e-98	0.0	1e-168	2e-136	8e-93	3e-97	1e-75	2e-71	3e-68	9e-72	6e-73
PTPRH	6e-87	5e-80	6e-79	6e-91	1e-86	9e-80	3e-97	2e-90	3e-91	0.0	6e-101	3e-76	1e-71	2e-67	2e-71	4e-63
PTPRQ	1e-92	1e-93	2e-83	1e-83	3e-78	3e-73	7e-76	2e-78	6e-76	6e-74	1e-74	0.0	0.0	1e-156	1e-75	8e-70
