# Supplementary material for: Phosphotyrosine phosphatase R3 receptors: Origin, evolution and structural diversification
Source: PLoS One. 2017 Mar 3;12(3):e0172887. doi: 10.1371/journal.pone.0172887 (PMC5336234; doi:10.1371/journal.pone.0172887)
Supplement: S2 Table — Organism reporte and NCBI PBLAST E values obtained after blasting the PTP domains of PTP52F (A) and PTP10D (B) in Protostomia (taxid:33317). (PDF) [file pone.0172887.s009.pdf]

# A) PTP52F

**Organism Report** — **E value**

**Drosophila melanogaster** [flies] taxid 7227
gb|ADO33192.1| MIP25208p [Drosophila melanogaster] 0.0
ref|NP_611093.2| Ptp52F [Drosophila melanogaster] 0.0
gb|AAF58051.3| Ptp52F [Drosophila melanogaster] 0.0
gb|AFH41846.1| FI18312p1 [Drosophila melanogaster] 0.0

**Drosophila sechellia** [flies] taxid 7238
ref|XP_002034076.1| GM20086 [Drosophila sechellia] 0.0
gb|EDW48089.1| GM20086 [Drosophila sechellia] 0.0

**Drosophila simulans** [flies] taxid 7240
ref|XP_002081725.1| GD25563 [Drosophila simulans] 0.0
gb|EDX07310.1| GD25563 [Drosophila simulans] 0.0

**Drosophila erecta** [flies] taxid 7220
ref|XP_001975408.1| GG22297 [Drosophila erecta] 0.0
gb|EDV55808.1| GG22297 [Drosophila erecta] 0.0

**Drosophila yakuba** [flies] taxid 7245
ref|XP_002092265.1| GE14093 [Drosophila yakuba] 0.0
gb|EDW91977.1| GE14093 [Drosophila yakuba] 0.0

**Drosophila persimilis** [flies] taxid 7234
ref|XP_002016055.1| GL11392 [Drosophila persimilis] 4e-180
gb|EDW31945.1| GL11392 [Drosophila persimilis] 4e-180

**Drosophila pseudoobscura pseudoobscura** [flies] taxid 46245
ref|XP_001360519.2| GA14851 [Drosophila pseudoobscura pseu... 7e-180
gb|EAL25094.2| GA14851 [Drosophila pseudoobscura pseudoobs... 7e-180

**Drosophila ananassae** [flies] taxid 7217
ref|XP_001959786.1| GF13044 [Drosophila ananassae] 1e-173
gb|EDV36608.1| GF13044 [Drosophila ananassae] 1e-173

**Drosophila mojavensis** [flies] taxid 7230
ref|XP_002005632.1| GI18965 [Drosophila mojavensis] 8e-172
gb|EDW09567.1| GI18965 [Drosophila mojavensis] 8e-172

**Drosophila virilis** [flies] taxid 7244
ref|XP_002049150.1| GJ20908 [Drosophila virilis] 4e-170
gb|EDW60343.1| GJ20908 [Drosophila virilis] 4e-170

**Drosophila willistoni** [flies] taxid 7260
ref|XP_002063690.1| GK15811 [Drosophila willistoni] 5e-170
gb|EDW74676.1| GK15811 [Drosophila willistoni] 5e-170

**Drosophila grimshawi** [flies] taxid 7222
ref|XP_001995168.1| GH22999 [Drosophila grimshawi] 6e-167
gb|EDV98240.1| GH22999 [Drosophila grimshawi] 6e-167

**Musca domestica** [flies] taxid 7370
ref|XP_005185072.1| PREDICTED: phosphatidylinositol phosph... 1e-151
ref|XP_005185071.1| PREDICTED: phosphatidylinositol phosph... 1e-151

**Ceratitis capitata** [flies] taxid 7213
ref|XP_004527553.1| PREDICTED: phosphatidylinositol phosph... 4e-131

**Bactrocera cucurbitae** (melon fruit fly) [flies] taxid 28588
ref|XP_011195089.1| PREDICTED: receptor-type tyrosine-prot... 1e-91

**Anopheles darlingi** [mosquitos] taxid 43151
gb|ETN64617.1| protein-tyrosine phosphatase [Anopheles dar... 2e-71

**Aedes aegypti** [mosquitos] taxid 7159
ref|XP_001651298.1| AAEL000845-PA [Aedes aegypti] 6e-71
gb|EAT48124.1| AAEL000845-PA [Aedes aegypti] 6e-71

**Anopheles sinensis** [mosquitos] taxid 74873
gb|KFB37697.1| AGAP011650-PA-like protein [Anopheles sinen... 1e-70
gb|KFB37699.1| AGAP011650-PA-like protein [Anopheles sinen... 1e-67
gb|KFB37706.1| AGAP011650-PA-like protein [Anopheles sinen... 8e-55

**Anopheles gambiae str. PEST** [mosquitos] taxid 180454
ref|XP_320862.4| AGAP011650-PA [Anopheles gambiae str. PEST] 1e-69
gb|EAA00409.4| AGAP011650-PA [Anopheles gambiae str. PEST] 1e-69

**Harpegnathos saltator** [ants] taxid 610380
ref|XP_011136614.1| PREDICTED: phosphatidylinositol phosph... 9e-63
ref|XP_011136553.1| PREDICTED: phosphatidylinositol phosph... 1e-62
ref|XP_011136564.1| PREDICTED: phosphatidylinositol phosph... 1e-62
ref|XP_011136570.1| PREDICTED: phosphatidylinositol phosph... 1e-62
ref|XP_011136579.1| PREDICTED: phosphatidylinositol phosph... 1e-62
ref|XP_011136598.1| PREDICTED: phosphatidylinositol phosph... 1e-62
ref|XP_011136588.1| PREDICTED: receptor-type tyrosine-prot... 1e-62
ref|XP_011136607.1| PREDICTED: phosphatidylinositol phosph... 1e-62
gb|EFN89816.1| Phosphotidylinositol phosphatase PTPRQ [Har... 7e-59

**Acromyrmex echinatior** [ants] taxid 103372
ref|XP_011054097.1| PREDICTED: tyrosine-protein phosphatas... 1e-62
gb|EGI65741.1| Receptor-type tyrosine-protein phosphatase ... 2e-62

**Pogonomyrmex barbatus** [ants] taxid 144034
ref|XP_011645940.1| PREDICTED: LOW QUALITY PROTEIN: tyrosi... 2e-62
ref|XP_011638691.1| PREDICTED: tyrosine-protein phosphatas... 2e-54
ref|XP_011638689.1| PREDICTED: tyrosine-protein phosphatas... 2e-54
ref|XP_011638690.1| PREDICTED: tyrosine-protein phosphatas... 3e-54
ref|XP_011638683.1| PREDICTED: tyrosine-protein phosphatas... 3e-54
ref|XP_011638681.1| PREDICTED: tyrosine-protein phosphatas... 3e-54
ref|XP_011638682.1| PREDICTED: tyrosine-protein phosphatas... 3e-54
ref|XP_011638681.1| PREDICTED: tyrosine-protein phosphatas... 3e-48
ref|XP_011638682.1| PREDICTED: tyrosine-protein phosphatas... 3e-48
ref|XP_011638686.1| PREDICTED: tyrosine-protein phosphatas... 3e-54

**Solenopsis invicta** (red imported fire ant) [ants] taxid 13686
ref|XP_011175573.1| PREDICTED: tyrosine-protein phosphatas... 2e-62
ref|XP_011175575.1| PREDICTED: tyrosine-protein phosphatas... 2e-62
ref|XP_011175576.1| PREDICTED: tyrosine-protein phosphatas... 3e-62

**Bombus impatiens** [bees] taxid 132113
ref|XP_003490381.1| PREDICTED: receptor-type tyrosine-prot... 1e-61

**Wuchereria bancrofti** [nematodes] taxid 6293
gb|EJW78780.1| ptprd protein, partial [Wuchereria bancrofti] 2e-61

**Camponotus floridanus** [ants] taxid 104421
ref|XP_011263447.1| PREDICTED: tyrosine-protein phosphatas... 6e-61
gb|EFN63464.1| Receptor-type tyrosine-protein phosphatase ... 8e-61
ref|XP_011263446.1| PREDICTED: tyrosine-protein phosphatas... 8e-61
ref|XP_011263445.1| PREDICTED: tyrosine-protein phosphatas... 1e-60
ref|XP_011263443.1| PREDICTED: tyrosine-protein phosphatas... 1e-60
ref|XP_011263444.1| PREDICTED: tyrosine-protein phosphatas... 1e-60

**Zootermopsis nevadensis** [termites] taxid 136037
gb|KDR07555.1| Receptor-type tyrosine-protein phosphatase ... 2e-60

**Brugia malayi** [nematodes] taxid 6279
ref|XP_001894311.1| protein-tyrosine phosphatase Lar-like ... 2e-60
emb|CDQ04589.1| Protein BM-PTP-3, isoform d [Brugia malayi] 2e-58

**Crassostrea gigas** [bivalves] taxid 29159
ref|XP_011431373.1| PREDICTED: receptor-type tyrosine-prot... 7e-60
gb|EKC25397.1| Receptor-type tyrosine-protein phosphatase ... 1e-56
ref|XP_011443225.1| PREDICTED: receptor-type tyrosine-prot... 3e-56
gb|EKC36845.1| Tyrosine-protein phosphatase 10D [Crassostr... 1e-55
ref|XP_011434022.1| PREDICTED: tyrosine-protein phosphatas... 1e-55
ref|XP_011429025.1| PREDICTED: tyrosine-protein phosphatas... 2e-55
ref|XP_011429022.1| PREDICTED: tyrosine-protein phosphatas... 2e-55
ref|XP_011429020.1| PREDICTED: tyrosine-protein phosphatas... 2e-55
ref|XP_011429024.1| PREDICTED: receptor-type tyrosine-prot... 3e-55
ref|XP_011429021.1| PREDICTED: tyrosine-protein phosphatas... 3e-55
ref|XP_011429023.1| PREDICTED: receptor-type tyrosine-prot... 3e-55
ref|XP_011429017.1| PREDICTED: tyrosine-protein phosphatas... 3e-55
ref|XP_011429016.1| PREDICTED: tyrosine-protein phosphatas... 3e-55
gb|EKC35634.1| Tyrosine-protein phosphatase Lar [Crassostr... 3e-55
ref|XP_011429019.1| PREDICTED: tyrosine-protein phosphatas... 3e-55
ref|XP_011456389.1| PREDICTED: receptor-type tyrosine-prot... 1e-54

**Microplitis demolitor** [wasps &c.] taxid 69319
ref|XP_008546124.1| PREDICTED: tyrosine-protein phosphatas... 2e-59
ref|XP_008546121.1| PREDICTED: receptor-type tyrosine-prot... 2e-59
ref|XP_008546122.1| PREDICTED: receptor-type tyrosine-prot... 2e-59
ref|XP_008546123.1| PREDICTED: receptor-type tyrosine-prot... 2e-59
gb|EZA45550.1| protein tyrosine phosphatase-like protein [... 2e-59
ref|XP_008559253.1| PREDICTED: tyrosine-protein phosphatas... 2e-54

**Diaphorina citri** [psyllids] taxid 121845
ref|XP_008475773.1| PREDICTED: tyrosine-protein phosphatas... 2e-59

**Trichuris suis** [nematodes] taxid 68888
gb|KHJ41233.1| Protein-tyrosine phosphatase [Trichuris suis] 4e-59
gb|KHJ45812.1| Protein-tyrosine phosphatase [Trichuris suis] 5e-55
gb|KFD57941.1| hypothetical protein M513_01174 [Trichuris ... 1e-54

**Fopius arisanus** [wasps &c.] taxid 64838
ref|XP_011298186.1| PREDICTED: receptor-type tyrosine-prot... 6e-59
ref|XP_011298182.1| PREDICTED: receptor-type tyrosine-prot... 8e-59
ref|XP_011298183.1| PREDICTED: receptor-type tyrosine-prot... 8e-59
ref|XP_011298185.1| PREDICTED: receptor-type tyrosine-prot... 8e-59

**Megachile rotundata** [bees] taxid 143995
ref|XP_003706492.1| PREDICTED: tyrosine-protein phosphatas... 4e-58

**Loa loa** [nematodes] taxid 7209
gb|EFO22634.2| fibronectin type III domain-containing prot... 4e-58
ref|XP_003141435.1| fibronectin type III domain-containing... 4e-58

**Trichuris trichiura** [nematodes] taxid 36087
emb|CDW53553.1| Y phosphatase domain containing protein [T... 7e-58

**Nasonia vitripennis** [wasps &c.] taxid 7425
ref|XP_008205234.1| PREDICTED: phosphatidylinositol phosph... 7e-58
ref|XP_008205235.1| PREDICTED: phosphatidylinositol phosph... 7e-58
ref|XP_008205236.1| PREDICTED: phosphatidylinositol phosph... 8e-58

**Tribolium castaneum** (rust-red flour) [beetles] taxid 7070
ref|XP_008192255.1| PREDICTED: receptor-type tyrosine-prot... 9e-58
gb|EFA01732.1| hypothetical protein TcasGA2_TC007325 [Trib... 4e-57

**Trichinella spiralis** [nematodes] taxid 6334
ref|XP_003372931.1| putative fibronectin type III domain p... 2e-57
gb|EFV52927.1| putative fibronectin type III domain protei... 2e-57
ref|XP_003372931.1| putative fibronectin type III domain p... 2e-44
gb|EFV52927.1| putative fibronectin type III domain protei... 2e-44

**Lottia gigantea** [gastropods] taxid 225164
ref|XP_009065639.1| hypothetical protein LOTGIDRAFT_133136... 4e-57
gb|ESO83607.1| hypothetical protein LOTGIDRAFT_133136, par... 4e-57
ref|XP_009062602.1| hypothetical protein LOTGIDRAFT_128794... 1e-55
gb|ESO86619.1| hypothetical protein LOTGIDRAFT_128794, par... 1e-55
ref|XP_009062602.1| hypothetical protein LOTGIDRAFT_128794... 2e-49
gb|ESO86619.1| hypothetical protein LOTGIDRAFT_128794, par... 2e-49

**Ceratosolen solmsi marchali** [wasps &c.] taxid 326594
ref|XP_011502139.1| PREDICTED: phosphatidylinositol phosph... 7e-57

**Helobdella robusta** [segmented worms] taxid 6412
ref|XP_009014877.1| hypothetical protein HELRODRAFT_98742 ... 3e-56
gb|ESO06781.1| hypothetical protein HELRODRAFT_98742 [Helo... 3e-56
ref|XP_009014877.1| hypothetical protein HELRODRAFT_98742 ... 3e-45
gb|ESO06781.1| hypothetical protein HELRODRAFT_98742 [Helo... 3e-45

**Ancylostoma ceylanicum** [nematodes] taxid 53326
gb|EPB68150.1| Protein-tyrosine phosphatase [Ancylostoma c... 1e-55
gb|EYC30618.1| hypothetical protein Y032_0005g2754 [Ancylo... 1e-55
gb|EYC30619.1| hypothetical protein Y032_0005g2754 [Ancylo... 1e-55
gb|EYC30620.1| hypothetical protein Y032_0005g2754 [Ancylo... 2e-55

**Oesophagostomum dentatum** [nematodes] taxid 61180
gb|KHJ98251.1| Protein-tyrosine phosphatase [Oesophagostom... 2e-55

**Capitella teleta** [segmented worms] taxid 283909
gb|ELT95888.1| hypothetical protein CAPTEDRAFT_120303, par... 2e-55

**Haemonchus contortus** (red stomach worm) [nematodes] taxid 6289
emb|CDJ82921.1| Fibronectin and Protein-tyrosine phosphata... 2e-55

**Necator americanus** [nematodes] taxid 51031
gb|ETN81441.1| Protein-tyrosine phosphatase [Necator ameri... 4e-55

**Toxocara canis** [nematodes] taxid 6265
gb|KHN75199.1| Tyrosine-protein phosphatase Lar-like [Toxo... 7e-55

**Stegodyphus mimosarum** [spiders] taxid 407821
gb|KFM66889.1| Tyrosine-protein phosphatase Lar, partial [... 9e-55

**Bombus terrestris** (large earth bumblebee) [bees] taxid 30195
ref|XP_003396194.1| PREDICTED: receptor-type tyrosine-prot... 3e-54

# B) PTP10D

```
                    Organism Report                              E value

Drosophila melanogaster [flies] taxid 7227
 pdb|3S3E|A Chain A, Crystal Structure Of The Catalytic Dom...    0.0
 pdb|3S3E|B Chain B, Crystal Structure Of The Catalytic Dom...    0.0
 pdb|3S3F|A Chain A, Crystal Structure Of The Catalytic Dom...    0.0
 pdb|3S3F|B Chain B, Crystal Structure Of The Catalytic Dom...    0.0
 pdb|3S3H|A Chain A, Crystal Structure Of The Catalytic Dom...    0.0
 pdb|3S3H|B Chain B, Crystal Structure Of The Catalytic Dom...    0.0
 pdb|3S3K|A Chain A, Crystal Structure Of The Catalytic Dom...    0.0
 pdb|3S3K|B Chain B, Crystal Structure Of The Catalytic Dom...    0.0
 gb|AAA28952.1| receptor-linked protein tyrosine phosphatas...    0.0
 ref|NP_001138187.1| protein tyrosine phosphatase 10D, isof...    0.0
 ref|NP_001259454.1| protein tyrosine phosphatase 10D, isof...    0.0
 gb|ACL82919.1| protein tyrosine phosphatase 10D, isoform E...    0.0
 gb|AGB95297.1| protein tyrosine phosphatase 10D, isoform I...    0.0
 gb|AAA28484.1| protein tyrosine phosphatase [Drosophila me...    0.0
 ref|NP_727544.2| protein tyrosine phosphatase 10D, isoform...    0.0
 gb|AAF48072.3| protein tyrosine phosphatase 10D, isoform B...    0.0
 ref|NP_001259453.1| protein tyrosine phosphatase 10D, isof...    0.0
 gb|AGB95296.1| protein tyrosine phosphatase 10D, isoform H...    0.0
 gb|AAO42638.1| RE52018p [Drosophila melanogaster]                0.0
 ref|NP_996413.2| protein tyrosine phosphatase 10D, isoform...    0.0
 ref|NP_996414.2| protein tyrosine phosphatase 10D, isoform...    0.0
 sp|P35992.4|PTP10_DROME RecName: Full=Tyrosine-protein pho...    0.0
 gb|AAS65320.2| protein tyrosine phosphatase 10D, isoform F...    0.0
 gb|AAS65319.2| protein tyrosine phosphatase 10D, isoform G...    0.0
 ref|NP_001162671.2| protein tyrosine phosphatase 4E, isofo...    6e-171
 gb|ACZ95208.2| protein tyrosine phosphatase 4E, isoform G ...    6e-171
 ref|NP_726967.1| protein tyrosine phosphatase 4E, isoform ...    6e-171
 gb|AAN09133.1| protein tyrosine phosphatase 4E, isoform B ...    6e-171
 ref|NP_525076.2| protein tyrosine phosphatase 4E, isoform ...    4e-170
 ref|NP_001162669.1| protein tyrosine phosphatase 4E, isofo...    4e-170
 ref|NP_001162670.1| protein tyrosine phosphatase 4E, isofo...    4e-170
 ref|NP_001162672.1| protein tyrosine phosphatase 4E, isofo...    4e-170
 gb|AAF45998.1| protein tyrosine phosphatase 4E, isoform A ...    4e-170
 gb|ACE82587.1| RE30833p [Drosophila melanogaster]                4e-170
 gb|ACZ95206.1| protein tyrosine phosphatase 4E, isoform C ...    4e-170
 gb|ACZ95207.1| protein tyrosine phosphatase 4E, isoform D ...    4e-170
 gb|ACZ95209.1| protein tyrosine phosphatase 4E, isoform F ...    4e-170
 ref|NP_001284875.1| protein tyrosine phosphatase 4E, isofo...    4e-170
 gb|AHN59346.1| protein tyrosine phosphatase 4E, isoform H ...    4e-170
 gb|AAA76834.1| receptor protein tyrosine phosphatase [Dros...    1e-168
 gb|AAM11067.1| GH15539p [Drosophila melanogaster]                3e-162

  Drosophila persimilis [flies] taxid 7234
 ref|XP_002022579.1| GL13110 [Drosophila persimilis]              0.0
 gb|EDW26614.1| GL13110 [Drosophila persimilis]                   0.0
 ref|XP_002024427.1| GL15029 [Drosophila persimilis]              1e-174
 gb|EDW29843.1| GL15029 [Drosophila persimilis]                   1e-174

  Drosophila willistoni [flies] taxid 7260
 ref|XP_002071311.1| GK25195 [Drosophila willistoni]              0.0
 gb|EDW82297.1| GK25195 [Drosophila willistoni]                   0.0
 ref|XP_002064297.1| GK19778 [Drosophila willistoni]              1e-171
 gb|EDW75283.1| GK19778 [Drosophila willistoni]                   1e-171

  Drosophila mojavensis [flies] taxid 7230
 ref|XP_002011039.1| GI16226 [Drosophila mojavensis]              0.0
 gb|EDW05881.1| GI16226 [Drosophila mojavensis]                   0.0
 ref|XP_002010656.1| GI21662 [Drosophila mojavensis]              2e-173
```

gb|EDW06311.1| GI21662 [Drosophila mojavensis] 2e-173

**Drosophila yakuba** [flies] taxid 7245
ref|XP_002101393.1| GE15658 [Drosophila yakuba] 0.0
gb|EDX02501.1| GE15658 [Drosophila yakuba] 0.0
ref|XP_002100047.1| GE16377 [Drosophila yakuba] 2e-169
gb|EDX01155.1| GE16377 [Drosophila yakuba] 2e-169

**Drosophila sechellia** [flies] taxid 7238
ref|XP_002044105.1| GM13099 [Drosophila sechellia] 0.0
gb|EDW51417.1| GM13099 [Drosophila sechellia] 0.0
ref|XP_002036958.1| GM12377 [Drosophila sechellia] 1e-170
gb|EDW53117.1| GM12377 [Drosophila sechellia] 1e-170

**Drosophila ananassae** [flies] taxid 7217
ref|XP_001966042.1| GF19443 [Drosophila ananassae] 0.0
gb|EDV38451.1| GF19443 [Drosophila ananassae] 0.0
ref|XP_001963349.1| GF20352 [Drosophila ananassae] 5e-169
gb|EDV44425.1| GF20352 [Drosophila ananassae] 5e-169

**Drosophila erecta** [flies] taxid 7220
ref|XP_001977094.1| GG18436 [Drosophila erecta] 0.0
gb|EDV46021.1| GG18436 [Drosophila erecta] 0.0
ref|XP_001976932.1| GG18737 [Drosophila erecta] 2e-169
gb|EDV45859.1| GG18737 [Drosophila erecta] 2e-169

**Drosophila pseudoobscura pseudoobscura** [flies] taxid 46245
ref|XP_001355649.2| GA14821 [Drosophila pseudoobscura pseu... 0.0
gb|EAL32708.2| GA14821 [Drosophila pseudoobscura pseudoobs... 0.0
ref|XP_001354911.2| GA19940 [Drosophila pseudoobscura pseu... 2e-169
gb|EAL31967.2| GA19940 [Drosophila pseudoobscura pseudoobs... 2e-169

**Drosophila virilis** [flies] taxid 7244
ref|XP_002058131.1| GJ15918 [Drosophila virilis] 0.0
gb|EDW66239.1| GJ15918 [Drosophila virilis] 0.0
ref|XP_002057923.1| GJ15786 [Drosophila virilis] 6e-174
gb|EDW66031.1| GJ15786 [Drosophila virilis] 6e-174

**Bactrocera cucurbitae** (melon fruit fly) [flies] taxid 28588
ref|XP_011181681.1| PREDICTED: tyrosine-protein phosphatas... 0.0

**Drosophila grimshawi** [flies] taxid 7222
ref|XP_001991335.1| GH12596 [Drosophila grimshawi] 0.0
gb|EDV99959.1| GH12596 [Drosophila grimshawi] 0.0
ref|XP_001992309.1| GH24682 [Drosophila grimshawi] 2e-172
gb|EDV92016.1| GH24682 [Drosophila grimshawi] 2e-172

**Ceratitis capitata** [flies] taxid 7213
ref|XP_004527471.1| PREDICTED: LOW QUALITY PROTEIN: tyrosi... 0.0

**Bactrocera dorsalis** [flies] taxid 27457
ref|XP_011201536.1| PREDICTED: LOW QUALITY PROTEIN: tyrosi... 0.0

**Musca domestica** [flies] taxid 7370
ref|XP_005183225.1| PREDICTED: LOW QUALITY PROTEIN: tyrosi... 0.0

**Aedes aegypti** [mosquitos] taxid 7159
ref|XP_001662235.1| AAEL012083-PA, partial [Aedes aegypti] 0.0
gb|EAT35785.1| AAEL012083-PA, partial [Aedes aegypti] 0.0

**Anopheles gambiae str. PEST** [mosquitos] taxid 180454
ref|XP_313165.5| AGAP004246-PA [Anopheles gambiae str. PEST] 0.0
gb|EAA08669.6| AGAP004246-PA [Anopheles gambiae str. PEST] 0.0
ref|XP_003436753.1| AGAP004246-PB [Anopheles gambiae str. ... 0.0
gb|EGK97027.1| AGAP004246-PB [Anopheles gambiae str. PEST] 0.0

**Culex quinquefasciatus** [mosquitos] taxid 7176
ref|XP_001847466.1| receptor protein-tyrosine phosphatase ... 0.0

gb|EDS26250.1| receptor protein-tyrosine phosphatase 10d [...    0.0

**Anopheles darlingi** [mosquitos] taxid 43151
gb|ETN63979.1| protein-tyrosine phosphatase [Anopheles dar...    0.0

**Zootermopsis nevadensis** [termites] taxid 136037
gb|KDR19048.1| Tyrosine-protein phosphatase 10D, partial [...    2e-179

**Dendroctonus ponderosae** [beetles] taxid 77166
gb|ENN80085.1| hypothetical protein YQE_03484, partial [De...    5e-177
gb|ERL89353.1| hypothetical protein D910_06724 [Dendrocton...    3e-176

**Tribolium castaneum** (rust-red flour beetle) [beetles] taxid 7070
ref|XP_974913.2| PREDICTED: tyrosine-protein phosphatase 1...    7e-177
gb|EFA02044.1| hypothetical protein TcasGA2_TC007672 [Trib...    9e-177
ref|XP_008191712.1| PREDICTED: tyrosine-protein phosphatas...    1e-176
ref|XP_008191713.1| PREDICTED: tyrosine-protein phosphatas...    1e-176

**Pogonomyrmex barbatus** [ants] taxid 144034
ref|XP_011639069.1| PREDICTED: tyrosine-protein phosphatas...    3e-176
ref|XP_011639070.1| PREDICTED: tyrosine-protein phosphatas...    3e-176
ref|XP_011639071.1| PREDICTED: tyrosine-protein phosphatas...    4e-176
ref|XP_011639072.1| PREDICTED: tyrosine-protein phosphatas...    6e-175

**Ceratosolen solmsi marchali** [wasps &c.] taxid 326594
ref|XP_011501250.1| PREDICTED: tyrosine-protein phosphatas...    4e-176

**Camponotus floridanus** [ants] taxid 104421
ref|XP_011258051.1| PREDICTED: tyrosine-protein phosphatas...    6e-176
ref|XP_011258050.1| PREDICTED: tyrosine-protein phosphatas...    2e-175
ref|XP_011258049.1| PREDICTED: tyrosine-protein phosphatas...    2e-175
ref|XP_011258048.1| PREDICTED: tyrosine-protein phosphatas...    2e-175
gb|EFN67223.1| Tyrosine-protein phosphatase 10D [Camponotu...    6e-175

**Acromyrmex echinatior** [ants] taxid 103372
ref|XP_011056325.1| PREDICTED: tyrosine-protein phosphatas...    2e-175
gb|EGI64344.1| Tyrosine-protein phosphatase 10D [Acromyrme...    3e-174
ref|XP_011056322.1| PREDICTED: tyrosine-protein phosphatas...    3e-174
ref|XP_011056321.1| PREDICTED: tyrosine-protein phosphatas...    3e-174
ref|XP_011056324.1| PREDICTED: tyrosine-protein phosphatas...    3e-174
ref|XP_011056323.1| PREDICTED: tyrosine-protein phosphatas...    4e-174

**Cerapachys biroi** [ants] taxid 443821
gb|EZA56383.1| Tyrosine-protein phosphatase 10D [Cerapachy...    3e-175
ref|XP_011335665.1| PREDICTED: LOW QUALITY PROTEIN: tyrosi...    7e-175

**Solenopsis invicta** (red imported fire ant, ...) [ants] taxid 13686
gb|EFZ20845.1| hypothetical protein SINV_13485, partial [S...    4e-175
ref|XP_011155396.1| PREDICTED: tyrosine-protein phosphatas...    5e-175
ref|XP_011155397.1| PREDICTED: tyrosine-protein phosphatas...    6e-175

**Harpegnathos saltator** [ants] taxid 610380
ref|XP_011138009.1| PREDICTED: tyrosine-protein phosphatas...    5e-175

**Fopius arisanus** [wasps &c.] taxid 64838
ref|XP_011298899.1| PREDICTED: tyrosine-protein phosphatas...    6e-175
ref|XP_011298900.1| PREDICTED: tyrosine-protein phosphatas...    9e-175

**Megachile rotundata** [bees] taxid 143995
ref|XP_003700732.1| PREDICTED: tyrosine-protein phosphatas...    7e-175

**Microplitis demolitor** [wasps &c.] taxid 69319
ref|XP_008553329.1| PREDICTED: tyrosine-protein phosphatas...    8e-175
ref|XP_008553330.1| PREDICTED: tyrosine-protein phosphatas...    1e-174

**Apis dorsata** (rock honeybee) [bees] taxid 7462
ref|XP_006610768.1| PREDICTED: tyrosine-protein phosphatas...    1e-174

**Apis florea** (dwarf honeybee) [bees] taxid 7463
ref|XP_003689555.1| PREDICTED: LOW QUALITY PROTEIN: tyrosi... 3e-174

**Bombus impatiens** [bees] taxid 132113
ref|XP_003491955.1| PREDICTED: tyrosine-protein phosphatas... 6e-174

**Bombus terrestris** (large earth bumblebee) [bees] taxid 30195
ref|XP_003399784.1| PREDICTED: tyrosine-protein phosphatas... 1e-173

**Apis mellifera** (honeybee, ...) [bees] taxid 7460
ref|XP_006562444.1| PREDICTED: tyrosine-protein phosphatas... 1e-173

**Pediculus humanus corporis** (human body lice) [lice] taxid 121224
ref|XP_002431484.1| tyrosine-protein phosphatase 10D precu... 7e-173
gb|EEB18746.1| tyrosine-protein phosphatase 10D precursor,... 7e-173

**Drosophila simulans** [flies] taxid 7240
ref|XP_002106166.1| GD16715 [Drosophila simulans] 3e-170
gb|EDX17114.1| GD16715 [Drosophila simulans] 3e-170

**Danaus plexippus** (American monarch) [butterflies] taxid 13037
gb|EHJ68696.1| receptor protein-tyrosine phosphatase 10d [... 6e-164

**Nasonia vitripennis** [wasps &c.] taxid 7425
ref|XP_008203387.1| PREDICTED: tyrosine-protein phosphatas... 7e-163
ref|XP_008203384.1| PREDICTED: tyrosine-protein phosphatas... 8e-163
ref|XP_008203383.1| PREDICTED: tyrosine-protein phosphatas... 8e-163
ref|XP_008203382.1| PREDICTED: tyrosine-protein phosphatas... 9e-163
ref|XP_008203386.1| PREDICTED: tyrosine-protein phosphatas... 9e-163
ref|XP_008203380.1| PREDICTED: tyrosine-protein phosphatas... 1e-162
ref|XP_008203381.1| PREDICTED: tyrosine-protein phosphatas... 1e-162
ref|XP_001601370.3| PREDICTED: tyrosine-protein phosphatas... 1e-162
ref|XP_008203385.1| PREDICTED: tyrosine-protein phosphatas... 1e-162

**Daphnia pulex** [crustaceans] taxid 6669
gb|EFX76960.1| hypothetical protein DAPPUDRAFT_306015 [Dap... 2e-158

**Stegodyphus mimosarum** [spiders] taxid 407821
gb|KFM77963.1| Tyrosine-protein phosphatase 10D, partial [... 7e-158
gb|KFM71044.1| Tyrosine-protein phosphatase 10D, partial [... 2e-157

**Metaseiulus occidentalis** [mites & ticks] taxid 34638
ref|XP_003747088.1| PREDICTED: tyrosine-protein phosphatas... 3e-154

**Acyrthosiphon pisum** [aphids] taxid 7029
ref|XP_001947832.2| PREDICTED: tyrosine-protein phosphatas... 7e-146

**Trichuris suis** [nematodes] taxid 68888
gb|KHJ41233.1| Protein-tyrosine phosphatase [Trichuris suis] 4e-132
